# Predicting Functions of Proteins in Mouse Based on Weighted Protein-Protein Interaction Network and Protein Hybrid Properties

**DOI:** 10.1371/journal.pone.0014556

**Published:** 2011-01-19

**Authors:** Lele Hu, Tao Huang, Xiaohe Shi, Wen-Cong Lu, Yu-Dong Cai, Kuo-Chen Chou

**Affiliations:** 1 Institute of Systems Biology, Shanghai University, Shanghai, China; 2 Department of Chemistry, College of Sciences, Shanghai University, Shanghai, China; 3 Key Laboratory of Systems Biology, Shanghai Institutes for Biological Sciences, Chinese Academy of Sciences, Shanghai, China; 4 Shanghai Center for Bioinformation Technology, Shanghai, China; 5 Institute of Health Sciences, Shanghai Institutes for Biological Sciences, Chinese Academy of Sciences and Shanghai Jiao Tong University School of Medicine, Shanghai, China; 6 Centre for Computational Systems Biology, Fudan University, Shanghai, China; 7 Gordon Life Science Institute, San Diego, California, United States of America; King's College London, United Kingdom

## Abstract

**Background:**

With the huge amount of uncharacterized protein sequences generated in the post-genomic age, it is highly desirable to develop effective computational methods for quickly and accurately predicting their functions. The information thus obtained would be very useful for both basic research and drug development in a timely manner.

**Methodology/Principal Findings:**

Although many efforts have been made in this regard, most of them were based on either sequence similarity or protein-protein interaction (PPI) information. However, the former often fails to work if a query protein has no or very little sequence similarity to any function-known proteins, while the latter had similar problem if the relevant PPI information is not available. In view of this, a new approach is proposed by hybridizing the PPI information and the biochemical/physicochemical features of protein sequences. The overall first-order success rates by the new predictor for the functions of mouse proteins on training set and test set were 69.1% and 70.2%, respectively, and the success rate covered by the results of the top-4 order from a total of 24 orders was 65.2%.

**Conclusions/Significance:**

The results indicate that the new approach is quite promising that may open a new avenue or direction for addressing the difficult and complicated problem.

## Introduction

With the rapid growth of genome sequences and gene expression profiles, there is increasing concern about using computational methods to determine the linkages between protein sequences and their biological functions [1], [2], [3], [4]. This is because experimental determination of protein functions is not only expensive but also time-consuming and hence can no longer catch up with the pace of the fast growth of newly found proteins. In this paper, we are to propose a computational method to tackle the problem by studying the functions of proteins in mouse, one of the most extensively studied organisms. On one side, functions of many proteins of mouse are already known, which can help us establish a statistical predictor with solid training dataset. On the other side, thousands of proteins of mouse still lack functional annotation [5] and it would be beneficial if a well-performed predictor can be developed to provide us with their possible functions in a timely manner, particularly for drug target selecting and screening [4].

The most established approaches [6] for protein function prediction are based on sequence similarity using BLAST [7], sequence motifs such as PROSITE [8], profile methods such as PFAM [9] and PSI-BLAST [7], and structure based method such as FATCAT [10] and ProCAT [10]. However, estimates based on 2 million known proteins suggested that about 33% of proteins with unknown function were closely related to well-characterized homologues and could be effectively targeted by these methods [11]. Because protein-protein interaction (PPI) data sets are becoming increasingly available for more and more organisms, using PPI data to assign protein function has also been extensively studied. Algorithms based on PPI data suggest that proteins with short distances to each other in the network are likely to share the common biological functions [12], [13], [14], [15], and interactive neighbors are more likely to have the same biological functions than non-interactive ones [16], [17]. This is because the query protein and its interactive proteins may form a protein complex to perform a particular function. In a recent review [12], R Sharan *et al* described two main classes of the network-based methods for predicting protein functions: direct methods such as neighborhood counting based method [18],Graph theoretic methods [19], [20]; module-assisted methods such as hierarchical clustering-based methods [21], [22], graph clustering methods [23], [24]. However, some few researches were carried out by only considering binary interactions [25], [26], [27] in this regard; i.e., whether they are interactive with each other or not, without considering the likelihood of the occurring of these interactions. Actually, such likelihood is often associated with the interaction strengths. Another problem is that some proteins still lack interaction data, preventing them from being predicted and analyzed. In view of this, in the current study we are to adopt a weighted interaction network instead of binary one. For those proteins that do not have PPI data, the hybrid properties of proteins, including their biochemical and physicochemical properties, are used to code the protein samples for prediction. Because such coding treatments have been successfully used to improve the quality in predicting many other protein attributes, such as membrane protein type [28], protein subcellular locations [29], and protein complexes [30].

A total of 24 functional categories are adopted in this paper. Each protein is predicted as belonging to some of these 24 functional categories. Here, we are concerned about not only the first-order predicted function - the most likely function predicted by the prediction model, but also the lower-order functions sorted by the prediction criteria. As a result, the accuracies of the first-order function prediction for the overall training set and test set were 69.1% and 70.2%, respectively.

## Materials and Methods

### Data set

The dataset for studying the functions of proteins in mouse here was downloaded from MfunGD [31] (MOUSE Functional Genome Database, ftp://ftpmips.gsf.de/MfunGD/). There are a total of 24 function categories from FunCat [32], which are collected from manually annotation in the literature and GO annotation [33], [34]. Among the 42,682 proteins obtained, there were 14,732 proteins with both sequence and function information, constituting the benchmark dataset. These proteins belonged to 24 functional categories. The number of proteins in each of the 24 categories is shown in **Table 1**, from which we found that most proteins perform more than one function.

**Table 1:** Functional classification of proteins of mouse.

Functional number	Functional Category	Number of proteins
1	METABOLISM	2714
2	ENERGY	605
3	CELL CYCLE AND DNA PROCESSING	1123
4	TRANSCRIPTION	2128
5	PROTEIN SYNTHESIS	490
6	PROTEIN FATE (folding, modification, destination)	2490
7	PROTEIN WITH BINDING FUNCTION OR COFACTOR REQUIREMENT (structural or catalytic)	8414
8	REGULATION OF METABOLISM AND PROTEIN FUNCTION	1115
9	CELLULAR TRANSPORT, TRANSPORT FACILITIES AND TRANSPORT ROUTES	2411
10	CELLULAR COMMUNICATION/SIGNAL TRANSDUCTION MECHANISM	4077
11	CELL RESCUE, DEFENSE AND VIRULENCE	778
12	INTERACTION WITH THE ENVIRONMENT	1492
13	SYSTEMIC INTERACTION WITH THE ENVIRONMENT	2086
14	TRANSPOSABLE ELEMENTS, VIRAL AND PLASMID PROTEINS	11
15	CELL FATE	1313
16	DEVELOPMENT (Systemic)	1044
17	BIOGENESIS OF CELLULAR COMPONENTS	980
18	CELL TYPE DIFFERENTIATION	370
19	TISSUE DIFFERENTIATION	426
20	ORGAN DIFFERENTIATION	559
21	SUBCELLULAR LOCALIZATION	9767
22	CELL TYPE LOCALIZATION	274
23	TISSUE LOCALIZATION	366
24	ORGAN LOCALIZATION	620

The interaction network takes proteins as its nodes, with an edge between two proteins if they interact with each other. The initial weighted PPI network was retrieved from STRING [35] (http://string.embl.de/), which is a large database of known and predicted protein interactions. These interactions contain direct (physical) and indirect (functional) interactions, derived from numerous sources such as experimental repositories, computational prediction methods. In the network, each edge is marked with a score as the edge weight to quantify the interaction confidence, i.e., the likelihood that an interaction occurs.

Then the obtained 14,732 proteins were separated into two subsets: (A) 10,194 proteins in the above PPI network for training and testing the network-based method (see the following section); (B) 4,538 proteins not in the PPI network for training and testing hybrid-property based method (see the following section). For subset A, 1,076 proteins were randomly selected as the independent test set denoted by 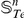 for network-based method, the remaining 9,118 proteins were comprised of training set 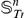. Because the initial network was divided into two parts, some edges were removed, causing a few proteins to drop the connection to all their neighbors in the initial network. Such proteins losing PPI information should be taken away from the training set and test set for network-based method. As a result, 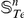 consisted of 1,074 proteins and 88,960 interactions, and 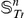 consisted of 9,093 proteins and 742,200 interactions. For subset B, the homologous proteins were removed by CLUSTAL-W [36] to keep any two proteins having lower than 50% sequence identity, and then 248 proteins were randomly selected to constitute the test set 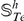 for hybrid-property based method, the remaining 2,905 proteins were assigned as training set 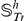. These four datasets can be found in Table S1, Table S2, Table S3, and Table S4.

### Network-based method

Firstly the proteins in the MfunGD [31] database and those in the STRING [35] database need to be registered with each other to construct the weighted PPI interaction network. Then the functions of a query protein can be predicted by using the interaction network according to some criteria.

#### Network mapping

The protein IDs in MfunGD were different from those, the ensemble protein IDs in STRING. To convert MfunGD IDs to ensemble IDs, the ID (the MGI ID [37]) of each MfunGD protein was mapped to the ensemble ID by applying BioMart [38] to get the corresponding ensemble IDs from the MGI IDs.

#### Prediction with PPI information

Towards a query protein in the PPI network, we care not only about its neighbor proteins, but also about the weights of the interactions. Generally, let us consider a PPI network in which proteins belong to 24 functions (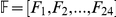), where 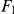 denotes the “METABOLISM”, 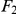 the “ENERGY”, 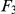 the “CELL CYCLE AND DNA PROCESSING”, and so forth (cf. **Table 1**). Suppose the network consists of 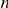 proteins 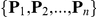, in which the functions of the 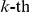 protein is denoted by
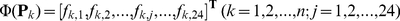
(1)where 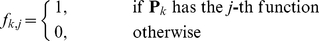
(2)


For a query protein 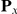, we define its interaction with the proteins in the PPI network like this
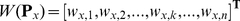
(3)where 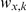 represents the interaction confidence score [35] between 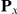 and the 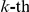 protein in the network; when there is no interaction between them, we have 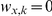. By default, we also have 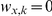 if 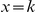 since there is no self-interaction in the network. Here, let us introduce a new concept, the so-called “inclined potential” of protein 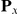 to the 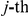
function, as can be formulated by 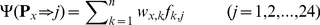
(4)where 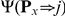 is the “inclined potential” of protein 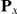 to the 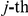
function in the PPI network. Therefore, the larger the value of 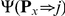, the more likely the protein 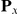 performs the 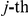
function. In other words, the most likely function of the protein 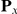 can be predicted as the 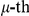 function if 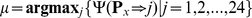
(5)where 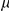 represents the argument of 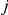 that maximizes the value of 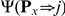. However, most proteins in vivo often perform more than one function, the prediction with only one candidate function is not sufficient. In view of this, to make the predictor capable to deal with proteins with multiple functions and provide experimental biologists with more flexible information in prioritizing candidate targets, let us introduce a 24-D (dimensional vector) to reflect the probability with which the query protein may perform each of the 24 functions, as formulated as follows
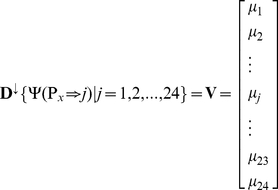
(6)where 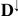 is a descending operator to arrange the 24 scores of 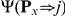 according to the descending order; i.e., 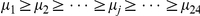. Accordingly, if 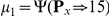, 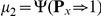, 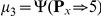, …, then that the query protein 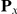 performs the 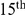
function (CELL FATE) will have the highest likelihood, that 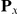 performs the 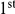
function (METABOLISM) will have the second highest likelihood, that 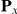 performs the 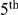
function (PROTEIN SYNTHESIS) will have the third highest likelihood, and so forth (cf. **Table 1**). In rare case when more than one element in Eq.6 has the same score, their order will be assigned randomly. According to the descending order of Eq.6, the predicted results are respectively called the 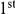-order result, the 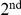-order result, the 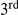-order result, and so forth.

### Hybrid-property approach

Firstly, each protein is coded into feature vector using the hybrid properties. Secondly the features are sorted in descending order by mRMR method. Thirdly, Incremental Feature Selection Method was applied to select the best-performing predictor constructed by Nearest Neighbor Algorithm.

#### Biochemical and physicochemical description of proteins

Many studies have indicated that the success rates for predicting protein attributes could be remarkably improved by incorporating various biochemical or physicochemical properties into the descriptors of protein samples [39], [40] and a long list of relevant references cited in a recent review [41]). Therefore, the biochemical and physicochemical properties (hybrid properties) of proteins are used to code proteins. As the name suggests, it consists of two kinds of properties: (1) Biochemical properties, including two features: amino acid composition, and secondary structural propensity; (2) Physicochemical properties, including five features: polarizability, solvent accessibility, normalized van der Waals volume, and polarity [40].

Of the above seven features, except for the amino acid composition that is an extended quantity to reflect the entire protein, the rest are local quantities to reflect each of the constituent amino acids. Each of such local feature quantities can be classified into two or three groups. For instance, for each amino acid, the secondary structural propensity is characterized as three groups: helix, sheet and coil as predicted by Predator [42]; the hydrophobicity is classified into polar, neutral, or hydrophobic [43]; the solvent accessibility is marked as buried or exposed to solvent by PredAcc [44].

The biochemical or physicochemical character of a protein sequence can be reflected through each of these local feature properties. For instance, using P, N and H to represent the three groups of hydrophobicity: polar, neutral, and hydrophobic, a given protein sequence “MSDKPDMAEIEKFSKETIEQEKQAGESTQEKNPLPMLLPATDKSKLKKTE” can be transformed into “HNPPNPHNPHPPHNPPNHPPPPPNNPNNPPPPNHNHHHNNNPPNPHPPNP”.

For each such letter sequence, three properties can be obtained: composition (C), transition (T), and distribution (D). C describes the global percent composition of each of the groups in the letter sequence; T, the percent frequencies with which the letter changes to another along the entire length of the letter sequence; and D, the distribution pattern of the letters along the sequence, measuring the percentage of the sequence length within which the first, 25%, 50%, 75%, and 100% of the amino acids of each letter is located.

Take the 50-length letter sequence described above as an example. It is composed of 10 Hs, 16Ns, 24Ps. The first feature C is 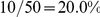, 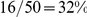, and 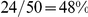 for H, N and P, respectively. For the feature T, there are totally 31 transitions in the sequence, with 8 between H and N, 16 between N and P, and 7 between H and P, so the feature T can be calculated as 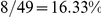, 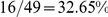 and 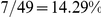, respectively. The first, 25%, 50%, 75% and 100% of H is located at the position of the 1st, 10th, 18th, 37th, and 46th in the letter sequence. Thus the feature D for H is 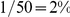, 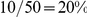, 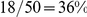, 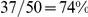, and 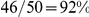. The feature D for N and P can be calculated with the similar method, and the results are: the feature D for N is 4%, 28%, 54%, 78%, and 98%; and that for P is 6%, 24%, 44%, 64%, and 100%, respectively. With all these, the three properties of the letter sequence are 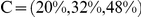, 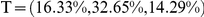, and 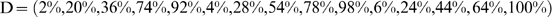, a total of 21 features.

For the solvent accessibility, there are only two local feature groups, and hence resulting in seven features rather than 21 as illustrated above. The amino acid compositions have 20 features, each of which represents the percentage or occurrence frequency of the constituent amino acids in a protein sample [45]. For each of the other five local feature properties, 21 global features can be obtained as in the case of hydrophobicity described above. Using all these results, a total of 132 (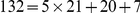) features can be obtained to represent a protein sequence. Listed in **Table 2** are the 132 features used in our study.

**Table 2:** Biochemical and physicochemical features of proteins and their dimensionality.

Property name	Number of feature			Total
	C	T	D	
Hydrophobicity	3	3	15	21
Secondary structure	3	3	15	21
Solvent accessibility	1	1	5	7
Normalized van der Waals Volume	3	3	15	21
Polarity	3	3	15	21
Polarizability	3	3	15	21
Amino Acid Composition	20			20

After each protein was coded, two criteria were applied to the vectors set. (1) Excluded proteins with the same coding vectors, but the different functional categories. (2) Keep one of the proteins that share common coding vectors and functional categories.

#### Feature sorting

Maximum Relevance, Minimum Redundancy (mRMR) Method was originally developed by Peng *et al*. to process microarray data [46]. The idea is to rank each feature based on its relevance to the target and redundancy with other features. A “good” feature is defined as one that has the best trade-off between maximum relevance to target and minimum redundancy within the features. To quantify both relevance and redundancy, mutual information (MI), which estimates how much one vector is related to another, is defined as following.
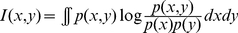
(7)


where 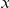, 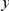 are two vectors, 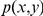 is the joint probabilistic density, 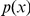 and 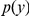 are the marginal probabilistic densities.

Let 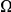 denotes the whole feature set, while 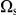 denotes the already-selected feature set which contains 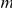 vectors. The to-be-selected feature set with 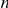 features is denoted by 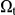. The relevance 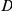 of the feature 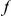 in 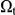 with the target 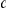 can be calculated by:
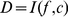
(8)


And redundancy 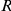 of the feature *f* in 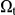 with all the features in 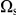 can be calculated by:
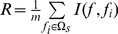
(9)


To obtain the feature 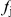 in 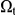 with maximum relevance and minimum redundancy, Eq. (8) and Eq. (9) are combined to obtain the mRMR function:
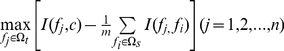
(10)


For a feature set with 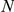, the feature evaluation will be executed 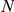 rounds. In the first round, the redundancy is 0 for 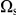 is null, therefore the feature with the maximum relevance to target 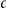 is selected. After the 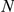 evaluations, the following feature set 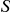 in the selection order can be obtained by the mRMR method:
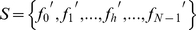
(11)where the subscript index indicates at which round the feature is selected. The better the feature, the earlier it will satisfy Eq. (10), the earlier it will be selected, and the smaller its index will be.

#### Prediction with hybrid properties

Nearest Neighbor Algorithm (NNA) is a simple machine learning method that arranges the object to the class of its nearest neighbor sample. It has been widely used for predicting protein subcellular localization (see, e.g., a comprehensive review [47] and the references cited therein). In this study, the similarity between vector 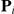 and 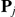 is defined as
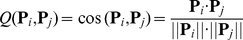
(12)where 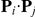 is the inner product of 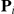 and 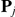, and 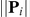 and 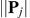 represent their modules, respectively.

Also suppose that a query proteins 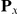 in the dataset consisting of 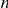 proteins 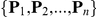, then the larger the value of 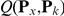, the more likely 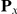 has the same function as 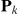.

#### Modeling

Incremental Feature Selection Method [48], [49] (IFS) is employed to select the optimal feature subset from the feature space and the predictor with best performance. Firstly, 132 feature subsets were generated according to the sorted features like this
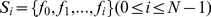
(13)where 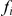 is the 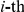 feature in the sorted 132 features. With each feature subset, the proteins were recoded to 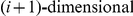
vectors. Then the functions of protein are predicted with the hybrid properties as described above (see Eq.12 and Eq.6) according to NNA. A curve named IFS curve, was plotted by using index *i* as the x-axis and the first order accuracy of feature subset 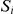 as the y-axis. The optimal feature set 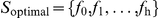 was selected when the curve arrived at the apogee with index 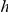. Meanwhile, the predictor based on 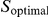 was used to predict the functions of proteins.

For more discussions about the hybrid-property approach, refer to [49], [50].

### Overall prediction

The prediction was carried out according to such a procedure that if a test protein has PPI information, the network-based method was applied for identifying its functions; otherwise, the hybrid-property based method was applied.

Three cross-validation methods are often used in statistical prediction [51]: independent dataset test, subsampling (K-fold) test, and jackknife test. Of these three, the jackknife is deem the most objective that can always yield a unique outcome for a given benchmark dataset as elucidated in [29] and demonstrated by Eq.50 of [47]. Accordingly, the jackknife test has been increasingly used by investigators to evaluate various predictors (see, e.g., [52], [53], [54], [55]). During the jackknifing for the network-based method, each node (protein) was in turn taken away from the PPI network and then predicted. During the jackknifing for the hybrid-property based method, each protein was in turn singled out and predicted according to the NNA. In this study, the *j-th* order overall accuracy 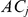 for the dataset can be calculated like this
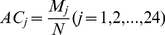
(14)where 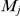 is the number of proteins whose *j-th* order predicted function is the one of the true functions of the proteins, and 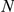 is the total number of proteins in the dataset. Therefore, the 24-order overall accuracies were used as an evaluation for the both methods. The higher 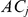 with a small 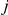 and the lower 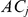 with a large 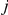 mean that the method performs well in the prediction.

Besides, the average number of functions that each protein in the dataset performs can be calculated like this
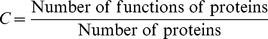
(15)


Hence, another evaluation for both the methods was presented as the likelihood that the first *k-order* prediction results include all the functions of proteins, which can be calculated like this
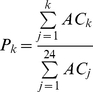
(16)where 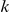 is the smallest integer greater than or equal to 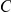. A large 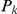 also means a good performance of the method for the protein functions prediction.

## Results and Discussion

### Performance of network-based method

In this study, 9,093 proteins and 1,074 proteins were used to train and test the network-based method. The overall jackknife success rate on 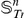 and 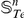
thus obtained for the first-order function was 75.9% and 78.2%, respectively. Shown in **Table 3** are the accuracies of all the 24-order function predictions using the PPI network-based approach. For training set 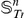, in all the cases, higher-order function prediction is better than the lower one, implying that the protein functions are nicely sorted using the prediction criteria. The average number of functions that a protein possesses is 3.25 according to Eq. (15). Therefore we only consider the first 4 (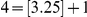) ones in the 24-order predictions. The probability that all true functions included for a protein by taking the first 4-order predicted functions is 68.4% according to Eq. (16), indicating that the predictor performs quite well in predicting these functions.

**Table 3:** The 24-order prediction accuracies of the three methods on the training/test sets.

	Network-based method		Hybrid-property based method				Motif-based method		Overall prediction	
Order	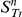	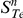	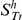	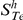	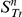	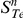	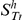	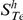	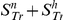	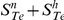
1	75.93%	78.21%	47.15%	35.37%	57.12%	42.67%	31.83%	32.69%	69.07%	70.23%
2	64.38%	70.11%	40.71%	32.52%	51.79%	40.04%	30.48%	27.88%	58.74%	63.11%
3	50.52%	53.91%	35.26%	24.80%	45.65%	39.57%	28.48%	30.77%	46.89%	48.48%
4	31.09%	35.10%	26.14%	21.54%	34.68%	32.33%	24.22%	29.81%	29.91%	32.58%
5	20.07%	24.21%	20.16%	24.39%	25.64%	27.07%	22.82%	18.27%	20.09%	24.24%
6	14.71%	17.60%	14.07%	16.67%	17.95%	20.96%	16.87%	15.87%	14.56%	17.42%
7	11.33%	12.76%	11.86%	13.41%	14.42%	18.42%	14.46%	12.98%	11.45%	12.88%
8	8.37%	9.68%	10.70%	15.85%	10.88%	14.19%	14.41%	12.98%	8.92%	10.83%
9	6.82%	9.87%	8.97%	14.63%	9.11%	13.16%	12.11%	12.98%	7.33%	10.76%
10	6.16%	6.61%	8.27%	13.01%	8.18%	11.75%	13.21%	8.65%	6.66%	7.80%
11	4.76%	5.49%	7.00%	6.50%	6.69%	12.31%	11.41%	11.06%	5.30%	5.68%
12	4.65%	5.87%	6.33%	5.28%	5.95%	10.15%	9.61%	9.62%	5.05%	5.76%
13	3.86%	4.56%	5.77%	5.28%	5.30%	9.40%	8.96%	8.65%	4.32%	4.70%
14	3.66%	3.54%	6.30%	3.66%	5.42%	9.02%	7.11%	8.17%	4.29%	3.56%
15	3.04%	4.10%	4.33%	3.25%	4.43%	8.74%	8.56%	7.21%	3.34%	3.94%
16	2.64%	3.35%	4.22%	2.85%	3.67%	8.74%	6.91%	1.92%	3.02%	3.26%
17	2.36%	2.51%	3.52%	1.22%	3.77%	8.83%	5.21%	2.40%	2.64%	2.27%
18	2.13%	1.86%	4.26%	2.44%	3.19%	6.86%	5.46%	1.92%	2.64%	1.97%
19	1.67%	2.23%	3.87%	3.66%	2.84%	6.11%	5.61%	1.92%	2.20%	2.50%
20	1.63%	2.05%	2.78%	2.03%	2.34%	4.32%	4.50%	0.96%	1.90%	2.05%
21	1.59%	1.49%	2.74%	4.47%	2.07%	4.51%	3.55%	0.48%	1.87%	2.05%
22	1.46%	1.30%	1.83%	0.41%	1.64%	4.51%	4.50%	0.48%	1.55%	1.14%
23	1.07%	1.12%	1.90%	1.22%	1.10%	3.57%	3.20%	0.48%	1.27%	1.14%
24	0.78%	1.12%	0.49%	0.41%	0.06%	0.66%	2.75%	0.48%	0.71%	0.98%

### Performance of hybrid-property based method

**Figure 1:**
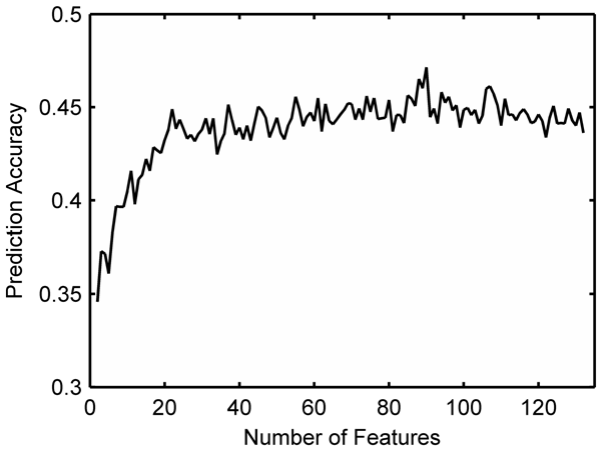
The IFS curve of 132 hybrid features used in hybrid-property based method. It shows that the first order prediction accuracy by the hybrid-property based method varies with the increment of the features. The curve arises to the apogee when the number of features is 90.

**Figure 2:**
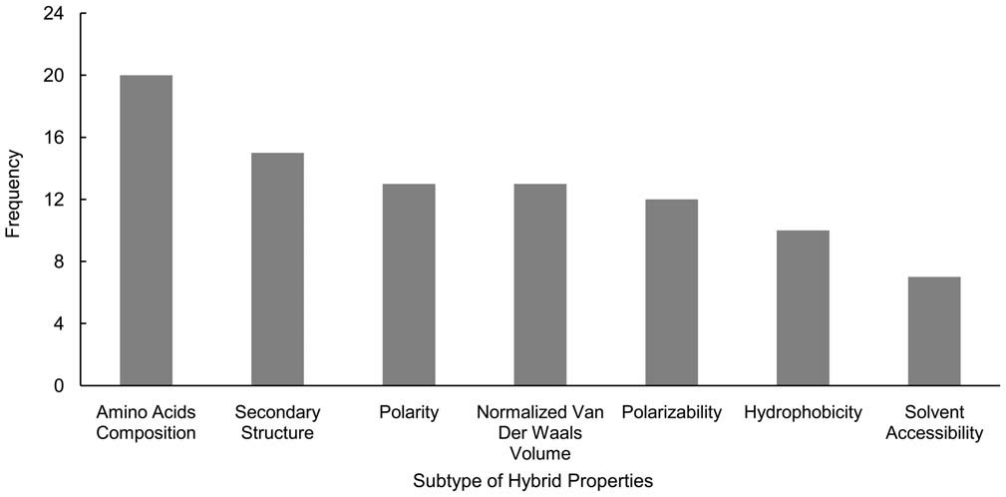
Distribution of the subtype of hybrid properties in the optimized 90 features. X-coordinates represent seven kinds of biochemical and physicochemical attributes, and Y-coordinates correspond with the frequency of each attribute occurring in the selected the 90 features.

After the filtering procedure (see biochemical and physicochemical description of proteins section), the obtained 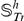 comprised of 2,842 proteins and 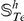 comprised of 246 proteins were then used to train and test the hybrid-property based method. Listed in **Table 3** are the accuracies by the jackknife test with the hybrid-property based method. The prediction accuracy of the first-order predicted function for 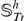 and 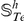 were 47.2% and 35.4%, respectively, using 90 optimized hybrid features selected by IFS procedure from a total of 132 features, which can be seen from the IFS curve in **Figure 1**. Detail of these 90 features can be found in **Table S5**, and the distribution of the subtypes of protein hybrid properties in the 90 features is showed in **Figure 2**. For the training set 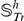, the average number of functions that a protein possesses is 2.81. Thus the first 3 (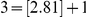) ones in the 24-order predictions is considered. According to Eq. (16), it is 44.1% for the probability that all true functions of a protein are included by taking the first 3-order predicted functions, indicating that the predictor using hybrid properties performs fairly well.

### The overall prediction

The overall prediction combines the results of predictions from both network-based and hybrid-property based methods. The accuracies of the first-order function prediction for the overall training set (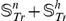) and test set (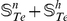) were 69.1% and 70.2%, respectively. Results of the other-order function predictions are shown in **Table 3**. For the overall training set, the average number of functions that a protein possesses is 3.14. Here, we still only consider the first 4 (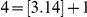) ones in the 24-order predictions. According to Eq. (16), it is 65.2% for the probability that all true functions of a protein are included by taking the first 4-order predicted functions, indicating that our method performs quite well for the entire prediction. In this study, for the 24-order predicted functions generated by the method, the first 4-order predicted functions should be paid more attention to than other functions.

### Comparison of network-based methods with STRING data and IntAct data

Besides the known PPIs, STRING also includes the predicted PPIs from the computational prediction methods. Here we want to investigate whether using both the known and predicted PPIs will improve the performance of the predictor using only the known PPIs or not. The known PPIs were downloaded from the IntAct [56], where the high-quality molecular interactions were collected from the curation of published literature or from the data depositions of the expert curators. After mapping the IntAct data to MfunGD, we filtered the proteins without function annotation. As a result, a PPI network consisted of 1,939 proteins and 6,543 known interactions was obtained. With the network, the jackknife test prediction accuracies of the network-based method for the 1,939 proteins are listed in **Table 4**. Meanwhile, with the STRING network (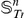), the prediction accuracies for the 1,939 proteins are also listed in **Table 4**. The first-order prediction accuracy with STRING data is 83.5%, 26% higher than the first-order prediction accuracy with IntAct data (57.5%). For the collected 1939 proteins, the average number of functions that a protein possesses is 3.94. The probabilities that all true functions of a protein are included by taking the first 4-order predicted functions are 66.3% and 47.4% for STRING data and IntAct data, respectively. The comparison indicates that the network-based method performed better with the STRING PPIs than the IntAct PPIs.

**Table 4:** The jackknife test prediction accuracies of network-based method with STRING data and IntAct data on the selected 1939 proteins.

Order	STRING data	IntAct data
1	83.50%	57.50%
2	75.45%	53.12%
3	62.09%	41.98%
4	40.23%	33.94%
5	25.84%	27.64%
6	18.05%	19.65%
7	13.67%	16.25%
8	12.27%	12.94%
9	9.95%	11.04%
10	7.79%	10.06%
11	6.34%	9.80%
12	6.29%	9.33%
13	5.83%	8.46%
14	5.16%	7.68%
15	3.92%	7.68%
16	3.82%	8.05%
17	2.94%	8.56%
18	3.09%	7.01%
19	1.70%	8.20%
20	1.81%	8.51%
21	1.39%	6.29%
22	1.08%	6.19%
23	0.83%	6.14%
24	0.88%	7.89%

Limited to the existing biotechnology means, many PPIs are difficult to detect in the lab. Therefore, the existing PPI networks derived from the experiments only cover a very small part of the total proteome. For example, InterAct [56] stores ∼3000 mouse protein with PPI information about 7% of the mouse proteom; and DIP [57] contains ∼1,000 mouse protein with PPI information, about 2% of the mouse proteom. STRING trys to integrate as many PPIs data as possible mainly from four sources: genomic context, high-throughput experiments, co-expression and previous knowledge. STRING (version 8.0) gathered ∼2.5 million proteins of 630 organisms together. For mouse, STRING covers ∼25% of the proteom. Therefore, the functions of more proteins will be predicted using the network-based method with STIRNG PPIs.

It should be pointed out that STRING data contains many predicted PPIs, which may lead to the wrong classifications. To avid the problem, we used the interaction confidence score (i.e. edge weight) in the network-based method as described above. According to STRING, a more reliable PPI will be assigned a higher edge weight. From the principle (Eq. (1) – Eq. (6)), the network-based method is robust with respect to false PPIs. Overall, the predicted PPIs should be used very cautiously.

### Comparison between the network-based method and hybrid-property based method

In this study, network-based method and hybrid-property based method were developed to predict the functions of protein in mouse. In order to compare the performance between them, we also trained and tested the hybrid-property based method using the 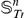 and 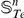. The prediction results are listed in the **Table 3**. The first-order prediction accuracies on the 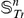 and 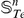 are 57.1% and 42.7%, respectively, which are much lower than the prediction accuracies of the network-based method on the same training set and test. For the training set 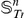, the probability that all true functions included for a protein by taking the first 4-order predicted functions is 58.4% according to Eq. (16), which are also lower than the probability of 68.4% of the network-based method. Therefore, the network-based method outperforms the hybrid-property based method.

### Comparison between the hybrid-property based method and the motif-based method

As a sequence-based method, the hybrid-property based method should be compared to other sequence-based method. We selected the method based on the motif information to predict functions of proteins, which has been proved to effective for the predicting functions of proteins in yeast [58]. The motif-based method can be described as follows: First, 739 short domain sequence were downloaded from SBASE [59], which is a collection of domain sequences designed for facilitating the detection of domain homologies. Then BLASTP [7] was used to compare the protein with the 739 domain sequences to find the alignments with e-value lower than 0.8. The protein sequences can be represented by vector: 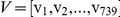, where 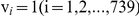 when e-value of the alignment lower than 0.8, otherwise 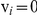. Using NNA, the method was trained and tested on the same training set (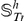) and test set (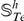). The prediction results are listed in the **Table 3**. The first-order prediction accuracies on the 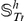 and 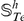 are 31.8% and 32.7%, respectively, which are lower than the prediction accuracies of the hybrid-property based method. For 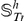, the probability that all true functions included for a protein by taking the first 3-order predicted functions is 30.6%, which are also lower than the probability of 44.1% of the hybrid-property based method. Overall, the hybrid-property based method performs a little better than the motif-based method.

### Biological relevance of the optimized hybrid features

It is shown in **Figure 2** that amino acid compositions and secondary structure contribute the most towards protein function prediction. These protein properties have also been used for predicting many other protein attributes, such as classification of nuclear receptors, protein fold recognition [60], protein quaternary structure [61], membrane protein types [28], and protein folding rate [62], [63], among many others. Amino acid compositions are reported to correlate to proteins' structural and biological characters [64], [65]. Alteration of secondary structure is a common and causative factor for causing human diseases [66], [67], [68] by probably altering the protein functions. It has also been reported that the alteration of secondary structure of amyloid beta peptide relates to the neurotoxic activity in vitro [69], [70]. Listed above are just a few examples of showing the importance of these protein properties in shaping protein functions. There are surely a number of other findings in validating their importance, as well as the importance of other properties investigated in this study, such as the polarity, normalized van der Waals volume, polarizability, hydrophobicity, and solvent accessibility.

In this study, we propose a novel multi-target model, in which a sample may belong to several classes, for predicting protein functions. Two kinds of multi-target predictors are implemented: one is for proteins with PPI information and the other for those without PPI information. The average number of functions that a protein possesses is 3.14. There are 24 protein functional categories, meaning that in average a random guess of a protein function will have a success chance of 13.1% (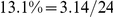), much lower than the first order prediction accuracy of 69.1%. Therefore, our method can serve as a useful high throughput tool for annotating the functions for many uncharacterized protein sequences. It is very interesting to see that the PPI network-based method is significantly better than the hybrid-property based method in both the rates of first-order function prediction and the probability rates calculated by Eq. (16). It is anticipated that the method based on the PPI network information is quite promising, and may become a powerful tool for annotating the functions of proteins.

## Supporting Information

Table S1 Training set for network-based method. The Mfun ID and Functional number (see Table 1) of proteins are shown.
(5.22 MB DOC)

Table S2 Test set for network-based method. The Mfun ID and Functional number (see Table 1) of proteins are shown.
(0.63 MB DOC)

Table S3 Training set for hybrid-property based method. The Mfun ID and Functional number (see Table 1) of proteins are shown.
(1.65 MB DOC)

Table S4 Test set for hybrid-property based method. The Mfun ID and Functional number (see Table 1) of proteins are shown.
(0.16 MB DOC)

Table S5 The 90 optimized hybrid features generated by IFS procedure from a total of 132 features.
(0.11 MB DOC)
