# Supplementary material for: Predicting Functions of Proteins in Mouse Based on Weighted Protein-Protein Interaction Network and Protein Hybrid Properties
Source: PLoS One. 2011 Jan 19;6(1):e14556. doi: 10.1371/journal.pone.0014556 (PMC3023709; doi:10.1371/journal.pone.0014556)
Supplement: Table S1 — Training set for network-based method. The Mfun ID and Functional number (see Table 1) of proteins are shown. (5.22 MB DOC) [file pone.0014556.s001.doc]

Table S1. Training set for network-based method. The Mfun ID and Functional number (see Table 1) of proteins are shown.

| MfunGD ID | Functional number(s) | MfunGD ID | Functional number(s) |
| --- | --- | --- | --- |
| mc10000023 | 1 7 21 | mc2001084 | 4 7 21 |
| mc10000024 | 6 7 8 10 12 13 17 | mc2001093 | 4 7 21 |
| mc10000030 | 4 7 10 13 20 21 | mc2001098 | 1 7 24 |
| mc10000036 | 10 21 | mc2001100 | 7 21 |
| mc10000038 | 3 7 16 21 | mc2001101 | 1 3 6 7 10 11 21 |
| mc10000039 | 5 21 | mc2001106 | 1 21 |
| mc10000046 | 8 10 13 | mc2001114 | 1 3 6 7 17 21 |
| mc10000052 | 1 | mc2001115 | 6 |
| mc10000056 | 1 6 | mc2001117 | 4 7 15 16 21 |
| mc10000057 | 9 17 21 | mc2001118 | 4 7 15 16 21 |
| mc10000060 | 1 3 6 7 17 21 | mc2001121 | 7 10 12 21 |
| mc10000063 | 6 | mc2001122 | 1 6 8 21 |
| mc10000066 | 6 7 8 10 | mc2001124 | 1 6 7 8 9 10 21 |
| mc10000071 | 21 | mc2001134 | 3 7 21 |
| mc10000075 | 3 | mc2001147 | 4 7 13 18 19 21 |
| mc1000008 | 10 12 13 | mc2001150 | 7 |
| mc1000010 | 4 7 21 | mc2001155 | 4 6 7 8 17 21 |
| mc10000105 | 6 7 9 10 21 | mc2001159 | 6 7 9 15 17 21 |
| mc10000107 | 7 10 | mc2001161 | 9 10 12 21 |
| mc10000111 | 3 4 7 17 21 | mc2001168 | 4 7 18 21 |
| mc10000114 | 1 2 6 8 10 13 16 21 | mc2001184 | 7 16 21 |
| mc10000123 | 7 10 12 13 16 19 21 | mc2001185 | 4 7 15 16 20 21 |
| mc10000132 | 4 7 | mc2001186 | 4 7 15 16 20 21 |
| mc10000137 | 1 11 | mc2001188 | 4 7 15 16 20 21 |
| mc10000138 | 6 7 17 21 | mc2001189 | 4 7 15 16 21 |
| mc10000151 | 4 7 10 21 | mc2001193 | 4 7 15 16 21 |
| mc10000164 | 10 | mc2001195 | 4 7 15 16 21 |
| mc10000179 | 4 7 8 16 20 21 | mc2001196 | 6 7 9 21 |
| mc10000185 | 7 | mc2001213 | 7 9 21 |
| mc10000194 | 21 | mc2001215 | 4 7 21 |
| mc1000019 | 5 21 | mc2001220 | 1 |
| mc10000198 | 15 21 | mc2001231 | 2 6 7 9 20 21 24 |
| mc10000203 | 4 7 21 | mc2001236 | 1 9 |
| mc10000209 | 4 7 21 | mc2001237 | 1 6 7 11 |
| mc10000214 | 7 10 21 | mc2001239 | 6 7 21 |
| mc10000215 | 7 8 10 21 | mc2001240 | 7 |
| mc10000222 | 1 6 9 12 16 21 | mc2001279 | 6 7 15 21 |
| mc10000224 | 1 6 7 8 10 15 | mc2001290 | 4 7 15 16 18 20 21 |
| mc1000022 | 4 7 21 | mc2001295 | 3 7 10 12 16 18 21 22 |
| mc10000225 | 7 17 21 | mc2001304 | 6 7 11 21 |
| mc10000227 | 4 7 15 21 | mc2001306 | 7 8 10 21 |
| mc10000230 | 1 10 | mc2001309 | 7 10 17 |
| mc10000235 | 3 24 | mc2001311 | 1 6 7 8 10 21 |
| mc10000236 | 3 4 7 9 21 | mc2001337 | 10 15 |
| mc10000242 | 5 7 | mc2001339 | 7 10 12 15 16 20 |
| mc10000243 | 1 7 9 24 | mc2001341 | 6 7 21 |
| mc10000256 | 1 6 7 11 15 21 | mc2001352 | 7 10 21 |
| mc1000025 | 8 10 21 | mc2001353 | 8 13 21 |
| mc10000269 | 1 4 7 9 16 21 | mc2001359 | 2 7 9 21 |
| mc10000271 | 4 7 16 21 | mc2001363 | 4 7 21 |
| mc10000274 | 1 4 6 7 8 10 16 21 | mc2001365 | 8 13 21 |
| mc10000283 | 1 21 | mc2001366 | 6 7 |
| mc10000284 | 1 21 | mc2001368 | 6 7 9 21 |
| mc10000286 | 8 10 | mc2001370 | 10 |
| mc1000029 | 1 2 7 8 9 21 | mc2001373 | 7 13 21 |
| mc10000299 | 7 9 21 | mc2001374 | 7 17 21 |
| mc10000305 | 3 7 10 12 15 20 21 | mc2001375 | 9 10 21 |
| mc10000306 | 1 3 7 16 19 21 | mc2001376 | 4 7 21 |
| mc10000312 | 1 7 21 24 | mc2001378 | 7 10 21 |
| mc1000032 | 10 21 | mc2001386 | 10 13 |
| mc10000323 | 3 6 10 15 17 21 | mc2001396 | 10 12 13 |
| mc10000336 | 4 21 | mc2001492 | 10 13 |
| mc1000034 | 3 4 21 | mc2001529 | 10 13 21 |
| mc10000343 | 7 12 16 17 21 | mc2001544 | 10 13 |
| mc10000348 | 1 6 8 10 21 | mc2001552 | 10 13 21 |
| mc10000361 | 2 7 9 | mc2001561 | 10 13 |
| mc10000371 | 1 | mc2001595 | 10 13 |
| mc10000372 | 7 | mc2001637 | 1 6 8 10 19 20 21 |
| mc10000378 | 4 7 16 19 20 21 | mc2001643 | 6 9 17 21 |
| mc10000408 | 7 21 | mc2001644 | 7 |
| mc10000410 | 10 | mc2001646 | 9 21 |
| mc10000415 | 21 24 | mc2001650 | 1 6 8 10 21 |
| mc10000417 | 3 7 21 | mc2001651 | 4 7 21 |
| mc10000421 | 16 17 19 20 21 | mc2001653 | 6 7 12 21 |
| mc10000424 | 1 3 6 10 15 21 | mc2001654 | 6 7 8 11 21 |
| mc10000436 | 3 4 6 7 21 | mc2001656 | 4 7 18 19 21 |
| mc10000437 | 7 | mc2001657 | 7 12 13 17 19 21 23 |
| mc10000450 | 1 7 12 16 17 20 21 | mc2001659 | 1 15 |
| mc10000464 | 7 13 15 | mc2001661 | 1 21 |
| mc10000468 | 3 7 21 | mc2001662 | 3 7 11 21 |
| mc10000476 | 9 | mc2001664 | 7 9 21 23 24 |
| mc10000479 | 21 | mc2001666 | 7 21 |
| mc10000485 | 1 6 7 | mc2001670 | 7 9 21 |
| mc10000489 | 1 2 7 9 21 | mc2001673 | 6 7 13 21 |
| mc10000495 | 6 7 12 15 17 21 | mc2001675 | 8 10 |
| mc10000497 | 10 13 21 | mc2001681 | 10 12 |
| mc10000500 | 1 10 21 | mc2001682 | 3 7 15 16 21 |
| mc10000504 | 4 7 21 | mc2001683 | 7 10 21 |
| mc10000507 | 1 2 7 9 21 | mc2001686 | 4 7 21 |
| mc10000508 | 1 7 | mc2001690 | 4 7 |
| mc10000510 | 12 21 | mc2001692 | 6 7 17 21 |
| mc1000051 | 7 21 | mc2001694 | 7 8 10 15 21 |
| mc10000526 | 4 7 9 11 15 16 18 19 20 21 | mc2001700 | 1 6 13 21 |
| mc10000531 | 4 7 10 12 16 18 20 21 | mc2001708 | 9 12 21 |
| mc10000533 | 2 7 9 18 19 21 | mc2001718 | 21 |
| mc10000534 | 6 7 9 11 21 | mc2001722 | 4 7 16 21 22 |
| mc10000550 | 21 | mc2001723 | 1 21 |
| mc1000055 | 5 21 | mc2001728 | 1 21 |
| mc10000556 | 7 9 21 | mc2001736 | 4 7 15 21 |
| mc1000056 | 1 7 21 24 | mc2001769 | 3 7 21 |
| mc10000564 | 15 21 | mc2001770 | 3 7 13 18 21 |
| mc10000566 | 4 5 7 16 18 20 21 | mc2001772 | 4 7 8 10 13 15 |
| mc10000581 | 21 | mc2001784 | 7 |
| mc10000600 | 7 9 10 12 21 | mc2001790 | 9 12 21 23 24 |
| mc10000602 | 4 7 16 19 20 21 | mc2001794 | 7 10 12 20 21 |
| mc1000060 | 3 4 7 21 | mc2001796 | 2 6 7 21 |
| mc10000607 | 7 8 10 13 21 | mc2001797 | 15 |
| mc10000610 | 10 21 | mc2001799 | 4 7 21 |
| mc1000061 | 6 21 | mc2001801 | 4 7 16 21 |
| mc10000616 | 4 7 8 16 19 21 | mc2001802 | 7 9 11 21 |
| mc10000618 | 1 3 6 7 21 | mc2001809 | 6 9 21 23 |
| mc10000620 | 7 9 10 21 | mc2001815 | 7 21 |
| mc10000621 | 21 | mc2001818 | 6 |
| mc10000631 | 8 9 | mc2001825 | 1 4 6 7 10 15 21 |
| mc10000637 | 3 7 21 | mc2001828 | 4 7 21 |
| mc10000643 | 1 6 7 11 21 | mc2001832 | 7 |
| mc10000660 | 9 10 13 16 21 | mc2001842 | 4 7 15 21 |
| mc10000672 | 4 6 7 11 16 21 | mc2001844 | 21 |
| mc10000673 | 21 | mc2001846 | 7 21 |
| mc10000674 | 1 7 8 | mc2001854 | 4 7 20 21 24 |
| mc10000677 | 1 11 21 | mc2001858 | 6 9 |
| mc10000683 | 6 7 9 21 | mc2001859 | 6 7 11 |
| mc1000068 | 4 6 7 8 21 | mc2001879 | 10 21 |
| mc10000687 | 7 9 10 15 16 20 21 | mc2001881 | 7 9 12 21 |
| mc10000697 | 1 7 21 | mc2001892 | 1 |
| mc10000707 | 6 7 11 | mc2001893 | 2 7 9 21 |
| mc10000711 | 7 21 | mc2001900 | 7 12 13 15 16 18 20 21 |
| mc10000712 | 1 6 21 | mc2001908 | 1 21 |
| mc10000714 | 1 21 | mc2001909 | 21 |
| mc10000715 | 7 12 13 17 20 21 22 | mc2001952 | 10 13 |
| mc10000723 | 9 | mc2001964 | 4 5 7 21 |
| mc10000724 | 7 10 15 16 | mc2001970 | 21 |
| mc10000729 | 1 4 8 21 | mc2001971 | 9 12 |
| mc10000730 | 1 15 21 | mc2001972 | 7 9 15 |
| mc10000734 | 7 15 21 | mc2001978 | 7 8 9 16 17 21 |
| mc10000737 | 10 12 15 16 18 21 | mc2001981 | 10 15 16 21 |
| mc10000738 | 5 7 10 | mc2001983 | 6 7 9 10 11 21 |
| mc10000742 | 1 7 21 | mc2001987 | 9 10 12 13 21 |
| mc10000743 | 7 9 10 21 | mc2001995 | 9 13 |
| mc1000074 | 6 7 | mc2002017 | 4 7 20 21 |
| mc10000748 | 12 21 | mc2002035 | 7 8 10 15 16 |
| mc10000753 | 4 7 15 16 19 21 | mc2002038 | 7 8 10 |
| mc10000759 | 10 21 | mc2002056 | 7 |
| mc10000761 | 1 2 7 21 | mc2002061 | 1 5 6 7 10 |
| mc10000763 | 4 7 21 | mc2002062 | 6 7 9 10 21 |
| mc10000764 | 9 21 | mc2002063 | 7 15 21 |
| mc10000765 | 21 | mc2002065 | 1 3 6 7 21 |
| mc10000767 | 7 | mc2002068 | 1 7 10 21 |
| mc10000768 | 3 4 7 21 | mc2002075 | 1 21 |
| mc10000770 | 3 4 7 21 | mc2002078 | 1 21 |
| mc10000774 | 15 | mc2002083 | 1 |
| mc10000776 | 9 21 | mc2002085 | 3 7 |
| mc10000777 | 7 9 21 | mc2002087 | 1 |
| mc10000780 | 1 | mc2002088 | 4 6 7 11 21 |
| mc10000782 | 12 13 | mc2002091 | 6 8 15 20 21 |
| mc10000786 | 3 4 6 7 15 16 19 20 21 | mc2002092 | 7 10 |
| mc10000787 | 6 7 11 | mc2002093 | 6 7 9 17 21 |
| mc10000801 | 1 7 12 21 | mc2002102 | 7 |
| mc10000822 | 4 7 21 | mc2002104 | 3 7 17 21 |
| mc10000827 | 21 | mc2002105 | 6 7 21 |
| mc10000832 | 4 7 21 | mc2002108 | 1 7 10 |
| mc10000850 | 1 3 6 7 10 21 | mc2002113 | 1 6 7 10 12 21 |
| mc10000876 | 6 17 21 | mc2002118 | 4 7 21 |
| mc10000893 | 5 7 18 | mc2002123 | 7 |
| mc10000898 | 4 7 21 | mc2002124 | 1 |
| mc10000903 | 21 | mc2002131 | 2 7 9 10 21 |
| mc10000918 | 21 | mc2002134 | 7 9 17 21 |
| mc10000928 | 7 12 13 19 21 | mc2002139 | 1 6 7 |
| mc10000938 | 7 10 21 | mc2002140 | 5 6 7 21 |
| mc10000948 | 10 12 13 21 | mc2002142 | 7 21 |
| mc10000951 | 1 7 | mc2002143 | 6 7 9 15 18 21 |
| mc10000953 | 4 7 21 | mc2002144 | 1 6 7 |
| mc10000956 | 1 16 21 | mc2002149 | 17 21 |
| mc10000957 | 1 13 21 | mc2002150 | 3 4 7 11 21 |
| mc10000961 | 1 | mc2002153 | 1 |
| mc10000962 | 11 24 | mc2002156 | 13 21 |
| mc10000965 | 11 21 24 | mc2002157 | 9 12 16 21 22 23 24 |
| mc10000966 | 11 13 15 21 | mc2002158 | 6 7 9 21 |
| mc10000968 | 3 4 7 17 21 | mc2002162 | 7 |
| mc10000969 | 6 7 21 | mc2002177 | 5 |
| mc10000970 | 21 | mc2002183 | 7 10 11 13 21 |
| mc10000972 | 21 | mc2002184 | 7 |
| mc10000977 | 7 9 | mc2002187 | 5 7 21 |
| mc10000978 | 1 4 7 21 | mc2002188 | 1 7 |
| mc10000979 | 7 13 16 21 | mc2002195 | 7 9 21 |
| mc10000982 | 3 21 | mc2002199 | 1 21 |
| mc10000985 | 7 21 | mc2002201 | 2 7 9 |
| mc10000986 | 1 | mc2002203 | 7 9 18 |
| mc10000988 | 1 7 21 | mc2002205 | 1 9 21 |
| mc10000989 | 7 9 12 17 21 | mc2002230 | 7 9 21 |
| mc1000099 | 1 15 21 | mc2002232 | 1 7 21 |
| mc10000992 | 19 21 | mc2002243 | 4 6 7 8 10 21 |
| mc10000996 | 7 9 21 | mc2002244 | 1 7 21 |
| mc10000997 | 7 9 12 15 17 18 20 21 | mc2002247 | 7 10 15 19 21 |
| mc10001002 | 1 6 | mc2002252 | 1 7 9 21 |
| mc10001004 | 1 4 7 24 | mc2002253 | 1 7 |
| mc10001009 | 7 10 12 13 16 | mc2002255 | 4 7 21 |
| mc10001010 | 16 21 | mc2002257 | 3 6 7 10 |
| mc10001011 | 6 | mc2002258 | 6 |
| mc10001012 | 6 7 21 | mc2002259 | 1 6 7 9 |
| mc10001018 | 21 | mc2002262 | 1 2 7 9 |
| mc10001023 | 7 | mc2002263 | 3 17 21 |
| mc10001027 | 1 7 9 10 | mc2002267 | 3 4 7 |
| mc10001031 | 4 7 13 21 | mc2002268 | 4 7 15 21 |
| mc10001032 | 3 7 16 21 | mc2002272 | 1 6 8 10 21 |
| mc10001033 | 7 11 13 21 | mc2002274 | 6 7 |
| mc10001035 | 21 | mc2002275 | 7 10 20 21 |
| mc10001036 | 10 21 | mc2002279 | 21 |
| mc10001044 | 4 5 7 21 | mc2002282 | 4 7 21 |
| mc10001046 | 8 | mc2002283 | 5 21 |
| mc10001047 | 1 7 | mc2002289 | 1 3 6 7 21 |
| mc10001055 | 6 15 20 21 24 | mc2002304 | 3 6 7 21 |
| mc10001057 | 9 12 21 23 24 | mc2002305 | 1 3 6 7 10 21 |
| mc10001063 | 10 13 21 | mc2002307 | 21 |
| mc10001064 | 10 | mc2002309 | 7 21 |
| mc1000106 | 4 7 10 13 21 | mc2002313 | 6 7 |
| mc10001066 | 1 21 | mc2002314 | 1 4 7 21 |
| mc10001073 | 7 12 18 20 21 | mc2002318 | 9 10 21 |
| mc10001074 | 13 16 17 | mc2002321 | 3 7 10 13 15 21 |
| mc10001076 | 6 15 21 | mc2002324 | 3 7 10 12 13 15 21 |
| mc10001077 | 7 21 | mc2002328 | 6 7 10 12 17 21 |
| mc10001078 | 7 9 12 13 21 24 | mc2002329 | 9 10 12 |
| mc10001079 | 4 7 21 | mc2002331 | 1 6 7 |
| mc10001080 | 15 21 | mc2002334 | 1 6 7 19 |
| mc10001081 | 6 7 21 | mc2002335 | 1 6 |
| mc10001085 | 15 17 21 | mc2002337 | 10 13 21 24 |
| mc10001090 | 6 21 | mc2002339 | 5 21 |
| mc10001093 | 6 13 | mc2002340 | 1 2 7 9 21 |
| mc10001094 | 4 | mc2002344 | 6 7 9 12 21 |
| mc10001096 | 7 10 12 15 21 | mc2002349 | 7 9 21 |
| mc10001097 | 4 7 21 | mc2002350 | 1 6 8 10 21 |
| mc10001099 | 7 9 10 12 21 | mc2002351 | 5 21 |
| mc10001102 | 7 | mc2002353 | 10 13 20 21 |
| mc10001103 | 2 7 9 10 | mc2002354 | 1 6 7 21 |
| mc10001106 | 7 21 | mc2002357 | 1 |
| mc10001108 | 1 6 7 10 | mc2002364 | 7 10 13 15 16 |
| mc10001110 | 2 7 9 21 | mc2002365 | 10 16 |
| mc10001112 | 7 21 | mc2002366 | 6 7 10 12 21 |
| mc10001114 | 7 21 | mc2002367 | 7 12 21 |
| mc10001117 | 2 7 9 21 | mc2002368 | 7 11 |
| mc10001118 | 1 15 16 | mc2002371 | 4 7 21 |
| mc10001119 | 7 15 16 21 | mc2002372 | 1 3 6 7 8 21 |
| mc10001120 | 5 7 21 | mc2002374 | 12 13 21 |
| mc10001121 | 3 7 17 20 24 | mc2002375 | 1 13 16 21 24 |
| mc10001123 | 6 | mc2002378 | 1 2 9 21 |
| mc10001124 | 10 | mc2002379 | 7 10 13 21 |
| mc10001131 | 2 7 9 21 | mc2002382 | 1 7 11 12 21 |
| mc10001132 | 4 7 | mc2002384 | 10 |
| mc10001135 | 4 7 21 | mc2002386 | 1 7 9 |
| mc10001137 | 7 9 16 17 21 | mc2002390 | 3 6 7 8 21 |
| mc10001140 | 4 7 10 21 | mc2002400 | 21 |
| mc10001141 | 1 | mc2002401 | 1 |
| mc1000114 | 6 9 21 | mc2002403 | 3 7 21 |
| mc1000115 | 1 21 | mc2002404 | 1 |
| mc10001152 | 6 9 17 21 | mc2002425 | 1 2 21 24 |
| mc10001153 | 1 6 21 | mc2002435 | 1 7 10 |
| mc10001156 | 10 16 19 20 21 23 24 | mc2002444 | 1 10 |
| mc10001157 | 13 21 | mc2002446 | 15 21 |
| mc10001158 | 1 8 | mc2002459 | 7 16 |
| mc10001161 | 4 7 21 | mc2002462 | 7 10 15 16 18 19 21 |
| mc10001162 | 6 7 | mc2002483 | 1 |
| mc10001163 | 6 7 9 21 | mc2002486 | 1 6 |
| mc10001164 | 7 21 | mc2002522 | 4 7 21 |
| mc10001165 | 3 7 8 10 15 16 21 | mc2002523 | 20 21 |
| mc10001168 | 7 10 | mc2002527 | 6 21 |
| mc10001169 | 9 21 | mc2002531 | 4 7 |
| mc10001170 | 6 7 11 | mc2002537 | 7 11 |
| mc10001172 | 7 21 | mc2002542 | 4 7 21 |
| mc10001174 | 6 7 10 21 | mc2002551 | 1 4 7 21 |
| mc10001176 | 4 7 21 | mc2002554 | 21 |
| mc10001177 | 4 6 7 8 10 21 | mc2002555 | 7 9 17 21 |
| mc10001178 | 5 7 | mc2002557 | 1 5 21 |
| mc10001180 | 1 6 7 10 15 21 | mc2002558 | 9 |
| mc10001182 | 9 | mc2002564 | 9 12 21 |
| mc10001184 | 1 6 7 10 | mc2002574 | 8 9 10 |
| mc10001186 | 7 8 12 | mc2002576 | 1 6 |
| mc10001187 | 7 17 | mc2002577 | 4 7 21 |
| mc10001188 | 1 10 16 | mc2002580 | 7 21 |
| mc10001192 | 3 4 5 7 17 21 | mc2002592 | 1 3 4 7 21 |
| mc10001195 | 3 6 | mc2002594 | 4 7 16 21 |
| mc10001207 | 10 21 | mc2002597 | 4 16 20 21 |
| mc10001208 | 6 7 10 21 | mc2002606 | 4 7 15 16 20 21 |
| mc10001209 | 7 8 10 20 21 | mc2002618 | 4 7 19 20 21 |
| mc10001213 | 4 7 10 21 | mc2002622 | 10 |
| mc10001215 | 1 3 4 6 7 21 | mc2002623 | 7 10 13 16 21 |
| mc10001230 | 4 7 21 | mc2002624 | 7 10 11 12 21 |
| mc10001233 | 1 6 21 | mc2002629 | 4 7 20 21 |
| mc10001243 | 3 8 21 | mc2002636 | 8 |
| mc10001254 | 1 4 7 21 | mc2002638 | 8 21 |
| mc10001257 | 4 7 21 | mc2002640 | 8 21 |
| mc10001264 | 12 21 | mc2002649 | 8 21 |
| mc10001273 | 21 | mc2002667 | 8 21 |
| mc10001279 | 6 7 21 | mc2002668 | 1 |
| mc10001280 | 7 9 12 16 21 22 | mc2002669 | 1 2 21 |
| mc10001283 | 8 21 | mc2002670 | 4 7 12 13 16 21 |
| mc10001288 | 6 7 11 21 | mc2002679 | 1 |
| mc10001295 | 7 9 10 12 | mc2002680 | 7 21 |
| mc10001306 | 4 7 16 19 20 21 | mc2002683 | 7 8 12 22 |
| mc10001307 | 1 7 | mc2002689 | 2 6 9 |
| mc10001317 | 6 9 17 21 | mc2002691 | 7 10 15 20 21 24 |
| mc10001321 | 7 | mc2002698 | 7 11 |
| mc10001324 | 3 7 21 | mc2002700 | 4 7 16 21 |
| mc1000132 | 4 7 21 | mc2002703 | 6 7 8 10 |
| mc10001328 | 4 7 18 21 22 | mc2002705 | 6 7 21 |
| mc10001332 | 7 9 10 21 | mc2002707 | 1 4 6 7 8 15 21 |
| mc10001334 | 9 | mc2002719 | 8 9 11 12 13 21 |
| mc10001338 | 1 4 7 10 21 | mc2002723 | 7 10 |
| mc10001342 | 9 | mc2002724 | 6 21 |
| mc10001344 | 7 17 21 | mc2002727 | 4 7 8 21 |
| mc10001359 | 7 8 11 15 16 20 21 | mc2002730 | 2 7 9 21 24 |
| mc10001363 | 9 21 | mc2002731 | 7 8 10 15 21 |
| mc10001365 | 4 7 10 21 | mc2002733 | 3 7 21 |
| mc10001394 | 1 6 7 | mc2002736 | 4 7 21 |
| mc10001395 | 1 21 | mc2002737 | 1 6 8 10 |
| mc10001400 | 7 16 21 | mc2002743 | 1 6 7 8 10 |
| mc10001405 | 6 7 | mc2002750 | 4 7 15 21 |
| mc10001411 | 4 6 7 10 21 | mc2002753 | 6 |
| mc10001412 | 2 9 21 | mc2002762 | 7 9 |
| mc10001422 | 8 10 16 21 | mc2002767 | 3 7 15 21 |
| mc10001425 | 7 10 15 | mc2002771 | 6 7 9 21 |
| mc10001427 | 10 15 18 | mc2002773 | 7 16 17 21 22 |
| mc10001431 | 6 7 | mc2002774 | 7 |
| mc10001432 | 1 7 | mc2002776 | 7 21 |
| mc1000144 | 3 4 7 11 17 21 | mc2002780 | 7 |
| mc10001453 | 4 6 7 8 12 15 16 19 20 21 | mc2002782 | 7 |
| mc10001466 | 21 | mc2002787 | 7 21 |
| mc10001468 | 21 | mc2002788 | 6 7 12 13 17 21 23 24 |
| mc10001501 | 1 2 21 | mc2002790 | 4 7 21 |
| mc1000150 | 4 5 7 21 | mc2002791 | 1 6 7 21 |
| mc10001504 | 6 7 12 15 18 21 | mc2002793 | 21 |
| mc1000151 | 1 13 21 | mc2002799 | 7 21 |
| mc10001520 | 10 20 21 | mc2002800 | 5 7 |
| mc10001521 | 1 7 10 | mc2002803 | 7 10 21 |
| mc10001526 | 4 7 16 21 | mc2002809 | 2 7 17 21 |
| mc10001530 | 9 21 | mc2002813 | 21 |
| mc1000153 | 7 9 21 | mc2002814 | 4 7 8 10 21 |
| mc10001558 | 4 7 15 16 19 21 | mc2002816 | 1 |
| mc10001559 | 4 7 15 16 19 21 | mc2002817 | 1 21 |
| mc10001591 | 3 4 7 15 21 | mc2002818 | 1 7 |
| mc10001597 | 1 6 7 21 | mc2002820 | 10 |
| mc10001601 | 1 9 | mc2002822 | 1 6 7 |
| mc10001607 | 3 21 | mc2002824 | 6 7 21 |
| mc1000161 | 11 13 21 | mc2002825 | 5 10 12 21 |
| mc10001618 | 7 21 | mc2002827 | 7 |
| mc10001619 | 21 | mc2002828 | 7 10 15 16 19 20 21 |
| mc10001621 | 21 | mc2002833 | 21 |
| mc10001635 | 1 6 7 | mc2002835 | 1 7 9 21 |
| mc10001638 | 1 7 | mc2002836 | 7 21 |
| mc10001639 | 21 | mc2002838 | 1 7 21 |
| mc10001640 | 7 9 10 | mc2002839 | 21 |
| mc1000164 | 7 19 21 23 | mc2002840 | 4 7 8 21 |
| mc10001650 | 21 | mc2002853 | 7 |
| mc10001654 | 1 6 8 10 21 | mc2002861 | 4 7 21 |
| mc10001659 | 1 6 8 10 21 | mc2002865 | 21 |
| mc10001660 | 9 21 | mc2002869 | 1 10 13 |
| mc1000166 | 15 16 20 21 24 | mc2002870 | 3 4 7 21 |
| mc10001663 | 4 7 21 | mc2002872 | 1 6 7 21 |
| mc10001675 | 21 | mc2002873 | 10 21 |
| mc10001676 | 6 7 11 21 | mc2002876 | 6 7 10 |
| mc10001678 | 7 10 21 | mc2002886 | 7 15 21 |
| mc10001681 | 3 4 7 15 21 | mc2002891 | 6 7 21 |
| mc10001682 | 1 6 9 21 | mc2002894 | 7 11 21 |
| mc10001692 | 3 6 7 8 15 21 | mc2002895 | 7 11 21 |
| mc10001696 | 6 9 17 21 | mc2002904 | 9 21 |
| mc10001698 | 3 7 9 10 | mc2002905 | 7 17 21 |
| mc10001703 | 21 | mc2002906 | 4 7 |
| mc10001708 | 4 5 7 9 10 11 12 13 15 21 | mc2002908 | 3 4 7 |
| mc10001734 | 6 7 12 | mc2002935 | 4 7 13 16 21 |
| mc10001735 | 3 4 7 | mc2002939 | 3 7 21 |
| mc1000173 | 8 21 | mc2002940 | 1 7 10 16 |
| mc10001738 | 6 7 10 15 21 | mc2002942 | 4 7 21 |
| mc10001742 | 3 4 7 17 21 | mc2002943 | 19 23 |
| mc10001750 | 7 11 21 | mc2002945 | 21 |
| mc1000175 | 21 | mc2002957 | 1 6 8 10 21 |
| mc10001756 | 1 6 7 10 16 21 | mc2002967 | 4 7 21 |
| mc10001763 | 1 | mc2002973 | 6 10 16 20 |
| mc10001765 | 10 | mc2002974 | 3 4 7 21 |
| mc10001768 | 1 6 7 10 | mc2002979 | 1 7 13 16 21 23 |
| mc10001781 | 21 | mc2002985 | 4 7 8 10 20 21 24 |
| mc10001786 | 10 | mc2002988 | 15 21 |
| mc10001794 | 6 | mc2002990 | 1 7 8 |
| mc10001814 | 9 21 | mc2002991 | 1 7 21 |
| mc10001847 | 7 11 17 21 | mc2002993 | 1 7 12 15 21 |
| mc10001848 | 5 21 | mc2002995 | 9 21 |
| mc10001850 | 1 4 9 21 | mc2002999 | 6 7 9 21 |
| mc1000185 | 4 7 21 | mc2003000 | 1 6 7 10 11 15 21 |
| mc10001858 | 1 21 | mc2003001 | 7 9 12 21 |
| mc10001860 | 8 | mc2003002 | 6 |
| mc10001862 | 1 10 | mc2003003 | 8 13 21 |
| mc10001864 | 3 7 18 21 | mc2003004 | 13 21 |
| mc1000186 | 4 7 21 | mc2003005 | 21 |
| mc10001868 | 4 7 11 18 21 | mc2003009 | 21 |
| mc10001872 | 8 | mc2003010 | 21 |
| mc10001873 | 4 7 10 16 21 | mc2003011 | 21 |
| mc10001874 | 10 15 21 | mc2003013 | 7 17 21 |
| mc10001875 | 10 15 21 | mc2003014 | 4 7 21 24 |
| mc10001880 | 1 7 21 | mc2003015 | 7 17 21 |
| mc10001881 | 7 21 | mc2003016 | 9 21 |
| mc10001882 | 1 7 9 10 21 | mc2003020 | 6 7 21 |
| mc10001883 | 4 7 10 21 | mc2003022 | 8 21 |
| mc10001884 | 4 21 | mc2003024 | 8 21 |
| mc10001887 | 10 12 21 | mc2003029 | 21 |
| mc10001894 | 21 | mc2003031 | 7 8 21 |
| mc10001897 | 1 | mc2003032 | 3 6 7 15 |
| mc1000190 | 20 | mc2003035 | 1 2 21 |
| mc10001902 | 3 4 7 21 | mc2003040 | 6 7 21 |
| mc10001903 | 4 7 8 9 21 | mc2003041 | 9 21 |
| mc10001906 | 2 7 9 21 | mc2003042 | 21 |
| mc10001907 | 3 4 7 17 21 | mc2003045 | 6 7 21 |
| mc10001909 | 7 21 | mc2003048 | 4 21 |
| mc10001912 | 1 6 7 9 12 13 20 21 24 | mc2003053 | 5 7 10 13 15 18 21 |
| mc10001913 | 4 7 12 16 20 21 | mc2003063 | 7 9 |
| mc10001915 | 1 9 21 | mc2003065 | 9 21 |
| mc10001916 | 4 7 10 21 | mc2003068 | 1 4 6 7 8 10 15 16 21 |
| mc10001917 | 21 | mc2003071 | 1 |
| mc10001919 | 21 | mc2003073 | 4 7 8 10 21 |
| mc10001928 | 3 4 7 17 21 | mc2003086 | 1 6 7 21 |
| mc10001930 | 2 7 9 19 21 | mc2003088 | 3 6 7 9 15 21 |
| mc1000193 | 13 15 21 | mc2003089 | 7 9 21 |
| mc10001934 | 4 6 7 21 | mc2003092 | 3 4 7 21 |
| mc10001938 | 5 7 21 | mc2003098 | 9 12 13 21 |
| mc10001940 | 7 21 | mc2003100 | 1 2 7 9 21 |
| mc1000194 | 10 13 15 18 21 22 | mc2003102 | 1 7 21 |
| mc10001941 | 1 7 21 | mc2003106 | 18 21 24 |
| mc10001946 | 1 21 | mc2003107 | 6 7 15 16 21 |
| mc10001947 | 1 7 8 10 21 | mc2003109 | 7 16 20 21 |
| mc10001950 | 6 7 15 16 20 21 | mc2003111 | 6 7 21 |
| mc10001952 | 6 7 10 11 | mc2003115 | 4 7 10 13 21 |
| mc10001956 | 21 | mc2003120 | 1 6 8 10 21 |
| mc10001957 | 1 13 16 21 | mc2003138 | 4 7 13 15 21 |
| mc10001958 | 7 9 17 18 | mc2003142 | 7 21 |
| mc10001959 | 7 10 12 15 20 21 | mc2003145 | 4 7 16 21 |
| mc10001960 | 1 21 | mc2003164 | 7 21 |
| mc1000196 | 3 7 21 | mc2003166 | 1 7 9 11 21 |
| mc10001965 | 10 13 | mc2003169 | 7 10 16 |
| mc10001995 | 10 13 | mc2003197 | 10 |
| mc1000200 | 6 9 | mc2003200 | 1 3 6 7 10 |
| mc10002026 | 4 7 10 12 15 16 18 20 21 | mc2003201 | 4 7 21 |
| mc10002040 | 1 | mc2003211 | 4 7 15 21 |
| mc10002041 | 15 21 | mc2003216 | 7 10 12 15 16 18 20 21 |
| mc10002043 | 7 16 21 | mc2003217 | 1 3 7 20 21 |
| mc10002046 | 7 10 15 16 18 21 | mc2003218 | 3 7 9 21 |
| mc10002047 | 3 4 7 20 21 | mc2003219 | 7 |
| mc10002052 | 1 3 4 6 7 21 | mc2003221 | 1 2 7 8 |
| mc1000224 | 1 7 21 | mc2003223 | 4 7 |
| mc1000227 | 18 | mc2003224 | 7 |
| mc1000233 | 17 21 | mc2003237 | 7 9 10 |
| mc1000234 | 7 16 17 19 21 | mc2003238 | 7 9 21 |
| mc1000270 | 1 3 6 8 10 16 21 | mc2003246 | 6 9 21 |
| mc1000276 | 1 | mc2003247 | 6 7 21 |
| mc1000296 | 3 4 7 21 | mc2003257 | 4 21 |
| mc1000301 | 7 9 10 16 | mc2003258 | 6 21 |
| mc1000302 | 6 7 11 15 | mc2003259 | 2 7 9 21 |
| mc1000303 | 4 7 21 | mc2003264 | 11 13 18 20 21 |
| mc1000310 | 7 16 17 19 21 | mc2003292 | 7 12 17 20 21 23 24 |
| mc1000316 | 4 5 7 21 | mc2003311 | 6 7 8 11 13 21 |
| mc1000318 | 1 6 7 8 10 21 | mc2003315 | 10 |
| mc1000320 | 1 2 6 21 | mc2003318 | 1 9 |
| mc1000321 | 15 21 | mc2003319 | 7 12 |
| mc1000322 | 6 21 | mc2003322 | 5 7 21 |
| mc1000344 | 6 21 | mc2003323 | 7 8 15 |
| mc1000355 | 1 21 | mc2003326 | 4 7 21 |
| mc1000361 | 2 7 9 21 | mc2003329 | 7 10 13 |
| mc1000362 | 7 9 21 | mc2003333 | 10 15 21 |
| mc1000366 | 1 6 7 10 13 18 21 | mc2003334 | 7 12 17 21 |
| mc1000375 | 1 10 | mc2003335 | 7 21 |
| mc1000383 | 7 15 21 | mc2003340 | 4 15 20 |
| mc1000387 | 5 7 21 | mc2003345 | 7 8 9 21 |
| mc1000391 | 2 9 | mc2003348 | 9 10 12 21 22 |
| mc1000392 | 5 7 21 | mc2003352 | 5 7 8 15 21 |
| mc1000393 | 1 3 7 11 21 | mc2003355 | 1 6 7 10 21 |
| mc1000408 | 1 6 13 21 | mc2003356 | 1 6 7 10 |
| mc1000410 | 12 15 | mc2003359 | 4 7 21 |
| mc1000415 | 4 7 10 20 21 24 | mc2003363 | 7 9 10 16 21 |
| mc1000416 | 5 21 | mc2003372 | 1 7 |
| mc1000417 | 8 | mc2003376 | 4 7 21 |
| mc1000423 | 5 7 21 | mc2003378 | 4 7 21 |
| mc1000426 | 1 6 7 10 | mc2003380 | 7 10 |
| mc1000428 | 10 15 21 | mc2003382 | 10 |
| mc1000430 | 8 10 13 21 | mc2003385 | 1 6 |
| mc1000431 | 8 10 13 21 | mc2003386 | 1 4 7 21 |
| mc1000433 | 1 3 7 10 11 21 | mc2003393 | 1 7 |
| mc1000435 | 10 21 | mc2003397 | 7 12 15 16 20 21 |
| mc1000436 | 10 21 | mc2003398 | 7 15 16 21 |
| mc1000437 | 9 21 | mc2003402 | 1 3 6 7 10 17 21 |
| mc1000441 | 9 | mc2003404 | 7 15 16 21 |
| mc1000471 | 4 7 21 | mc2003414 | 9 21 |
| mc1000476 | 5 21 | mc2003419 | 13 21 |
| mc1000483 | 7 15 | mc2003421 | 10 13 |
| mc1000496 | 7 10 12 | mc2003424 | 7 9 21 22 |
| mc1000499 | 21 | mc2003426 | 1 2 6 7 10 21 |
| mc1000504 | 6 | mc2003427 | 7 9 10 |
| mc1000508 | 21 | mc2003432 | 2 7 9 21 |
| mc1000510 | 1 3 7 11 21 | mc2003434 | 15 |
| mc1000517 | 7 9 10 13 | mc2003439 | 1 6 7 |
| mc1000521 | 7 9 12 17 21 | mc2003440 | 7 21 |
| mc1000522 | 7 12 17 21 | mc2003441 | 7 |
| mc1000531 | 7 9 | mc2003442 | 16 |
| mc1000544 | 9 | mc2003447 | 7 13 15 21 |
| mc1000567 | 7 17 21 | mc2003449 | 7 9 12 |
| mc1000576 | 2 7 9 17 21 | mc2003457 | 1 7 |
| mc1000578 | 3 4 7 10 15 21 | mc3000012 | 4 7 21 |
| mc1000579 | 3 4 7 10 15 21 | mc3000014 | 6 7 12 16 17 21 |
| mc1000585 | 4 7 8 18 21 | mc3000024 | 1 7 8 |
| mc1000592 | 1 7 10 | mc3000027 | 7 10 13 15 18 21 |
| mc1000593 | 1 21 | mc3000028 | 1 2 7 |
| mc1000596 | 7 10 15 21 | mc3000039 | 4 7 20 |
| mc1000597 | 3 11 | mc3000041 | 5 21 |
| mc1000599 | 6 7 | mc3000047 | 7 9 10 15 21 |
| mc1000606 | 1 6 7 10 15 21 | mc3000063 | 1 7 9 |
| mc1000617 | 4 7 21 | mc3000068 | 7 9 |
| mc1000619 | 6 7 11 15 21 | mc3000071 | 7 10 21 |
| mc1000620 | 6 7 11 21 | mc3000074 | 9 |
| mc1000622 | 7 9 10 21 | mc3000076 | 10 |
| mc1000626 | 5 7 | mc3000098 | 7 |
| mc1000629 | 3 5 7 15 16 21 | mc3000104 | 9 21 |
| mc1000649 | 4 7 16 21 | mc3000107 | 3 4 7 15 21 |
| mc1000670 | 1 2 7 9 | mc3000110 | 1 2 7 |
| mc1000673 | 5 | mc3000113 | 1 2 7 |
| mc1000674 | 1 6 7 10 21 | mc3000128 | 4 21 |
| mc1000676 | 6 | mc3000129 | 1 2 7 9 21 |
| mc1000677 | 21 | mc3000137 | 21 |
| mc1000678 | 3 4 7 21 | mc3000138 | 1 10 21 |
| mc1000680 | 2 7 9 21 | mc3000142 | 2 6 7 9 11 |
| mc1000686 | 6 7 8 10 15 | mc3000146 | 13 |
| mc1000689 | 6 7 8 13 15 21 | mc3000150 | 1 7 9 21 |
| mc1000691 | 6 7 9 21 | mc3000151 | 6 7 9 17 18 |
| mc1000696 | 8 10 | mc3000153 | 1 |
| mc1000702 | 3 6 7 9 21 | mc3000154 | 6 7 21 |
| mc1000703 | 4 7 17 21 | mc3000188 | 7 12 21 |
| mc1000704 | 6 7 10 21 | mc3000199 | 21 |
| mc1000708 | 4 7 21 22 24 | mc3000203 | 3 8 10 |
| mc1000714 | 7 8 15 17 21 | mc3000204 | 1 |
| mc1000720 | 13 15 18 21 | mc3000206 | 7 13 15 21 |
| mc1000723 | 13 21 22 | mc3000207 | 10 |
| mc1000724 | 7 11 21 | mc3000208 | 18 19 |
| mc1000742 | 10 12 15 16 19 20 21 | mc3000213 | 1 10 21 |
| mc1000747 | 2 7 9 21 | mc3000219 | 9 13 21 24 |
| mc1000748 | 5 7 8 21 | mc3000223 | 5 7 21 |
| mc1000749 | 10 | mc3000234 | 4 7 21 |
| mc1000757 | 7 10 16 20 21 24 | mc3000240 | 4 7 21 |
| mc1000760 | 1 7 | mc3000255 | 9 |
| mc1000767 | 4 7 21 | mc3000262 | 15 21 |
| mc1000774 | 4 7 15 16 21 | mc3000263 | 7 17 21 22 23 24 |
| mc1000779 | 10 15 16 18 20 21 | mc3000266 | 9 |
| mc1000792 | 20 21 24 | mc3000278 | 9 |
| mc1000799 | 1 7 10 | mc3000280 | 15 21 |
| mc1000800 | 10 | mc3000284 | 6 7 10 |
| mc1000808 | 7 16 17 21 22 | mc3000288 | 6 7 8 10 21 |
| mc1000812 | 1 2 | mc3000290 | 3 4 7 17 21 |
| mc1000815 | 1 2 7 21 | mc3000292 | 2 7 9 21 |
| mc1000822 | 7 8 10 21 | mc3000294 | 6 7 9 17 21 |
| mc1000840 | 4 7 21 | mc3000310 | 7 |
| mc1000844 | 7 16 21 | mc3000324 | 4 7 13 15 21 |
| mc1000853 | 4 6 7 21 | mc3000345 | 16 19 20 |
| mc1000859 | 1 | mc3000346 | 1 7 21 |
| mc1000860 | 1 7 12 13 15 17 21 | mc3000348 | 1 2 7 21 |
| mc1000873 | 1 7 9 21 | mc3000354 | 7 13 |
| mc1000875 | 3 7 21 | mc3000357 | 1 3 4 7 21 |
| mc1000878 | 3 4 7 21 | mc3000358 | 3 7 8 21 |
| mc1000880 | 5 21 | mc3000359 | 12 13 |
| mc1000882 | 7 15 21 | mc3000370 | 1 4 6 7 |
| mc1000883 | 7 15 21 | mc3000371 | 4 7 8 9 10 13 15 21 |
| mc1000887 | 7 15 16 17 21 | mc3000374 | 7 10 15 21 |
| mc1000909 | 7 10 21 | mc3000377 | 3 7 10 15 16 18 20 21 |
| mc1000910 | 10 12 21 | mc3000378 | 4 5 15 16 |
| mc1000912 | 10 | mc3000379 | 7 |
| mc1000920 | 7 17 21 | mc3000382 | 8 10 16 20 |
| mc1000923 | 10 15 | mc3000385 | 5 7 21 |
| mc1000924 | 1 7 10 16 | mc3000430 | 7 9 21 |
| mc1000926 | 2 6 7 21 | mc3000432 | 6 7 11 |
| mc1000927 | 6 7 21 | mc3000434 | 1 6 7 10 |
| mc1000928 | 1 6 7 10 | mc3000464 | 7 12 21 |
| mc1000932 | 1 7 9 21 | mc3000485 | 4 7 21 |
| mc1000935 | 7 10 15 16 21 | mc3000489 | 7 12 21 |
| mc1000936 | 7 10 15 16 21 | mc3000498 | 9 |
| mc1000940 | 7 20 21 24 | mc3000508 | 12 |
| mc1000943 | 6 7 10 15 16 18 19 20 21 | mc3000509 | 4 7 21 |
| mc1000949 | 7 9 12 21 | mc3000511 | 2 7 9 21 |
| mc1000952 | 4 7 | mc3000512 | 4 7 21 |
| mc1000954 | 1 6 7 10 | mc3000514 | 7 9 10 21 |
| mc1000958 | 1 6 8 10 21 | mc3000515 | 1 3 21 |
| mc1000959 | 21 | mc3000518 | 1 8 10 11 21 |
| mc1000960 | 6 7 21 | mc3000528 | 4 7 10 20 21 |
| mc1000961 | 21 | mc3000543 | 6 9 21 |
| mc1000962 | 1 6 21 | mc3000544 | 7 21 |
| mc1000964 | 1 | mc3000549 | 21 |
| mc1000965 | 9 10 21 | mc3000551 | 7 10 21 |
| mc1000969 | 10 15 21 | mc3000552 | 7 12 16 19 21 |
| mc1000970 | 1 | mc3000559 | 6 21 |
| mc1000971 | 9 21 | mc3000562 | 7 12 21 |
| mc1001009 | 1 21 | mc3000571 | 1 3 4 7 21 |
| mc1001016 | 7 9 | mc3000572 | 1 6 21 |
| mc1001025 | 7 21 | mc3000574 | 4 7 20 |
| mc1001033 | 7 8 15 16 21 | mc3000575 | 4 7 21 |
| mc1001042 | 3 6 21 | mc3000580 | 3 7 8 16 21 24 |
| mc1001066 | 7 10 21 | mc3000582 | 21 |
| mc1001072 | 7 12 21 | mc3000584 | 7 |
| mc1001075 | 21 | mc3000585 | 1 6 7 10 15 16 |
| mc1001079 | 7 8 9 16 21 | mc3000594 | 15 20 |
| mc1001087 | 9 21 | mc3000605 | 10 15 21 |
| mc1001098 | 1 | mc3000607 | 10 15 21 |
| mc1001107 | 10 | mc3000608 | 4 7 8 21 |
| mc1001109 | 7 8 10 21 | mc3000611 | 6 7 21 |
| mc1001110 | 6 | mc3000626 | 6 17 21 |
| mc1001116 | 7 | mc3000633 | 3 6 7 15 16 21 |
| mc1001117 | 21 24 | mc3000645 | 10 |
| mc1001125 | 10 19 20 | mc3000647 | 10 |
| mc1001131 | 4 5 7 9 21 | mc3000648 | 10 13 21 |
| mc1001133 | 10 21 | mc3000662 | 1 9 21 |
| mc1001137 | 21 | mc3000663 | 10 |
| mc1001138 | 1 12 13 | mc3000668 | 4 7 21 |
| mc1001139 | 6 7 8 10 21 | mc3000674 | 10 15 21 |
| mc1001141 | 1 7 10 20 21 | mc3000688 | 3 4 7 |
| mc1001143 | 1 7 | mc3000695 | 6 7 |
| mc1001149 | 1 24 | mc3000698 | 1 10 |
| mc1001150 | 6 7 21 | mc3000728 | 9 21 |
| mc1001154 | 9 10 12 21 | mc3000735 | 6 |
| mc1001155 | 9 10 12 21 | mc3000742 | 3 4 7 8 21 |
| mc1001156 | 5 7 21 | mc3000746 | 1 7 8 9 12 13 21 |
| mc1001163 | 10 15 22 | mc3000758 | 4 7 16 21 |
| mc1001168 | 1 7 10 13 15 18 21 | mc3000759 | 4 7 21 |
| mc1001169 | 7 9 17 21 | mc3000762 | 21 |
| mc1001170 | 7 10 12 13 | mc3000764 | 5 7 21 |
| mc1001177 | 1 21 | mc3000766 | 8 |
| mc1001185 | 9 11 12 13 | mc3000796 | 7 21 |
| mc1001186 | 16 19 21 | mc3000797 | 7 10 13 15 21 |
| mc1001206 | 4 7 16 24 | mc3000804 | 3 7 17 21 |
| mc1001210 | 10 21 | mc3000808 | 1 6 7 10 |
| mc1001216 | 6 7 8 21 | mc3000819 | 1 6 7 21 |
| mc1001220 | 7 8 9 18 21 | mc3000821 | 5 7 21 |
| mc1001222 | 7 9 10 | mc3000841 | 16 |
| mc1001230 | 6 7 | mc3000843 | 1 |
| mc1001234 | 1 | mc3000848 | 8 |
| mc1001238 | 1 7 10 12 | mc3000851 | 15 |
| mc1001239 | 4 7 8 15 16 21 | mc3000852 | 8 21 |
| mc1001240 | 4 10 12 21 | mc3000855 | 21 |
| mc1001248 | 4 6 7 8 21 | mc3000886 | 6 7 11 21 |
| mc1001252 | 2 7 9 21 | mc3000887 | 2 7 9 21 |
| mc1001266 | 10 16 21 | mc3000889 | 10 17 20 |
| mc1001270 | 6 15 21 | mc3000901 | 1 3 9 10 12 21 |
| mc1001271 | 10 | mc3000904 | 6 7 8 10 15 21 |
| mc1001272 | 9 | mc3000908 | 6 |
| mc1001273 | 2 7 9 17 21 | mc3000909 | 1 7 |
| mc1001274 | 1 2 7 21 24 | mc3000911 | 1 10 13 21 |
| mc1001280 | 1 6 7 10 | mc3000912 | 1 10 |
| mc1001282 | 21 | mc3000917 | 10 |
| mc1001284 | 1 7 9 21 | mc3000921 | 1 12 13 21 |
| mc1001285 | 3 7 | mc3000925 | 7 12 13 21 |
| mc1001286 | 7 8 10 12 16 21 | mc3000926 | 21 |
| mc1001288 | 15 | mc3000927 | 13 21 |
| mc1001291 | 6 9 21 | mc3000928 | 4 7 21 |
| mc1001293 | 1 7 | mc3000938 | 7 10 21 |
| mc1001294 | 4 7 | mc3000940 | 10 13 15 21 |
| mc1001298 | 1 11 | mc3000943 | 3 6 7 8 11 15 17 21 |
| mc1001299 | 15 21 | mc3000944 | 6 7 21 |
| mc1001308 | 1 6 21 | mc3000959 | 4 6 7 10 21 |
| mc1001311 | 9 | mc3000968 | 1 4 5 6 21 |
| mc1001322 | 3 7 | mc3000973 | 6 |
| mc1001323 | 1 5 7 21 | mc3000976 | 5 21 |
| mc1001356 | 7 12 21 | mc3000983 | 1 6 7 10 |
| mc1001362 | 1 21 | mc3000985 | 13 15 18 21 |
| mc1001363 | 21 | mc3000993 | 13 21 |
| mc1001365 | 10 13 18 21 | mc3000996 | 10 21 |
| mc1001381 | 7 8 10 15 21 | mc3000997 | 6 7 10 15 21 |
| mc1001384 | 1 7 9 21 | mc3001001 | 4 6 7 21 |
| mc1001386 | 8 | mc3001008 | 1 6 7 9 10 16 21 |
| mc1001388 | 8 | mc3001009 | 3 10 |
| mc1001389 | 8 | mc3001010 | 3 |
| mc1001393 | 8 | mc3001011 | 7 15 21 |
| mc1001399 | 8 | mc3001012 | 5 21 |
| mc1001401 | 6 8 | mc3001015 | 1 7 9 16 21 |
| mc1001402 | 6 8 21 | mc3001017 | 16 21 |
| mc1001405 | 8 | mc3001018 | 7 12 18 |
| mc1001458 | 3 7 21 | mc3001021 | 7 9 21 |
| mc1001459 | 7 21 | mc3001024 | 4 7 21 |
| mc1001476 | 7 9 10 | mc3001027 | 9 21 |
| mc1001480 | 21 | mc3001028 | 6 7 11 21 |
| mc1001481 | 1 6 7 8 10 21 | mc3001034 | 7 19 20 21 23 24 |
| mc1001487 | 9 10 21 | mc3001041 | 1 4 7 21 |
| mc1001488 | 7 10 21 | mc3001042 | 9 21 |
| mc1001490 | 6 9 | mc3001044 | 10 15 16 |
| mc1001494 | 6 7 9 10 21 | mc3001047 | 7 17 21 |
| mc1001497 | 4 7 16 19 20 21 23 24 | mc3001050 | 4 7 9 10 |
| mc1001510 | 10 | mc3001051 | 7 21 |
| mc1001511 | 3 7 | mc3001052 | 6 21 |
| mc1001522 | 6 | mc3001054 | 6 7 9 21 |
| mc1001532 | 6 7 12 17 21 | mc3001056 | 7 8 10 |
| mc1001539 | 10 | mc3001058 | 10 |
| mc1001553 | 1 6 21 | mc3001060 | 7 10 21 |
| mc1001561 | 8 | mc3001061 | 7 9 21 |
| mc1001567 | 21 | mc3001068 | 5 15 21 |
| mc1001569 | 3 4 7 21 | mc3001069 | 4 7 21 |
| mc1001571 | 4 5 7 21 | mc3001070 | 21 |
| mc1001573 | 10 11 12 15 16 18 20 | mc3001074 | 1 2 7 12 |
| mc1001591 | 13 | mc3001076 | 1 6 7 10 21 |
| mc1001593 | 7 16 21 | mc3001077 | 9 21 |
| mc1001594 | 13 21 | mc3001079 | 1 11 17 21 |
| mc1001595 | 1 2 7 | mc3001080 | 7 9 16 21 |
| mc1001596 | 6 | mc3001081 | 7 12 21 |
| mc1001599 | 7 10 13 | mc3001082 | 7 21 |
| mc1001600 | 10 | mc3001084 | 21 |
| mc1001604 | 6 10 13 15 21 | mc3001086 | 21 |
| mc1001608 | 5 13 15 21 | mc3001087 | 10 16 18 21 |
| mc1001609 | 1 4 6 7 10 21 | mc3001090 | 21 |
| mc1001611 | 1 6 7 10 | mc3001091 | 6 7 10 12 16 21 |
| mc1001612 | 5 7 | mc3001094 | 4 7 15 16 21 |
| mc1001613 | 7 10 | mc3001099 | 6 7 10 21 |
| mc1001614 | 1 6 7 8 10 | mc3001101 | 4 7 21 |
| mc1001618 | 10 | mc3001102 | 1 7 21 |
| mc1001620 | 6 7 14 21 | mc3001105 | 7 9 |
| mc1001621 | 7 9 21 | mc3001107 | 1 4 7 21 |
| mc1001622 | 9 | mc3001108 | 9 10 12 13 21 |
| mc1001625 | 7 9 10 | mc3001110 | 6 7 |
| mc1001626 | 1 21 | mc3001113 | 10 21 |
| mc1001629 | 9 | mc3001118 | 7 10 11 21 |
| mc1001631 | 3 4 7 21 | mc3001122 | 7 13 19 21 |
| mc1001639 | 1 6 7 | mc3001126 | 3 5 7 10 21 |
| mc1001643 | 1 6 7 10 | mc3001128 | 14 21 |
| mc1001645 | 21 | mc3001129 | 7 21 |
| mc1001651 | 12 18 20 21 22 23 24 | mc3001133 | 4 7 21 |
| mc1001660 | 6 7 21 | mc3001138 | 1 6 7 10 |
| mc1001663 | 1 5 6 7 10 11 | mc3001140 | 4 7 21 |
| mc1001667 | 21 | mc3001141 | 7 9 |
| mc1001670 | 6 21 | mc3001143 | 7 21 |
| mc1001672 | 1 2 9 | mc3001144 | 7 10 21 |
| mc1001673 | 4 7 16 21 24 | mc3001145 | 7 |
| mc1001679 | 7 | mc3001147 | 2 7 9 |
| mc1001688 | 21 | mc3001148 | 7 |
| mc1001689 | 17 20 21 | mc3001149 | 7 |
| mc1001692 | 21 | mc3001150 | 3 7 15 |
| mc1001698 | 1 2 21 | mc3001151 | 7 |
| mc1001699 | 7 8 13 23 24 | mc3001152 | 7 10 12 13 |
| mc1001701 | 4 7 16 19 21 | mc3001153 | 7 10 12 13 |
| mc1001706 | 1 7 9 | mc3001165 | 7 17 19 21 |
| mc1001710 | 7 8 9 10 | mc3001172 | 7 19 |
| mc1001715 | 7 15 21 | mc3001177 | 7 19 |
| mc1001718 | 7 9 12 19 21 22 24 | mc3001180 | 7 15 17 19 21 |
| mc1001726 | 1 6 8 10 21 | mc3001181 | 7 19 |
| mc1001728 | 7 10 | mc3001183 | 7 19 |
| mc1001731 | 4 7 15 16 17 21 | mc3001218 | 7 21 |
| mc1001732 | 6 7 21 | mc3001221 | 7 15 21 |
| mc1001737 | 1 7 9 12 16 17 21 | mc3001222 | 7 |
| mc1001742 | 7 13 17 19 21 | mc3001224 | 7 |
| mc1001744 | 21 | mc3001229 | 8 10 |
| mc1001745 | 13 17 19 21 23 | mc3001231 | 4 6 7 10 15 20 21 |
| mc1001746 | 7 12 21 | mc3001232 | 6 7 21 |
| mc1001766 | 7 21 | mc3001234 | 1 7 8 21 |
| mc1001772 | 1 4 7 10 21 | mc3001238 | 7 |
| mc1001785 | 6 7 10 21 22 | mc3001241 | 20 |
| mc1001786 | 2 9 | mc3001244 | 7 |
| mc1001801 | 7 12 13 17 20 | mc3001248 | 6 21 |
| mc1001805 | 3 21 | mc3001250 | 4 7 21 |
| mc1001806 | 13 21 | mc3001251 | 1 7 10 21 |
| mc1001814 | 13 21 | mc3001252 | 7 21 |
| mc1001828 | 1 6 7 21 | mc3001253 | 6 7 8 11 21 |
| mc1001833 | 7 | mc3001255 | 4 7 21 24 |
| mc1001834 | 6 7 21 | mc3001256 | 7 17 21 |
| mc1001835 | 3 8 10 21 | mc3001260 | 6 9 |
| mc1001836 | 8 10 21 | mc3001261 | 10 15 16 21 |
| mc1001838 | 8 10 13 21 | mc3001263 | 7 |
| mc1001841 | 8 10 21 | mc3001265 | 10 15 |
| mc1001858 | 1 7 13 21 | mc3001267 | 15 |
| mc1001861 | 1 7 12 13 20 21 | mc3001268 | 1 2 21 |
| mc1001864 | 2 8 9 10 12 13 21 | mc3001271 | 1 4 7 21 |
| mc1001866 | 21 | mc3001272 | 1 3 7 16 21 |
| mc1001882 | 4 7 11 21 | mc3001276 | 4 7 9 10 12 21 |
| mc1001885 | 4 7 21 | mc3001277 | 6 21 |
| mc1001900 | 4 21 | mc3001279 | 6 21 |
| mc1001904 | 8 10 | mc3001283 | 7 8 9 |
| mc1001911 | 1 7 8 11 13 21 22 | mc3001284 | 7 15 16 21 |
| mc1001916 | 1 | mc3001287 | 7 9 21 |
| mc1001917 | 7 12 17 21 | mc3001288 | 5 |
| mc1001918 | 7 12 17 21 | mc3001291 | 4 7 21 |
| mc1001925 | 3 4 7 21 | mc3001292 | 5 21 |
| mc1001926 | 1 | mc3001295 | 6 7 8 10 21 |
| mc1001930 | 8 10 21 | mc3001296 | 1 2 7 21 |
| mc1001931 | 1 3 4 6 7 | mc3001297 | 8 21 |
| mc1001938 | 1 21 | mc3001301 | 7 21 |
| mc1001944 | 7 9 10 12 13 21 24 | mc3001302 | 7 9 21 |
| mc1001955 | 3 21 | mc3001314 | 7 21 |
| mc1001957 | 7 9 21 | mc3001318 | 7 8 9 10 11 13 15 21 |
| mc1001961 | 10 | mc3001324 | 21 |
| mc1001966 | 7 | mc3001329 | 7 21 |
| mc1001970 | 1 2 7 9 21 | mc3001330 | 17 21 |
| mc1001979 | 7 13 21 | mc3001335 | 4 6 7 21 |
| mc1001988 | 1 | mc3001339 | 6 7 21 |
| mc1001989 | 21 | mc3001340 | 1 10 21 |
| mc1001990 | 8 10 | mc3001341 | 6 7 10 21 |
| mc1001991 | 20 21 24 | mc3001343 | 9 10 20 21 |
| mc1002020 | 7 12 13 | mc3001344 | 9 10 16 20 21 |
| mc1002031 | 6 7 21 | mc3001346 | 1 21 |
| mc1002039 | 7 12 17 21 | mc3001353 | 3 4 7 11 |
| mc1002045 | 7 10 12 16 21 | mc3001354 | 7 9 11 24 |
| mc1002047 | 5 21 | mc3001361 | 7 9 21 |
| mc1002048 | 6 7 21 24 | mc3001363 | 4 7 10 15 16 19 21 |
| mc1002059 | 7 8 13 21 | mc3001364 | 6 7 10 21 24 |
| mc1002061 | 5 7 | mc3001367 | 7 21 |
| mc1002067 | 1 11 13 | mc3001368 | 1 21 |
| mc1002072 | 7 13 15 21 | mc3001372 | 1 21 24 |
| mc1002073 | 10 | mc3001383 | 5 7 21 |
| mc1002079 | 7 10 13 15 20 21 | mc3001385 | 4 7 21 |
| mc1002083 | 1 21 | mc3001397 | 21 |
| mc1002088 | 7 9 21 | mc3001398 | 10 19 |
| mc1002092 | 7 10 21 | mc3001408 | 7 13 |
| mc1002096 | 1 2 7 9 21 | mc3001412 | 10 21 |
| mc1002097 | 1 7 11 21 24 | mc3001415 | 1 6 7 11 12 21 |
| mc1002098 | 1 2 7 9 11 21 | mc3001419 | 13 21 |
| mc1002100 | 7 9 11 24 | mc3001428 | 4 7 15 16 21 |
| mc1002113 | 7 9 21 | mc3001431 | 7 13 19 21 23 |
| mc1002114 | 1 6 7 21 | mc3001432 | 16 |
| mc1002117 | 7 12 21 23 | mc3001439 | 10 13 15 16 20 21 |
| mc1002118 | 7 12 21 | mc3001441 | 12 15 |
| mc1002119 | 7 12 13 | mc3001442 | 10 21 |
| mc1002120 | 7 13 17 21 | mc3001443 | 3 7 21 |
| mc1002122 | 7 9 10 21 | mc3001446 | 24 |
| mc1002124 | 7 9 17 21 | mc3001447 | 3 7 10 21 |
| mc1002126 | 7 9 21 | mc3001450 | 4 7 21 |
| mc1002130 | 7 12 21 | mc3001452 | 4 6 7 21 |
| mc1002132 | 10 12 13 15 21 | mc3001457 | 21 |
| mc1002136 | 2 4 7 9 18 21 | mc3001458 | 1 4 6 7 10 21 |
| mc1002142 | 1 10 21 | mc3001459 | 1 3 11 21 |
| mc1002147 | 7 10 21 | mc3001461 | 6 9 17 21 |
| mc1002153 | 12 18 21 23 24 | mc3001465 | 1 6 8 10 |
| mc1002177 | 1 7 9 21 | mc3001468 | 4 7 21 |
| mc1002178 | 11 21 | mc3001469 | 1 6 |
| mc1002186 | 4 7 12 16 18 20 21 | mc3001473 | 9 21 |
| mc1002207 | 3 21 | mc3001484 | 6 7 10 15 16 21 |
| mc1002209 | 8 10 | mc3001491 | 7 9 12 21 24 |
| mc1002212 | 8 10 21 | mc3001495 | 3 4 7 21 |
| mc1002217 | 1 21 | mc3001500 | 7 10 21 |
| mc1002218 | 1 6 7 10 12 15 | mc3001504 | 7 8 |
| mc1002219 | 1 | mc3001505 | 16 21 24 |
| mc1002220 | 1 6 7 10 21 | mc3001509 | 1 2 7 11 21 |
| mc1002223 | 7 8 10 | mc3001520 | 21 |
| mc1002233 | 1 6 7 8 10 | mc3001535 | 9 10 12 21 |
| mc1002235 | 10 21 | mc3001539 | 4 7 16 21 |
| mc1002237 | 10 | mc3001541 | 1 7 21 |
| mc1002238 | 7 8 9 10 12 13 15 21 | mc3001543 | 15 18 19 20 21 |
| mc1002241 | 1 2 7 9 21 | mc3001548 | 10 |
| mc1002242 | 7 12 20 21 22 23 24 | mc3001549 | 11 21 |
| mc1002244 | 4 7 10 21 | mc3001560 | 1 |
| mc1002246 | 7 9 13 21 | mc3001561 | 7 8 10 21 |
| mc1002247 | 7 8 9 10 12 13 15 21 | mc3001563 | 10 |
| mc1002248 | 2 7 9 21 | mc3001566 | 9 12 21 |
| mc1002249 | 6 7 21 | mc3001569 | 6 7 8 11 13 21 |
| mc1002250 | 1 7 21 | mc3001570 | 9 10 21 |
| mc1002251 | 1 7 21 23 24 | mc3001572 | 21 |
| mc1002252 | 6 | mc3001573 | 7 10 12 16 21 |
| mc1002253 | 6 | mc3001575 | 5 7 21 |
| mc1002255 | 4 7 15 21 | mc3001580 | 4 7 21 |
| mc1002256 | 1 21 | mc3001582 | 9 12 21 24 |
| mc1002257 | 6 7 17 21 | mc3001583 | 7 8 10 |
| mc1002262 | 4 7 21 | mc3001586 | 7 9 17 21 |
| mc1002264 | 7 12 17 19 | mc3001591 | 1 |
| mc1002268 | 7 12 21 | mc3001596 | 7 8 9 10 12 |
| mc1002269 | 10 21 | mc3001600 | 7 15 16 21 |
| mc1002271 | 7 10 11 12 21 | mc3001602 | 1 21 |
| mc1002273 | 10 | mc3001621 | 1 2 21 24 |
| mc1002275 | 21 | mc3001625 | 1 7 9 12 17 20 21 |
| mc1002277 | 10 13 21 22 | mc3001630 | 8 10 12 20 |
| mc1002278 | 21 | mc3001632 | 1 6 |
| mc1002279 | 10 21 | mc3001636 | 7 9 21 |
| mc1002282 | 4 7 21 | mc3001637 | 1 21 |
| mc1002283 | 6 7 21 | mc3001638 | 7 12 21 |
| mc1002284 | 7 9 17 21 | mc3001642 | 10 |
| mc1002285 | 6 7 17 21 | mc3001645 | 4 21 |
| mc1002288 | 7 9 10 15 21 | mc3001646 | 1 21 |
| mc1002293 | 7 21 | mc3001651 | 9 |
| mc1002295 | 9 12 21 | mc3001652 | 9 21 |
| mc1002296 | 1 | mc3001655 | 1 10 21 |
| mc1002299 | 21 | mc3001656 | 21 |
| mc1002300 | 16 21 22 | mc3001670 | 1 2 7 9 21 |
| mc1002308 | 7 13 21 | mc3001675 | 4 7 21 |
| mc1002309 | 7 21 | mc3001691 | 7 9 21 |
| mc1002311 | 10 13 21 | mc3001692 | 7 13 |
| mc1002317 | 1 7 8 9 10 13 15 21 | mc3001700 | 10 13 |
| mc1002331 | 4 7 8 11 13 15 18 21 | mc3001701 | 7 9 21 |
| mc1002334 | 13 21 | mc3001706 | 10 |
| mc1002336 | 13 21 | mc3001707 | 1 7 9 12 13 17 21 |
| mc1002348 | 7 17 21 | mc3001711 | 1 |
| mc1002350 | 21 | mc3001716 | 10 |
| mc1002355 | 15 21 | mc3001720 | 1 7 10 |
| mc1002365 | 1 2 21 | mc3001722 | 7 9 |
| mc1002366 | 1 7 9 21 | mc3001726 | 7 21 23 |
| mc1002367 | 6 10 12 13 | mc3001729 | 7 21 23 |
| mc1002375 | 1 3 7 | mc3001735 | 7 9 17 21 |
| mc1002390 | 21 | mc3001737 | 6 7 10 21 |
| mc1002394 | 4 7 21 | mc3001751 | 1 16 21 |
| mc1002418 | 1 3 7 21 | mc3001759 | 7 17 21 23 |
| mc1002425 | 4 21 | mc3001772 | 4 7 12 15 16 18 20 21 |
| mc1002430 | 1 | mc3001778 | 7 10 |
| mc1002432 | 4 7 21 | mc3001795 | 4 7 10 12 16 19 20 21 |
| mc1002437 | 2 6 21 | mc3001799 | 3 6 7 12 20 21 |
| mc1002438 | 7 12 15 21 | mc3001805 | 1 21 |
| mc1002445 | 1 3 6 7 11 15 17 21 | mc3001809 | 6 7 10 15 21 |
| mc1002447 | 10 | mc3001812 | 6 10 13 |
| mc1002448 | 4 7 16 19 20 21 | mc3001813 | 4 7 21 |
| mc1002450 | 1 7 9 17 21 | mc3001814 | 6 10 13 21 |
| mc1002454 | 7 15 16 | mc3001816 | 6 15 21 |
| mc1002455 | 1 9 | mc3001818 | 7 9 17 21 |
| mc1002459 | 1 11 21 | mc3001821 | 1 7 |
| mc1002463 | 1 7 21 | mc3001842 | 4 7 10 16 18 20 21 |
| mc1002476 | 7 10 21 | mc3001843 | 1 2 21 |
| mc1002478 | 12 17 21 | mc3001846 | 1 21 |
| mc1002481 | 5 6 7 9 10 21 | mc3001849 | 1 7 |
| mc1002484 | 6 7 | mc3001856 | 10 16 21 |
| mc1002489 | 3 15 | mc3001862 | 5 7 15 21 |
| mc1002491 | 6 7 24 | mc3001867 | 7 10 12 21 |
| mc1002506 | 1 6 8 10 11 21 | mc3001868 | 1 21 |
| mc1002520 | 4 7 18 21 22 | mc3001874 | 1 7 21 |
| mc1002522 | 21 | mc3001887 | 10 21 |
| mc1002524 | 1 6 7 10 21 | mc3001891 | 2 3 7 9 21 |
| mc1002527 | 8 | mc3001892 | 1 |
| mc1002528 | 5 7 21 | mc3001894 | 9 |
| mc1002529 | 1 4 7 10 | mc3001895 | 9 |
| mc1002543 | 1 | mc3001900 | 1 6 21 |
| mc1002554 | 3 7 10 12 15 16 17 19 20 21 | mc3001901 | 4 7 15 21 |
| mc1002585 | 4 7 8 13 21 | mc3001904 | 9 21 |
| mc1002594 | 7 9 | mc3001925 | 21 |
| mc1002599 | 7 9 10 12 21 | mc3001930 | 3 7 17 21 |
| mc1002608 | 1 6 7 8 10 21 | mc3001933 | 6 7 8 10 |
| mc1002609 | 7 | mc3001934 | 1 7 10 21 |
| mc1002613 | 4 7 15 16 20 21 | mc3001938 | 1 6 7 9 21 |
| mc1002630 | 9 | mc3001940 | 1 |
| mc1002633 | 21 | mc3001945 | 1 7 21 24 |
| mc1002637 | 2 4 7 21 | mc3001949 | 1 7 21 24 |
| mc1002640 | 10 15 21 | mc3001951 | 6 7 |
| mc1002645 | 21 | mc3001954 | 5 7 21 |
| mc1002646 | 6 9 | mc3001960 | 3 12 |
| mc1002649 | 1 3 6 7 10 21 | mc3001962 | 7 8 10 |
| mc1002652 | 7 9 12 16 21 | mc3001974 | 1 2 7 21 24 |
| mc1002656 | 4 7 10 11 15 21 | mc3001978 | 7 10 12 15 16 20 21 |
| mc1002658 | 21 | mc3001986 | 7 23 |
| mc1002660 | 4 7 9 10 21 | mc3001988 | 7 13 |
| mc1002664 | 6 10 | mc3001992 | 7 13 |
| mc1002672 | 7 9 21 | mc3001996 | 1 7 |
| mc1002678 | 1 21 | mc3001997 | 4 7 21 |
| mc1002679 | 3 | mc3001998 | 1 6 7 10 21 |
| mc1002680 | 16 17 21 23 | mc3002017 | 4 7 16 21 |
| mc1002681 | 6 7 10 21 | mc3002022 | 1 21 |
| mc1002699 | 6 10 21 22 23 | mc3002024 | 7 21 |
| mc1002703 | 11 13 21 | mc3002026 | 7 9 15 21 |
| mc1002704 | 10 13 | mc3002032 | 9 21 |
| mc1002705 | 7 10 13 21 | mc3002035 | 9 21 |
| mc1002713 | 1 6 7 10 15 17 | mc3002036 | 9 |
| mc1002715 | 1 9 | mc3002037 | 9 21 |
| mc1002716 | 7 21 | mc3002045 | 7 12 13 15 20 21 |
| mc1002718 | 6 7 11 | mc3002049 | 1 6 7 |
| mc1002719 | 1 7 17 21 | mc3002056 | 9 13 20 21 |
| mc1002721 | 6 | mc3002057 | 9 |
| mc1002725 | 3 4 7 | mc3002058 | 10 |
| mc1002729 | 1 7 | mc3002060 | 7 12 |
| mc1002732 | 4 7 21 | mc3002061 | 1 2 11 21 |
| mc1002736 | 4 7 21 | mc3002066 | 1 21 |
| mc11000004 | 6 7 9 21 | mc3002067 | 6 21 |
| mc11000005 | 7 | mc3002072 | 1 6 7 10 21 |
| mc11000007 | 4 7 21 | mc3002129 | 7 10 21 |
| mc11000009 | 1 6 7 16 | mc3002133 | 13 21 22 |
| mc11000012 | 1 7 21 | mc3002134 | 13 |
| mc11000013 | 1 10 21 | mc3002135 | 10 |
| mc11000014 | 7 21 | mc3002142 | 4 7 21 |
| mc11000033 | 7 9 21 | mc3002143 | 7 17 21 |
| mc11000035 | 3 5 17 21 | mc3002152 | 6 21 |
| mc11000036 | 1 16 21 | mc3002153 | 1 6 21 |
| mc11000048 | 4 6 7 21 | mc3002164 | 1 6 21 |
| mc11000049 | 6 7 21 | mc3002170 | 10 |
| mc11000054 | 1 6 7 10 13 15 21 | mc3002171 | 1 3 7 11 16 20 21 |
| mc11000058 | 1 6 7 8 10 | mc3002172 | 1 6 7 |
| mc11000060 | 4 | mc3002173 | 1 2 7 21 |
| mc11000061 | 2 7 9 21 | mc3002179 | 4 7 20 21 |
| mc11000065 | 3 7 8 17 21 | mc3002186 | 2 7 9 21 24 |
| mc11000066 | 21 | mc3002207 | 7 12 15 19 21 24 |
| mc11000067 | 7 18 | mc3002221 | 7 9 10 13 21 |
| mc11000068 | 7 17 21 | mc3002223 | 1 |
| mc11000069 | 6 7 9 17 21 | mc3002225 | 7 21 |
| mc11000072 | 4 7 21 | mc3002235 | 10 |
| mc11000074 | 7 9 21 | mc3002236 | 1 4 5 12 13 |
| mc11000076 | 10 | mc3002238 | 8 9 10 13 21 |
| mc11000082 | 4 7 21 | mc3002239 | 1 3 4 6 7 21 |
| mc11000085 | 4 7 | mc3002244 | 10 |
| mc11000089 | 5 21 | mc3002248 | 7 |
| mc11000092 | 7 9 10 13 21 | mc3002258 | 4 7 10 21 |
| mc11000094 | 1 3 7 18 21 | mc3002263 | 1 7 21 |
| mc11000095 | 4 6 7 18 19 | mc3002264 | 21 |
| mc11000097 | 2 7 9 17 19 21 | mc3002268 | 7 10 21 |
| mc11000098 | 1 2 7 10 21 24 | mc3002269 | 4 5 7 21 |
| mc11000100 | 6 7 10 13 15 23 24 | mc3002272 | 1 |
| mc11000105 | 1 9 10 | mc4000001 | 1 |
| mc11000107 | 3 4 5 7 21 | mc4000002 | 1 7 10 21 |
| mc11000110 | 1 2 7 21 | mc4000007 | 5 7 21 |
| mc11000117 | 4 7 21 | mc4000009 | 7 21 |
| mc11000118 | 7 | mc4000032 | 1 2 7 9 21 |
| mc11000119 | 20 | mc4000034 | 1 6 7 10 12 17 21 |
| mc11000122 | 8 21 | mc4000035 | 7 15 |
| mc11000123 | 9 10 21 | mc4000049 | 1 2 7 10 21 |
| mc11000131 | 7 15 21 | mc4000069 | 7 21 23 |
| mc11000132 | 7 15 21 | mc4000071 | 7 10 15 19 20 21 |
| mc11000169 | 21 | mc4000092 | 11 15 21 |
| mc11000170 | 16 21 | mc4000097 | 7 |
| mc11000177 | 4 7 19 20 21 | mc4000107 | 7 9 12 17 20 21 24 |
| mc11000178 | 7 | mc4000141 | 9 |
| mc11000179 | 1 7 | mc4000156 | 7 8 12 21 22 23 24 |
| mc11000181 | 7 10 15 21 | mc4000157 | 1 2 7 21 |
| mc11000186 | 16 | mc4000158 | 3 7 11 21 |
| mc11000218 | 6 7 8 10 21 | mc4000161 | 1 6 7 10 15 |
| mc11000222 | 7 10 | mc4000177 | 1 7 9 10 12 13 21 |
| mc11000229 | 7 | mc4000184 | 9 |
| mc11000231 | 4 7 8 10 21 | mc4000186 | 1 7 9 16 21 |
| mc11000269 | 7 8 10 15 16 21 | mc4000187 | 1 6 21 |
| mc11000270 | 6 7 12 17 21 | mc4000194 | 3 4 21 |
| mc11000271 | 7 9 10 21 | mc4000198 | 1 6 21 |
| mc11000275 | 9 21 22 23 24 | mc4000204 | 6 |
| mc11000282 | 3 4 | mc4000226 | 10 21 |
| mc11000287 | 9 | mc4000230 | 4 7 8 21 |
| mc11000299 | 9 | mc4000236 | 1 6 21 |
| mc11000300 | 1 4 7 | mc4000239 | 21 |
| mc11000304 | 1 7 21 | mc4000257 | 1 6 7 8 10 21 24 |
| mc11000309 | 4 7 16 20 21 | mc4000280 | 1 6 7 10 |
| mc11000310 | 7 | mc4000289 | 4 7 21 |
| mc11000316 | 1 6 7 12 13 16 21 | mc4000290 | 9 10 21 |
| mc11000318 | 3 4 7 8 12 21 | mc4000291 | 7 8 15 |
| mc11000320 | 7 21 | mc4000295 | 4 7 |
| mc11000322 | 6 7 11 21 | mc4000301 | 6 7 21 |
| mc11000332 | 4 6 7 9 21 | mc4000304 | 7 9 10 12 21 23 24 |
| mc11000334 | 6 11 | mc4000308 | 1 4 6 21 |
| mc11000337 | 6 7 9 21 | mc4000317 | 10 |
| mc11000339 | 4 7 21 | mc4000328 | 3 4 7 21 |
| mc11000342 | 1 4 7 | mc4000329 | 5 7 |
| mc11000348 | 4 7 8 18 21 | mc4000335 | 7 21 |
| mc11000373 | 3 6 7 11 16 21 | mc4000337 | 10 21 |
| mc11000374 | 1 6 7 21 | mc4000342 | 7 10 11 12 13 15 21 |
| mc11000395 | 7 12 21 | mc4000350 | 6 |
| mc11000397 | 1 2 4 7 | mc4000385 | 1 4 5 7 21 |
| mc11000405 | 5 7 21 | mc4000386 | 3 4 7 12 13 |
| mc11000418 | 1 6 7 17 21 | mc4000388 | 1 4 7 11 15 21 |
| mc11000424 | 1 7 | mc4000393 | 3 7 11 21 |
| mc11000429 | 7 21 | mc4000395 | 6 9 11 21 |
| mc11000432 | 10 21 | mc4000396 | 4 21 |
| mc11000433 | 10 | mc4000398 | 1 7 16 17 21 |
| mc11000436 | 4 7 10 | mc4000401 | 6 7 11 15 18 21 |
| mc11000437 | 10 21 | mc4000402 | 9 21 |
| mc11000447 | 7 10 21 | mc4000403 | 4 5 7 |
| mc11000449 | 7 10 15 18 21 | mc4000404 | 9 21 |
| mc11000450 | 4 6 7 21 | mc4000405 | 9 21 |
| mc11000451 | 10 | mc4000407 | 6 7 |
| mc11000453 | 3 7 21 | mc4000414 | 1 |
| mc11000455 | 7 9 13 | mc4000418 | 2 7 9 17 21 |
| mc11000456 | 7 13 16 21 22 23 | mc4000420 | 10 21 |
| mc11000459 | 7 9 13 | mc4000422 | 3 9 17 |
| mc11000460 | 10 | mc4000424 | 10 21 |
| mc11000465 | 1 6 7 10 | mc4000428 | 6 7 11 |
| mc11000478 | 7 10 15 20 | mc4000431 | 1 6 7 9 15 21 |
| mc11000479 | 3 4 5 7 11 17 21 | mc4000432 | 3 7 11 21 |
| mc11000480 | 4 7 12 13 15 16 18 20 21 | mc4000433 | 1 7 21 |
| mc11000482 | 6 7 9 10 13 21 | mc4000434 | 9 10 21 |
| mc11000486 | 9 10 12 21 | mc4000436 | 7 9 10 12 |
| mc11000494 | 9 21 | mc4000437 | 7 9 21 |
| mc11000495 | 10 13 15 | mc4000442 | 1 6 7 10 |
| mc11000498 | 4 7 20 21 | mc4000443 | 7 11 21 |
| mc11000515 | 5 7 | mc4000444 | 21 |
| mc11000552 | 1 | mc4000447 | 1 2 7 |
| mc11000553 | 7 10 | mc4000449 | 7 17 21 |
| mc11000555 | 3 8 21 | mc4000460 | 10 13 21 |
| mc11000563 | 7 9 10 12 21 23 24 | mc4000463 | 10 13 21 |
| mc11000568 | 7 9 10 12 21 23 24 | mc4000470 | 21 |
| mc11000579 | 1 3 8 11 21 | mc4000472 | 7 9 21 |
| mc11000584 | 1 7 9 | mc4000473 | 1 2 6 7 |
| mc11000587 | 7 | mc4000476 | 6 7 21 |
| mc11000588 | 7 10 21 | mc4000478 | 1 6 7 10 21 |
| mc11000600 | 6 | mc4000480 | 4 7 16 18 21 22 24 |
| mc11000606 | 4 7 16 21 | mc4000487 | 1 7 |
| mc11000630 | 4 7 21 | mc4000490 | 1 4 7 21 |
| mc11000632 | 4 7 21 | mc4000498 | 1 3 4 7 21 |
| mc11000634 | 6 7 18 20 21 23 24 | mc4000508 | 1 7 9 21 |
| mc11000636 | 7 12 21 24 | mc4000509 | 7 |
| mc11000641 | 1 6 7 10 | mc4000513 | 7 |
| mc11000643 | 4 7 8 10 21 | mc4000514 | 7 11 17 21 |
| mc11000645 | 1 6 7 10 | mc4000517 | 1 3 7 11 21 |
| mc11000650 | 10 21 | mc4000522 | 4 7 20 21 |
| mc11000656 | 6 7 13 17 19 21 23 | mc4000525 | 21 |
| mc11000676 | 6 10 13 21 | mc4000527 | 3 21 |
| mc11000680 | 6 7 21 | mc4000528 | 1 21 |
| mc11000682 | 7 11 21 | mc4000529 | 7 |
| mc11000689 | 7 11 21 | mc4000530 | 7 10 17 21 |
| mc11000690 | 10 13 21 | mc4000540 | 1 7 21 |
| mc11000695 | 4 7 21 | mc4000542 | 7 9 12 21 |
| mc11000696 | 1 6 21 | mc4000545 | 1 6 |
| mc11000713 | 1 6 7 10 21 | mc4000546 | 6 9 21 |
| mc11000716 | 15 21 | mc4000555 | 4 7 10 16 21 |
| mc11000718 | 1 7 21 | mc4000557 | 6 7 9 11 21 |
| mc11000722 | 1 7 9 21 | mc4000558 | 4 7 10 16 21 |
| mc11000726 | 6 7 15 21 | mc4000563 | 8 15 |
| mc11000732 | 4 6 7 8 10 13 15 21 | mc4000573 | 1 21 |
| mc11000734 | 1 | mc4000576 | 1 2 21 24 |
| mc11000736 | 4 7 8 10 21 | mc4000579 | 7 |
| mc11000737 | 6 7 11 12 21 | mc4000611 | 7 9 21 |
| mc11000740 | 7 21 | mc4000612 | 1 7 9 13 17 21 |
| mc11000741 | 7 9 10 21 | mc4000618 | 9 |
| mc11000748 | 6 7 21 | mc4000644 | 7 21 |
| mc11000749 | 4 7 21 | mc4000651 | 1 3 6 11 16 21 |
| mc11000760 | 4 7 15 16 21 | mc4000654 | 4 7 21 |
| mc11000777 | 1 6 7 10 21 | mc4000674 | 1 7 |
| mc11000780 | 1 | mc4000676 | 1 7 10 12 21 |
| mc11000781 | 4 7 21 | mc4000680 | 7 9 17 21 |
| mc11000782 | 4 5 7 21 | mc4000691 | 6 7 8 10 |
| mc11000786 | 9 21 | mc4000693 | 9 11 21 |
| mc11000787 | 7 9 17 21 | mc4000694 | 9 |
| mc11000793 | 3 6 7 11 16 17 21 | mc4000699 | 1 4 6 7 9 10 15 16 21 |
| mc11000796 | 3 6 7 8 10 15 21 | mc4000701 | 10 21 |
| mc11000801 | 6 7 | mc4000708 | 10 13 |
| mc11000802 | 4 7 10 15 21 | mc4000709 | 7 21 |
| mc11000820 | 21 | mc4000713 | 1 7 21 |
| mc11000821 | 13 | mc4000715 | 7 10 |
| mc11000822 | 2 7 9 21 | mc4000718 | 1 19 21 |
| mc11000823 | 15 21 | mc4000723 | 21 |
| mc11000825 | 7 | mc4000729 | 10 |
| mc11000827 | 3 7 | mc4000732 | 9 21 |
| mc11000829 | 7 9 | mc4000742 | 7 9 |
| mc11000830 | 1 4 5 7 8 10 13 15 18 21 | mc4000744 | 4 7 18 21 |
| mc11000831 | 7 10 13 15 21 | mc4000747 | 7 9 21 |
| mc11000833 | 1 3 7 11 17 21 | mc4000749 | 9 21 |
| mc11000834 | 6 7 10 13 15 21 | mc4000753 | 6 9 10 |
| mc11000835 | 4 7 13 15 21 | mc4000754 | 7 |
| mc11000840 | 9 21 24 | mc4000755 | 21 |
| mc11000842 | 7 10 | mc4000756 | 1 7 21 |
| mc11000844 | 1 6 7 21 | mc4000757 | 1 3 7 21 |
| mc11000847 | 7 10 13 15 18 21 | mc4000759 | 7 8 10 21 |
| mc11000848 | 6 7 10 13 15 21 | mc4000767 | 7 8 9 13 21 |
| mc11000859 | 1 | mc4000769 | 2 7 9 17 21 |
| mc11000862 | 21 | mc4000774 | 7 9 13 21 |
| mc11000864 | 7 11 21 | mc4000788 | 7 8 15 21 |
| mc11000865 | 21 | mc4000789 | 7 13 15 21 |
| mc11000866 | 7 9 13 21 | mc4000792 | 16 21 |
| mc11000868 | 1 8 13 21 | mc4000801 | 7 21 |
| mc11000871 | 9 21 | mc4000813 | 7 8 10 11 13 |
| mc11000877 | 7 12 17 21 | mc4000822 | 21 |
| mc11000878 | 6 7 9 11 12 21 | mc4000840 | 7 |
| mc11000887 | 10 | mc4000864 | 21 |
| mc11000898 | 7 9 10 12 21 22 24 | mc4000868 | 4 7 |
| mc11000906 | 1 6 7 21 | mc4000891 | 1 7 |
| mc11000909 | 4 7 16 19 20 21 | mc4000896 | 7 12 17 |
| mc11000916 | 4 21 | mc4000929 | 1 7 |
| mc11000917 | 4 7 21 | mc4000930 | 7 10 15 16 21 |
| mc11000918 | 5 21 | mc4000932 | 7 10 12 16 21 |
| mc11000920 | 21 | mc4000940 | 4 7 8 21 |
| mc11000945 | 10 13 | mc4000941 | 4 7 21 |
| mc11000949 | 10 13 | mc4000955 | 4 7 21 |
| mc11000962 | 4 7 15 16 21 | mc4000985 | 7 15 21 |
| mc11000970 | 7 8 21 | mc4000988 | 7 9 21 |
| mc11000971 | 7 8 21 | mc4000992 | 5 7 21 |
| mc11000977 | 5 21 | mc4000996 | 9 12 13 21 22 24 |
| mc11000979 | 7 9 10 17 21 | mc4001005 | 4 16 21 |
| mc11000982 | 10 16 17 21 | mc4001012 | 7 10 11 12 15 21 |
| mc11000986 | 7 12 21 22 | mc4001029 | 7 10 11 12 15 21 |
| mc11000990 | 4 7 21 | mc4001033 | 12 15 21 |
| mc11000992 | 15 | mc4001037 | 1 |
| mc11001002 | 6 7 8 16 21 | mc4001039 | 1 3 4 7 8 15 19 21 |
| mc11001004 | 4 7 21 | mc4001041 | 3 7 8 21 |
| mc11001005 | 7 9 10 | mc4001048 | 7 21 |
| mc11001006 | 1 21 24 | mc4001077 | 1 7 8 10 13 |
| mc11001011 | 4 7 15 20 21 | mc4001081 | 6 7 8 10 12 16 21 24 |
| mc11001013 | 1 4 7 21 | mc4001086 | 7 21 |
| mc11001014 | 9 21 | mc4001089 | 4 7 21 |
| mc11001017 | 6 21 | mc4001101 | 7 15 16 17 21 |
| mc11001019 | 7 | mc4001102 | 7 9 |
| mc11001025 | 16 17 21 | mc4001111 | 1 7 9 21 24 |
| mc11001026 | 4 7 16 17 21 | mc4001127 | 3 4 7 21 |
| mc11001029 | 3 7 21 24 | mc4001134 | 7 15 |
| mc11001032 | 1 21 | mc4001141 | 7 |
| mc11001035 | 10 21 | mc4001144 | 6 |
| mc11001038 | 1 6 7 10 13 15 | mc4001149 | 1 2 13 15 20 21 24 |
| mc11001044 | 10 13 18 21 | mc4001153 | 6 |
| mc11001048 | 1 7 9 21 | mc4001164 | 4 7 16 21 |
| mc11001053 | 7 | mc4001168 | 4 10 15 21 |
| mc11001054 | 7 12 21 | mc4001170 | 1 2 7 |
| mc11001055 | 1 3 6 7 10 20 | mc4001172 | 1 6 7 10 21 |
| mc11001057 | 21 | mc4001195 | 21 |
| mc11001058 | 7 9 | mc4001197 | 10 |
| mc11001061 | 10 21 | mc4001198 | 7 10 12 21 |
| mc11001065 | 1 2 | mc4001221 | 10 21 |
| mc11001068 | 6 7 8 10 21 24 | mc4001224 | 1 7 9 |
| mc11001081 | 7 10 21 | mc4001246 | 7 8 10 12 15 16 |
| mc11001083 | 7 | mc4001254 | 13 15 21 |
| mc11001085 | 4 7 10 21 | mc4001255 | 13 15 21 |
| mc11001088 | 6 21 | mc4001260 | 1 10 16 20 21 |
| mc11001091 | 4 7 9 11 12 21 | mc4001276 | 1 6 7 8 18 21 |
| mc11001101 | 7 21 | mc4001277 | 12 |
| mc11001106 | 7 12 20 21 22 23 24 | mc4001279 | 1 6 9 10 21 |
| mc11001118 | 1 2 6 7 21 | mc4001282 | 5 7 21 |
| mc11001126 | 1 6 21 | mc4001283 | 7 |
| mc11001137 | 1 3 4 7 21 | mc4001288 | 1 |
| mc11001142 | 4 7 8 15 19 20 21 | mc4001291 | 5 21 |
| mc11001152 | 1 6 7 8 10 11 | mc4001301 | 1 7 21 |
| mc11001154 | 4 7 21 | mc4001308 | 4 7 16 21 |
| mc11001186 | 7 15 16 17 21 | mc4001313 | 7 9 |
| mc11001188 | 10 | mc4001314 | 3 7 9 16 |
| mc11001189 | 7 12 13 | mc4001316 | 1 2 21 |
| mc11001195 | 7 9 21 | mc4001317 | 9 12 21 |
| mc11001197 | 7 12 15 16 17 21 | mc4001319 | 7 9 21 |
| mc11001201 | 21 | mc4001320 | 1 |
| mc11001207 | 7 9 15 21 22 24 | mc4001326 | 11 21 |
| mc11001209 | 5 7 21 | mc4001327 | 1 7 21 |
| mc11001210 | 23 | mc4001329 | 3 4 7 21 |
| mc11001212 | 7 9 | mc4001334 | 2 9 21 |
| mc11001213 | 7 8 9 21 | mc4001337 | 6 7 |
| mc11001222 | 4 10 12 21 | mc4001339 | 9 |
| mc11001223 | 4 10 12 16 20 21 | mc4001343 | 6 7 10 21 |
| mc11001224 | 1 7 9 13 21 24 | mc4001345 | 6 7 21 |
| mc11001225 | 1 7 9 13 21 24 | mc4001348 | 3 7 8 21 |
| mc11001226 | 1 7 9 13 21 24 | mc4001349 | 15 21 |
| mc11001228 | 1 6 7 10 12 13 21 | mc4001367 | 9 |
| mc11001232 | 7 9 21 | mc4001392 | 4 7 21 |
| mc11001240 | 12 13 15 16 21 | mc4001393 | 4 7 18 19 20 21 |
| mc11001244 | 3 4 7 11 15 21 | mc4001394 | 1 7 21 |
| mc11001245 | 7 9 12 21 | mc4001395 | 16 19 23 |
| mc11001246 | 1 7 21 | mc4001397 | 4 7 15 16 18 19 21 |
| mc11001248 | 7 9 21 | mc4001398 | 21 |
| mc11001249 | 4 7 21 | mc4001417 | 1 2 7 9 21 |
| mc11001250 | 1 | mc4001423 | 6 21 |
| mc11001251 | 21 | mc4001425 | 1 5 6 7 10 |
| mc11001253 | 6 7 21 | mc4001429 | 4 7 16 21 |
| mc11001254 | 7 13 15 21 | mc4001431 | 1 21 |
| mc11001257 | 4 7 21 | mc4001432 | 1 |
| mc11001260 | 9 10 12 21 | mc4001433 | 2 7 9 21 |
| mc11001261 | 7 10 15 | mc4001435 | 6 |
| mc11001264 | 7 12 | mc4001436 | 3 4 7 11 21 |
| mc11001265 | 7 | mc4001438 | 1 6 7 21 |
| mc11001266 | 1 6 7 10 | mc4001440 | 21 |
| mc11001273 | 5 15 21 | mc4001444 | 1 6 7 8 10 16 18 24 |
| mc11001275 | 4 5 7 21 | mc4001446 | 7 21 |
| mc11001279 | 7 17 21 | mc4001450 | 7 21 |
| mc11001280 | 21 | mc4001451 | 1 7 |
| mc11001281 | 21 | mc4001455 | 1 6 7 10 21 |
| mc11001282 | 7 9 17 21 | mc4001457 | 1 3 7 11 21 |
| mc11001284 | 7 10 21 | mc4001461 | 1 |
| mc11001285 | 1 2 7 21 | mc4001462 | 6 7 |
| mc11001286 | 6 7 10 12 16 18 19 21 22 23 24 | mc4001464 | 10 |
| mc11001287 | 7 9 10 | mc4001467 | 1 6 7 |
| mc11001289 | 7 | mc4001470 | 5 7 21 |
| mc11001293 | 4 7 21 | mc4001471 | 3 7 21 |
| mc11001296 | 1 7 9 13 21 | mc4001482 | 1 7 21 |
| mc11001299 | 1 7 9 13 21 22 | mc4001483 | 3 4 7 8 15 21 |
| mc11001304 | 10 12 13 21 | mc4001487 | 4 7 16 21 |
| mc11001305 | 4 7 21 | mc4001489 | 7 9 12 21 22 23 |
| mc11001306 | 10 12 13 15 21 | mc4001492 | 1 2 7 9 21 |
| mc11001308 | 10 12 15 21 24 | mc4001494 | 6 7 9 21 |
| mc11001311 | 6 7 8 11 13 21 | mc4001495 | 7 10 12 15 16 20 21 |
| mc11001312 | 1 9 10 21 | mc4001496 | 1 6 21 |
| mc11001314 | 1 6 7 10 18 | mc4001497 | 4 7 |
| mc11001315 | 9 10 12 21 | mc4001504 | 4 7 10 21 |
| mc11001316 | 10 12 13 21 | mc4001505 | 1 21 |
| mc11001317 | 7 9 21 | mc4001506 | 3 6 7 21 |
| mc11001319 | 6 7 8 17 | mc4001508 | 3 4 7 10 21 |
| mc11001320 | 1 2 7 21 | mc4001509 | 6 7 8 10 12 16 21 24 |
| mc11001321 | 7 21 | mc4001514 | 3 4 5 7 21 |
| mc11001322 | 4 7 | mc4001533 | 1 2 9 21 |
| mc11001324 | 7 9 21 | mc4001536 | 9 21 24 |
| mc11001328 | 7 17 21 | mc4001538 | 21 |
| mc11001329 | 9 21 | mc4001543 | 7 17 |
| mc11001331 | 7 13 21 | mc4001544 | 4 7 21 23 |
| mc11001332 | 3 4 5 7 21 | mc4001545 | 6 21 |
| mc11001333 | 21 | mc4001549 | 1 |
| mc11001334 | 3 21 | mc4001552 | 7 |
| mc11001346 | 6 7 12 13 15 18 22 | mc4001558 | 8 21 |
| mc11001350 | 2 9 | mc4001559 | 1 8 9 13 21 |
| mc11001351 | 4 7 21 | mc4001568 | 11 13 20 21 |
| mc11001355 | 7 17 21 | mc4001571 | 4 7 21 |
| mc11001358 | 2 3 4 7 9 21 | mc4001572 | 4 16 21 |
| mc11001364 | 7 9 21 | mc4001574 | 1 |
| mc11001368 | 1 7 9 21 | mc4001576 | 4 8 21 |
| mc11001369 | 7 9 10 15 | mc4001579 | 4 7 21 |
| mc11001370 | 6 7 10 | mc4001588 | 7 9 12 17 20 21 |
| mc11001373 | 7 10 12 21 | mc4001589 | 6 21 |
| mc11001374 | 1 3 6 7 10 12 21 | mc4001604 | 21 |
| mc11001375 | 7 9 10 21 | mc4001608 | 6 13 21 |
| mc11001377 | 4 7 15 21 | mc4001612 | 3 4 7 21 |
| mc11001378 | 9 21 | mc4001614 | 10 15 16 |
| mc11001379 | 1 | mc4001616 | 1 2 21 24 |
| mc11001380 | 7 9 10 11 12 13 | mc4001618 | 6 7 21 |
| mc11001381 | 7 9 10 11 12 | mc4001622 | 4 7 21 |
| mc11001382 | 1 | mc4001644 | 4 7 18 21 24 |
| mc11001421 | 10 13 21 | mc4001645 | 7 9 |
| mc11001422 | 10 13 | mc4001659 | 4 7 21 |
| mc11001440 | 3 7 9 12 15 16 17 21 | mc4001660 | 7 17 19 21 23 |
| mc11001443 | 3 4 7 15 21 | mc4001662 | 4 7 21 |
| mc11001447 | 1 13 24 | mc4001664 | 1 10 21 |
| mc11001456 | 4 7 16 21 | mc4001665 | 4 7 21 |
| mc11001464 | 3 7 21 | mc4001675 | 2 9 21 |
| mc11001466 | 8 21 | mc4001677 | 5 7 21 |
| mc11001467 | 8 13 21 | mc4001678 | 2 7 9 12 17 21 |
| mc11001473 | 4 7 21 | mc4001679 | 4 7 10 21 |
| mc11001477 | 9 21 | mc4001698 | 7 10 12 21 |
| mc11001478 | 7 9 | mc4001699 | 5 21 |
| mc11001482 | 2 7 9 17 21 | mc4001701 | 4 7 21 |
| mc11001483 | 3 7 8 10 15 21 | mc4001708 | 4 7 8 10 |
| mc11001484 | 3 6 7 8 10 16 | mc4001712 | 9 21 |
| mc11001485 | 7 9 10 21 | mc4001713 | 7 12 19 21 |
| mc11001487 | 7 9 12 21 22 24 | mc4001717 | 7 12 17 21 |
| mc11001491 | 9 21 | mc4001723 | 5 |
| mc11001493 | 4 7 21 | mc4001724 | 3 7 11 21 |
| mc11001494 | 1 7 9 | mc4001726 | 6 7 8 11 13 21 |
| mc11001495 | 2 | mc4001732 | 4 7 21 |
| mc11001496 | 1 4 7 | mc4001739 | 10 12 22 23 24 |
| mc11001497 | 1 2 7 9 21 | mc4001742 | 9 10 20 21 |
| mc11001499 | 6 7 9 21 | mc4001743 | 9 10 20 21 |
| mc11001500 | 8 20 | mc4001744 | 9 10 21 |
| mc11001511 | 7 9 17 21 | mc4001745 | 9 10 21 |
| mc11001512 | 6 7 21 | mc4001767 | 7 21 |
| mc11001514 | 6 11 | mc4001773 | 1 |
| mc11001517 | 9 21 | mc4001774 | 1 7 21 |
| mc11001524 | 1 6 7 8 10 21 | mc4001781 | 5 7 |
| mc11001530 | 21 | mc4001785 | 7 17 21 23 |
| mc11001537 | 7 20 21 24 | mc4001786 | 3 4 7 15 21 |
| mc11001540 | 2 7 9 21 | mc4001788 | 7 21 |
| mc11001541 | 1 2 9 17 21 | mc4001789 | 4 7 21 |
| mc11001543 | 21 | mc4001792 | 6 16 18 22 23 24 |
| mc11001544 | 4 7 21 | mc4001794 | 1 7 |
| mc11001546 | 7 12 21 | mc4001800 | 5 |
| mc11001547 | 7 | mc4001803 | 10 |
| mc11001550 | 6 7 10 13 15 16 20 21 | mc4001809 | 3 7 8 10 |
| mc11001551 | 1 6 7 10 | mc4001817 | 10 21 24 |
| mc11001554 | 7 9 10 21 | mc4001821 | 7 12 21 |
| mc11001556 | 1 4 7 10 21 | mc4001822 | 7 |
| mc11001560 | 21 | mc4001825 | 6 9 |
| mc11001561 | 7 9 12 13 21 | mc4001829 | 1 7 9 |
| mc11001562 | 4 7 16 18 20 21 | mc4001833 | 5 7 |
| mc11001563 | 7 9 21 | mc4001837 | 21 |
| mc11001565 | 9 | mc4001852 | 1 6 8 10 12 21 |
| mc11001568 | 6 12 13 15 21 | mc4001853 | 1 7 21 |
| mc11001571 | 7 8 21 | mc4001855 | 4 7 21 |
| mc11001573 | 21 | mc4001859 | 7 17 21 |
| mc11001575 | 1 4 6 7 10 21 | mc4001863 | 7 10 21 22 23 |
| mc11001584 | 1 7 9 10 11 13 | mc4001865 | 13 15 |
| mc11001585 | 7 11 12 13 20 21 | mc4001867 | 4 7 21 |
| mc11001586 | 7 10 21 | mc4001868 | 4 7 8 21 |
| mc11001589 | 10 | mc4001872 | 3 7 8 21 |
| mc11001591 | 8 18 | mc4001882 | 3 21 |
| mc11001593 | 7 10 12 | mc4001886 | 3 7 21 |
| mc11001600 | 7 | mc4001888 | 1 3 4 7 8 16 21 |
| mc11001606 | 4 21 | mc4001889 | 7 9 21 |
| mc11001607 | 3 4 7 8 16 21 | mc4001901 | 7 10 12 17 |
| mc11001608 | 10 | mc4001903 | 10 21 |
| mc11001610 | 1 3 | mc4001906 | 1 6 8 10 |
| mc11001611 | 7 8 10 21 | mc4001907 | 7 8 9 21 |
| mc11001612 | 7 10 | mc4001909 | 7 |
| mc11001613 | 7 10 21 | mc4001910 | 7 9 12 21 |
| mc11001616 | 4 7 21 | mc4001915 | 3 7 |
| mc11001617 | 6 7 8 11 21 | mc4001916 | 4 7 10 21 |
| mc11001618 | 7 8 16 22 24 | mc4001918 | 7 |
| mc11001624 | 9 10 12 21 22 24 | mc4001919 | 3 7 10 21 23 |
| mc11001636 | 7 10 12 13 15 21 | mc4001923 | 3 4 6 7 17 21 |
| mc11001637 | 7 10 12 13 15 21 | mc4001929 | 1 |
| mc11001639 | 7 10 12 13 15 21 | mc4001931 | 21 |
| mc11001640 | 7 10 12 13 15 21 | mc4001937 | 21 |
| mc11001641 | 7 10 12 13 15 21 | mc4001939 | 21 |
| mc11001643 | 7 10 12 13 15 21 | mc4001943 | 9 12 16 21 22 23 24 |
| mc11001651 | 6 7 11 21 | mc4001945 | 4 7 21 |
| mc11001652 | 3 7 16 21 | mc4001951 | 1 21 |
| mc11001653 | 3 7 11 21 | mc4001953 | 1 |
| mc11001654 | 6 7 15 21 | mc4001956 | 7 10 |
| mc11001658 | 7 10 21 | mc4001961 | 1 7 21 |
| mc11001666 | 15 | mc4001963 | 1 7 9 10 21 |
| mc11001672 | 6 7 9 17 21 | mc4001964 | 21 |
| mc11001678 | 7 | mc4001966 | 9 |
| mc11001680 | 7 10 12 13 15 21 | mc4001967 | 21 |
| mc11001681 | 7 10 12 13 15 18 21 | mc4001970 | 7 21 |
| mc11001682 | 7 10 12 13 15 21 24 | mc4001975 | 4 7 12 16 18 20 21 |
| mc11001685 | 7 10 12 13 15 21 | mc4001980 | 4 7 21 |
| mc11001686 | 7 10 12 13 15 21 | mc4001991 | 10 |
| mc11001687 | 5 7 8 | mc4001997 | 4 21 |
| mc11001691 | 4 7 16 20 21 | mc4001998 | 10 |
| mc11001693 | 3 4 7 21 | mc4001999 | 1 11 21 |
| mc11001694 | 7 9 21 | mc4002002 | 1 |
| mc11001695 | 1 6 8 10 | mc4002005 | 4 7 21 |
| mc11001697 | 7 | mc4002006 | 5 7 21 |
| mc11001703 | 3 5 7 12 16 21 | mc4002008 | 4 7 8 21 |
| mc11001704 | 4 7 16 21 | mc4002010 | 3 4 7 15 21 |
| mc11001709 | 1 | mc4002015 | 4 7 9 21 22 |
| mc11001711 | 7 21 | mc4002020 | 21 |
| mc11001723 | 7 9 21 | mc4002021 | 2 3 4 9 21 |
| mc11001727 | 1 3 6 7 10 12 | mc4002028 | 6 7 8 13 21 23 |
| mc11001728 | 21 | mc4002029 | 6 7 8 13 21 23 |
| mc11001734 | 4 7 19 21 | mc4002030 | 6 7 8 13 21 23 |
| mc11001737 | 4 7 19 21 | mc4002034 | 1 6 7 8 10 21 24 |
| mc11001740 | 1 3 4 7 11 21 | mc4002036 | 7 21 |
| mc11001746 | 1 5 6 7 18 21 | mc4002039 | 10 16 20 21 |
| mc11001747 | 7 9 15 16 17 21 | mc4002055 | 1 21 |
| mc11001749 | 15 17 21 | mc4002066 | 7 9 21 22 23 |
| mc11001750 | 5 15 21 | mc4002067 | 6 21 |
| mc11001752 | 3 4 7 | mc4002068 | 6 7 10 21 |
| mc11001757 | 1 | mc4002078 | 1 7 13 21 |
| mc11001761 | 7 | mc4002079 | 1 7 13 21 |
| mc11001766 | 3 7 11 21 | mc4002080 | 1 7 13 21 |
| mc11001768 | 1 6 10 | mc4002081 | 1 7 13 21 |
| mc11001770 | 3 7 12 21 | mc4002082 | 1 7 21 |
| mc11001771 | 1 6 7 | mc4002084 | 1 7 13 21 |
| mc11001774 | 7 | mc4002088 | 10 |
| mc11001778 | 7 21 24 | mc4002089 | 3 21 |
| mc11001779 | 7 11 21 | mc4002093 | 7 12 13 17 21 |
| mc11001780 | 7 11 21 | mc4002095 | 1 11 21 |
| mc11001785 | 7 9 12 17 21 | mc4002099 | 5 21 |
| mc11001787 | 4 6 7 9 21 | mc4002105 | 1 9 21 |
| mc11001788 | 4 7 | mc4002106 | 10 12 13 21 |
| mc11001800 | 6 7 8 10 21 | mc4002109 | 4 7 15 16 21 |
| mc11001801 | 6 | mc4002122 | 8 |
| mc11001806 | 6 7 21 | mc4002123 | 3 21 |
| mc11001807 | 1 2 7 8 9 10 | mc4002124 | 6 7 21 |
| mc11001812 | 10 15 16 18 20 21 | mc4002125 | 6 7 |
| mc11001823 | 7 9 | mc4002126 | 6 7 |
| mc11001829 | 4 7 12 21 | mc4002130 | 6 7 |
| mc11001832 | 6 7 9 10 | mc4002131 | 1 2 7 9 21 |
| mc11001833 | 2 6 7 21 | mc4002132 | 1 7 9 |
| mc11001862 | 7 8 10 21 | mc4002133 | 21 |
| mc11001865 | 4 6 7 8 10 18 19 21 | mc4002140 | 6 |
| mc11001866 | 7 10 | mc4002144 | 1 6 7 10 18 21 |
| mc11001868 | 4 21 | mc4002146 | 9 |
| mc11001872 | 7 9 10 12 21 | mc4002150 | 4 7 16 21 |
| mc11001874 | 7 | mc4002151 | 4 7 9 10 21 |
| mc11001875 | 7 21 | mc4002156 | 7 12 15 21 |
| mc11001878 | 1 21 | mc4002166 | 6 8 15 21 |
| mc11001879 | 21 | mc4002169 | 7 |
| mc11001881 | 21 | mc4002177 | 21 |
| mc11001883 | 5 21 | mc4002192 | 12 15 20 21 |
| mc11001896 | 3 4 7 15 16 21 | mc4002239 | 1 21 |
| mc11001904 | 7 10 12 21 | mc4002247 | 1 3 10 13 15 21 |
| mc11001906 | 4 7 16 21 | mc4002248 | 10 15 21 |
| mc11001907 | 4 7 16 21 | mc4002272 | 12 21 |
| mc11001911 | 7 10 12 21 | mc4002273 | 7 16 17 21 |
| mc11001912 | 1 3 4 6 7 21 | mc4002279 | 9 |
| mc11001914 | 9 21 | mc4002280 | 1 7 |
| mc11001915 | 7 21 | mc4002283 | 7 8 13 |
| mc11001917 | 7 21 | mc4002285 | 3 7 21 |
| mc11001920 | 1 7 10 12 15 16 20 21 | mc4002286 | 6 7 21 |
| mc11001924 | 6 7 8 21 | mc4002288 | 6 7 21 |
| mc11001927 | 21 | mc4002296 | 3 21 |
| mc11001931 | 7 8 12 | mc4002297 | 1 3 5 6 7 8 10 15 |
| mc11001932 | 7 10 21 | mc4002298 | 4 5 7 21 |
| mc11001934 | 1 21 | mc4002299 | 1 |
| mc11001938 | 10 21 | mc4002302 | 4 7 21 |
| mc11001939 | 4 7 9 21 | mc4002317 | 6 7 17 21 |
| mc11001946 | 4 21 | mc4002319 | 7 8 15 21 |
| mc11001950 | 4 7 15 16 21 | mc4002321 | 21 |
| mc11001951 | 4 7 15 16 21 | mc4002323 | 1 2 7 |
| mc11001953 | 4 7 15 16 21 | mc4002324 | 7 9 17 21 |
| mc11001954 | 4 7 15 16 20 21 | mc4002326 | 6 7 21 |
| mc11001956 | 3 4 7 15 16 18 21 | mc4002328 | 7 9 21 |
| mc11001959 | 4 7 16 21 | mc4002332 | 7 10 16 21 |
| mc11001961 | 4 7 15 16 21 | mc4002333 | 7 12 |
| mc11001962 | 4 7 16 21 | mc4002334 | 1 7 10 13 21 |
| mc11001972 | 3 4 7 17 21 | mc4002337 | 9 21 |
| mc11001973 | 4 7 21 | mc4002339 | 6 10 |
| mc11001974 | 7 9 17 21 | mc4002345 | 9 21 24 |
| mc11001978 | 1 7 | mc4002347 | 1 2 7 21 |
| mc11001981 | 4 7 21 | mc4002363 | 7 10 11 21 |
| mc11001984 | 5 21 | mc4002364 | 3 6 7 11 13 21 |
| mc11001986 | 4 7 13 21 | mc4002366 | 7 13 18 21 22 |
| mc11001990 | 6 7 9 21 | mc4002367 | 10 21 |
| mc11001992 | 6 7 21 | mc4002368 | 4 10 12 21 |
| mc11001994 | 5 21 | mc4002384 | 6 7 11 21 |
| mc11001996 | 10 15 | mc4002386 | 4 7 |
| mc11002000 | 7 8 9 12 21 | mc4002389 | 10 12 13 21 |
| mc11002005 | 4 7 21 | mc4002394 | 10 21 |
| mc11002007 | 4 7 15 16 18 21 | mc4002398 | 4 7 21 |
| mc11002008 | 6 7 8 11 13 21 | mc4002400 | 1 21 23 24 |
| mc11002014 | 7 9 21 | mc4002401 | 10 |
| mc11002017 | 10 16 21 | mc4002402 | 4 7 21 |
| mc11002021 | 5 7 21 | mc4002411 | 7 10 13 17 20 |
| mc11002025 | 4 7 8 20 21 | mc4002434 | 1 3 7 15 21 |
| mc11002027 | 1 6 7 10 | mc4002438 | 4 7 11 15 |
| mc11002030 | 4 7 15 16 21 | mc4002439 | 9 |
| mc11002032 | 4 8 10 21 | mc4002447 | 4 7 21 |
| mc11002033 | 1 9 21 | mc4002462 | 6 7 21 |
| mc11002035 | 7 16 19 23 | mc4002464 | 10 |
| mc11002036 | 1 | mc4002465 | 4 7 12 15 16 18 21 |
| mc11002040 | 7 8 10 | mc4002466 | 1 |
| mc11002045 | 21 | mc4002467 | 1 7 10 21 |
| mc11002050 | 21 | mc4002470 | 21 |
| mc11002051 | 6 7 8 11 21 | mc4002474 | 4 7 16 21 |
| mc11002052 | 7 10 13 15 21 | mc4002479 | 6 7 8 10 |
| mc11002053 | 4 | mc4002482 | 9 10 12 21 |
| mc11002055 | 4 7 10 12 21 | mc4002488 | 1 7 |
| mc11002057 | 4 7 9 21 | mc4002492 | 1 3 6 7 8 15 21 |
| mc11002059 | 7 | mc4002494 | 9 |
| mc11002060 | 3 7 21 | mc4002500 | 7 21 |
| mc11002062 | 21 | mc4002501 | 7 17 |
| mc11002064 | 7 15 21 | mc4002504 | 5 21 |
| mc11002066 | 5 7 10 21 | mc4002507 | 6 7 8 10 |
| mc11002068 | 10 12 13 15 21 | mc4002509 | 7 10 21 |
| mc11002069 | 3 4 7 17 21 | mc4002510 | 10 12 13 21 |
| mc11002075 | 17 21 | mc4002518 | 6 7 21 |
| mc11002077 | 7 17 21 | mc4002520 | 1 6 7 21 |
| mc11002079 | 7 17 21 | mc4002521 | 7 21 |
| mc11002080 | 21 | mc4002530 | 8 12 13 |
| mc11002094 | 21 | mc4002540 | 7 21 |
| mc11002124 | 21 | mc4002542 | 10 21 |
| mc11002129 | 17 21 | mc4002545 | 7 10 13 15 21 |
| mc11002130 | 7 17 18 19 20 21 22 23 24 | mc4002547 | 10 12 13 15 20 21 |
| mc11002138 | 5 | mc4002549 | 4 7 21 |
| mc11002139 | 10 21 | mc4002556 | 7 |
| mc11002144 | 6 7 21 | mc4002562 | 1 2 7 9 21 |
| mc11002146 | 7 21 | mc4002564 | 7 |
| mc11002148 | 1 7 21 | mc4002566 | 7 15 21 |
| mc11002150 | 1 4 13 16 | mc4002569 | 10 13 |
| mc11002151 | 6 7 11 | mc4002571 | 6 7 11 |
| mc11002155 | 3 7 | mc4002572 | 1 8 21 |
| mc11002156 | 3 4 7 21 | mc4002575 | 1 4 6 7 8 10 16 21 |
| mc11002158 | 7 9 10 17 21 | mc4002585 | 17 21 |
| mc11002161 | 10 13 21 | mc4002587 | 1 6 9 21 |
| mc11002164 | 4 7 10 21 | mc4002588 | 7 9 12 13 |
| mc11002168 | 4 21 | mc4002589 | 16 |
| mc11002169 | 6 9 21 | mc5000011 | 9 10 |
| mc11002170 | 1 11 21 | mc5000016 | 3 4 7 21 |
| mc11002171 | 1 21 | mc5000018 | 1 9 21 |
| mc11002172 | 1 7 | mc5000019 | 1 7 9 21 |
| mc11002173 | 4 7 9 21 | mc5000024 | 9 21 |
| mc11002174 | 7 21 | mc5000025 | 3 7 8 10 21 |
| mc11002177 | 7 17 21 | mc5000053 | 11 21 24 |
| mc11002180 | 7 10 11 12 13 15 21 | mc5000060 | 7 17 |
| mc11002182 | 4 7 21 | mc5000068 | 1 6 7 10 21 |
| mc11002184 | 9 10 21 | mc5000072 | 10 21 |
| mc11002185 | 21 | mc5000081 | 1 2 6 7 9 21 |
| mc11002187 | 21 | mc5000082 | 1 7 21 |
| mc11002189 | 7 12 21 | mc5000086 | 7 8 10 20 21 |
| mc11002192 | 1 7 12 21 | mc5000090 | 7 |
| mc11002196 | 5 | mc5000097 | 1 3 6 7 |
| mc11002199 | 5 7 21 | mc5000109 | 10 15 16 |
| mc11002200 | 13 21 | mc5000119 | 12 15 16 21 |
| mc11002201 | 7 | mc5000125 | 15 16 21 |
| mc11002203 | 3 4 7 11 15 21 | mc5000127 | 7 9 10 17 21 |
| mc11002204 | 7 | mc5000137 | 9 10 12 13 21 23 24 |
| mc11002211 | 4 7 12 15 16 21 | mc5000141 | 3 6 10 15 |
| mc11002213 | 4 7 16 21 | mc5000149 | 7 15 16 21 |
| mc11002215 | 10 20 21 | mc5000152 | 7 9 10 12 21 |
| mc11002216 | 1 6 8 10 | mc5000171 | 1 7 10 |
| mc11002217 | 1 7 10 21 | mc5000173 | 21 |
| mc11002219 | 6 7 | mc5000178 | 1 6 8 10 21 |
| mc11002221 | 10 21 | mc5000180 | 6 |
| mc11002222 | 10 12 21 | mc5000182 | 15 21 |
| mc11002227 | 21 | mc5000185 | 6 |
| mc11002228 | 4 6 7 8 21 23 24 | mc5000191 | 6 7 11 21 |
| mc11002231 | 10 | mc5000192 | 6 7 8 11 21 |
| mc11002234 | 4 7 17 21 | mc5000193 | 2 7 9 15 21 |
| mc11002236 | 9 21 | mc5000194 | 6 7 10 12 13 15 16 18 20 21 |
| mc11002237 | 8 10 | mc5000218 | 1 4 6 7 10 15 21 |
| mc11002241 | 15 16 19 21 | mc5000224 | 3 9 21 |
| mc11002243 | 7 10 12 13 21 23 | mc5000226 | 6 7 9 21 |
| mc11002253 | 7 10 16 20 21 24 | mc5000230 | 21 |
| mc11002255 | 9 10 12 13 21 | mc5000239 | 9 10 12 13 21 22 |
| mc11002258 | 4 5 7 21 | mc5000240 | 1 3 6 7 12 16 21 |
| mc11002261 | 7 17 21 | mc5000241 | 9 21 |
| mc11002264 | 13 22 23 24 | mc5000243 | 6 21 |
| mc11002266 | 1 | mc5000244 | 4 7 21 |
| mc11002269 | 7 | mc5000245 | 4 7 16 20 21 |
| mc11002270 | 4 7 19 20 21 | mc5000246 | 4 7 10 |
| mc11002275 | 7 10 12 17 21 | mc5000247 | 7 |
| mc11002278 | 1 6 7 10 | mc5000248 | 7 9 |
| mc11002291 | 9 21 | mc5000255 | 7 21 |
| mc11002293 | 7 10 15 16 21 | mc5000259 | 4 7 9 10 21 |
| mc11002294 | 1 6 7 9 21 | mc5000261 | 1 6 10 11 |
| mc11002301 | 7 17 21 22 | mc5000265 | 1 7 21 |
| mc11002310 | 6 7 10 12 13 21 23 | mc5000268 | 1 7 21 |
| mc11002316 | 1 21 | mc5000272 | 6 7 11 |
| mc11002321 | 1 7 9 12 21 22 | mc5000275 | 3 7 21 |
| mc11002331 | 2 7 9 | mc5000288 | 6 |
| mc11002337 | 21 | mc5000300 | 21 |
| mc11002343 | 21 | mc5000307 | 7 11 21 |
| mc11002344 | 3 4 7 | mc5000311 | 4 7 16 19 20 21 23 24 |
| mc11002345 | 1 4 21 | mc5000316 | 1 6 7 10 12 15 16 18 19 20 21 |
| mc11002346 | 6 7 8 11 21 | mc5000324 | 21 |
| mc11002351 | 7 10 21 | mc5000329 | 4 7 13 21 |
| mc11002352 | 7 9 21 | mc5000330 | 18 |
| mc11002354 | 7 12 13 21 | mc5000331 | 6 7 21 |
| mc11002355 | 4 6 7 11 15 21 | mc5000333 | 6 11 |
| mc11002358 | 7 10 12 21 | mc5000339 | 7 10 13 15 21 |
| mc11002360 | 3 7 21 | mc5000342 | 1 |
| mc11002361 | 3 4 7 8 21 | mc5000345 | 1 2 21 |
| mc11002366 | 6 7 9 21 | mc5000346 | 1 2 4 7 21 |
| mc11002374 | 1 7 9 10 21 | mc5000348 | 1 7 |
| mc11002375 | 6 7 8 11 21 | mc5000355 | 7 9 21 |
| mc11002379 | 9 10 12 13 21 23 | mc5000357 | 3 7 17 21 |
| mc11002380 | 9 10 12 21 24 | mc5000358 | 7 12 16 21 22 |
| mc11002384 | 9 10 12 21 | mc5000360 | 3 7 17 21 |
| mc11002389 | 7 21 | mc5000367 | 7 9 12 21 |
| mc11002397 | 3 7 10 15 16 18 19 21 | mc5000368 | 1 |
| mc11002403 | 7 8 10 12 13 | mc5000371 | 4 7 9 21 |
| mc11002407 | 6 | mc5000374 | 7 9 |
| mc11002413 | 4 10 21 | mc5000375 | 15 |
| mc11002420 | 1 7 9 | mc5000377 | 7 9 |
| mc11002421 | 7 | mc5000378 | 6 7 11 |
| mc11002422 | 7 9 | mc5000379 | 6 7 15 16 17 21 |
| mc11002433 | 7 9 21 | mc5000380 | 10 21 |
| mc11002434 | 9 12 21 | mc5000382 | 4 21 |
| mc11002456 | 4 7 15 16 18 19 20 21 | mc5000383 | 5 8 21 |
| mc11002466 | 9 | mc5000384 | 7 9 21 |
| mc11002474 | 10 15 21 22 23 24 | mc5000386 | 1 3 6 7 10 21 |
| mc11002476 | 6 9 21 | mc5000388 | 1 6 7 10 |
| mc11002481 | 7 10 15 21 | mc5000390 | 16 21 |
| mc11002484 | 6 7 9 12 21 | mc5000394 | 1 6 7 8 9 21 |
| mc11002497 | 5 7 21 | mc5000402 | 1 7 10 |
| mc11002501 | 10 | mc5000404 | 9 21 |
| mc11002502 | 10 13 21 | mc5000407 | 1 2 |
| mc11002504 | 10 | mc5000412 | 4 7 21 |
| mc11002510 | 10 21 | mc5000421 | 1 |
| mc11002512 | 10 | mc5000429 | 7 12 13 16 21 |
| mc11002513 | 7 10 18 19 | mc5000430 | 1 7 21 |
| mc11002514 | 7 9 10 | mc5000438 | 4 7 21 |
| mc11002515 | 7 10 12 21 | mc5000440 | 3 6 7 9 11 19 21 |
| mc11002518 | 7 9 10 12 21 | mc5000442 | 1 6 7 10 15 18 20 21 |
| mc11002519 | 2 7 9 21 | mc5000443 | 7 10 12 21 |
| mc11002529 | 5 | mc5000446 | 4 21 |
| mc11002530 | 2 7 9 21 | mc5000451 | 3 7 21 |
| mc11002535 | 1 7 | mc5000455 | 4 7 21 |
| mc11002539 | 6 7 9 21 | mc5000459 | 4 7 21 |
| mc11002540 | 5 7 21 | mc5000464 | 7 10 21 |
| mc11002542 | 7 9 21 | mc5000465 | 10 |
| mc11002544 | 7 8 10 | mc5000466 | 6 7 21 |
| mc11002549 | 4 7 | mc5000467 | 9 |
| mc11002554 | 3 7 21 | mc5000468 | 4 5 21 |
| mc11002555 | 4 7 21 | mc5000470 | 7 9 15 21 |
| mc11002557 | 1 7 21 | mc5000472 | 7 8 9 10 |
| mc11002559 | 2 6 7 9 21 | mc5000474 | 6 21 |
| mc11002561 | 7 | mc5000476 | 7 8 9 |
| mc11002565 | 5 21 | mc5000479 | 7 10 13 20 21 |
| mc11002568 | 1 2 9 21 | mc5000482 | 4 7 21 |
| mc11002570 | 1 6 7 | mc5000487 | 6 21 |
| mc11002571 | 7 19 21 | mc5000490 | 1 21 |
| mc11002572 | 7 10 21 | mc5000492 | 6 7 15 21 |
| mc11002574 | 10 16 | mc5000495 | 7 17 |
| mc11002575 | 6 7 9 19 21 | mc5000496 | 7 17 21 |
| mc11002585 | 1 7 15 18 21 | mc5000497 | 7 9 21 |
| mc11002587 | 1 6 | mc5000502 | 6 9 21 |
| mc11002589 | 7 9 13 | mc5000509 | 7 9 17 18 |
| mc11002592 | 1 6 21 | mc5000510 | 7 |
| mc11002594 | 1 6 21 | mc5000511 | 1 18 21 |
| mc11002596 | 10 15 16 21 | mc5000513 | 13 16 20 |
| mc11002597 | 1 | mc5000516 | 7 10 |
| mc11002598 | 4 7 21 | mc5000520 | 16 |
| mc11002606 | 9 | mc5000521 | 16 21 |
| mc11002621 | 21 | mc5000525 | 4 7 16 19 20 21 |
| mc11002623 | 9 17 21 | mc5000528 | 7 9 21 |
| mc11002624 | 1 7 21 | mc5000530 | 7 10 21 |
| mc11002625 | 1 21 | mc5000532 | 7 21 |
| mc11002626 | 7 8 15 16 17 21 | mc5000540 | 7 13 21 |
| mc11002630 | 1 6 7 10 15 | mc5000545 | 7 10 |
| mc11002639 | 1 7 8 10 15 18 21 | mc5000556 | 1 6 21 |
| mc11002642 | 6 7 10 | mc5000568 | 4 7 20 21 |
| mc11002645 | 10 21 | mc5000582 | 7 21 |
| mc11002660 | 3 4 7 17 21 | mc5000586 | 9 21 |
| mc11002661 | 3 4 7 17 21 | mc5000588 | 6 |
| mc11002662 | 3 4 7 17 21 | mc5000590 | 13 15 16 18 21 |
| mc11002667 | 1 2 11 21 | mc5000592 | 1 10 21 |
| mc11002668 | 3 4 5 7 21 | mc5000597 | 4 7 21 |
| mc11002670 | 7 10 15 | mc5000598 | 21 |
| mc11002672 | 9 | mc5000614 | 1 7 |
| mc11002675 | 1 | mc5000616 | 6 7 21 |
| mc11002677 | 7 21 | mc5000617 | 4 7 21 |
| mc11002685 | 7 10 | mc5000622 | 4 21 |
| mc11002688 | 15 16 | mc5000666 | 2 4 7 8 21 |
| mc11002704 | 6 7 11 21 | mc5000679 | 1 3 4 7 21 |
| mc11002706 | 1 7 10 12 13 | mc5000681 | 7 11 12 21 |
| mc11002710 | 6 7 9 10 21 | mc5000689 | 7 |
| mc11002711 | 5 21 | mc5000693 | 3 6 7 21 |
| mc11002712 | 7 9 21 23 | mc5000703 | 4 7 21 |
| mc11002714 | 10 | mc5000704 | 10 12 13 16 20 21 24 |
| mc11002717 | 6 8 9 13 21 | mc5000724 | 7 12 21 |
| mc11002718 | 7 8 10 12 20 21 | mc5000762 | 1 2 9 |
| mc11002719 | 4 6 7 9 11 21 | mc5000765 | 8 21 |
| mc11002720 | 3 6 7 21 | mc5000772 | 4 7 21 |
| mc11002721 | 8 10 21 | mc5000775 | 10 13 |
| mc11002722 | 1 | mc5000781 | 21 |
| mc11002723 | 3 4 7 21 | mc5000782 | 3 7 21 |
| mc11002724 | 4 7 13 16 21 | mc5000783 | 1 2 |
| mc11002730 | 7 10 21 | mc5000785 | 1 7 21 |
| mc11002731 | 1 13 21 | mc5000786 | 1 2 9 16 |
| mc11002735 | 1 15 16 21 | mc5000788 | 6 7 |
| mc11002736 | 6 7 8 21 | mc5000804 | 7 10 |
| mc11002738 | 1 7 18 | mc5000808 | 3 6 7 9 11 12 13 16 21 22 |
| mc11002742 | 9 | mc5000819 | 4 7 16 18 21 |
| mc11002746 | 13 15 21 22 | mc5000824 | 21 |
| mc11002747 | 21 | mc5000831 | 1 7 9 |
| mc11002748 | 21 22 | mc5000852 | 2 7 9 21 |
| mc11002753 | 10 | mc5000853 | 9 10 12 21 23 24 |
| mc11002760 | 7 9 10 | mc5000864 | 6 10 |
| mc11002762 | 1 | mc5000879 | 1 6 7 10 |
| mc11002764 | 21 | mc5000918 | 6 7 10 21 |
| mc11002765 | 1 | mc5000925 | 9 21 |
| mc11002770 | 3 21 | mc5000927 | 4 7 16 20 21 |
| mc11002774 | 7 | mc5000928 | 6 7 8 10 21 |
| mc11002776 | 1 6 7 10 | mc5000931 | 1 6 7 8 10 16 18 21 |
| mc11002777 | 4 7 21 | mc5000935 | 6 7 10 18 21 |
| mc11002782 | 9 10 21 | mc5000939 | 21 |
| mc11002783 | 8 | mc5000940 | 4 7 10 12 21 |
| mc11002784 | 2 7 9 13 17 20 21 | mc5000941 | 12 |
| mc11002790 | 6 7 | mc5000942 | 10 13 21 |
| mc11002793 | 3 4 7 21 | mc5000958 | 1 |
| mc11002796 | 7 21 | mc5000961 | 1 21 |
| mc11002799 | 16 21 | mc5000962 | 1 |
| mc11002800 | 7 9 21 | mc5000966 | 4 7 16 19 20 21 |
| mc11002802 | 4 7 10 12 13 15 20 21 | mc5000968 | 4 7 21 |
| mc11002803 | 1 21 | mc5000969 | 21 |
| mc11002805 | 1 6 7 11 21 | mc5000970 | 4 7 21 |
| mc11002807 | 9 21 | mc5000971 | 1 7 15 21 |
| mc11002811 | 1 7 9 21 | mc5001016 | 1 6 7 10 |
| mc12000008 | 6 7 22 24 | mc5001024 | 7 21 |
| mc12000010 | 3 21 | mc5001025 | 7 10 13 19 |
| mc12000013 | 10 21 | mc5001026 | 6 7 8 |
| mc12000018 | 1 7 10 13 21 | mc5001027 | 10 |
| mc12000022 | 4 7 10 13 21 | mc5001029 | 6 |
| mc12000024 | 7 9 | mc5001033 | 6 |
| mc12000029 | 4 7 21 | mc5001035 | 6 |
| mc12000030 | 6 10 13 21 23 24 | mc5001045 | 1 11 21 |
| mc12000074 | 7 12 15 16 18 21 | mc5001049 | 1 11 21 |
| mc12000077 | 7 10 18 | mc5001054 | 1 6 21 |
| mc12000081 | 3 7 12 15 21 | mc5001055 | 1 6 |
| mc12000082 | 9 | mc5001056 | 1 6 7 |
| mc12000084 | 7 17 21 | mc5001058 | 9 21 |
| mc12000088 | 9 | mc5001060 | 9 21 |
| mc12000089 | 7 17 21 | mc5001062 | 9 21 |
| mc12000105 | 1 7 21 | mc5001066 | 21 |
| mc12000106 | 1 | mc5001076 | 11 21 |
| mc12000113 | 7 9 | mc5001078 | 21 |
| mc12000114 | 16 | mc5001080 | 7 16 17 19 20 21 |
| mc12000135 | 3 4 6 7 10 15 18 21 | mc5001081 | 20 21 |
| mc12000139 | 1 2 3 4 5 6 7 16 | mc5001082 | 13 21 |
| mc12000163 | 1 6 7 8 | mc5001083 | 3 16 21 |
| mc12000173 | 1 19 21 | mc5001086 | 7 16 19 |
| mc12000175 | 2 7 9 10 | mc5001089 | 1 7 21 |
| mc12000176 | 2 9 | mc5001095 | 9 12 21 |
| mc12000178 | 3 4 7 21 | mc5001100 | 7 10 21 |
| mc12000179 | 1 6 7 10 | mc5001102 | 6 10 12 |
| mc12000184 | 7 9 | mc5001113 | 16 |
| mc12000187 | 2 7 9 | mc5001114 | 7 9 13 21 |
| mc12000190 | 1 | mc5001115 | 7 18 21 24 |
| mc12000200 | 4 7 | mc5001122 | 12 13 15 |
| mc12000202 | 4 7 | mc5001123 | 10 13 15 21 |
| mc12000207 | 1 3 7 | mc5001124 | 12 13 15 |
| mc12000213 | 4 7 8 21 | mc5001125 | 12 13 15 |
| mc12000229 | 6 7 21 | mc5001126 | 10 12 13 15 19 21 |
| mc12000231 | 1 7 | mc5001128 | 3 10 13 15 21 |
| mc12000261 | 1 | mc5001133 | 10 15 21 |
| mc12000263 | 5 21 | mc5001135 | 3 15 16 20 21 |
| mc12000264 | 1 7 21 | mc5001136 | 15 21 |
| mc12000265 | 1 7 21 | mc5001149 | 6 7 10 21 |
| mc12000281 | 4 7 15 16 21 | mc5001154 | 7 9 10 21 |
| mc12000284 | 1 7 11 21 24 | mc5001156 | 7 9 21 |
| mc12000286 | 7 21 | mc5001158 | 1 6 7 10 12 13 |
| mc12000295 | 1 6 21 | mc5001161 | 12 13 15 21 |
| mc12000305 | 7 12 16 17 21 | mc5001162 | 12 13 15 21 |
| mc12000306 | 1 2 7 9 21 | mc5001163 | 10 12 13 15 21 |
| mc12000309 | 9 21 24 | mc5001164 | 1 6 21 |
| mc12000310 | 6 7 9 12 | mc5001165 | 9 21 |
| mc12000311 | 9 13 15 | mc5001166 | 10 12 21 |
| mc12000312 | 7 9 10 13 21 22 | mc5001181 | 3 8 |
| mc12000316 | 10 | mc5001183 | 3 8 |
| mc12000321 | 6 7 10 21 | mc5001192 | 10 12 13 15 20 21 |
| mc12000325 | 1 6 7 10 15 21 | mc5001196 | 5 7 21 |
| mc12000336 | 1 21 | mc5001204 | 7 8 |
| mc12000350 | 4 7 8 16 | mc5001210 | 1 6 7 8 16 21 |
| mc12000351 | 4 7 8 16 | mc5001211 | 10 |
| mc12000358 | 1 2 6 7 16 | mc5001216 | 1 7 21 |
| mc12000365 | 3 4 7 10 12 21 | mc5001225 | 3 7 10 15 18 21 |
| mc12000382 | 7 10 16 20 21 | mc5001232 | 10 15 16 20 21 |
| mc12000385 | 1 | mc5001233 | 1 6 7 8 10 |
| mc12000390 | 4 7 16 21 | mc5001237 | 3 |
| mc12000391 | 1 | mc5001251 | 4 7 11 17 21 |
| mc12000394 | 1 7 9 21 | mc5001252 | 1 7 21 |
| mc12000397 | 4 7 12 13 16 19 21 | mc5001253 | 1 |
| mc12000405 | 7 17 21 | mc5001267 | 6 7 8 21 |
| mc12000406 | 16 19 21 | mc5001270 | 1 7 21 |
| mc12000415 | 6 9 21 | mc5001290 | 4 7 15 16 18 21 |
| mc12000418 | 21 | mc5001292 | 1 21 |
| mc12000429 | 6 7 11 21 | mc5001295 | 1 21 |
| mc12000462 | 4 7 20 21 22 24 | mc5001303 | 8 |
| mc12000467 | 1 6 7 8 10 | mc5001312 | 1 6 7 8 10 21 |
| mc12000484 | 13 21 | mc5001317 | 1 4 7 21 |
| mc12000485 | 7 10 21 | mc5001319 | 7 |
| mc12000486 | 6 9 17 21 | mc5001322 | 1 |
| mc12000494 | 10 21 | mc5001324 | 1 7 |
| mc12000524 | 7 15 21 | mc5001326 | 7 21 |
| mc12000535 | 21 | mc5001328 | 7 20 21 |
| mc12000536 | 9 | mc5001329 | 7 17 20 21 |
| mc12000538 | 7 21 | mc5001332 | 7 10 12 20 21 |
| mc12000544 | 7 | mc5001333 | 16 20 21 |
| mc12000548 | 6 7 8 11 13 21 | mc5001336 | 7 10 12 13 15 16 19 20 21 |
| mc12000551 | 7 12 13 15 21 | mc5001338 | 7 9 10 12 21 |
| mc12000554 | 4 7 8 21 | mc5001348 | 1 7 9 |
| mc12000555 | 4 7 8 21 | mc5001353 | 7 13 |
| mc12000563 | 21 | mc5001367 | 4 7 21 |
| mc12000569 | 4 7 21 | mc5001391 | 3 6 7 8 21 |
| mc12000576 | 9 21 | mc5001393 | 10 21 |
| mc12000587 | 4 7 19 20 21 | mc5001395 | 3 7 21 |
| mc12000597 | 10 21 | mc5001403 | 7 10 15 |
| mc12000603 | 4 6 7 21 | mc5001405 | 4 7 13 20 21 |
| mc12000605 | 4 7 21 | mc5001407 | 7 11 15 16 18 |
| mc12000624 | 7 21 | mc5001410 | 5 7 21 |
| mc12000649 | 6 7 21 | mc5001415 | 4 7 21 |
| mc12000678 | 5 7 15 21 | mc5001419 | 4 7 21 |
| mc12000682 | 5 7 21 | mc5001423 | 2 7 9 21 |
| mc12000683 | 1 6 21 | mc5001425 | 7 |
| mc12000686 | 1 3 7 21 | mc5001428 | 7 9 |
| mc12000691 | 21 | mc5001431 | 1 |
| mc12000699 | 21 | mc5001432 | 1 2 11 21 |
| mc12000700 | 2 9 21 | mc5001433 | 9 |
| mc12000701 | 1 3 6 7 10 21 | mc5001435 | 7 10 15 21 |
| mc12000706 | 7 13 21 | mc5001437 | 9 21 |
| mc12000707 | 7 10 | mc5001462 | 3 6 7 21 |
| mc12000709 | 7 21 | mc5001463 | 7 15 16 21 |
| mc12000712 | 1 2 7 | mc5001466 | 17 21 |
| mc12000713 | 6 7 9 21 | mc5001467 | 1 3 7 11 21 |
| mc12000715 | 2 9 21 | mc5001468 | 9 10 12 |
| mc12000721 | 7 21 | mc5001475 | 21 |
| mc12000724 | 7 9 17 | mc5001477 | 3 4 7 |
| mc12000725 | 6 7 8 11 13 21 | mc5001478 | 3 4 7 21 |
| mc12000732 | 21 | mc5001479 | 1 4 7 8 21 |
| mc12000740 | 7 10 17 | mc5001480 | 1 6 7 10 16 21 |
| mc12000743 | 10 | mc5001482 | 6 21 |
| mc12000749 | 21 | mc5001483 | 1 3 6 7 8 10 11 21 |
| mc12000759 | 1 | mc5001512 | 6 21 |
| mc12000767 | 4 7 16 21 | mc5001513 | 7 20 21 |
| mc12000768 | 3 4 6 7 21 | mc5001514 | 6 7 17 18 21 |
| mc12000770 | 1 | mc5001545 | 10 12 21 |
| mc12000780 | 1 6 7 10 | mc5001548 | 4 7 21 |
| mc12000785 | 4 7 21 | mc5001549 | 6 21 |
| mc12000788 | 4 7 8 21 | mc5001551 | 7 12 |
| mc12000799 | 7 10 15 17 | mc5001559 | 1 3 7 21 |
| mc12000803 | 10 21 | mc5001566 | 6 7 |
| mc12000808 | 5 7 21 | mc5001567 | 1 21 |
| mc12000811 | 1 15 21 | mc5001568 | 1 7 21 |
| mc12000818 | 1 | mc5001574 | 7 9 10 11 12 |
| mc12000826 | 8 | mc5001575 | 9 |
| mc12000834 | 4 7 9 10 | mc5001579 | 1 7 8 10 |
| mc12000835 | 1 6 7 15 18 | mc5001586 | 1 6 7 13 |
| mc12000837 | 4 7 21 | mc5001589 | 4 7 21 |
| mc12000840 | 1 21 | mc5001597 | 1 2 7 21 |
| mc12000846 | 1 6 7 9 16 17 21 | mc5001599 | 6 9 |
| mc12000850 | 1 2 7 9 | mc5001603 | 7 |
| mc12000851 | 5 7 21 | mc5001604 | 1 21 |
| mc12000852 | 5 7 10 21 | mc5001609 | 2 7 9 21 24 |
| mc12000857 | 1 6 21 | mc5001610 | 5 7 21 |
| mc12000859 | 1 21 | mc5001611 | 1 21 24 |
| mc12000860 | 7 9 21 | mc5001615 | 6 7 10 12 17 21 |
| mc12000861 | 1 21 | mc5001622 | 7 9 10 |
| mc12000864 | 1 3 7 11 21 | mc5001625 | 1 3 6 7 10 15 16 |
| mc12000875 | 1 4 7 21 | mc5001627 | 1 2 6 10 11 21 |
| mc12000879 | 4 7 8 21 | mc5001633 | 1 6 7 11 |
| mc12000896 | 4 7 21 | mc5001643 | 3 4 7 8 17 |
| mc12000897 | 7 9 21 | mc5001649 | 10 |
| mc12000898 | 7 21 | mc5001657 | 7 10 12 21 22 |
| mc12000905 | 6 7 21 24 | mc5001658 | 6 |
| mc12000907 | 6 7 9 21 | mc5001660 | 7 12 21 |
| mc12000908 | 6 7 16 24 | mc5001661 | 6 |
| mc12000909 | 4 7 8 10 21 | mc5001662 | 15 |
| mc12000930 | 7 8 10 21 | mc5001684 | 4 7 16 21 |
| mc12000937 | 7 21 | mc5001687 | 4 7 19 20 21 |
| mc12000941 | 4 7 | mc5001690 | 7 21 |
| mc12000942 | 6 7 8 10 15 21 | mc5001696 | 1 |
| mc12000944 | 7 16 20 21 | mc5001697 | 1 2 |
| mc12000946 | 3 5 7 21 | mc5001700 | 9 |
| mc12000948 | 1 21 | mc5001703 | 3 4 7 21 |
| mc12000964 | 7 | mc5001705 | 7 8 10 |
| mc12000965 | 4 7 21 | mc5001710 | 1 7 11 13 21 |
| mc12000967 | 1 7 9 | mc5001711 | 1 7 11 13 |
| mc12000968 | 1 7 21 | mc5001718 | 7 11 13 |
| mc12000972 | 1 7 9 21 | mc5001723 | 7 8 9 21 |
| mc12000974 | 4 7 16 18 20 21 | mc5001728 | 5 7 21 |
| mc12000975 | 1 7 9 21 | mc5001735 | 21 |
| mc12000978 | 21 | mc5001737 | 6 7 12 16 21 22 24 |
| mc12000980 | 1 7 | mc5001739 | 1 6 7 10 21 |
| mc12000983 | 21 | mc5001742 | 7 9 21 |
| mc12000986 | 1 2 21 | mc5001744 | 6 7 9 10 21 |
| mc12000988 | 3 15 16 20 21 | mc5001745 | 21 22 23 24 |
| mc12000989 | 5 21 | mc5001746 | 7 10 15 16 19 |
| mc12000994 | 1 3 6 7 | mc5001750 | 4 7 21 |
| mc12000995 | 7 9 21 | mc5001760 | 7 |
| mc12000997 | 4 7 8 21 | mc5001762 | 9 |
| mc12001001 | 4 7 21 | mc5001765 | 6 7 12 17 21 |
| mc12001002 | 9 10 | mc5001766 | 3 6 7 21 |
| mc12001003 | 1 21 | mc5001771 | 7 9 10 16 21 |
| mc12001006 | 3 7 10 15 16 21 | mc5001773 | 7 9 10 21 |
| mc12001017 | 3 | mc5001774 | 6 7 10 13 |
| mc12001023 | 6 7 21 | mc5001776 | 3 6 7 21 |
| mc12001029 | 7 9 13 | mc5001777 | 6 7 15 21 |
| mc12001030 | 1 6 7 8 21 | mc5001785 | 7 9 10 21 |
| mc12001031 | 1 11 21 24 | mc5001787 | 1 7 |
| mc12001036 | 6 7 8 9 11 | mc5001788 | 6 7 8 11 21 |
| mc12001038 | 1 21 | mc5001795 | 1 6 21 |
| mc12001039 | 4 7 21 | mc5001796 | 7 15 21 |
| mc12001047 | 1 6 | mc5001797 | 6 9 17 21 |
| mc12001071 | 1 7 21 | mc5001798 | 7 21 |
| mc12001079 | 10 12 13 15 21 | mc5001799 | 7 |
| mc12001081 | 7 9 21 | mc5001803 | 7 10 |
| mc12001084 | 10 21 | mc5001804 | 10 |
| mc12001127 | 1 11 21 | mc5001805 | 5 |
| mc12001128 | 10 21 | mc5001807 | 7 9 21 |
| mc12001131 | 9 | mc5001809 | 1 7 9 21 |
| mc12001133 | 1 6 7 8 10 21 | mc5001811 | 7 21 |
| mc12001152 | 1 3 11 21 | mc5001819 | 1 3 |
| mc12001156 | 6 7 8 11 21 | mc5001826 | 7 |
| mc12001158 | 7 8 10 | mc5001829 | 9 21 |
| mc12001164 | 1 5 6 7 10 21 | mc5001830 | 3 4 7 |
| mc12001166 | 7 21 | mc5001831 | 5 8 |
| mc12001167 | 10 13 21 | mc5001832 | 3 4 7 21 |
| mc12001181 | 7 12 21 | mc5001834 | 9 21 |
| mc12001185 | 4 21 | mc5001839 | 9 10 |
| mc12001186 | 4 7 21 | mc5001840 | 3 4 7 17 21 |
| mc12001188 | 9 12 21 | mc5001843 | 10 12 21 |
| mc12001190 | 8 10 | mc5001844 | 6 21 |
| mc12001191 | 6 15 20 21 | mc5001846 | 3 4 7 |
| mc12001192 | 21 | mc5001848 | 1 |
| mc12001193 | 7 21 | mc5001872 | 7 9 |
| mc12001194 | 1 7 | mc5001887 | 7 16 21 |
| mc12001197 | 7 15 21 | mc5001892 | 7 9 16 19 21 |
| mc12001200 | 3 6 7 | mc5001893 | 3 6 7 9 10 21 |
| mc12001211 | 4 7 10 | mc5001903 | 4 7 21 |
| mc12001213 | 6 | mc5001907 | 6 7 21 |
| mc12001214 | 3 4 7 | mc5001913 | 5 21 |
| mc12001217 | 21 | mc5001915 | 1 7 |
| mc12001219 | 8 | mc5001924 | 1 2 11 21 |
| mc12001220 | 7 9 21 | mc5001925 | 1 21 |
| mc12001223 | 8 21 | mc5001926 | 10 21 |
| mc12001234 | 8 21 | mc5001929 | 8 9 10 13 21 |
| mc12001236 | 8 21 | mc5001935 | 7 9 |
| mc12001246 | 8 21 | mc5001939 | 7 |
| mc12001251 | 4 7 16 21 | mc5001941 | 1 7 21 |
| mc12001256 | 7 21 | mc5001976 | 7 |
| mc12001261 | 21 | mc5001978 | 1 2 3 9 10 11 12 13 21 |
| mc12001266 | 22 23 | mc5001979 | 4 7 21 |
| mc12001269 | 21 | mc5001987 | 21 |
| mc12001278 | 10 | mc5001989 | 1 6 7 10 21 |
| mc12001279 | 10 | mc5001990 | 7 16 17 19 21 |
| mc12001288 | 4 7 21 | mc5001994 | 7 17 21 |
| mc12001291 | 1 6 7 21 | mc5001995 | 1 21 |
| mc12001319 | 4 7 15 16 21 | mc5001998 | 7 17 |
| mc12001328 | 21 | mc5002003 | 1 21 |
| mc12001329 | 3 4 7 8 | mc5002004 | 6 7 11 |
| mc12001334 | 1 2 7 9 21 | mc5002007 | 4 7 21 |
| mc12001340 | 7 | mc5002008 | 21 |
| mc12001346 | 4 7 21 | mc5002010 | 3 4 7 9 21 |
| mc12001347 | 9 21 | mc5002015 | 21 |
| mc12001361 | 15 21 | mc5002016 | 9 21 |
| mc12001362 | 16 | mc5002020 | 7 10 12 13 15 21 |
| mc12001383 | 1 6 7 8 10 21 | mc5002024 | 1 7 9 11 21 |
| mc12001384 | 2 7 9 21 | mc5002026 | 1 |
| mc12001386 | 6 7 11 21 | mc5002027 | 1 2 7 9 21 |
| mc12001394 | 1 | mc5002031 | 6 11 21 23 |
| mc12001398 | 21 | mc5002035 | 7 10 16 21 |
| mc12001400 | 7 10 15 | mc5002036 | 6 7 10 21 |
| mc12001402 | 6 13 16 21 | mc5002037 | 21 |
| mc12001403 | 1 6 7 10 | mc5002040 | 4 7 21 |
| mc12001408 | 15 16 20 | mc5002043 | 5 7 21 |
| mc12001411 | 5 7 | mc5002044 | 1 7 8 21 |
| mc12001413 | 1 6 7 10 | mc5002046 | 7 9 10 13 15 17 21 22 |
| mc12001416 | 1 21 | mc5002048 | 4 7 16 20 21 |
| mc12001422 | 3 7 11 21 | mc5002052 | 7 |
| mc12001424 | 11 15 21 | mc5002053 | 7 9 21 |
| mc12001428 | 21 | mc5002056 | 21 |
| mc12001447 | 1 7 23 | mc5002057 | 7 17 21 |
| mc12001448 | 7 15 21 | mc5002059 | 1 6 7 21 |
| mc12001459 | 3 10 21 | mc5002063 | 6 7 8 20 21 |
| mc12001462 | 7 10 16 20 21 | mc5002065 | 12 21 |
| mc12001463 | 1 7 | mc5002067 | 11 21 |
| mc12001464 | 5 | mc5002068 | 2 4 7 9 10 21 |
| mc12001469 | 4 7 10 21 | mc5002069 | 9 21 |
| mc12001471 | 4 7 | mc5002071 | 1 6 7 10 |
| mc12001483 | 6 7 21 24 | mc5002073 | 7 10 11 18 21 |
| mc12001579 | 10 21 | mc5002074 | 4 7 21 |
| mc12001585 | 7 16 | mc5002076 | 7 10 |
| mc12001592 | 1 6 8 10 21 | mc5002079 | 6 9 10 12 21 22 |
| mc12001602 | 8 10 21 | mc5002080 | 7 19 20 |
| mc12001604 | 2 6 7 9 12 16 17 21 | mc5002082 | 6 7 21 |
| mc12001605 | 4 7 8 13 21 | mc5002084 | 21 23 |
| mc12001628 | 4 7 10 21 | mc5002086 | 7 10 21 |
| mc12001629 | 9 21 | mc5002087 | 7 8 |
| mc12001630 | 2 7 9 10 21 | mc5002089 | 21 |
| mc12001632 | 1 6 7 10 | mc5002091 | 4 7 21 |
| mc12001635 | 4 7 10 12 16 20 21 | mc5002094 | 1 21 |
| mc12001637 | 7 | mc5002097 | 7 10 13 |
| mc12001638 | 4 21 | mc5002101 | 9 10 21 |
| mc12001639 | 7 | mc5002102 | 7 10 13 21 |
| mc12001641 | 21 | mc5002105 | 4 7 15 16 21 |
| mc12001646 | 8 | mc5002106 | 4 6 7 8 21 |
| mc12001647 | 21 | mc5002107 | 3 7 21 |
| mc13000012 | 7 | mc5002108 | 6 9 17 21 |
| mc13000014 | 8 10 21 | mc5002109 | 4 7 21 |
| mc13000016 | 7 10 | mc5002117 | 21 |
| mc13000019 | 1 | mc5002118 | 3 7 21 24 |
| mc13000027 | 1 7 21 | mc5002127 | 4 7 21 |
| mc13000039 | 4 7 10 15 21 | mc5002130 | 7 |
| mc13000043 | 6 | mc5002134 | 3 6 15 17 20 21 |
| mc13000044 | 1 2 7 21 | mc5002140 | 7 21 |
| mc13000059 | 1 4 7 21 | mc5002142 | 7 21 |
| mc13000064 | 1 | mc5002147 | 10 |
| mc13000066 | 5 7 10 21 | mc5002156 | 4 7 21 |
| mc13000069 | 7 | mc5002162 | 1 |
| mc13000076 | 4 6 7 21 | mc5002170 | 3 7 21 |
| mc13000077 | 7 10 12 13 21 | mc5002173 | 3 |
| mc13000101 | 7 | mc5002174 | 1 3 21 |
| mc13000108 | 7 12 21 | mc5002176 | 5 7 |
| mc13000110 | 9 10 13 21 | mc5002179 | 1 21 |
| mc13000112 | 7 10 21 | mc5002182 | 1 16 21 |
| mc13000116 | 6 7 11 16 17 | mc5002187 | 1 7 10 15 21 |
| mc13000119 | 1 | mc5002189 | 7 10 13 15 |
| mc13000126 | 5 21 | mc5002204 | 7 12 |
| mc13000138 | 10 15 21 | mc5002212 | 4 7 21 |
| mc13000161 | 4 7 21 | mc5002216 | 2 7 9 10 21 |
| mc13000163 | 7 9 21 | mc5002220 | 10 15 |
| mc13000165 | 7 9 13 21 | mc5002223 | 9 |
| mc13000173 | 7 12 21 | mc5002231 | 7 |
| mc13000174 | 4 7 10 15 16 21 | mc5002233 | 6 7 15 21 |
| mc13000175 | 1 2 7 9 11 15 16 | mc5002237 | 4 7 21 |
| mc13000178 | 10 | mc5002248 | 3 7 10 17 |
| mc13000195 | 1 13 | mc5002262 | 4 7 |
| mc13000198 | 10 13 21 | mc5002264 | 3 7 11 |
| mc13000200 | 4 6 7 21 | mc5002267 | 7 |
| mc13000207 | 4 7 21 | mc5002270 | 4 7 9 10 17 21 |
| mc13000208 | 4 7 21 | mc5002274 | 1 10 |
| mc13000223 | 10 12 13 | mc5002279 | 7 21 |
| mc13000226 | 7 17 21 | mc5002284 | 6 7 |
| mc13000234 | 3 7 19 21 | mc5002291 | 6 7 12 17 21 |
| mc13000250 | 6 21 | mc5002295 | 4 7 21 |
| mc13000311 | 4 7 21 | mc5002296 | 2 7 9 21 |
| mc13000314 | 21 | mc5002298 | 4 7 21 |
| mc13000333 | 3 7 15 16 17 21 | mc5002304 | 1 9 11 21 24 |
| mc13000335 | 10 13 21 | mc5002316 | 3 4 7 15 16 17 21 |
| mc13000347 | 9 | mc5002318 | 1 3 4 6 7 21 |
| mc13000348 | 9 | mc5002319 | 7 21 |
| mc13000349 | 7 9 21 | mc5002323 | 6 |
| mc13000356 | 7 | mc5002324 | 5 7 21 |
| mc13000366 | 1 2 7 9 21 | mc5002326 | 7 21 |
| mc13000370 | 3 15 21 | mc5002327 | 5 |
| mc13000373 | 1 21 | mc5002328 | 6 7 10 21 |
| mc13000374 | 1 10 21 | mc5002329 | 1 4 7 21 |
| mc13000378 | 1 9 10 12 21 | mc5002332 | 4 16 20 21 24 |
| mc13000380 | 10 | mc5002335 | 4 7 16 20 21 24 |
| mc13000396 | 6 10 13 21 | mc5002336 | 4 7 15 16 21 |
| mc13000397 | 10 21 | mc5002339 | 1 6 7 10 18 19 21 |
| mc13000401 | 10 21 | mc5002346 | 1 6 7 10 12 15 16 20 21 |
| mc13000402 | 10 21 | mc5002359 | 9 10 |
| mc13000403 | 10 21 | mc5002369 | 1 3 4 7 8 9 17 21 |
| mc13000404 | 10 21 | mc5002375 | 1 7 8 13 21 |
| mc13000406 | 10 21 | mc5002381 | 6 7 11 12 |
| mc13000410 | 10 21 | mc5002388 | 9 10 16 20 21 |
| mc13000413 | 10 21 | mc5002395 | 3 7 |
| mc13000414 | 10 21 24 | mc5002407 | 1 2 11 15 21 |
| mc13000415 | 10 21 | mc5002410 | 8 |
| mc13000416 | 10 21 | mc5002429 | 5 7 |
| mc13000417 | 10 21 | mc5002432 | 6 8 |
| mc13000422 | 10 21 | mc5002433 | 1 6 7 21 |
| mc13000437 | 1 4 7 21 | mc5002451 | 7 |
| mc13000454 | 7 8 10 13 21 | mc5002456 | 12 20 |
| mc13000456 | 2 7 9 21 | mc5002457 | 10 12 21 |
| mc13000460 | 1 6 7 8 10 | mc6000006 | 7 10 21 |
| mc13000461 | 4 7 21 | mc6000008 | 8 13 21 |
| mc13000462 | 6 7 9 10 19 21 | mc6000009 | 7 10 21 |
| mc13000472 | 4 7 16 21 | mc6000011 | 9 21 |
| mc13000475 | 4 7 21 24 | mc6000017 | 7 9 12 17 21 |
| mc13000479 | 4 7 16 21 | mc6000021 | 12 13 19 21 |
| mc13000481 | 1 7 | mc6000025 | 7 8 17 21 22 |
| mc13000493 | 3 7 11 21 | mc6000027 | 1 7 11 21 |
| mc13000494 | 6 8 | mc6000029 | 1 11 |
| mc13000504 | 6 8 | mc6000034 | 4 7 10 21 24 |
| mc13000521 | 8 | mc6000037 | 2 6 7 12 17 21 |
| mc13000523 | 1 2 7 9 | mc6000040 | 7 9 21 |
| mc13000526 | 1 6 7 10 15 | mc6000048 | 6 7 |
| mc13000527 | 1 11 21 23 24 | mc6000059 | 10 12 13 21 |
| mc13000545 | 1 4 6 7 10 21 | mc6000060 | 1 |
| mc13000549 | 1 2 17 21 | mc6000063 | 1 6 20 21 |
| mc13000553 | 1 3 7 21 | mc6000071 | 3 7 21 |
| mc13000555 | 4 7 21 | mc6000075 | 21 |
| mc13000561 | 2 7 9 21 | mc6000078 | 9 21 |
| mc13000562 | 5 7 21 | mc6000097 | 2 7 9 21 |
| mc13000569 | 16 | mc6000123 | 1 2 6 |
| mc13000572 | 1 6 7 13 21 | mc6000134 | 4 7 16 21 |
| mc13000576 | 11 13 21 | mc6000136 | 7 |
| mc13000586 | 7 10 21 | mc6000140 | 7 8 9 10 21 |
| mc13000588 | 1 7 | mc6000142 | 7 8 9 10 21 |
| mc13000592 | 10 15 19 24 | mc6000143 | 1 6 7 10 13 16 19 20 21 |
| mc13000594 | 9 21 | mc6000144 | 7 12 13 17 21 |
| mc13000596 | 5 21 | mc6000147 | 7 10 21 |
| mc13000630 | 1 21 | mc6000152 | 4 7 21 |
| mc13000633 | 10 21 | mc6000156 | 1 7 9 |
| mc13000634 | 21 | mc6000162 | 4 7 21 |
| mc13000635 | 1 6 7 | mc6000175 | 3 4 7 15 21 |
| mc13000637 | 4 7 12 15 16 21 | mc6000183 | 10 16 21 |
| mc13000640 | 1 21 | mc6000184 | 15 21 |
| mc13000644 | 3 7 12 15 21 | mc6000192 | 1 7 11 21 |
| mc13000656 | 4 7 21 | mc6000198 | 7 9 21 22 24 |
| mc13000659 | 7 9 10 11 12 13 16 20 21 | mc6000203 | 7 9 21 |
| mc13000672 | 3 4 7 21 | mc6000208 | 2 7 9 21 |
| mc13000673 | 21 | mc6000209 | 4 7 10 21 |
| mc13000674 | 3 7 17 21 | mc6000214 | 1 7 9 11 12 16 21 |
| mc13000683 | 21 | mc6000221 | 10 21 23 |
| mc13000696 | 4 7 16 21 | mc6000222 | 3 7 11 17 21 |
| mc13000698 | 7 12 13 19 21 | mc6000231 | 10 12 13 |
| mc13000702 | 6 7 17 21 | mc6000243 | 7 21 |
| mc13000716 | 7 8 10 12 17 21 | mc6000244 | 7 16 21 |
| mc13000718 | 7 21 | mc6000245 | 4 7 20 21 24 |
| mc13000719 | 2 7 9 17 21 | mc6000247 | 1 3 4 7 21 |
| mc13000722 | 6 7 21 | mc6000252 | 21 |
| mc13000724 | 1 21 | mc6000258 | 1 7 10 12 13 15 19 21 |
| mc13000725 | 2 9 | mc6000260 | 7 21 |
| mc13000726 | 6 7 10 21 | mc6000263 | 1 |
| mc13000729 | 6 7 21 | mc6000265 | 7 17 |
| mc13000747 | 4 7 8 21 | mc6000270 | 7 21 |
| mc13000754 | 4 7 18 21 | mc6000271 | 6 10 12 13 |
| mc13000757 | 4 7 21 | mc6000275 | 1 2 7 9 |
| mc13000761 | 7 12 | mc6000279 | 4 7 21 |
| mc13000764 | 7 8 21 | mc6000282 | 6 7 10 12 21 |
| mc13000765 | 7 9 21 | mc6000283 | 10 16 18 19 20 |
| mc13000770 | 21 | mc6000292 | 2 4 7 21 |
| mc13000772 | 15 | mc6000294 | 6 7 |
| mc13000774 | 5 7 21 | mc6000295 | 1 3 6 7 15 21 |
| mc13000789 | 3 16 22 | mc6000302 | 6 7 21 |
| mc13000794 | 2 7 8 9 10 13 15 21 | mc6000303 | 6 7 |
| mc13000795 | 7 10 12 13 | mc6000305 | 1 9 11 |
| mc13000798 | 1 3 6 8 | mc6000320 | 1 6 7 12 21 |
| mc13000802 | 7 10 15 16 21 | mc6000337 | 21 |
| mc13000805 | 3 7 8 10 15 16 18 21 | mc6000342 | 6 7 9 19 21 |
| mc13000814 | 6 7 10 13 | mc6000349 | 9 21 |
| mc13000817 | 1 7 21 | mc6000351 | 1 7 |
| mc13000818 | 4 7 21 | mc6000356 | 1 2 21 22 |
| mc13000821 | 1 6 7 10 15 16 20 21 | mc6000360 | 7 13 17 21 |
| mc13000824 | 1 21 | mc6000366 | 3 16 21 |
| mc13000839 | 8 10 13 21 24 | mc6000372 | 4 6 7 21 |
| mc13000840 | 9 12 21 | mc6000375 | 9 |
| mc13000843 | 10 | mc6000377 | 7 18 |
| mc13000848 | 4 7 9 21 | mc6000385 | 3 7 13 15 16 20 21 |
| mc13000857 | 7 9 21 | mc6000392 | 7 21 |
| mc13000858 | 15 | mc6000394 | 1 21 |
| mc13000862 | 1 8 10 13 21 24 | mc6000399 | 4 6 7 8 10 21 |
| mc13000868 | 7 10 15 16 | mc6000401 | 7 9 21 24 |
| mc13000870 | 4 10 21 | mc6000418 | 7 |
| mc13000872 | 7 15 21 | mc6000421 | 1 3 4 6 7 8 15 21 |
| mc13000873 | 1 6 7 10 20 21 | mc6000427 | 1 2 7 9 21 |
| mc13000874 | 1 3 4 6 7 8 10 16 21 | mc6000428 | 6 7 21 |
| mc13000875 | 7 9 10 21 | mc6000434 | 9 |
| mc13000877 | 4 7 21 | mc6000435 | 7 9 10 |
| mc13000878 | 7 9 21 | mc6000436 | 6 7 21 |
| mc13000879 | 3 8 10 21 | mc6000438 | 1 |
| mc13000881 | 7 9 21 | mc6000439 | 2 7 9 21 |
| mc13000883 | 3 7 16 17 24 | mc6000453 | 1 6 |
| mc13000884 | 6 21 | mc6000455 | 1 6 7 10 |
| mc13000889 | 7 15 16 17 21 | mc6000456 | 3 7 21 |
| mc13000891 | 7 15 16 20 21 | mc6000458 | 10 12 13 21 24 |
| mc13000892 | 7 10 21 | mc6000462 | 7 13 21 |
| mc13000893 | 3 4 7 21 | mc6000463 | 10 12 13 21 24 |
| mc13000896 | 9 21 | mc6000465 | 1 7 21 |
| mc13000897 | 1 7 21 | mc6000470 | 6 13 21 |
| mc13000899 | 7 10 13 18 21 | mc6000496 | 7 8 10 21 |
| mc13000906 | 9 12 16 21 22 23 24 | mc6000497 | 4 7 9 |
| mc13000911 | 3 7 17 21 | mc6000501 | 6 7 |
| mc13000915 | 4 7 15 16 18 21 | mc6000503 | 21 |
| mc13000921 | 7 10 13 15 21 | mc6000505 | 21 |
| mc13000922 | 6 | mc6000507 | 21 |
| mc13000923 | 12 13 21 | mc6000509 | 11 21 |
| mc13000925 | 7 12 21 | mc6000511 | 6 7 11 15 21 |
| mc13000926 | 4 10 18 | mc6000512 | 9 |
| mc13000934 | 7 21 | mc6000514 | 7 12 21 |
| mc13000943 | 6 21 | mc6000515 | 6 7 10 21 |
| mc13000945 | 1 7 10 21 | mc6000542 | 10 12 13 21 |
| mc13000947 | 7 9 17 21 | mc6000548 | 4 7 20 21 |
| mc13000948 | 4 7 21 | mc6000549 | 1 7 |
| mc13000952 | 6 9 21 | mc6000574 | 3 6 7 15 |
| mc13000959 | 1 6 7 10 13 15 16 18 20 21 | mc6000576 | 6 7 21 |
| mc13000967 | 7 18 21 | mc6000592 | 21 |
| mc13000972 | 21 | mc6000599 | 11 12 21 |
| mc13000980 | 7 21 | mc6000607 | 7 |
| mc13000981 | 1 7 21 | mc6000611 | 7 13 21 |
| mc13000990 | 3 7 15 | mc6000619 | 1 2 7 9 21 |
| mc13000996 | 1 6 7 10 15 21 | mc6000626 | 1 7 10 12 21 |
| mc13001005 | 6 21 | mc6000628 | 7 |
| mc13001006 | 6 | mc6000632 | 1 |
| mc13001009 | 6 21 | mc6000634 | 1 6 7 21 |
| mc13001013 | 6 21 | mc6000642 | 10 13 21 |
| mc13001025 | 1 2 6 | mc6000649 | 20 21 |
| mc13001032 | 3 21 | mc6000650 | 1 9 |
| mc13001033 | 7 8 10 15 16 20 21 | mc6000658 | 10 12 21 |
| mc13001045 | 1 21 | mc6000674 | 4 7 21 |
| mc13001047 | 4 7 21 | mc6000682 | 6 10 21 |
| mc13001048 | 1 7 21 | mc6000686 | 4 7 21 |
| mc13001049 | 1 6 8 10 21 | mc6000687 | 4 7 15 16 20 21 |
| mc13001053 | 6 7 21 | mc6000690 | 4 7 15 16 21 |
| mc13001054 | 1 6 7 10 | mc6000691 | 4 7 21 |
| mc13001078 | 2 6 7 9 21 | mc6000692 | 4 7 16 20 21 |
| mc13001081 | 1 | mc6000693 | 4 7 21 |
| mc13001101 | 1 7 9 23 | mc6000694 | 4 7 16 21 |
| mc13001104 | 1 7 10 21 24 | mc6000695 | 4 7 21 |
| mc13001117 | 1 3 7 21 | mc6000697 | 4 7 21 |
| mc13001119 | 1 | mc6000698 | 4 7 21 |
| mc13001160 | 4 7 21 | mc6000700 | 4 7 21 |
| mc13001173 | 4 7 21 | mc6000702 | 4 7 16 21 |
| mc13001184 | 4 7 21 | mc6000706 | 1 7 21 |
| mc13001189 | 5 21 | mc6000708 | 21 |
| mc13001191 | 1 7 | mc6000709 | 4 7 8 21 |
| mc13001193 | 9 12 21 22 | mc6000726 | 4 7 21 |
| mc13001195 | 11 15 21 | mc6000735 | 7 8 10 |
| mc13001196 | 1 3 7 11 17 21 | mc6000741 | 6 9 |
| mc13001197 | 9 21 | mc6000742 | 6 7 21 |
| mc13001201 | 4 7 21 | mc6000748 | 6 7 9 21 22 24 |
| mc13001210 | 2 7 9 | mc6000750 | 7 10 15 21 |
| mc13001213 | 6 7 9 19 21 | mc6000753 | 5 7 21 |
| mc13001214 | 7 8 12 | mc6000754 | 7 10 20 21 |
| mc13001215 | 10 12 15 | mc6000755 | 1 |
| mc13001222 | 6 7 10 20 21 | mc6000758 | 9 21 |
| mc13001238 | 1 2 9 11 12 | mc6000761 | 7 10 16 21 24 |
| mc13001239 | 7 | mc6000765 | 4 19 20 21 23 24 |
| mc13001244 | 10 | mc6000770 | 8 21 |
| mc13001268 | 4 7 10 13 16 21 | mc6000772 | 1 7 10 24 |
| mc13001294 | 3 7 21 | mc6000785 | 6 7 21 |
| mc13001305 | 4 7 8 16 19 21 | mc6000786 | 1 7 |
| mc13001322 | 3 4 7 8 21 | mc6000809 | 1 6 7 10 |
| mc13001323 | 7 8 10 21 | mc6000812 | 21 |
| mc13001329 | 2 7 9 21 | mc6000837 | 1 7 9 |
| mc13001337 | 7 12 16 21 | mc6000867 | 7 9 12 21 23 24 |
| mc13001340 | 7 12 21 | mc6000873 | 4 7 15 18 20 21 |
| mc13001343 | 3 11 21 | mc6000879 | 1 11 |
| mc13001351 | 6 7 9 | mc6000890 | 21 |
| mc13001359 | 1 7 21 | mc6000892 | 7 21 |
| mc13001360 | 7 | mc6000900 | 3 7 21 |
| mc13001362 | 1 2 7 21 | mc6000915 | 7 10 21 |
| mc13001363 | 8 10 | mc6000917 | 3 7 8 11 17 21 |
| mc13001366 | 1 3 7 11 21 | mc6000924 | 7 21 |
| mc13001367 | 1 7 | mc6000925 | 10 21 |
| mc13001369 | 4 7 | mc6000927 | 7 10 13 15 21 |
| mc13001371 | 7 | mc6000928 | 11 21 |
| mc13001372 | 4 7 21 | mc6001015 | 1 2 |
| mc13001374 | 2 9 | mc6001016 | 3 5 6 7 8 10 11 21 |
| mc13001375 | 7 12 21 | mc6001024 | 7 9 |
| mc13001382 | 7 | mc6001025 | 2 3 4 7 8 9 19 20 21 |
| mc13001386 | 6 7 12 21 | mc6001027 | 7 10 13 21 |
| mc13001387 | 4 7 8 15 | mc6001031 | 6 7 21 |
| mc13001391 | 1 21 | mc6001033 | 9 |
| mc13001392 | 1 7 21 | mc6001037 | 10 |
| mc13001393 | 1 7 21 | mc6001038 | 5 21 |
| mc13001405 | 6 9 21 | mc6001041 | 21 |
| mc13001409 | 6 7 11 17 21 | mc6001044 | 1 4 7 21 |
| mc13001410 | 4 7 16 21 | mc6001046 | 1 6 21 |
| mc13001415 | 1 | mc6001047 | 7 |
| mc13001420 | 7 15 16 20 | mc6001049 | 1 13 21 |
| mc13001422 | 7 10 | mc6001050 | 6 7 |
| mc13001424 | 10 13 21 | mc6001054 | 7 |
| mc13001427 | 10 13 21 | mc6001056 | 7 9 21 |
| mc13001429 | 10 13 | mc6001057 | 1 6 7 |
| mc13001444 | 1 3 7 11 21 | mc6001064 | 7 11 13 17 21 |
| mc13001445 | 1 6 | mc6001068 | 9 21 |
| mc13001459 | 21 | mc6001070 | 3 4 7 10 16 21 |
| mc13001460 | 5 7 21 | mc6001072 | 6 7 9 21 |
| mc13001461 | 1 6 7 11 12 13 16 17 20 21 | mc6001077 | 1 2 7 21 |
| mc13001462 | 7 15 21 24 | mc6001095 | 1 7 12 21 |
| mc13001471 | 3 8 15 21 | mc6001097 | 21 |
| mc13001475 | 4 21 | mc6001108 | 7 13 21 |
| mc13001476 | 7 21 | mc6001109 | 7 21 |
| mc13001478 | 4 7 21 | mc6001111 | 7 21 |
| mc13001494 | 6 7 9 21 | mc6001119 | 21 |
| mc13001506 | 5 21 | mc6001134 | 7 |
| mc13001507 | 7 17 21 24 | mc6001135 | 5 21 |
| mc13001522 | 4 21 | mc6001140 | 10 13 21 |
| mc13001523 | 1 7 11 15 | mc6001141 | 3 7 21 |
| mc13001529 | 3 4 7 21 | mc6001142 | 1 2 23 |
| mc13001530 | 7 17 21 | mc6001145 | 7 10 12 15 16 18 21 |
| mc13001533 | 1 3 7 9 11 16 21 | mc6001148 | 1 7 10 |
| mc13001539 | 1 3 4 6 7 8 21 | mc6001149 | 6 7 10 21 |
| mc13001543 | 1 3 6 7 8 21 | mc6001150 | 6 11 15 21 |
| mc13001547 | 3 7 21 | mc6001152 | 1 3 4 7 9 21 |
| mc13001549 | 9 21 | mc6001154 | 7 |
| mc13001577 | 13 21 22 24 | mc6001156 | 4 7 21 |
| mc13001596 | 4 7 21 | mc6001160 | 1 11 21 |
| mc13001601 | 6 7 21 | mc6001161 | 7 |
| mc13001602 | 7 | mc6001162 | 7 21 |
| mc13001604 | 6 7 10 21 | mc6001166 | 6 7 18 |
| mc13001605 | 6 | mc6001168 | 1 7 9 21 |
| mc13001611 | 7 | mc6001175 | 1 7 21 |
| mc13001625 | 10 21 | mc6001176 | 7 13 17 21 23 |
| mc13001632 | 6 7 9 21 | mc6001177 | 6 7 10 15 21 |
| mc13001634 | 1 4 21 | mc6001179 | 7 9 10 |
| mc13001635 | 2 7 9 17 21 | mc6001181 | 7 21 |
| mc13001666 | 4 7 21 | mc6001183 | 4 7 16 20 21 |
| mc13001695 | 7 9 10 21 | mc6001184 | 7 9 12 13 21 24 |
| mc13001701 | 1 3 6 7 8 10 | mc6001186 | 21 |
| mc13001716 | 1 6 7 8 10 21 | mc6001188 | 1 7 |
| mc13001738 | 3 4 7 21 | mc6001193 | 4 7 21 |
| mc13001739 | 3 4 7 | mc6001195 | 7 17 21 |
| mc13001743 | 11 | mc6001200 | 1 7 10 16 21 |
| mc13001745 | 6 15 21 | mc6001208 | 1 |
| mc13001747 | 6 21 | mc6001211 | 9 12 21 |
| mc13001749 | 21 | mc6001216 | 6 7 11 21 |
| mc13001760 | 9 10 21 | mc6001217 | 6 7 21 |
| mc13001762 | 6 11 | mc6001219 | 4 7 21 |
| mc13001777 | 2 7 9 21 | mc6001229 | 7 |
| mc13001778 | 10 16 18 20 21 | mc6001235 | 4 7 15 16 21 |
| mc13001786 | 1 7 | mc6001236 | 6 7 19 21 24 |
| mc13001787 | 7 10 12 21 | mc6001237 | 3 7 15 20 |
| mc13001817 | 12 13 15 16 19 20 21 24 | mc6001243 | 4 7 21 |
| mc13001823 | 7 9 12 13 21 24 | mc6001250 | 4 5 7 21 |
| mc13001826 | 5 21 | mc6001253 | 6 |
| mc13001829 | 7 10 15 18 20 21 | mc6001254 | 4 7 21 |
| mc13001833 | 2 9 11 21 | mc6001256 | 4 7 15 16 21 |
| mc13001834 | 5 7 | mc6001257 | 7 20 21 |
| mc13001837 | 10 | mc6001260 | 21 |
| mc13001838 | 1 7 21 | mc6001263 | 7 10 21 |
| mc13001839 | 7 10 15 16 20 | mc6001265 | 3 15 21 |
| mc13001840 | 3 7 13 15 17 21 | mc6001266 | 21 |
| mc13001848 | 7 9 | mc6001268 | 15 19 20 21 |
| mc13001864 | 4 7 21 | mc6001273 | 10 12 |
| mc13001866 | 1 6 10 21 | mc6001281 | 21 |
| mc13001868 | 21 | mc6001282 | 7 |
| mc14000007 | 1 3 7 15 21 | mc6001285 | 1 4 7 15 21 |
| mc14000009 | 1 | mc6001289 | 21 |
| mc14000010 | 4 21 | mc6001292 | 1 6 21 |
| mc14000011 | 1 6 7 10 | mc6001296 | 4 7 13 18 21 |
| mc14000012 | 1 2 21 | mc6001300 | 5 7 21 |
| mc14000015 | 1 2 21 | mc6001303 | 3 4 7 9 15 21 |
| mc14000029 | 1 3 7 | mc6001313 | 4 7 9 |
| mc14000048 | 1 2 6 7 8 10 21 | mc6001315 | 3 7 21 |
| mc14000056 | 7 13 20 21 | mc6001316 | 10 |
| mc14000057 | 7 9 21 22 24 | mc6001328 | 12 21 |
| mc14000068 | 9 21 24 | mc6001329 | 21 |
| mc14000079 | 13 21 | mc6001331 | 1 2 7 9 11 21 |
| mc14000080 | 6 7 8 11 21 | mc6001339 | 10 12 |
| mc14000083 | 10 13 21 | mc6001341 | 10 |
| mc14000093 | 21 | mc6001350 | 1 |
| mc14000101 | 1 21 | mc6001351 | 7 21 |
| mc14000102 | 1 3 7 12 16 20 21 | mc6001355 | 4 7 9 21 |
| mc14000120 | 4 7 10 13 15 16 18 21 | mc6001357 | 1 7 9 |
| mc14000122 | 4 7 10 21 | mc6001368 | 9 21 |
| mc14000124 | 5 7 21 | mc6001369 | 3 4 21 |
| mc14000125 | 7 10 | mc6001370 | 7 21 |
| mc14000138 | 7 12 21 | mc6001379 | 1 3 7 11 21 |
| mc14000143 | 9 21 | mc6001380 | 4 7 21 |
| mc14000148 | 1 | mc6001386 | 9 21 |
| mc14000152 | 6 7 11 | mc6001398 | 7 9 21 |
| mc14000153 | 5 21 | mc6001400 | 7 10 21 |
| mc14000155 | 3 7 12 21 | mc6001426 | 1 7 10 17 21 |
| mc14000157 | 1 6 7 10 18 19 20 | mc6001437 | 9 21 |
| mc14000161 | 7 21 | mc6001438 | 21 |
| mc14000163 | 7 9 17 21 | mc6001473 | 3 6 7 8 |
| mc14000167 | 1 6 21 | mc6001474 | 7 9 21 |
| mc14000169 | 1 6 21 | mc6001476 | 21 |
| mc14000173 | 7 12 21 | mc6001485 | 4 7 18 21 |
| mc14000174 | 6 9 21 | mc6001500 | 4 7 21 |
| mc14000175 | 1 7 21 | mc6001512 | 10 21 |
| mc14000178 | 1 3 4 6 7 17 21 | mc6001513 | 10 12 21 |
| mc14000180 | 1 6 16 18 24 | mc6001518 | 7 15 21 |
| mc14000184 | 9 12 15 21 | mc6001536 | 6 7 10 21 |
| mc14000185 | 1 6 21 | mc6001545 | 7 12 21 |
| mc14000206 | 1 2 3 7 9 21 | mc6001550 | 12 13 16 22 |
| mc14000237 | 4 7 21 | mc6001553 | 1 7 9 10 12 21 |
| mc14000238 | 6 15 21 | mc6001564 | 10 21 |
| mc14000242 | 7 21 | mc6001565 | 4 7 21 |
| mc14000251 | 6 7 11 | mc6001567 | 6 7 |
| mc14000258 | 1 | mc6001572 | 21 |
| mc14000263 | 10 | mc6001576 | 7 21 |
| mc14000265 | 3 10 21 | mc6001577 | 7 9 10 21 |
| mc14000266 | 4 7 16 20 21 | mc6001581 | 4 7 21 |
| mc14000267 | 7 10 21 | mc6001583 | 1 6 7 11 21 |
| mc14000273 | 8 | mc6001606 | 4 7 8 21 |
| mc14000281 | 7 21 | mc6001617 | 3 6 7 11 21 |
| mc14000298 | 3 7 10 15 16 18 21 | mc6001619 | 7 8 |
| mc14000304 | 21 | mc6001630 | 2 4 7 9 21 |
| mc14000322 | 7 | mc6001631 | 3 7 11 21 |
| mc14000323 | 7 17 21 | mc6001632 | 6 7 10 16 |
| mc14000324 | 10 21 | mc6001633 | 4 7 8 21 |
| mc14000326 | 7 9 10 12 21 | mc6001638 | 7 15 21 |
| mc14000334 | 1 4 7 10 21 | mc6001640 | 10 |
| mc14000336 | 1 2 7 15 | mc6001641 | 10 |
| mc14000337 | 1 6 7 10 13 | mc6001642 | 7 |
| mc14000341 | 4 21 | mc6001649 | 4 6 7 21 |
| mc14000345 | 21 | mc6001650 | 1 6 7 10 15 |
| mc14000346 | 1 7 8 21 | mc6001652 | 10 21 |
| mc14000347 | 1 7 8 | mc6001653 | 7 9 17 21 |
| mc14000348 | 1 3 6 7 10 21 | mc6001654 | 7 9 12 13 21 |
| mc14000349 | 6 7 21 | mc6001658 | 9 12 21 22 24 |
| mc14000356 | 7 10 12 13 21 | mc6001659 | 9 12 21 |
| mc14000357 | 7 10 12 17 21 | mc6001663 | 10 12 |
| mc14000359 | 7 13 17 19 21 | mc6001666 | 6 7 8 9 |
| mc14000361 | 4 6 7 21 | mc6001670 | 4 21 |
| mc14000362 | 6 7 21 | mc6001674 | 8 21 |
| mc14000366 | 6 7 21 | mc6001677 | 1 4 7 10 13 17 21 23 |
| mc14000367 | 7 8 10 21 | mc6001678 | 1 3 4 7 21 |
| mc14000369 | 1 | mc6001680 | 6 7 21 |
| mc14000370 | 4 7 21 | mc6001681 | 3 6 7 10 15 21 |
| mc14000372 | 1 7 21 | mc6001684 | 4 7 21 23 |
| mc14000373 | 1 | mc6001685 | 5 7 21 |
| mc14000380 | 7 21 | mc6001687 | 1 3 7 11 15 21 |
| mc14000381 | 2 9 | mc6001688 | 21 |
| mc14000383 | 21 | mc6001690 | 6 10 12 13 |
| mc14000386 | 11 12 21 | mc6001691 | 3 4 7 21 |
| mc14000390 | 1 12 13 16 18 19 | mc6001699 | 7 21 |
| mc14000410 | 8 | mc6001708 | 4 7 21 |
| mc14000415 | 1 6 8 10 | mc6001719 | 12 13 15 |
| mc14000421 | 15 21 | mc6001736 | 9 21 |
| mc14000422 | 15 21 | mc6001741 | 1 7 17 21 |
| mc14000423 | 1 6 7 9 13 21 | mc6001742 | 6 7 8 10 21 |
| mc14000428 | 7 13 | mc6001749 | 7 9 10 12 13 21 |
| mc14000429 | 10 | mc6001754 | 21 |
| mc14000435 | 1 7 21 | mc6001756 | 1 2 7 12 21 24 |
| mc14000437 | 21 22 | mc6001760 | 6 |
| mc14000439 | 7 10 21 | mc6001767 | 3 7 21 |
| mc14000442 | 6 10 12 13 | mc6001768 | 1 6 7 8 10 21 |
| mc14000447 | 1 7 9 10 12 | mc6001769 | 7 12 |
| mc14000466 | 10 13 21 24 | mc6001776 | 10 21 |
| mc14000469 | 7 10 12 13 21 | mc6001778 | 1 |
| mc14000477 | 1 7 10 15 20 | mc6001783 | 15 21 |
| mc14000499 | 1 7 | mc6001784 | 7 15 21 |
| mc14000500 | 7 9 13 21 | mc6001795 | 6 |
| mc14000503 | 7 9 13 21 | mc6001800 | 8 9 13 21 23 |
| mc14000505 | 7 9 13 21 | mc6001803 | 8 21 |
| mc14000509 | 10 | mc6001810 | 7 10 |
| mc14000511 | 10 | mc6001812 | 7 9 10 21 |
| mc14000516 | 2 6 9 11 21 | mc6001813 | 4 6 7 16 17 18 21 |
| mc14000517 | 6 7 8 11 21 | mc6001819 | 7 21 |
| mc14000518 | 1 6 7 16 21 | mc6001820 | 1 4 7 13 18 21 |
| mc14000519 | 1 6 21 | mc6001823 | 15 21 |
| mc14000520 | 7 12 15 21 | mc6001824 | 16 21 |
| mc14000524 | 1 7 | mc6001832 | 4 7 21 |
| mc14000538 | 6 7 10 15 16 18 19 20 21 | mc6001833 | 10 12 13 |
| mc14000550 | 7 15 | mc6001834 | 9 |
| mc14000557 | 1 | mc6001836 | 10 |
| mc14000560 | 10 15 | mc6001840 | 7 13 |
| mc14000563 | 7 21 | mc6001842 | 7 13 |
| mc14000564 | 3 10 21 | mc6001843 | 7 10 13 |
| mc14000566 | 6 | mc6001844 | 7 12 13 |
| mc14000570 | 7 9 21 | mc6001854 | 10 |
| mc14000576 | 7 | mc6001859 | 10 21 |
| mc14000585 | 4 7 12 13 15 16 18 20 21 | mc6001860 | 6 7 9 21 |
| mc14000612 | 10 13 | mc6001862 | 7 12 |
| mc14000615 | 10 13 | mc6001863 | 6 21 |
| mc14000620 | 10 13 | mc6001865 | 6 7 13 21 |
| mc14000622 | 2 9 10 12 13 | mc6001867 | 4 5 21 |
| mc14000634 | 7 | mc6001868 | 6 7 8 21 |
| mc14000636 | 1 3 6 7 21 | mc6001869 | 6 7 8 10 21 22 |
| mc14000637 | 7 11 17 21 | mc6001871 | 7 11 21 |
| mc14000639 | 6 7 | mc6001874 | 10 |
| mc14000640 | 3 4 7 21 | mc6001875 | 1 2 |
| mc14000642 | 1 | mc6001876 | 6 7 |
| mc14000647 | 1 3 5 7 15 16 20 21 | mc6001877 | 3 |
| mc14000648 | 1 3 7 | mc6001879 | 7 21 |
| mc14000652 | 1 3 7 21 | mc6001881 | 6 7 10 12 13 15 21 |
| mc14000653 | 1 3 7 21 | mc6001882 | 7 10 13 15 21 |
| mc14000675 | 15 | mc6001884 | 21 |
| mc14000678 | 8 | mc6001885 | 6 7 8 21 |
| mc14000679 | 7 21 | mc6001890 | 3 4 7 21 |
| mc14000683 | 4 7 21 | mc6001891 | 7 8 16 18 |
| mc14000685 | 5 | mc6001892 | 3 4 7 21 |
| mc14000686 | 16 21 | mc6001897 | 3 7 17 21 |
| mc14000688 | 7 9 10 21 | mc6001898 | 5 21 |
| mc14000689 | 4 7 21 | mc6001899 | 7 9 21 22 23 24 |
| mc14000690 | 1 4 7 21 | mc6001900 | 21 |
| mc14000691 | 4 7 21 | mc6001904 | 7 10 15 20 21 |
| mc14000695 | 10 13 | mc6001905 | 7 9 12 13 |
| mc14000700 | 10 13 | mc6001906 | 7 10 15 20 21 |
| mc14000725 | 6 7 15 21 | mc6001907 | 8 |
| mc14000726 | 1 6 | mc6001909 | 7 12 21 |
| mc14000727 | 10 13 21 | mc6001911 | 7 8 12 13 21 |
| mc14000732 | 6 7 21 | mc6001914 | 4 7 12 15 16 18 19 20 21 |
| mc14000733 | 1 7 9 21 | mc6001920 | 7 9 21 |
| mc14000734 | 4 7 10 | mc6001925 | 9 12 21 |
| mc14000735 | 1 6 21 | mc6001928 | 2 7 9 21 |
| mc14000737 | 7 8 12 15 17 21 | mc6001929 | 6 7 8 10 12 16 22 |
| mc14000739 | 6 7 11 13 21 | mc6001930 | 1 6 7 |
| mc14000741 | 7 12 21 | mc6001931 | 3 7 11 21 |
| mc14000743 | 3 7 15 21 | mc6001937 | 7 10 12 15 16 21 |
| mc14000745 | 4 7 13 15 19 21 | mc6001938 | 1 |
| mc14000746 | 9 21 | mc6001963 | 5 7 21 |
| mc14000748 | 4 7 21 | mc6001964 | 7 10 12 21 |
| mc14000751 | 7 15 21 | mc6001965 | 4 7 20 21 |
| mc14000752 | 4 7 9 21 | mc6001967 | 4 6 7 10 21 |
| mc14000753 | 5 7 9 21 | mc6001969 | 6 7 21 |
| mc14000754 | 10 12 21 | mc6001972 | 8 21 |
| mc14000755 | 8 12 13 15 19 21 | mc6001976 | 7 10 11 21 |
| mc14000756 | 12 13 15 21 | mc6001988 | 7 18 21 |
| mc14000757 | 2 7 9 13 17 19 21 23 | mc6001991 | 7 |
| mc14000763 | 6 7 9 17 21 | mc6001995 | 7 13 21 |
| mc14000765 | 21 | mc6001997 | 7 10 21 |
| mc14000768 | 1 21 | mc6001998 | 7 10 |
| mc14000773 | 1 21 | mc6001999 | 7 10 |
| mc14000778 | 4 5 7 12 20 21 | mc6002001 | 7 10 12 13 21 |
| mc14000781 | 21 | mc6002005 | 7 10 11 |
| mc14000782 | 6 7 8 21 | mc6002006 | 7 13 21 22 |
| mc14000785 | 6 7 21 | mc6002009 | 7 10 |
| mc14000786 | 4 7 10 21 | mc6002013 | 10 |
| mc14000787 | 3 7 17 21 | mc6002026 | 7 10 11 21 |
| mc14000788 | 5 6 7 9 21 | mc6002029 | 7 10 11 12 21 |
| mc14000789 | 9 21 | mc6002032 | 1 6 7 10 |
| mc14000790 | 1 6 7 15 16 | mc6002037 | 4 7 21 |
| mc14000791 | 1 6 7 8 10 | mc6002043 | 10 12 13 21 24 |
| mc14000792 | 4 6 7 21 | mc6002068 | 21 |
| mc14000794 | 7 21 | mc6002070 | 3 4 7 15 21 |
| mc14000795 | 1 6 7 15 19 21 | mc6002075 | 6 7 15 21 |
| mc14000796 | 1 6 7 | mc6002082 | 10 21 |
| mc14000798 | 2 7 | mc6002084 | 1 3 7 8 12 15 17 21 |
| mc14000800 | 7 15 21 | mc6002086 | 3 4 7 21 |
| mc14000801 | 10 | mc6002087 | 10 |
| mc14000802 | 10 | mc6002088 | 7 10 13 21 |
| mc14000803 | 1 7 10 21 24 | mc6002089 | 1 7 21 |
| mc14000804 | 1 6 7 10 15 | mc6002093 | 7 8 |
| mc14000805 | 1 2 4 7 19 20 | mc6002095 | 15 |
| mc14000811 | 6 21 | mc6002099 | 7 9 10 12 13 21 22 |
| mc14000817 | 6 21 | mc6002113 | 1 4 7 8 21 |
| mc14000823 | 6 21 | mc6002118 | 7 17 21 |
| mc14000824 | 6 21 | mc6002120 | 4 7 21 |
| mc14000825 | 6 15 21 | mc6002124 | 1 6 |
| mc14000833 | 6 15 21 | mc6002125 | 7 15 16 20 21 |
| mc14000836 | 6 7 21 | mc6002126 | 21 |
| mc14000841 | 3 4 7 11 21 | mc6002130 | 7 |
| mc14000846 | 4 7 8 21 | mc6002135 | 1 6 8 10 21 |
| mc14000848 | 9 10 21 | mc6002136 | 7 8 17 21 |
| mc14000852 | 7 9 12 21 | mc6002140 | 1 4 7 10 |
| mc14000857 | 1 21 | mc6002141 | 1 2 21 |
| mc14000860 | 6 7 10 15 16 20 | mc6002145 | 11 21 24 |
| mc14000862 | 10 11 12 13 15 21 24 | mc6002151 | 7 8 10 12 13 |
| mc14000863 | 1 3 7 9 13 | mc6002156 | 1 7 10 21 |
| mc14000864 | 7 9 21 | mc6002158 | 1 9 10 15 |
| mc14000865 | 1 3 6 7 | mc6002159 | 7 12 13 17 21 |
| mc14000867 | 3 4 6 7 21 | mc6002170 | 4 7 18 19 21 |
| mc14000871 | 7 | mc6002178 | 1 9 10 |
| mc14000875 | 3 6 7 9 10 15 16 20 | mc6002180 | 9 |
| mc14000901 | 6 7 9 21 | mc6002183 | 9 |
| mc14000902 | 1 6 | mc6002188 | 10 21 |
| mc14000914 | 6 7 21 | mc6002190 | 3 7 21 24 |
| mc14000917 | 10 21 | mc6002191 | 9 |
| mc14000921 | 6 7 13 17 19 21 23 | mc6002194 | 1 2 21 |
| mc14000927 | 6 7 9 21 | mc6002196 | 7 9 12 21 |
| mc14000932 | 3 6 7 21 | mc6002198 | 7 9 12 21 |
| mc14000933 | 9 | mc6002205 | 1 7 21 |
| mc14000947 | 1 | mc6002209 | 1 6 15 21 |
| mc14000954 | 7 | mc6002223 | 4 7 18 20 21 |
| mc14000958 | 6 8 17 21 22 | mc6002235 | 1 3 7 21 |
| mc14000959 | 1 7 21 | mc6002238 | 21 |
| mc14000960 | 1 3 7 11 21 | mc6002253 | 10 |
| mc14000961 | 4 7 20 21 24 | mc6002255 | 4 7 8 21 |
| mc14000969 | 1 | mc6002257 | 3 21 |
| mc14000975 | 3 7 11 17 21 | mc6002266 | 4 7 8 21 |
| mc14000976 | 4 7 21 | mc6002267 | 1 6 7 9 21 |
| mc14000981 | 6 11 | mc6002269 | 4 7 10 12 21 |
| mc14000988 | 7 | mc6002275 | 5 21 |
| mc14000991 | 1 21 | mc6002280 | 7 10 13 19 20 21 |
| mc14000996 | 10 16 21 | mc6002286 | 9 21 |
| mc14001000 | 4 7 21 | mc6002291 | 1 21 |
| mc14001001 | 10 12 21 | mc6002307 | 1 21 |
| mc14001003 | 1 6 | mc6002317 | 21 |
| mc14001006 | 1 6 7 10 | mc6002320 | 4 7 21 |
| mc14001007 | 1 3 7 21 | mc6002322 | 6 21 |
| mc14001010 | 10 | mc6002334 | 9 15 16 19 21 |
| mc14001012 | 15 21 | mc6002336 | 10 |
| mc14001013 | 1 2 7 9 21 | mc6002349 | 6 7 13 21 |
| mc14001014 | 6 7 12 21 24 | mc6002353 | 7 |
| mc14001015 | 11 21 | mc6002379 | 1 7 10 21 |
| mc14001016 | 9 10 12 21 | mc6002380 | 9 21 |
| mc14001017 | 6 7 8 10 21 | mc7000001 | 10 21 |
| mc14001020 | 6 7 15 21 | mc7000002 | 2 7 9 21 |
| mc14001021 | 10 | mc7000003 | 7 15 21 24 |
| mc14001026 | 7 10 13 21 | mc7000004 | 4 7 21 |
| mc14001030 | 1 10 16 21 | mc7000008 | 10 |
| mc14001035 | 7 10 15 21 | mc7000009 | 1 3 4 7 21 |
| mc14001045 | 7 9 | mc7000010 | 5 7 21 |
| mc14001051 | 7 17 21 | mc7000012 | 10 13 15 18 |
| mc14001052 | 7 16 17 21 22 | mc7000017 | 10 21 |
| mc14001057 | 7 16 21 24 | mc7000020 | 10 |
| mc14001058 | 6 7 13 16 21 22 24 | mc7000023 | 7 10 15 21 |
| mc14001064 | 1 6 7 10 12 21 | mc7000029 | 10 11 21 |
| mc14001067 | 4 7 16 21 | mc7000031 | 1 |
| mc14001068 | 4 7 16 21 | mc7000032 | 10 |
| mc14001073 | 1 7 21 | mc7000039 | 7 9 12 21 |
| mc14001074 | 6 7 10 | mc7000044 | 7 |
| mc14001080 | 7 8 10 13 15 | mc7000047 | 6 17 21 |
| mc14001087 | 4 7 15 21 | mc7000048 | 2 7 9 21 24 |
| mc14001088 | 3 6 7 9 15 17 21 | mc7000050 | 15 19 21 |
| mc14001092 | 7 21 | mc7000053 | 5 7 21 |
| mc14001093 | 7 12 21 | mc7000057 | 1 21 |
| mc14001097 | 21 | mc7000067 | 4 7 21 |
| mc14001098 | 1 3 4 7 21 | mc7000076 | 7 9 |
| mc14001100 | 7 | mc7000077 | 7 21 |
| mc14001102 | 13 21 23 24 | mc7000085 | 7 10 13 15 |
| mc14001103 | 20 21 24 | mc7000101 | 7 |
| mc14001105 | 4 7 21 | mc7000115 | 4 7 21 |
| mc14001109 | 7 17 | mc7000117 | 7 21 |
| mc14001110 | 7 10 15 21 | mc7000122 | 6 |
| mc14001111 | 3 7 21 | mc7000142 | 4 7 21 |
| mc14001114 | 7 8 10 16 21 | mc7000183 | 4 7 10 |
| mc14001116 | 7 10 16 21 | mc7000184 | 4 7 10 21 |
| mc14001142 | 10 | mc7000185 | 7 21 |
| mc14001143 | 7 | mc7000186 | 5 7 21 |
| mc14001144 | 3 4 7 15 21 | mc7000190 | 1 7 9 21 |
| mc14001145 | 7 15 | mc7000193 | 4 6 7 21 |
| mc14001147 | 4 7 10 21 | mc7000194 | 9 |
| mc14001148 | 1 7 11 | mc7000195 | 6 |
| mc14001149 | 1 2 21 | mc7000232 | 4 7 16 21 |
| mc14001161 | 10 | mc7000234 | 7 12 |
| mc14001163 | 1 | mc7000235 | 7 21 24 |
| mc14001172 | 6 7 21 | mc7000240 | 6 7 9 18 20 21 |
| mc14001182 | 6 9 21 | mc7000241 | 3 12 13 17 21 23 |
| mc14001188 | 4 7 21 | mc7000242 | 9 12 21 22 24 |
| mc14001193 | 7 21 | mc7000246 | 10 12 |
| mc14001199 | 4 7 21 | mc7000247 | 10 12 13 |
| mc14001213 | 6 7 11 | mc7000252 | 7 15 21 |
| mc14001220 | 7 10 13 15 16 18 19 20 21 | mc7000253 | 6 7 8 21 |
| mc14001238 | 3 7 8 21 | mc7000258 | 4 7 10 13 21 |
| mc14001240 | 5 21 | mc7000269 | 6 7 9 17 21 |
| mc14001243 | 4 7 21 | mc7000270 | 9 21 |
| mc14001244 | 4 7 10 13 15 21 | mc7000271 | 6 21 |
| mc14001246 | 6 7 | mc7000272 | 7 |
| mc14001247 | 15 16 20 | mc7000276 | 10 |
| mc14001249 | 7 16 19 21 | mc7000281 | 1 6 7 21 |
| mc14001295 | 1 7 10 17 | mc7000282 | 4 7 10 11 21 |
| mc14001307 | 7 12 | mc7000300 | 21 |
| mc14001344 | 7 21 | mc7000304 | 10 13 |
| mc14001347 | 4 7 16 21 | mc7000305 | 10 |
| mc14001359 | 1 7 | mc7000313 | 1 10 13 15 21 |
| mc14001365 | 4 7 15 20 21 | mc7000315 | 5 7 15 16 |
| mc14001377 | 4 7 21 | mc7000316 | 4 7 21 |
| mc14001396 | 8 10 | mc7000317 | 4 7 8 21 |
| mc14001400 | 6 12 13 | mc7000318 | 4 7 11 21 |
| mc14001413 | 6 21 | mc7000321 | 7 9 12 21 |
| mc14001415 | 4 6 7 21 | mc7000322 | 3 |
| mc14001419 | 7 21 | mc7000323 | 1 6 7 10 |
| mc14001447 | 21 | mc7000326 | 6 |
| mc14001457 | 8 10 13 16 18 20 21 | mc7000333 | 10 21 |
| mc14001477 | 16 | mc7000334 | 13 |
| mc14001482 | 16 | mc7000339 | 21 |
| mc14001501 | 21 | mc7000340 | 3 4 6 7 21 |
| mc14001527 | 1 7 18 21 | mc7000341 | 1 3 7 11 21 |
| mc14001529 | 1 7 | mc7000342 | 4 7 21 |
| mc14001530 | 21 | mc7000344 | 3 4 7 21 |
| mc14001544 | 7 17 | mc7000345 | 7 17 |
| mc14001548 | 6 7 8 11 21 | mc7000348 | 1 6 7 |
| mc14001571 | 7 10 15 | mc7000351 | 7 9 17 18 |
| mc14001591 | 6 7 8 9 21 | mc7000352 | 9 21 |
| mc14001598 | 1 6 7 10 | mc7000355 | 4 6 7 21 |
| mc14001600 | 9 13 21 24 | mc7000359 | 4 21 |
| mc14001609 | 10 21 | mc7000360 | 3 4 7 13 18 21 |
| mc14001611 | 10 | mc7000361 | 1 |
| mc14001616 | 9 21 | mc7000363 | 9 21 |
| mc14001619 | 1 21 | mc7000364 | 9 21 |
| mc14001621 | 7 15 16 20 21 | mc7000365 | 1 7 9 13 21 |
| mc14001622 | 7 15 16 20 21 | mc7000366 | 6 7 9 21 |
| mc14001625 | 7 21 | mc7000367 | 7 10 21 |
| mc14001633 | 7 9 21 | mc7000368 | 10 12 21 |
| mc14001635 | 10 12 | mc7000369 | 6 7 21 |
| mc14001646 | 5 7 21 | mc7000370 | 4 7 21 |
| mc14001648 | 1 2 7 9 21 | mc7000385 | 7 11 15 21 |
| mc14001650 | 7 21 | mc7000405 | 4 7 21 |
| mc14001651 | 9 21 | mc7000407 | 4 7 21 |
| mc14001653 | 7 15 21 | mc7000410 | 7 9 21 |
| mc14001656 | 10 13 | mc7000413 | 10 12 13 17 21 |
| mc14001662 | 1 7 18 19 | mc7000414 | 21 |
| mc14001664 | 6 7 10 12 21 | mc7000418 | 3 11 21 |
| mc14001665 | 7 9 | mc7000420 | 17 21 |
| mc14001667 | 10 | mc7000422 | 7 12 |
| mc14001673 | 7 10 15 | mc7000423 | 21 |
| mc15000003 | 1 7 13 15 16 20 21 | mc7000427 | 21 |
| mc15000005 | 7 10 15 21 | mc7000432 | 5 7 21 |
| mc15000012 | 6 | mc7000433 | 7 10 21 |
| mc15000018 | 11 13 21 | mc7000434 | 8 10 |
| mc15000026 | 5 7 21 | mc7000436 | 7 21 |
| mc15000030 | 10 19 20 | mc7000437 | 1 7 9 21 |
| mc15000039 | 7 9 13 15 16 18 | mc7000438 | 9 10 12 21 |
| mc15000040 | 13 15 21 | mc7000439 | 7 21 |
| mc15000041 | 6 7 13 18 21 22 | mc7000440 | 4 7 13 18 21 |
| mc15000046 | 10 21 | mc7000442 | 4 7 15 21 |
| mc15000053 | 10 21 | mc7000446 | 4 6 7 21 |
| mc15000063 | 7 10 13 15 16 17 20 21 | mc7000448 | 4 7 20 |
| mc15000069 | 9 21 | mc7000449 | 1 16 21 |
| mc15000073 | 3 7 21 | mc7000455 | 1 |
| mc15000092 | 1 3 6 7 21 | mc7000464 | 1 7 21 |
| mc15000097 | 1 | mc7000465 | 1 3 4 7 21 |
| mc15000101 | 10 13 21 | mc7000468 | 3 4 6 7 8 9 10 11 13 15 16 19 20 21 |
| mc15000108 | 7 10 21 | mc7000471 | 6 7 10 21 |
| mc15000113 | 5 21 | mc7000472 | 1 2 7 9 21 |
| mc15000114 | 1 3 21 | mc7000476 | 7 9 11 21 |
| mc15000117 | 4 7 21 | mc7000488 | 1 2 7 9 21 |
| mc15000121 | 9 21 | mc7000496 | 1 2 7 9 13 21 |
| mc15000122 | 1 21 | mc7000502 | 9 11 21 24 |
| mc15000123 | 1 12 13 | mc7000504 | 7 21 |
| mc15000124 | 10 | mc7000505 | 7 9 10 |
| mc15000126 | 6 7 10 12 21 | mc7000506 | 12 15 17 21 |
| mc15000137 | 7 10 21 | mc7000507 | 4 7 21 |
| mc15000140 | 4 7 8 21 | mc7000509 | 1 |
| mc15000145 | 6 10 21 | mc7000510 | 1 |
| mc15000146 | 21 | mc7000511 | 16 20 21 |
| mc15000156 | 7 12 17 20 21 | mc7000512 | 7 9 10 21 |
| mc15000213 | 4 7 21 | mc7000515 | 7 9 12 13 15 17 21 22 |
| mc15000218 | 2 7 9 17 21 | mc7000516 | 1 2 7 9 |
| mc15000221 | 7 21 | mc7000518 | 4 21 |
| mc15000241 | 9 16 21 | mc7000519 | 4 |
| mc15000258 | 12 21 | mc7000522 | 1 10 |
| mc15000267 | 7 12 16 17 19 20 23 24 | mc7000524 | 1 6 7 8 10 |
| mc15000276 | 15 | mc7000527 | 4 7 21 |
| mc15000281 | 1 | mc7000535 | 6 7 8 11 21 |
| mc15000286 | 1 | mc7000540 | 4 7 21 |
| mc15000287 | 6 7 11 21 | mc7000541 | 1 6 7 10 21 |
| mc15000288 | 1 21 | mc7000543 | 4 7 21 |
| mc15000301 | 10 12 15 16 20 | mc7000546 | 7 10 15 16 |
| mc15000302 | 7 12 21 23 | mc7000547 | 6 7 9 21 |
| mc15000304 | 6 | mc7000548 | 4 21 |
| mc15000314 | 4 7 21 | mc7000549 | 5 7 21 |
| mc15000319 | 21 | mc7000550 | 8 |
| mc15000320 | 5 21 | mc7000551 | 1 4 7 10 13 19 21 |
| mc15000322 | 1 5 11 21 24 | mc7000552 | 4 7 21 |
| mc15000323 | 1 4 7 21 | mc7000555 | 7 21 |
| mc15000334 | 7 21 | mc7000558 | 7 12 15 21 |
| mc15000344 | 2 7 9 21 | mc7000562 | 1 6 7 10 12 15 21 |
| mc15000349 | 4 7 21 | mc7000564 | 6 7 |
| mc15000350 | 7 16 | mc7000565 | 6 7 |
| mc15000351 | 6 7 21 | mc7000566 | 5 21 |
| mc15000356 | 4 7 | mc7000567 | 5 7 21 |
| mc15000364 | 7 21 | mc7000570 | 7 8 10 21 |
| mc15000368 | 4 7 | mc7000571 | 3 4 7 21 |
| mc15000370 | 7 9 21 | mc7000573 | 4 7 21 |
| mc15000385 | 15 16 23 | mc7000575 | 1 2 21 |
| mc15000389 | 4 7 16 18 21 | mc7000576 | 7 12 21 24 |
| mc15000393 | 1 | mc7000579 | 6 7 |
| mc15000395 | 1 2 7 9 | mc7000580 | 7 17 21 |
| mc15000398 | 10 16 21 | mc7000581 | 5 7 21 |
| mc15000401 | 9 21 | mc7000582 | 1 6 7 10 |
| mc15000404 | 7 8 9 10 21 | mc7000583 | 9 10 13 21 |
| mc15000409 | 1 | mc7000584 | 8 10 |
| mc15000410 | 7 9 21 | mc7000586 | 8 10 16 21 |
| mc15000422 | 4 7 8 21 | mc7000588 | 6 7 8 11 21 |
| mc15000437 | 17 21 | mc7000593 | 6 8 21 |
| mc15000438 | 4 7 10 21 23 | mc7000594 | 8 |
| mc15000442 | 7 8 10 15 20 21 24 | mc7000615 | 4 7 21 |
| mc15000445 | 6 7 10 21 | mc7000623 | 6 7 17 21 |
| mc15000446 | 5 | mc7000624 | 6 7 11 17 21 |
| mc15000447 | 7 21 | mc7000634 | 1 |
| mc15000450 | 10 | mc7000635 | 21 |
| mc15000453 | 24 | mc7000637 | 7 9 12 15 17 20 21 |
| mc15000455 | 21 | mc7000640 | 7 10 12 21 |
| mc15000457 | 8 10 15 21 | mc7000641 | 1 21 |
| mc15000463 | 9 | mc7000643 | 7 8 9 21 |
| mc15000481 | 4 7 21 | mc7000645 | 10 |
| mc15000492 | 5 | mc7000646 | 6 7 8 10 21 |
| mc15000495 | 3 7 17 21 | mc7000650 | 4 7 8 15 19 21 |
| mc15000506 | 1 16 21 | mc7000652 | 2 7 9 21 |
| mc15000519 | 7 10 15 20 21 | mc7000653 | 4 7 21 |
| mc15000529 | 1 3 7 21 | mc7000660 | 1 2 7 12 |
| mc15000537 | 7 9 12 21 | mc7000661 | 21 |
| mc15000540 | 5 21 | mc7000662 | 21 |
| mc15000541 | 3 7 15 | mc7000664 | 10 21 |
| mc15000542 | 7 21 | mc7000666 | 10 |
| mc15000562 | 4 7 21 | mc7000667 | 7 10 11 12 21 |
| mc15000567 | 9 21 | mc7000668 | 7 12 21 |
| mc15000571 | 4 7 21 | mc7000670 | 10 11 12 13 21 |
| mc15000574 | 1 7 | mc7000671 | 4 7 13 21 |
| mc15000589 | 1 | mc7000672 | 1 4 7 10 |
| mc15000591 | 6 7 10 21 | mc7000675 | 9 |
| mc15000592 | 2 7 9 21 | mc7000677 | 6 7 |
| mc15000593 | 7 17 21 | mc7000678 | 9 21 |
| mc15000599 | 1 2 7 9 21 | mc7000679 | 6 10 21 |
| mc15000606 | 1 6 7 8 | mc7000680 | 7 9 21 |
| mc15000633 | 3 4 6 7 15 18 21 | mc7000686 | 21 |
| mc15000639 | 7 17 21 | mc7000707 | 21 |
| mc15000659 | 4 7 8 21 | mc7000710 | 7 |
| mc15000665 | 1 7 10 13 21 | mc7000711 | 2 6 7 8 9 |
| mc15000672 | 1 7 21 | mc7000713 | 1 2 15 21 |
| mc15000676 | 7 9 21 | mc7000719 | 9 |
| mc15000685 | 7 24 | mc7000721 | 1 6 21 |
| mc15000690 | 7 10 12 15 21 | mc7000727 | 6 7 |
| mc15000691 | 13 | mc7000730 | 4 7 18 21 |
| mc15000694 | 1 6 21 | mc7000739 | 7 10 |
| mc15000703 | 7 21 | mc7000742 | 9 |
| mc15000751 | 1 3 7 21 | mc7000745 | 21 |
| mc15000756 | 6 7 8 10 12 21 | mc7000746 | 6 7 8 12 13 21 |
| mc15000773 | 7 | mc7000749 | 15 |
| mc15000784 | 7 10 16 19 20 21 24 | mc7000769 | 4 7 21 |
| mc15000787 | 7 16 21 | mc7000792 | 6 7 21 |
| mc15000788 | 5 7 21 24 | mc7000795 | 1 3 6 7 8 21 |
| mc15000790 | 21 | mc7000798 | 7 |
| mc15000792 | 15 21 | mc7000800 | 4 7 17 21 |
| mc15000795 | 7 8 9 11 12 21 | mc7000853 | 7 12 |
| mc15000796 | 11 21 | mc7000854 | 7 |
| mc15000802 | 1 7 9 21 | mc7000856 | 7 17 20 24 |
| mc15000805 | 11 18 22 | mc7000857 | 21 |
| mc15000806 | 21 | mc7000858 | 1 2 6 9 21 |
| mc15000820 | 11 18 22 | mc7000863 | 7 12 21 |
| mc15000824 | 7 8 10 21 | mc7000868 | 7 10 12 21 |
| mc15000826 | 4 7 21 | mc7000870 | 6 |
| mc15000827 | 7 | mc7000872 | 6 21 |
| mc15000831 | 1 | mc7000873 | 6 21 |
| mc15000832 | 5 7 8 21 | mc7000874 | 21 |
| mc15000835 | 1 7 | mc7000876 | 6 21 |
| mc15000840 | 1 7 | mc7000879 | 6 21 |
| mc15000846 | 7 10 16 19 | mc7000881 | 6 |
| mc15000847 | 7 | mc7000882 | 6 |
| mc15000848 | 7 10 | mc7000891 | 6 21 |
| mc15000858 | 10 | mc7000906 | 7 15 21 |
| mc15000861 | 1 | mc7000909 | 7 9 19 21 22 24 |
| mc15000863 | 1 3 4 7 21 | mc7000910 | 21 |
| mc15000864 | 1 6 7 21 | mc7000911 | 1 |
| mc15000865 | 2 7 9 21 | mc7000918 | 3 7 21 |
| mc15000866 | 1 | mc7000919 | 1 4 7 10 21 |
| mc15000867 | 4 21 | mc7000920 | 6 21 |
| mc15000870 | 1 6 7 10 | mc7000921 | 9 12 21 |
| mc15000873 | 4 5 7 21 | mc7000923 | 1 2 7 9 13 15 21 |
| mc15000874 | 4 6 7 11 15 16 21 | mc7000927 | 1 6 7 21 |
| mc15000875 | 1 21 | mc7000929 | 4 7 15 21 |
| mc15000879 | 6 | mc7000930 | 7 9 21 |
| mc15000880 | 10 21 | mc7000931 | 2 9 |
| mc15000881 | 1 | mc7000932 | 8 |
| mc15000882 | 4 7 21 | mc7000933 | 10 |
| mc15000883 | 9 12 21 | mc7000934 | 1 3 7 11 21 |
| mc15000884 | 7 9 21 | mc7000938 | 6 7 9 17 21 |
| mc15000885 | 4 10 23 | mc7000940 | 1 2 21 24 |
| mc15000887 | 2 7 9 17 21 | mc7000941 | 1 6 7 16 21 |
| mc15000888 | 4 7 16 20 21 | mc7000942 | 15 |
| mc15000890 | 21 | mc7000943 | 4 6 7 13 21 |
| mc15000893 | 3 7 17 21 | mc7000945 | 3 7 10 |
| mc15000898 | 21 | mc7000947 | 7 21 |
| mc15000901 | 4 7 21 | mc7000948 | 1 21 24 |
| mc15000902 | 3 21 | mc7000950 | 7 10 13 21 |
| mc15000903 | 5 7 21 | mc7000951 | 5 7 21 |
| mc15000908 | 7 9 11 13 18 19 | mc7000952 | 5 21 |
| mc15000929 | 9 11 21 | mc7000953 | 15 18 19 21 |
| mc15000932 | 5 | mc7000954 | 1 |
| mc15000937 | 7 19 21 22 | mc7000958 | 1 7 21 |
| mc15000939 | 8 13 21 22 | mc7000960 | 21 |
| mc15000944 | 10 13 15 18 21 | mc7000961 | 4 7 21 |
| mc15000946 | 9 11 21 | mc7000962 | 21 |
| mc15000948 | 9 11 21 | mc7000964 | 9 10 21 |
| mc15000950 | 6 21 | mc7000967 | 7 21 |
| mc15000952 | 7 10 15 21 | mc7000970 | 7 9 10 17 21 |
| mc15000953 | 13 15 21 | mc7000971 | 4 7 21 |
| mc15000955 | 10 | mc7000973 | 7 9 21 |
| mc15000965 | 1 16 21 | mc7000974 | 7 13 15 16 18 19 21 |
| mc15000966 | 7 10 15 | mc7000976 | 10 21 |
| mc15000969 | 7 10 15 21 | mc7000977 | 1 3 4 7 9 21 |
| mc15000970 | 7 | mc7000978 | 1 19 20 |
| mc15000971 | 6 7 9 21 | mc7000981 | 7 10 15 18 21 |
| mc15000973 | 7 8 21 | mc7000982 | 1 2 9 11 |
| mc15000974 | 1 7 | mc7000983 | 7 10 12 13 21 |
| mc15000975 | 7 16 19 21 | mc7000985 | 1 |
| mc15000977 | 7 21 | mc7000987 | 7 |
| mc15000979 | 1 7 21 | mc7000990 | 1 7 21 |
| mc15000980 | 10 | mc7000991 | 15 21 |
| mc15000981 | 4 7 | mc7000992 | 1 21 |
| mc15000982 | 5 6 7 11 21 | mc7001000 | 1 6 21 |
| mc15000989 | 7 9 10 21 | mc7001002 | 13 21 |
| mc15000990 | 9 | mc7001003 | 4 7 12 21 |
| mc15000993 | 1 | mc7001007 | 16 21 23 24 |
| mc15000997 | 1 6 7 10 12 | mc7001009 | 1 6 |
| mc15000999 | 7 9 12 21 24 | mc7001011 | 7 8 12 21 |
| mc15001001 | 9 10 21 | mc7001012 | 7 9 11 12 21 22 24 |
| mc15001005 | 1 3 7 20 21 | mc7001013 | 21 |
| mc15001007 | 21 | mc7001014 | 7 9 10 12 21 |
| mc15001008 | 6 7 9 21 | mc7001018 | 15 |
| mc15001011 | 21 | mc7001022 | 7 9 11 21 23 24 |
| mc15001012 | 2 7 9 17 21 | mc7001027 | 1 7 13 21 |
| mc15001015 | 3 4 7 21 | mc7001028 | 4 7 15 16 19 21 |
| mc15001017 | 1 4 7 21 | mc7001032 | 8 21 |
| mc15001018 | 3 4 7 15 17 21 | mc7001038 | 9 13 21 |
| mc15001021 | 3 6 12 15 17 21 | mc7001039 | 9 13 21 |
| mc15001023 | 5 7 21 | mc7001043 | 3 4 7 21 |
| mc15001025 | 6 9 21 | mc7001047 | 3 4 6 7 8 9 15 18 20 21 |
| mc15001026 | 7 8 10 | mc7001049 | 21 |
| mc15001028 | 1 6 21 | mc7001055 | 1 6 8 10 |
| mc15001031 | 2 4 7 21 | mc7001060 | 10 |
| mc15001037 | 7 10 | mc7001072 | 10 21 |
| mc15001043 | 7 10 | mc7001081 | 1 7 |
| mc15001044 | 1 21 | mc7001082 | 7 15 16 19 21 23 |
| mc15001046 | 4 7 15 21 | mc7001093 | 4 7 21 |
| mc15001050 | 10 | mc7001094 | 1 6 7 21 |
| mc15001051 | 9 21 | mc7001096 | 9 21 22 23 24 |
| mc15001052 | 6 7 11 | mc7001123 | 3 15 21 |
| mc15001053 | 6 21 | mc7001135 | 5 21 |
| mc15001056 | 6 7 21 | mc7001147 | 16 21 |
| mc15001060 | 3 4 5 7 8 21 | mc7001148 | 9 12 13 18 21 22 |
| mc15001062 | 8 21 | mc7001167 | 1 7 9 |
| mc15001066 | 4 7 12 21 | mc7001173 | 6 7 21 |
| mc15001068 | 3 7 21 | mc7001180 | 4 7 21 |
| mc15001070 | 4 7 21 | mc7001181 | 21 |
| mc15001071 | 1 2 7 21 | mc7001198 | 4 7 10 12 13 15 16 20 21 |
| mc15001073 | 4 7 21 | mc7001200 | 4 7 |
| mc15001074 | 1 21 | mc7001201 | 6 7 21 |
| mc15001077 | 3 7 21 | mc7001208 | 7 9 10 12 21 |
| mc15001078 | 4 5 7 16 21 | mc7001220 | 4 7 21 |
| mc15001086 | 10 13 15 18 21 | mc7001230 | 4 5 7 21 |
| mc15001091 | 3 7 | mc7001238 | 7 9 |
| mc15001093 | 1 2 11 21 | mc7001241 | 4 7 15 21 |
| mc15001096 | 2 7 9 21 | mc7001246 | 7 17 21 |
| mc15001099 | 7 9 21 | mc7001251 | 5 7 |
| mc15001114 | 4 7 21 | mc7001258 | 7 21 |
| mc15001120 | 1 21 | mc7001259 | 7 9 21 |
| mc15001122 | 7 21 | mc7001269 | 7 10 |
| mc15001123 | 1 7 9 21 | mc7001282 | 4 7 16 18 19 21 22 23 |
| mc15001126 | 7 8 9 | mc7001296 | 6 7 10 16 20 21 |
| mc15001131 | 6 | mc7001372 | 16 21 |
| mc15001132 | 7 15 21 | mc7001380 | 1 6 21 |
| mc15001134 | 9 21 | mc7001385 | 9 |
| mc15001135 | 15 21 | mc7001392 | 9 12 21 |
| mc15001138 | 7 | mc7001421 | 1 7 10 18 21 |
| mc15001141 | 1 6 21 | mc7001426 | 5 21 |
| mc15001143 | 1 21 23 | mc7001427 | 5 21 |
| mc15001145 | 21 | mc7001428 | 6 21 |
| mc15001147 | 7 12 21 | mc7001430 | 1 3 21 |
| mc15001168 | 7 9 16 21 | mc7001431 | 1 3 7 21 |
| mc15001173 | 21 | mc7001433 | 7 19 21 23 |
| mc15001175 | 3 7 21 | mc7001434 | 7 12 21 |
| mc15001177 | 7 21 | mc7001435 | 7 8 9 10 12 13 16 21 |
| mc15001179 | 7 | mc7001437 | 11 12 |
| mc15001181 | 7 10 15 16 18 21 | mc7001438 | 7 9 12 13 |
| mc15001186 | 1 4 7 10 13 17 21 | mc7001440 | 3 7 21 |
| mc15001188 | 7 9 10 21 | mc7001446 | 9 21 |
| mc15001190 | 21 | mc7001451 | 1 7 |
| mc15001191 | 4 5 21 | mc7001452 | 17 21 |
| mc15001192 | 7 10 12 16 18 19 21 | mc7001457 | 6 7 15 20 |
| mc15001194 | 1 7 8 | mc7001465 | 1 7 9 11 21 |
| mc15001198 | 8 | mc7001466 | 10 15 16 |
| mc15001227 | 1 6 21 | mc7001467 | 7 8 21 |
| mc15001228 | 7 | mc7001469 | 21 |
| mc15001229 | 1 6 7 | mc7001470 | 6 9 17 21 |
| mc15001231 | 9 | mc7001471 | 3 21 |
| mc15001232 | 3 4 7 16 | mc7001478 | 1 3 6 7 10 |
| mc15001235 | 1 7 21 | mc7001482 | 3 7 21 |
| mc15001237 | 3 4 6 7 21 | mc7001485 | 7 8 10 21 |
| mc15001238 | 1 3 6 7 10 | mc7001487 | 4 7 15 16 21 |
| mc15001249 | 13 21 | mc7001489 | 10 21 |
| mc15001250 | 1 21 | mc7001493 | 1 6 7 19 20 21 |
| mc15001253 | 1 17 21 | mc7001495 | 1 7 10 |
| mc15001258 | 1 7 | mc7001497 | 5 7 21 |
| mc15001260 | 1 7 10 | mc7001498 | 3 4 5 7 |
| mc15001261 | 1 7 10 16 21 | mc7001503 | 10 |
| mc15001263 | 7 10 12 17 18 19 22 23 24 | mc7001511 | 7 21 23 |
| mc15001264 | 6 7 16 18 | mc7001514 | 3 15 21 |
| mc15001265 | 7 | mc7001515 | 4 7 21 |
| mc15001267 | 7 9 21 | mc7001517 | 7 21 |
| mc15001277 | 2 7 9 17 21 | mc7001523 | 5 |
| mc15001280 | 1 7 9 21 | mc7001535 | 1 7 9 |
| mc15001292 | 1 6 7 | mc7001536 | 7 12 13 15 21 |
| mc15001303 | 1 3 7 10 12 21 | mc7001549 | 4 7 8 20 21 24 |
| mc15001324 | 4 7 21 | mc7001554 | 1 4 7 21 |
| mc15001334 | 6 7 21 | mc7001555 | 7 10 |
| mc15001340 | 1 6 7 10 13 15 | mc7001591 | 1 6 7 21 |
| mc15001341 | 1 7 21 | mc7001597 | 2 9 11 |
| mc15001356 | 7 12 21 | mc7001598 | 1 7 21 |
| mc15001379 | 9 13 21 22 | mc7001603 | 6 21 |
| mc15001386 | 9 13 | mc7001611 | 7 10 16 21 |
| mc15001391 | 7 12 15 18 21 22 | mc7001613 | 6 21 |
| mc15001400 | 1 6 7 8 10 | mc7001618 | 1 2 7 21 |
| mc15001403 | 4 6 7 8 21 | mc7001621 | 4 7 16 21 |
| mc15001406 | 4 7 9 10 12 13 15 16 20 21 | mc7001624 | 6 7 9 21 |
| mc15001411 | 6 21 | mc7001627 | 7 8 9 |
| mc15001414 | 4 7 10 | mc7001630 | 4 7 21 |
| mc15001419 | 7 21 | mc7001646 | 7 12 13 |
| mc15001429 | 1 7 21 | mc7001651 | 4 7 |
| mc15001432 | 3 4 7 8 21 | mc7001655 | 7 9 10 21 |
| mc15001434 | 1 7 10 21 | mc7001657 | 6 21 |
| mc15001440 | 6 21 | mc7001685 | 16 |
| mc15001443 | 7 10 15 16 20 21 | mc7001688 | 5 7 21 |
| mc15001448 | 7 9 | mc7001689 | 7 10 12 |
| mc15001450 | 6 7 10 18 19 20 21 | mc7001697 | 21 |
| mc15001451 | 10 | mc7001704 | 7 9 21 |
| mc15001452 | 7 12 17 21 | mc7001705 | 6 9 |
| mc15001459 | 7 21 22 | mc7001707 | 1 6 7 10 16 21 22 |
| mc15001463 | 6 7 11 | mc7001709 | 2 7 9 13 17 21 |
| mc15001464 | 21 | mc7001710 | 6 7 10 24 |
| mc15001465 | 7 9 10 21 | mc7001711 | 7 10 12 13 21 |
| mc15001466 | 21 | mc7001714 | 1 21 |
| mc15001469 | 7 | mc7001721 | 3 4 11 21 |
| mc15001470 | 15 21 | mc7001723 | 5 7 10 11 15 21 |
| mc15001473 | 15 | mc7001729 | 3 21 |
| mc15001475 | 9 21 | mc7001731 | 1 21 24 |
| mc15001477 | 9 21 | mc7001733 | 1 24 |
| mc15001478 | 2 7 9 10 | mc7001735 | 7 17 21 |
| mc15001480 | 7 9 11 13 21 | mc7001736 | 6 7 8 11 12 21 |
| mc15001481 | 3 4 7 17 21 | mc7001740 | 3 5 15 21 |
| mc15001482 | 1 21 | mc7001746 | 1 11 21 |
| mc15001485 | 1 4 7 21 | mc7001747 | 6 7 21 |
| mc15001486 | 7 21 | mc7001754 | 7 10 15 16 20 21 |
| mc15001496 | 6 | mc7001755 | 1 3 7 21 |
| mc15001499 | 7 9 21 | mc7001756 | 1 6 |
| mc15001502 | 10 21 | mc7001757 | 7 9 |
| mc15001503 | 15 21 | mc7001759 | 1 2 |
| mc15001506 | 4 7 21 | mc7001761 | 7 21 |
| mc15001508 | 4 7 21 | mc7001762 | 1 6 |
| mc15001510 | 6 | mc7001766 | 2 9 13 21 23 |
| mc15001516 | 7 9 21 | mc7001772 | 7 9 10 21 |
| mc15001519 | 4 7 | mc7001775 | 7 21 |
| mc15001520 | 1 6 7 8 10 21 | mc7001778 | 10 15 |
| mc15001521 | 1 6 7 8 10 21 | mc7001780 | 7 9 10 |
| mc15001522 | 7 9 10 21 | mc7001781 | 10 21 |
| mc15001525 | 4 7 10 21 | mc7001785 | 9 |
| mc15001537 | 7 17 20 21 24 | mc7001788 | 7 8 10 21 |
| mc15001548 | 7 17 | mc7001793 | 1 6 21 |
| mc15001550 | 7 17 20 | mc7001795 | 1 4 7 |
| mc15001561 | 7 17 | mc7001797 | 4 7 16 21 |
| mc15001562 | 7 17 18 19 20 21 22 23 24 | mc7001798 | 1 4 7 10 21 |
| mc15001564 | 5 7 | mc7001800 | 1 6 7 9 10 21 |
| mc15001565 | 7 | mc7001808 | 3 7 21 |
| mc15001567 | 7 15 21 | mc7001811 | 9 10 12 21 |
| mc15001569 | 1 6 21 | mc7001812 | 1 6 21 |
| mc15001570 | 1 7 | mc7001813 | 1 6 |
| mc15001573 | 7 10 12 | mc7001819 | 7 12 21 |
| mc15001574 | 4 7 10 16 21 | mc7001820 | 1 3 7 21 |
| mc15001578 | 6 7 17 21 | mc7001823 | 10 13 |
| mc15001579 | 21 | mc7001828 | 7 21 |
| mc15001580 | 9 21 | mc7001839 | 10 13 |
| mc15001581 | 4 7 18 19 21 | mc7001840 | 10 13 |
| mc15001583 | 4 7 21 | mc7001843 | 10 13 21 |
| mc15001584 | 10 16 19 20 21 23 24 | mc7001849 | 10 13 |
| mc15001587 | 1 6 7 10 | mc7001853 | 10 13 |
| mc15001588 | 7 21 | mc7001854 | 10 13 |
| mc15001589 | 12 13 21 24 | mc7001856 | 10 13 |
| mc15001590 | 2 4 7 9 21 | mc7001860 | 10 13 |
| mc15001593 | 4 7 8 21 | mc7001884 | 6 |
| mc15001600 | 4 7 15 16 21 | mc7001913 | 7 9 13 19 21 |
| mc15001601 | 4 7 15 16 21 | mc7001922 | 10 13 21 |
| mc15001604 | 4 7 15 16 21 | mc7001924 | 10 13 |
| mc15001605 | 4 7 15 16 21 | mc7001928 | 10 13 |
| mc15001608 | 4 7 15 16 21 | mc7001933 | 10 13 |
| mc15001611 | 4 7 15 16 21 | mc7001937 | 10 13 |
| mc15001614 | 1 3 7 11 21 | mc7001955 | 4 6 7 21 |
| mc15001619 | 4 7 8 13 16 21 | mc7002012 | 10 12 13 21 24 |
| mc15001621 | 10 | mc7002014 | 1 |
| mc15001623 | 10 12 17 18 21 22 | mc7002017 | 1 3 11 21 |
| mc15001628 | 1 7 15 21 | mc7002018 | 4 6 7 10 21 |
| mc15001629 | 1 2 8 10 | mc7002019 | 7 9 21 |
| mc15001630 | 7 12 21 | mc7002022 | 7 10 12 |
| mc15001636 | 7 9 21 | mc7002028 | 21 |
| mc15001649 | 7 21 | mc7002029 | 1 6 7 10 12 15 16 19 20 21 |
| mc15001650 | 4 7 16 21 | mc7002030 | 3 4 7 15 21 |
| mc15001652 | 7 21 | mc7002031 | 6 21 |
| mc15001653 | 2 7 9 10 12 15 17 18 21 | mc7002034 | 5 21 |
| mc15001654 | 21 | mc7002048 | 10 13 |
| mc15001657 | 19 21 | mc7002062 | 10 13 |
| mc15001661 | 1 21 | mc7002067 | 10 13 |
| mc15001663 | 17 21 | mc7002076 | 3 7 10 |
| mc16000003 | 7 13 21 | mc7002077 | 7 9 21 |
| mc16000004 | 4 7 21 | mc7002087 | 10 12 13 |
| mc16000006 | 10 12 13 21 | mc7002121 | 10 13 |
| mc16000010 | 1 6 | mc7002123 | 7 11 |
| mc16000016 | 1 3 7 15 21 | mc7002124 | 5 |
| mc16000017 | 6 7 11 21 | mc7002126 | 10 12 13 20 21 |
| mc16000021 | 1 7 10 21 24 | mc7002129 | 3 4 7 16 21 |
| mc16000025 | 7 21 | mc7002135 | 4 7 21 |
| mc16000026 | 4 7 21 | mc7002137 | 5 7 21 |
| mc16000028 | 4 7 21 | mc7002143 | 4 7 16 21 |
| mc16000029 | 18 21 | mc7002146 | 7 21 |
| mc16000030 | 9 21 | mc7002150 | 7 8 |
| mc16000033 | 6 7 10 11 15 21 | mc7002152 | 6 7 9 21 |
| mc16000036 | 1 7 21 | mc7002154 | 4 7 21 |
| mc16000040 | 6 7 | mc7002155 | 1 3 6 7 10 21 |
| mc16000047 | 15 19 | mc7002156 | 3 7 13 21 |
| mc16000051 | 7 19 21 | mc7002164 | 13 21 24 |
| mc16000053 | 1 6 21 | mc7002167 | 1 |
| mc16000056 | 1 7 21 | mc7002169 | 4 6 7 21 |
| mc16000068 | 4 7 | mc7002170 | 1 7 10 12 21 |
| mc16000096 | 1 7 21 | mc7002171 | 7 12 21 |
| mc16000101 | 4 7 10 21 | mc7002172 | 7 21 |
| mc16000114 | 7 9 10 12 21 22 | mc7002173 | 5 7 21 |
| mc16000118 | 5 7 21 | mc7002182 | 1 7 21 |
| mc16000127 | 7 9 | mc7002190 | 10 16 21 |
| mc16000131 | 4 7 21 | mc7002192 | 1 2 7 9 21 |
| mc16000134 | 6 7 8 10 18 | mc7002195 | 7 12 21 |
| mc16000135 | 7 15 16 17 21 | mc7002208 | 4 7 8 9 13 21 |
| mc16000136 | 3 22 | mc7002211 | 10 12 21 |
| mc16000142 | 4 7 15 | mc7002219 | 7 12 16 21 |
| mc16000144 | 11 | mc7002224 | 7 9 17 21 |
| mc16000146 | 2 9 21 | mc7002225 | 6 7 8 11 13 21 |
| mc16000150 | 5 7 21 | mc7002229 | 1 9 10 |
| mc16000151 | 4 5 7 21 | mc7002230 | 1 7 9 21 |
| mc16000152 | 10 13 | mc7002234 | 7 8 10 13 |
| mc16000174 | 1 3 7 11 21 | mc7002237 | 7 10 13 |
| mc16000180 | 1 3 4 7 21 | mc7002246 | 4 7 8 18 19 21 |
| mc16000182 | 7 15 | mc7002263 | 5 7 21 |
| mc16000184 | 1 7 21 | mc7002264 | 1 7 10 21 |
| mc16000187 | 6 13 21 | mc7002267 | 7 12 21 |
| mc16000201 | 3 7 9 16 17 20 21 | mc7002278 | 1 6 21 |
| mc16000206 | 4 7 11 15 16 21 | mc7002284 | 5 6 7 9 21 |
| mc16000217 | 3 4 7 21 | mc7002289 | 21 |
| mc16000218 | 1 3 6 7 11 15 17 21 | mc7002292 | 1 21 |
| mc16000225 | 12 19 20 21 | mc7002296 | 1 7 10 21 |
| mc16000227 | 5 7 21 | mc7002302 | 10 21 |
| mc16000228 | 7 | mc7002307 | 21 |
| mc16000232 | 7 8 10 15 21 | mc7002308 | 7 21 |
| mc16000236 | 7 12 16 21 | mc7002311 | 1 |
| mc16000242 | 1 6 7 10 15 | mc7002313 | 1 2 21 |
| mc16000246 | 21 | mc7002314 | 1 |
| mc16000247 | 6 21 | mc7002319 | 1 3 21 |
| mc16000249 | 21 | mc7002322 | 2 7 9 21 |
| mc16000253 | 6 7 21 | mc7002325 | 7 10 16 21 |
| mc16000255 | 4 7 21 | mc7002326 | 7 21 |
| mc16000260 | 7 8 13 21 | mc7002327 | 20 21 24 |
| mc16000262 | 7 9 21 | mc7002338 | 2 7 9 21 |
| mc16000265 | 2 9 21 | mc7002344 | 1 5 6 7 |
| mc16000266 | 7 | mc7002345 | 4 21 |
| mc16000267 | 4 7 21 | mc7002346 | 21 |
| mc16000269 | 7 9 10 21 | mc7002351 | 21 |
| mc16000270 | 9 | mc7002366 | 7 9 12 13 |
| mc16000273 | 4 21 | mc7002368 | 7 9 12 13 |
| mc16000277 | 7 10 12 | mc7002371 | 5 7 |
| mc16000282 | 1 6 7 10 15 16 | mc7002373 | 1 7 21 |
| mc16000284 | 21 | mc7002376 | 3 21 |
| mc16000286 | 9 21 | mc7002377 | 1 3 6 7 10 21 |
| mc16000289 | 21 | mc7002378 | 4 6 7 11 15 21 |
| mc16000292 | 1 21 | mc7002380 | 1 6 7 9 10 12 21 |
| mc16000294 | 7 10 16 21 | mc7002386 | 7 |
| mc16000300 | 3 8 9 21 | mc7002393 | 9 |
| mc16000301 | 1 7 | mc7002394 | 9 17 21 |
| mc16000303 | 4 7 21 | mc7002397 | 9 21 |
| mc16000307 | 7 12 21 | mc7002422 | 10 13 21 |
| mc16000308 | 1 7 21 | mc7002424 | 1 10 |
| mc16000309 | 7 9 11 21 | mc7002425 | 4 7 21 |
| mc16000311 | 4 7 12 15 16 19 20 21 | mc7002432 | 6 7 9 21 |
| mc16000314 | 7 12 13 21 | mc7002434 | 1 6 7 10 |
| mc16000319 | 7 12 17 21 | mc7002436 | 10 13 21 |
| mc16000320 | 3 7 21 | mc7002437 | 9 |
| mc16000321 | 6 | mc7002438 | 4 7 13 15 21 |
| mc16000323 | 5 21 | mc7002439 | 21 |
| mc16000324 | 4 7 9 16 21 | mc7002441 | 8 9 15 |
| mc16000334 | 10 13 | mc7002446 | 5 7 21 |
| mc16000337 | 10 13 | mc7002452 | 7 10 |
| mc16000344 | 21 | mc7002453 | 7 10 21 |
| mc16000346 | 1 6 | mc7002454 | 21 |
| mc16000351 | 7 | mc7002458 | 1 6 7 |
| mc16000360 | 1 7 9 | mc7002461 | 4 |
| mc16000361 | 5 7 8 | mc7002464 | 3 6 7 10 13 20 21 |
| mc16000363 | 6 7 9 17 21 | mc7002465 | 1 |
| mc16000364 | 7 | mc7002468 | 4 7 15 16 21 |
| mc16000366 | 1 6 21 | mc7002474 | 7 9 10 12 18 21 24 |
| mc16000369 | 6 7 21 | mc7002479 | 7 9 21 |
| mc16000370 | 6 7 8 11 21 | mc7002482 | 1 7 21 |
| mc16000375 | 9 21 | mc7002484 | 7 |
| mc16000376 | 4 7 21 | mc7002487 | 2 7 9 17 21 |
| mc16000377 | 3 10 15 19 21 | mc7002488 | 7 |
| mc16000378 | 7 10 16 18 19 20 21 | mc7002490 | 1 12 |
| mc16000390 | 6 7 8 10 15 21 22 | mc7002491 | 7 12 13 15 21 22 |
| mc16000400 | 1 2 21 | mc7002494 | 1 |
| mc16000408 | 1 21 | mc7002500 | 1 7 |
| mc16000409 | 6 10 21 | mc7002503 | 7 21 |
| mc16000416 | 4 7 21 | mc7002521 | 1 2 6 7 |
| mc16000418 | 4 7 15 21 | mc7002523 | 7 |
| mc16000419 | 1 7 8 10 | mc7002529 | 15 21 |
| mc16000421 | 15 19 20 21 24 | mc7002530 | 15 16 21 |
| mc16000423 | 4 6 7 11 21 | mc7002532 | 4 6 7 |
| mc16000424 | 8 16 21 | mc7002534 | 1 21 |
| mc16000425 | 8 21 | mc7002536 | 7 9 21 |
| mc16000426 | 8 21 | mc7002540 | 1 21 |
| mc16000431 | 8 13 20 21 | mc7002541 | 1 6 21 |
| mc16000434 | 3 7 21 | mc7002542 | 1 3 4 6 7 21 |
| mc16000435 | 1 2 7 10 12 13 21 23 | mc7002543 | 6 10 20 21 23 24 |
| mc16000440 | 1 6 21 | mc7002545 | 4 7 21 |
| mc16000443 | 6 7 13 21 | mc7002547 | 3 4 7 8 13 15 21 |
| mc16000451 | 10 21 | mc7002552 | 7 10 12 13 21 |
| mc16000453 | 4 7 15 18 21 | mc7002554 | 7 10 12 21 |
| mc16000462 | 7 12 21 | mc7002558 | 2 7 9 21 24 |
| mc16000473 | 4 7 10 15 16 17 18 19 20 21 | mc7002561 | 4 7 8 10 12 21 |
| mc16000474 | 7 21 | mc7002562 | 7 9 |
| mc16000477 | 7 12 17 21 23 | mc7002566 | 10 |
| mc16000482 | 10 15 | mc7002567 | 7 15 21 |
| mc16000488 | 10 15 16 18 19 20 21 | mc7002569 | 6 7 8 10 11 15 21 |
| mc16000490 | 10 21 | mc7002596 | 1 6 8 21 |
| mc16000506 | 10 15 21 | mc7002599 | 7 21 |
| mc16000507 | 1 7 9 | mc7002604 | 6 10 15 18 21 |
| mc16000510 | 7 13 17 21 22 | mc7002605 | 16 19 21 |
| mc16000520 | 4 7 12 15 18 21 | mc7002607 | 10 21 |
| mc16000530 | 7 12 13 21 | mc7002608 | 21 |
| mc16000544 | 1 2 8 | mc7002610 | 1 5 7 |
| mc16000546 | 7 9 21 | mc7002611 | 7 21 |
| mc16000553 | 6 7 9 24 | mc7002612 | 1 2 7 9 21 |
| mc16000554 | 7 9 12 21 | mc7002614 | 4 7 16 20 21 |
| mc16000558 | 4 7 9 21 | mc7002616 | 21 |
| mc16000559 | 6 | mc7002623 | 6 10 |
| mc16000567 | 6 21 | mc7002625 | 6 |
| mc16000571 | 7 21 | mc7002630 | 1 7 21 |
| mc16000582 | 6 9 10 12 21 22 | mc7002631 | 4 7 16 19 20 21 23 24 |
| mc16000583 | 1 6 7 10 22 | mc7002639 | 21 |
| mc16000586 | 7 10 | mc7002652 | 6 7 21 |
| mc16000596 | 5 21 | mc7002653 | 1 |
| mc16000601 | 10 | mc7002654 | 3 7 21 |
| mc16000602 | 4 7 16 21 | mc7002655 | 3 4 7 |
| mc16000607 | 11 21 | mc7002656 | 4 7 |
| mc16000608 | 7 10 12 21 | mc7002657 | 6 7 12 16 21 |
| mc16000609 | 1 | mc7002677 | 1 6 7 8 10 21 |
| mc16000616 | 6 7 23 | mc7002698 | 1 3 11 21 |
| mc16000620 | 1 6 7 17 21 | mc7002707 | 7 8 |
| mc16000621 | 1 6 | mc7002722 | 1 6 7 8 10 |
| mc16000625 | 9 21 | mc7002728 | 1 6 7 |
| mc16000626 | 6 21 | mc7002738 | 1 10 |
| mc16000631 | 10 15 16 21 | mc7002741 | 4 7 15 18 21 |
| mc16000632 | 6 | mc7002757 | 4 7 21 |
| mc16000634 | 7 11 24 | mc7002759 | 6 7 10 12 13 21 23 |
| mc16000641 | 7 9 12 21 | mc7002760 | 17 21 |
| mc16000642 | 6 7 9 21 | mc7002762 | 4 7 16 21 |
| mc16000643 | 10 | mc7002764 | 7 10 12 21 |
| mc16000658 | 8 | mc7002765 | 21 |
| mc16000660 | 10 12 16 19 20 21 23 24 | mc7002767 | 1 2 21 |
| mc16000662 | 13 18 21 22 | mc7002768 | 1 2 9 21 |
| mc16000664 | 10 21 | mc7002797 | 7 9 21 |
| mc16000666 | 9 21 | mc7002802 | 24 |
| mc16000667 | 4 7 15 21 | mc7002804 | 10 |
| mc16000668 | 7 | mc7002805 | 7 21 |
| mc16000671 | 7 21 22 | mc7002806 | 6 7 8 11 21 |
| mc16000673 | 3 | mc7002807 | 2 7 9 21 24 |
| mc16000675 | 9 | mc7002811 | 16 |
| mc16000677 | 4 7 21 | mc7002812 | 12 |
| mc16000679 | 1 7 | mc7002818 | 7 12 21 |
| mc16000680 | 2 7 9 21 | mc7002819 | 7 10 |
| mc16000683 | 7 21 | mc7002821 | 1 |
| mc16000685 | 10 | mc7002827 | 10 |
| mc16000687 | 10 12 21 | mc7002831 | 4 7 12 13 21 |
| mc16000689 | 6 7 8 10 15 19 21 | mc7002832 | 7 12 |
| mc16000690 | 1 4 7 10 21 | mc7002833 | 10 21 |
| mc16000695 | 19 20 21 23 24 | mc7002834 | 10 |
| mc16000696 | 1 21 | mc7002835 | 4 7 21 |
| mc16000697 | 6 7 | mc7002837 | 10 |
| mc16000699 | 10 11 21 | mc7002839 | 1 2 21 |
| mc16000706 | 7 8 10 | mc7002843 | 9 21 |
| mc16000708 | 1 7 21 | mc7002844 | 15 |
| mc16000709 | 21 23 24 | mc7002848 | 21 |
| mc16000711 | 21 | mc7002851 | 1 11 21 |
| mc16000730 | 7 12 | mc7002861 | 9 21 |
| mc16000742 | 7 15 19 21 23 | mc7002862 | 10 13 |
| mc16000747 | 2 7 9 21 | mc7002867 | 7 21 |
| mc16000756 | 10 13 21 24 | mc7002869 | 1 6 8 10 21 |
| mc16000763 | 7 9 12 13 21 | mc7002878 | 6 11 12 21 |
| mc16000765 | 1 6 | mc7002882 | 6 15 21 |
| mc16000770 | 7 12 19 21 | mc7002884 | 7 9 13 17 19 21 |
| mc16000777 | 7 10 21 | mc7002886 | 7 10 11 12 13 15 17 |
| mc16000782 | 7 10 21 | mc7002889 | 5 21 |
| mc16000785 | 6 9 | mc7002893 | 3 7 10 15 21 |
| mc16000787 | 7 10 13 18 21 | mc7002896 | 1 4 7 9 10 11 15 21 |
| mc16000788 | 7 | mc7002900 | 1 7 12 13 15 19 20 21 |
| mc16000795 | 6 | mc7002903 | 7 9 21 |
| mc16000806 | 12 13 21 22 | mc7002905 | 7 8 9 10 11 12 13 15 18 21 22 |
| mc16000811 | 7 10 12 21 | mc7002906 | 15 |
| mc16000830 | 7 16 21 | mc7002907 | 9 10 |
| mc16000831 | 10 21 | mc7002908 | 3 4 7 9 21 |
| mc16000833 | 10 21 | mc7002913 | 1 3 4 7 8 16 21 |
| mc16000834 | 10 21 | mc7002914 | 2 7 9 21 |
| mc16000847 | 7 15 21 | mc7002915 | 15 21 |
| mc16000849 | 7 10 12 13 21 | mc7002917 | 5 7 21 |
| mc16000857 | 4 7 | mc7002918 | 10 |
| mc16000872 | 7 12 | mc7002919 | 7 10 |
| mc16000893 | 4 7 13 21 | mc7002921 | 1 9 |
| mc16000902 | 1 | mc7002923 | 10 |
| mc16000905 | 7 10 21 | mc7002924 | 1 |
| mc16000907 | 21 | mc7002925 | 1 15 20 21 |
| mc16000909 | 15 | mc7002936 | 7 10 15 |
| mc16000910 | 10 | mc7002941 | 3 7 10 15 20 21 |
| mc16000911 | 21 | mc7002943 | 3 7 10 15 16 18 20 21 |
| mc16000915 | 6 7 9 21 | mc7002944 | 7 10 12 15 16 19 20 21 |
| mc16000916 | 1 21 | mc7002946 | 3 10 21 |
| mc16000925 | 7 9 12 19 21 | mc7002951 | 9 |
| mc16000936 | 12 | mc7002953 | 10 |
| mc16000942 | 1 7 21 | mc7002954 | 10 |
| mc16000971 | 21 | mc7002955 | 7 |
| mc16000975 | 6 7 10 21 | mc7002965 | 10 |
| mc16000976 | 1 6 7 8 10 21 24 | mc7002966 | 7 21 |
| mc16000992 | 7 | mc7002967 | 7 21 |
| mc16000995 | 7 13 21 | mc7002972 | 6 7 21 |
| mc16001009 | 7 21 | mc7002979 | 9 21 |
| mc16001012 | 10 | mc7002980 | 21 |
| mc16001016 | 9 | mc7002982 | 7 21 |
| mc16001023 | 7 12 | mc7002983 | 7 12 13 17 19 21 23 |
| mc16001038 | 1 2 | mc7002984 | 1 7 8 |
| mc16001050 | 10 12 13 15 16 21 | mc7002985 | 7 10 17 21 |
| mc16001068 | 1 21 | mc7002988 | 10 |
| mc16001070 | 7 | mc7002994 | 7 21 23 |
| mc16001071 | 6 7 21 | mc7003001 | 6 7 11 |
| mc16001085 | 6 7 21 | mc7003009 | 7 |
| mc16001098 | 7 10 12 17 19 20 | mc7003016 | 1 21 |
| mc16001099 | 3 7 21 | mc7003019 | 1 21 |
| mc16001103 | 7 | mc7003038 | 6 7 |
| mc16001114 | 7 12 21 | mc7003044 | 4 7 |
| mc16001128 | 5 21 | mc8000002 | 6 7 10 16 20 21 |
| mc16001129 | 17 21 | mc8000006 | 17 21 |
| mc16001130 | 2 7 9 21 | mc8000009 | 7 9 21 24 |
| mc16001131 | 4 7 21 | mc8000010 | 1 15 20 |
| mc16001133 | 4 7 8 11 12 13 15 16 19 21 24 | mc8000014 | 3 4 7 11 21 |
| mc16001142 | 6 7 21 | mc8000015 | 8 10 |
| mc16001143 | 6 7 10 21 | mc8000017 | 10 21 |
| mc16001152 | 1 21 | mc8000019 | 9 21 |
| mc16001156 | 6 7 11 21 | mc8000020 | 7 10 11 21 |
| mc16001163 | 4 7 8 21 | mc8000022 | 7 9 10 |
| mc16001174 | 7 17 | mc8000025 | 7 9 10 |
| mc16001176 | 7 17 21 | mc8000026 | 7 9 10 12 |
| mc16001183 | 21 | mc8000027 | 7 8 9 10 12 13 15 21 |
| mc16001191 | 21 | mc8000038 | 6 7 8 10 11 |
| mc16001223 | 7 8 10 16 21 | mc8000040 | 4 7 8 21 |
| mc16001230 | 7 10 11 12 15 21 | mc8000042 | 6 7 9 21 |
| mc16001235 | 1 6 7 10 | mc8000046 | 7 10 12 13 15 21 24 |
| mc16001241 | 21 | mc8000048 | 1 4 7 21 |
| mc16001253 | 4 7 16 21 | mc8000054 | 7 |
| mc16001254 | 4 7 16 21 | mc8000058 | 7 9 21 |
| mc16001257 | 3 4 10 21 | mc8000089 | 7 15 16 20 21 |
| mc16001258 | 7 10 13 21 | mc8000115 | 3 7 11 21 |
| mc16001259 | 7 10 13 21 22 | mc8000117 | 7 13 15 18 21 |
| mc16001263 | 10 21 | mc8000140 | 7 9 12 17 21 |
| mc16001265 | 6 7 11 | mc8000142 | 7 9 12 17 21 |
| mc16001266 | 1 | mc8000143 | 7 9 10 |
| mc16001267 | 7 21 | mc8000144 | 1 |
| mc16001270 | 7 | mc8000147 | 3 4 7 21 |
| mc16001272 | 7 9 10 | mc8000156 | 7 8 10 |
| mc16001274 | 2 7 9 21 | mc8000174 | 17 21 |
| mc16001276 | 5 21 | mc8000176 | 1 7 9 |
| mc16001281 | 7 9 | mc8000182 | 7 8 10 |
| mc16001282 | 21 | mc8000183 | 1 6 7 13 21 |
| mc16001285 | 7 9 21 | mc8000184 | 6 7 13 21 |
| mc16001287 | 4 10 11 13 16 21 | mc8000185 | 6 7 13 21 |
| mc16001291 | 9 | mc8000188 | 21 |
| mc16001293 | 4 7 18 20 21 | mc8000189 | 10 21 |
| mc16001312 | 1 7 21 | mc8000193 | 9 12 |
| mc16001321 | 3 4 7 10 11 21 | mc8000195 | 3 4 7 15 19 21 |
| mc16001322 | 7 17 21 | mc8000196 | 7 9 |
| mc16001325 | 4 7 16 20 21 24 | mc8000197 | 1 6 7 10 |
| mc16001326 | 1 6 7 21 | mc8000199 | 7 15 21 |
| mc16001331 | 1 | mc8000202 | 7 8 10 |
| mc16001332 | 22 24 | mc8000206 | 3 6 7 21 |
| mc16001334 | 9 | mc8000209 | 6 |
| mc16001336 | 1 6 7 10 21 | mc8000214 | 6 |
| mc16001338 | 7 9 10 21 24 | mc8000222 | 7 12 18 22 23 24 |
| mc16001348 | 3 4 7 21 | mc8000229 | 21 |
| mc16001352 | 3 4 7 21 | mc8000231 | 6 8 10 |
| mc16001355 | 21 | mc8000236 | 7 12 13 17 19 21 23 |
| mc16001356 | 4 7 21 | mc8000248 | 21 |
| mc16001357 | 3 7 17 21 | mc8000270 | 7 8 10 15 20 21 24 |
| mc16001360 | 1 6 21 | mc8000273 | 1 7 21 |
| mc16001361 | 7 12 17 21 | mc8000282 | 11 13 |
| mc16001362 | 10 12 16 21 | mc8000284 | 11 12 13 21 |
| mc16001365 | 7 12 16 21 | mc8000287 | 11 12 13 21 |
| mc16001374 | 6 9 21 | mc8000293 | 11 12 13 21 |
| mc16001376 | 15 21 | mc8000304 | 11 12 13 21 |
| mc16001377 | 7 11 12 13 21 | mc8000306 | 11 12 13 21 |
| mc16001378 | 6 10 | mc8000310 | 11 12 13 21 |
| mc16001379 | 1 4 6 7 10 | mc8000314 | 21 |
| mc16001384 | 7 21 | mc8000315 | 7 9 12 21 |
| mc16001395 | 1 6 10 | mc8000318 | 1 3 6 7 21 |
| mc16001397 | 21 | mc8000320 | 4 7 9 21 |
| mc17000004 | 10 21 | mc8000321 | 21 |
| mc17000007 | 3 4 11 21 | mc8000328 | 5 21 |
| mc17000009 | 1 7 10 | mc8000333 | 10 16 21 |
| mc17000012 | 9 10 | mc8000334 | 1 3 7 11 15 21 |
| mc17000015 | 2 7 9 21 | mc8000335 | 1 4 6 7 10 21 |
| mc17000042 | 12 13 | mc8000337 | 6 10 21 |
| mc17000044 | 4 7 21 | mc8000344 | 7 10 21 |
| mc17000045 | 7 8 9 21 | mc8000347 | 1 |
| mc17000075 | 7 15 16 17 21 | mc8000350 | 9 21 |
| mc17000080 | 15 | mc8000352 | 10 15 16 21 |
| mc17000087 | 4 7 16 21 | mc8000366 | 1 5 7 11 13 15 16 |
| mc17000095 | 1 10 | mc8000367 | 1 7 11 13 |
| mc17000111 | 4 7 21 | mc8000368 | 6 7 12 16 21 24 |
| mc17000119 | 4 7 16 21 | mc8000369 | 7 12 21 24 |
| mc17000132 | 6 7 9 12 13 21 | mc8000370 | 6 7 21 |
| mc17000149 | 1 7 21 24 | mc8000371 | 7 12 16 21 24 |
| mc17000150 | 1 6 7 10 | mc8000372 | 6 7 12 21 24 |
| mc17000153 | 3 7 13 15 21 23 | mc8000373 | 21 |
| mc17000154 | 9 21 | mc8000375 | 7 21 |
| mc17000158 | 7 9 21 | mc8000377 | 3 7 21 |
| mc17000159 | 7 9 10 21 | mc8000382 | 6 7 10 16 20 21 |
| mc17000164 | 10 | mc8000390 | 6 7 11 15 21 |
| mc17000166 | 5 7 21 | mc8000394 | 1 9 21 |
| mc17000169 | 21 | mc8000395 | 4 7 |
| mc17000171 | 7 11 21 | mc8000400 | 1 6 8 10 |
| mc17000181 | 7 21 | mc8000401 | 7 17 21 |
| mc17000186 | 7 12 21 | mc8000402 | 7 21 |
| mc17000197 | 7 10 15 16 21 | mc8000415 | 10 21 |
| mc17000201 | 6 7 8 11 13 21 | mc8000416 | 5 |
| mc17000202 | 4 7 21 | mc8000419 | 7 10 21 24 |
| mc17000203 | 7 15 21 | mc8000434 | 7 10 15 16 |
| mc17000209 | 3 4 7 21 | mc8000461 | 21 |
| mc17000210 | 7 10 12 21 | mc8000492 | 3 7 21 |
| mc17000224 | 1 6 7 | mc8000498 | 22 24 |
| mc17000235 | 1 7 10 21 | mc8000500 | 24 |
| mc17000236 | 10 12 13 | mc8000501 | 1 7 11 21 |
| mc17000241 | 10 12 13 | mc8000502 | 4 7 21 |
| mc17000268 | 10 | mc8000506 | 1 17 21 |
| mc17000284 | 3 6 7 10 15 21 | mc8000521 | 1 6 8 10 21 |
| mc17000295 | 7 21 | mc8000522 | 3 7 21 |
| mc17000297 | 4 7 21 | mc8000540 | 7 17 |
| mc17000331 | 7 10 12 15 16 20 21 | mc8000551 | 1 21 |
| mc17000332 | 7 17 21 | mc8000559 | 6 9 |
| mc17000337 | 1 3 6 7 10 21 | mc8000564 | 6 7 13 17 19 21 23 |
| mc17000339 | 10 | mc8000574 | 9 10 21 |
| mc17000348 | 6 16 21 | mc8000582 | 1 6 7 |
| mc17000351 | 21 | mc8000587 | 6 7 10 16 21 24 |
| mc17000353 | 21 | mc8000589 | 6 7 10 16 21 24 |
| mc17000355 | 6 7 9 21 | mc8000595 | 9 |
| mc17000356 | 6 21 | mc8000596 | 10 |
| mc17000358 | 6 | mc8000597 | 10 |
| mc17000360 | 1 6 7 10 11 21 | mc8000603 | 13 21 |
| mc17000361 | 1 | mc8000604 | 1 21 |
| mc17000362 | 7 8 9 12 21 | mc8000605 | 4 5 9 21 |
| mc17000364 | 4 7 16 21 | mc8000637 | 10 12 |
| mc17000366 | 3 7 8 21 | mc8000641 | 6 7 13 21 |
| mc17000369 | 7 9 21 | mc8000642 | 6 13 21 |
| mc17000371 | 1 4 7 21 | mc8000645 | 7 8 10 11 12 13 15 18 |
| mc17000372 | 1 2 21 | mc8000650 | 7 10 17 19 20 21 23 |
| mc17000374 | 3 4 7 16 21 | mc8000655 | 7 21 |
| mc17000377 | 7 | mc8000665 | 4 7 18 21 |
| mc17000380 | 4 7 | mc8000669 | 1 7 21 |
| mc17000381 | 4 6 7 15 21 | mc8000671 | 7 21 |
| mc17000384 | 7 12 17 21 23 24 | mc8000673 | 6 7 8 11 15 21 |
| mc17000385 | 3 5 6 7 8 15 21 | mc8000678 | 4 7 13 21 |
| mc17000386 | 3 7 11 21 | mc8000695 | 3 4 7 15 21 |
| mc17000389 | 7 21 | mc8000696 | 7 15 21 |
| mc17000390 | 9 17 21 22 24 | mc8000704 | 1 7 |
| mc17000393 | 8 11 | mc8000734 | 3 7 11 21 |
| mc17000394 | 6 | mc8000737 | 3 15 16 19 20 21 |
| mc17000395 | 7 | mc8000742 | 4 7 10 |
| mc17000396 | 5 21 | mc8000748 | 16 21 |
| mc17000400 | 7 11 21 | mc8000756 | 6 7 16 21 24 |
| mc17000403 | 1 21 | mc8000757 | 9 10 12 21 |
| mc17000404 | 1 7 21 | mc8000759 | 1 9 |
| mc17000405 | 7 12 21 | mc8000762 | 6 7 8 21 |
| mc17000406 | 7 | mc8000770 | 4 7 15 16 19 20 21 |
| mc17000407 | 10 | mc8000774 | 21 |
| mc17000409 | 5 21 | mc8000775 | 4 7 21 |
| mc17000410 | 1 | mc8000778 | 1 6 7 21 |
| mc17000411 | 7 8 10 | mc8000798 | 1 7 21 24 |
| mc17000420 | 9 | mc8000799 | 6 9 |
| mc17000431 | 6 21 | mc8000815 | 1 |
| mc17000432 | 6 16 21 | mc8000823 | 7 21 |
| mc17000433 | 6 21 | mc8000829 | 7 8 21 |
| mc17000435 | 6 21 22 | mc8000835 | 7 |
| mc17000437 | 6 21 | mc8000842 | 6 7 21 |
| mc17000438 | 7 9 10 12 21 | mc8000843 | 1 7 21 |
| mc17000441 | 4 7 18 21 | mc8000844 | 7 21 |
| mc17000448 | 3 7 | mc8000848 | 7 |
| mc17000449 | 1 2 7 9 | mc8000859 | 10 12 |
| mc17000451 | 11 12 21 23 24 | mc8000861 | 1 10 13 15 |
| mc17000460 | 6 7 21 | mc8000869 | 1 |
| mc17000462 | 7 10 21 | mc8000884 | 7 17 21 |
| mc17000469 | 1 21 | mc8000890 | 1 7 9 21 |
| mc17000478 | 2 17 21 | mc8000893 | 9 21 |
| mc17000479 | 1 7 21 | mc8000895 | 3 21 |
| mc17000482 | 5 21 | mc8000903 | 1 7 9 |
| mc17000486 | 8 21 | mc8000904 | 1 7 8 10 |
| mc17000499 | 9 21 | mc8000906 | 4 7 21 |
| mc17000501 | 1 2 7 9 21 | mc8000910 | 1 6 7 10 |
| mc17000503 | 15 21 | mc8000911 | 4 7 21 |
| mc17000505 | 4 7 16 20 21 24 | mc8000916 | 4 7 21 |
| mc17000507 | 2 7 9 17 21 | mc8000919 | 7 12 21 |
| mc17000512 | 15 | mc8000921 | 4 7 21 |
| mc17000519 | 1 15 | mc8000926 | 4 7 21 |
| mc17000534 | 5 7 | mc8000928 | 6 7 12 21 |
| mc17000538 | 4 7 21 | mc8000930 | 7 9 17 21 |
| mc17000541 | 4 7 21 | mc8000932 | 15 21 |
| mc17000544 | 4 7 21 | mc8000933 | 1 3 4 7 |
| mc17000546 | 7 10 21 | mc8000940 | 5 6 7 21 |
| mc17000547 | 15 16 | mc8000942 | 4 7 8 10 22 |
| mc17000554 | 7 21 | mc8000943 | 4 5 21 |
| mc17000555 | 7 | mc8000944 | 1 7 |
| mc17000559 | 5 21 | mc8000945 | 7 21 |
| mc17000561 | 10 12 13 20 | mc8000946 | 21 |
| mc17000562 | 6 7 8 | mc8000947 | 15 21 |
| mc17000567 | 1 8 13 21 | mc8000948 | 6 |
| mc17000569 | 1 3 4 6 7 10 15 21 | mc8000949 | 4 7 21 |
| mc17000571 | 9 | mc8000950 | 3 4 7 21 |
| mc17000584 | 3 8 15 21 | mc8000953 | 1 10 |
| mc17000588 | 6 9 | mc8000956 | 13 21 |
| mc17000589 | 6 9 21 | mc8000957 | 1 7 8 10 21 |
| mc17000592 | 6 21 | mc8000961 | 10 21 |
| mc17000593 | 9 21 | mc8000967 | 7 9 |
| mc17000594 | 7 8 21 | mc8000969 | 7 17 21 |
| mc17000610 | 24 | mc8000976 | 2 7 8 9 10 17 21 |
| mc17000616 | 1 7 | mc8000977 | 9 21 |
| mc17000617 | 7 9 12 17 21 24 | mc8000981 | 7 |
| mc17000620 | 1 7 9 21 | mc8000984 | 1 |
| mc17000621 | 21 | mc8000985 | 5 21 |
| mc17000622 | 21 | mc8000988 | 4 7 21 |
| mc17000623 | 7 11 12 15 21 | mc8000989 | 9 21 |
| mc17000624 | 6 9 10 12 21 | mc8000995 | 2 9 |
| mc17000625 | 1 10 13 15 | mc8000996 | 7 9 21 |
| mc17000626 | 3 | mc8000997 | 1 2 7 9 21 |
| mc17000628 | 1 7 10 | mc8001003 | 6 7 10 13 21 |
| mc17000629 | 4 21 | mc8001005 | 1 6 21 |
| mc17000632 | 4 7 21 | mc8001013 | 4 7 21 |
| mc17000635 | 3 4 7 21 | mc8001015 | 10 13 |
| mc17000636 | 3 17 20 21 24 | mc8001020 | 3 4 7 9 10 |
| mc17000643 | 1 3 6 7 15 16 | mc8001021 | 10 13 |
| mc17000649 | 4 7 10 15 16 18 20 21 | mc8001024 | 6 7 9 17 21 |
| mc17000652 | 3 6 7 8 10 21 | mc8001027 | 4 7 21 |
| mc17000653 | 3 6 7 8 10 21 | mc8001028 | 7 9 10 21 |
| mc17000663 | 2 9 21 | mc8001030 | 7 12 |
| mc17000672 | 4 7 21 | mc8001032 | 12 15 21 |
| mc17000682 | 2 7 9 17 21 | mc8001035 | 4 |
| mc17000684 | 4 7 10 11 21 | mc8001043 | 4 7 8 21 |
| mc17000688 | 1 2 13 15 20 21 24 | mc8001045 | 6 10 13 |
| mc17000689 | 7 | mc8001046 | 1 21 |
| mc17000691 | 2 7 9 21 | mc8001064 | 4 7 |
| mc17000692 | 10 | mc8001067 | 1 7 12 21 |
| mc17000697 | 4 7 21 | mc8001068 | 3 7 21 |
| mc17000698 | 11 13 21 | mc8001099 | 7 9 10 13 21 |
| mc17000699 | 8 10 | mc8001112 | 4 7 12 16 21 |
| mc17000700 | 6 7 17 21 | mc8001126 | 1 9 21 |
| mc17000701 | 5 7 | mc8001136 | 4 7 8 9 11 13 21 |
| mc17000702 | 1 6 21 | mc8001140 | 7 10 15 16 20 21 |
| mc17000703 | 5 21 | mc8001150 | 7 11 17 21 |
| mc17000710 | 4 7 21 | mc8001152 | 3 4 7 17 21 |
| mc17000711 | 1 7 13 21 | mc8001154 | 7 8 10 11 21 |
| mc17000712 | 7 9 21 | mc8001172 | 7 10 11 13 15 18 20 21 |
| mc17000713 | 7 10 13 21 | mc8001176 | 7 21 |
| mc17000714 | 7 9 12 17 21 | mc8001183 | 2 9 13 21 23 |
| mc17000718 | 13 18 | mc8001187 | 3 6 7 11 15 16 21 |
| mc17000719 | 1 6 16 21 | mc8001191 | 10 13 |
| mc17000722 | 6 7 9 10 13 18 21 | mc8001193 | 2 7 9 21 |
| mc17000723 | 6 7 10 13 21 | mc8001198 | 6 7 8 9 10 21 |
| mc17000726 | 6 7 8 11 13 21 | mc8001199 | 1 6 7 10 |
| mc17000727 | 7 9 13 21 | mc8001204 | 7 10 12 21 |
| mc17000728 | 6 7 8 11 13 21 | mc8001215 | 7 10 13 21 |
| mc17000729 | 7 9 13 21 | mc8001216 | 1 10 21 |
| mc17000732 | 7 10 13 18 21 | mc8001218 | 4 7 21 |
| mc17000733 | 10 13 | mc8001223 | 5 7 15 16 |
| mc17000735 | 10 11 13 21 | mc8001228 | 4 5 7 8 21 |
| mc17000743 | 4 7 10 15 16 | mc8001232 | 7 9 10 12 21 24 |
| mc17000747 | 7 21 24 | mc8001235 | 21 |
| mc17000748 | 7 | mc8001236 | 7 |
| mc17000749 | 1 7 21 | mc8001237 | 1 |
| mc17000754 | 4 7 10 21 | mc8001238 | 4 7 21 |
| mc17000756 | 7 17 21 | mc8001241 | 8 10 16 |
| mc17000758 | 13 | mc8001242 | 21 |
| mc17000759 | 1 6 7 21 | mc8001243 | 1 3 6 11 21 |
| mc17000762 | 1 2 7 9 21 | mc8001245 | 6 7 12 21 |
| mc17000768 | 3 6 13 21 | mc8001246 | 5 7 21 |
| mc17000769 | 6 13 21 | mc8001247 | 1 7 21 |
| mc17000770 | 7 21 | mc8001248 | 3 4 7 21 |
| mc17000771 | 1 3 7 21 | mc8001249 | 1 3 15 16 18 21 |
| mc17000774 | 1 7 11 21 | mc8001250 | 1 6 7 10 21 |
| mc17000779 | 1 4 7 21 | mc8001255 | 3 4 7 21 |
| mc17000781 | 5 7 21 | mc8001257 | 7 17 21 |
| mc17000784 | 1 3 7 16 | mc8001259 | 7 9 11 |
| mc17000786 | 1 10 | mc8001264 | 1 |
| mc17000788 | 21 | mc8001266 | 1 7 11 21 |
| mc17000789 | 21 | mc8001268 | 6 9 |
| mc17000792 | 13 21 | mc8001269 | 3 4 7 21 |
| mc17000795 | 1 6 7 8 10 21 | mc8001270 | 1 6 7 13 |
| mc17000796 | 4 7 21 | mc8001273 | 1 2 7 21 |
| mc17000798 | 7 9 21 | mc8001274 | 3 6 7 21 |
| mc17000799 | 7 15 | mc8001277 | 12 |
| mc17000800 | 4 7 13 15 21 | mc8001279 | 1 2 7 |
| mc17000801 | 7 13 17 21 24 | mc8001284 | 7 9 |
| mc17000803 | 7 13 15 20 21 | mc8001285 | 6 7 21 |
| mc17000804 | 1 3 4 6 7 9 10 11 12 13 15 16 18 19 21 | mc8001305 | 4 7 8 21 |
| mc17000805 | 3 7 13 15 20 21 | mc8001317 | 4 7 21 |
| mc17000806 | 4 7 21 | mc8001320 | 7 |
| mc17000807 | 1 2 7 9 | mc8001322 | 7 9 10 |
| mc17000808 | 1 3 4 7 21 | mc8001324 | 7 10 13 15 |
| mc17000812 | 7 11 13 21 | mc8001325 | 6 |
| mc17000819 | 4 7 21 | mc8001329 | 4 7 18 20 21 |
| mc17000820 | 4 7 21 | mc8001362 | 3 4 7 21 |
| mc17000821 | 19 20 21 | mc8001375 | 4 7 21 |
| mc17000830 | 5 7 | mc8001386 | 4 7 21 |
| mc17000831 | 3 4 7 21 | mc8001392 | 4 7 10 21 |
| mc17000832 | 1 6 7 10 12 21 | mc8001395 | 6 7 21 |
| mc17000835 | 11 13 15 | mc8001405 | 21 |
| mc17000836 | 21 | mc8001421 | 6 7 21 |
| mc17000840 | 21 | mc8001422 | 4 21 |
| mc17000842 | 4 7 21 | mc8001424 | 12 13 |
| mc17000846 | 5 21 | mc8001425 | 2 7 9 12 |
| mc17000847 | 6 7 | mc8001426 | 2 7 9 12 16 |
| mc17000849 | 1 7 | mc8001428 | 7 10 12 21 |
| mc17000850 | 7 | mc8001430 | 9 21 |
| mc17000862 | 10 11 13 | mc8001431 | 7 9 21 |
| mc17000867 | 4 21 | mc8001432 | 6 11 21 |
| mc17000868 | 6 7 21 | mc8001439 | 21 |
| mc17000870 | 11 13 21 | mc8001444 | 9 |
| mc17000878 | 11 13 21 | mc8001445 | 7 10 12 13 15 21 22 |
| mc17000884 | 6 7 21 | mc8001448 | 7 10 12 13 15 21 |
| mc17000886 | 6 7 21 | mc8001449 | 7 10 12 13 15 21 22 |
| mc17000888 | 7 | mc8001452 | 21 |
| mc17000890 | 7 8 | mc8001453 | 4 7 21 |
| mc17000891 | 1 4 7 21 | mc8001454 | 7 8 10 16 21 |
| mc17000896 | 4 7 21 | mc8001458 | 10 16 |
| mc17000897 | 16 19 20 21 23 24 | mc8001464 | 2 7 9 17 21 |
| mc17000899 | 7 10 12 21 | mc8001473 | 6 7 21 |
| mc17000908 | 6 | mc8001490 | 1 7 21 |
| mc17000910 | 10 13 | mc8001514 | 7 12 17 21 24 |
| mc17000914 | 11 13 21 | mc8001554 | 12 17 20 21 23 24 |
| mc17000926 | 11 | mc8001558 | 1 21 |
| mc17000956 | 10 13 | mc8001559 | 12 13 14 15 21 |
| mc17000966 | 21 | mc8001562 | 12 13 15 21 |
| mc17000972 | 21 | mc8001564 | 12 13 15 21 |
| mc17000973 | 6 | mc8001565 | 12 13 15 21 |
| mc17000995 | 7 21 | mc8001566 | 12 13 15 21 |
| mc17000996 | 7 10 15 | mc8001569 | 3 6 15 |
| mc17000998 | 10 21 | mc8001570 | 1 7 21 |
| mc17001001 | 6 7 21 | mc8001572 | 7 12 17 20 21 24 |
| mc17001003 | 1 13 21 | mc8001573 | 7 9 10 |
| mc17001004 | 7 | mc8001584 | 1 11 21 |
| mc17001005 | 2 9 13 21 | mc8001595 | 4 7 8 18 19 20 21 |
| mc17001006 | 1 2 7 9 21 | mc8001599 | 6 |
| mc17001014 | 1 21 | mc8001600 | 4 6 7 11 15 18 20 21 |
| mc17001015 | 1 | mc8001601 | 4 7 15 21 |
| mc17001025 | 4 7 10 18 19 20 21 | mc8001604 | 3 4 7 12 13 15 21 |
| mc17001031 | 4 7 21 | mc8001605 | 12 17 |
| mc17001036 | 3 4 6 7 9 21 | mc8001609 | 4 7 8 17 21 |
| mc17001045 | 4 7 | mc8001617 | 1 21 |
| mc17001048 | 9 21 | mc8001618 | 1 2 7 9 |
| mc17001050 | 5 7 21 | mc8001619 | 10 13 21 24 |
| mc17001057 | 3 7 12 15 16 19 20 21 | mc8001627 | 3 7 17 21 |
| mc17001059 | 5 21 | mc8001630 | 1 2 9 |
| mc17001061 | 3 7 21 | mc8001632 | 7 21 |
| mc17001062 | 5 7 | mc8001634 | 7 21 |
| mc17001063 | 1 3 7 11 21 | mc8001635 | 9 21 |
| mc17001064 | 6 7 9 | mc8001636 | 21 |
| mc17001065 | 1 4 7 21 | mc8001639 | 6 13 21 |
| mc17001071 | 7 9 | mc8001640 | 6 7 8 11 13 21 |
| mc17001076 | 7 9 21 | mc8001641 | 1 21 |
| mc17001079 | 21 | mc8001645 | 6 |
| mc17001081 | 4 7 16 21 | mc8001647 | 3 7 21 |
| mc17001082 | 1 6 10 12 16 19 21 | mc8001649 | 2 4 7 19 20 21 |
| mc17001084 | 2 7 9 21 | mc8001651 | 1 21 |
| mc17001085 | 5 21 | mc8001652 | 9 |
| mc17001090 | 21 | mc8001654 | 1 21 |
| mc17001091 | 15 16 21 | mc8001655 | 1 3 7 10 11 16 21 |
| mc17001094 | 7 17 21 | mc8001658 | 4 7 21 |
| mc17001095 | 1 11 21 24 | mc8001659 | 7 12 21 |
| mc17001097 | 21 | mc8001661 | 3 7 10 12 17 21 23 |
| mc17001099 | 4 5 7 21 | mc8001663 | 1 21 |
| mc17001101 | 6 17 21 | mc8001665 | 21 |
| mc17001102 | 7 12 13 | mc8001667 | 6 7 13 17 |
| mc17001110 | 7 21 | mc8001670 | 6 9 21 |
| mc17001111 | 5 21 | mc8001671 | 5 7 21 |
| mc17001112 | 7 8 9 10 12 13 | mc8001673 | 3 4 7 11 17 21 |
| mc17001113 | 7 8 12 13 | mc8001674 | 7 9 21 |
| mc17001117 | 4 7 19 21 | mc8001677 | 4 7 13 15 21 |
| mc17001118 | 3 7 8 10 21 | mc8001678 | 1 2 9 |
| mc17001119 | 7 12 | mc8001679 | 4 5 21 |
| mc17001120 | 4 6 21 | mc8001680 | 6 7 |
| mc17001122 | 6 7 | mc8001681 | 6 7 8 11 21 |
| mc17001125 | 2 7 9 10 | mc8001701 | 4 7 21 |
| mc17001126 | 6 13 | mc8001711 | 3 4 7 |
| mc17001129 | 4 7 21 | mc8001712 | 2 3 4 7 9 11 21 |
| mc17001130 | 7 15 16 | mc8001713 | 7 12 18 21 |
| mc17001132 | 4 7 19 20 21 | mc8001715 | 1 21 |
| mc17001140 | 10 21 | mc8001724 | 6 7 9 17 21 |
| mc17001141 | 7 10 21 | mc8001730 | 1 7 21 |
| mc17001142 | 10 | mc8001732 | 1 6 13 21 |
| mc17001146 | 10 | mc8001735 | 7 21 |
| mc17001149 | 4 7 21 | mc8001746 | 4 6 21 |
| mc17001151 | 1 4 7 21 23 | mc8001748 | 6 9 21 |
| mc17001154 | 10 | mc8001755 | 3 4 7 |
| mc17001166 | 1 21 | mc8001757 | 5 7 |
| mc17001175 | 3 5 7 15 16 18 21 | mc8001762 | 7 21 |
| mc17001181 | 1 10 | mc8001766 | 1 |
| mc17001192 | 3 4 6 7 17 21 | mc8001770 | 1 2 7 21 |
| mc17001213 | 6 21 | mc8001771 | 4 7 21 |
| mc17001218 | 1 6 21 | mc8001772 | 6 13 21 |
| mc17001221 | 7 9 12 21 | mc8001773 | 7 10 12 17 21 |
| mc17001231 | 1 6 21 | mc8001774 | 12 15 16 21 |
| mc17001237 | 7 10 21 | mc8001776 | 1 6 21 |
| mc17001242 | 7 10 15 21 | mc8001779 | 7 9 21 |
| mc17001244 | 7 10 24 | mc8001780 | 1 4 7 |
| mc17001246 | 7 10 21 | mc8001781 | 5 7 21 |
| mc17001248 | 7 21 | mc8001782 | 3 4 7 11 17 21 |
| mc17001249 | 3 4 6 7 11 21 | mc8001788 | 7 12 22 |
| mc17001251 | 4 7 21 | mc8001806 | 1 11 21 |
| mc17001252 | 21 | mc8001809 | 1 7 |
| mc17001254 | 21 | mc8001811 | 1 4 6 7 21 |
| mc17001255 | 10 15 16 | mc8001844 | 7 9 12 21 24 |
| mc17001260 | 6 | mc8001848 | 1 21 |
| mc17001264 | 10 | mc8001850 | 1 7 |
| mc17001265 | 9 10 | mc8001855 | 1 10 |
| mc17001267 | 3 4 6 7 9 11 16 21 | mc8001858 | 1 |
| mc17001268 | 4 7 | mc8001863 | 7 12 17 21 |
| mc17001269 | 1 6 8 10 21 | mc8001870 | 4 6 8 11 21 |
| mc17001271 | 7 21 | mc8001873 | 1 6 7 21 |
| mc17001276 | 5 7 21 | mc8001875 | 1 6 |
| mc17001283 | 15 21 | mc8001877 | 4 7 21 |
| mc17001287 | 4 7 21 | mc8001879 | 9 |
| mc17001291 | 4 21 | mc8001880 | 8 21 |
| mc17001294 | 1 21 | mc8001884 | 7 21 |
| mc17001295 | 6 21 | mc8001890 | 21 |
| mc17001297 | 7 15 20 21 | mc8001892 | 1 6 7 |
| mc17001298 | 4 7 21 | mc8001912 | 21 |
| mc17001301 | 9 21 | mc8001913 | 2 7 9 21 |
| mc17001302 | 7 9 21 | mc8001915 | 4 7 13 19 21 |
| mc17001305 | 7 16 17 19 21 | mc8001924 | 4 7 15 20 21 |
| mc17001308 | 7 11 13 15 21 | mc8001925 | 7 |
| mc17001309 | 7 13 15 21 | mc8001926 | 4 7 21 |
| mc17001310 | 7 13 15 21 | mc8001941 | 6 7 17 21 |
| mc17001311 | 7 8 9 13 21 | mc8001945 | 12 13 21 |
| mc17001314 | 7 9 10 17 21 | mc8001948 | 7 9 21 |
| mc17001316 | 8 10 13 17 18 | mc8001949 | 1 2 7 21 24 |
| mc17001317 | 7 10 21 | mc8001950 | 21 |
| mc17001336 | 1 21 | mc8001958 | 4 7 8 19 20 21 |
| mc17001360 | 15 16 21 | mc8001959 | 7 10 21 |
| mc17001388 | 1 3 6 7 10 12 | mc8001962 | 15 19 |
| mc17001390 | 7 | mc8001963 | 11 13 21 |
| mc17001398 | 1 11 21 | mc8001964 | 1 |
| mc17001410 | 2 6 9 11 15 16 | mc8001966 | 2 4 7 9 21 |
| mc17001414 | 1 6 7 | mc8001973 | 3 7 21 |
| mc17001415 | 7 8 9 | mc8001974 | 1 |
| mc17001430 | 7 9 10 | mc8001975 | 1 |
| mc17001446 | 7 10 12 16 17 19 21 | mc8001978 | 4 7 21 |
| mc17001474 | 7 17 21 | mc8001984 | 7 12 17 21 23 |
| mc17001475 | 4 7 21 | mc8001991 | 5 7 21 |
| mc17001495 | 4 7 8 21 | mc8001994 | 6 7 21 |
| mc17001502 | 7 17 21 | mc8001995 | 3 4 9 21 |
| mc17001504 | 7 12 13 17 19 21 23 | mc8002001 | 4 7 21 |
| mc17001507 | 7 12 21 | mc8002002 | 3 7 11 21 |
| mc17001511 | 3 7 21 | mc8002004 | 7 21 |
| mc17001522 | 1 6 7 10 21 24 | mc8002005 | 10 13 |
| mc17001541 | 4 21 | mc8002006 | 7 17 21 |
| mc17001554 | 1 2 7 9 13 19 | mc8002008 | 10 |
| mc17001556 | 1 15 16 20 21 | mc8002009 | 6 7 21 |
| mc17001561 | 1 7 21 | mc8002011 | 12 21 |
| mc17001562 | 9 | mc8002013 | 3 7 10 15 17 |
| mc17001568 | 6 7 15 | mc8002019 | 6 9 17 21 |
| mc17001573 | 7 8 10 21 | mc8002020 | 7 9 21 |
| mc17001592 | 7 | mc8002021 | 4 21 |
| mc17001606 | 4 6 7 10 13 21 | mc8002026 | 1 6 7 21 |
| mc17001608 | 4 7 21 | mc8002031 | 6 9 21 |
| mc17001609 | 1 | mc8002032 | 13 21 24 |
| mc17001611 | 1 6 7 10 | mc8002033 | 6 7 21 24 |
| mc17001616 | 7 15 21 | mc8002043 | 1 21 24 |
| mc17001622 | 1 2 7 9 21 | mc8002045 | 6 7 9 19 21 |
| mc17001629 | 1 4 7 14 21 | mc8002051 | 7 9 21 |
| mc17001632 | 1 | mc8002053 | 7 |
| mc17001633 | 4 7 21 | mc8002065 | 1 7 9 |
| mc17001635 | 4 6 7 21 | mc8002070 | 1 6 7 |
| mc17001636 | 3 4 7 | mc8002071 | 7 9 21 |
| mc17001639 | 7 8 10 | mc8002086 | 7 21 |
| mc17001651 | 9 12 13 21 23 24 | mc8002090 | 3 7 12 17 21 |
| mc17001673 | 2 7 9 21 | mc8002102 | 7 10 12 15 16 19 20 21 |
| mc17001676 | 7 9 | mc8002108 | 7 10 12 13 21 |
| mc17001686 | 7 21 | mc8002119 | 1 6 9 21 |
| mc17001692 | 16 | mc8002120 | 7 9 21 |
| mc17001693 | 1 7 9 | mc8002125 | 4 7 21 |
| mc17001694 | 1 7 9 | mc8002128 | 9 |
| mc17001695 | 6 7 9 17 21 | mc8002129 | 7 10 |
| mc17001699 | 1 6 7 10 | mc8002134 | 1 |
| mc17001700 | 1 9 21 | mc8002137 | 7 10 |
| mc17001701 | 9 21 | mc8002138 | 1 15 19 |
| mc17001714 | 4 7 10 16 20 21 | mc8002141 | 2 3 4 7 9 21 |
| mc17001716 | 4 7 16 21 | mc8002144 | 1 7 |
| mc17001729 | 6 21 | mc8002145 | 1 6 21 |
| mc17001735 | 4 7 11 19 21 | mc8002147 | 1 6 7 21 |
| mc17001738 | 2 7 9 21 | mc8002150 | 11 12 13 21 |
| mc17001740 | 1 6 7 21 | mc8002152 | 11 21 |
| mc17001746 | 7 10 15 | mc9000012 | 1 7 |
| mc17001749 | 1 7 9 21 | mc9000019 | 6 8 11 13 15 21 |
| mc17001752 | 7 12 19 | mc9000020 | 6 8 13 15 21 |
| mc17001758 | 11 21 | mc9000021 | 6 8 11 15 21 |
| mc17001760 | 1 3 7 11 21 | mc9000030 | 6 7 15 21 |
| mc17001761 | 9 | mc9000040 | 6 7 16 20 21 |
| mc17001767 | 1 3 7 11 15 21 | mc9000041 | 6 7 21 |
| mc17001774 | 4 7 21 | mc9000044 | 6 7 21 |
| mc17001778 | 4 7 21 | mc9000045 | 6 7 21 |
| mc17001779 | 7 10 21 | mc9000047 | 6 7 21 |
| mc17001784 | 8 10 21 | mc9000049 | 6 7 21 |
| mc17001792 | 7 9 12 16 | mc9000051 | 6 7 21 |
| mc17001812 | 7 8 10 21 | mc9000056 | 6 7 15 21 |
| mc17001815 | 2 7 9 17 21 | mc9000058 | 4 7 8 21 |
| mc17001818 | 7 8 9 | mc9000063 | 9 10 12 21 |
| mc17001822 | 10 | mc9000066 | 4 7 8 10 13 15 18 20 21 |
| mc17001827 | 4 6 7 21 | mc9000097 | 7 21 |
| mc17001828 | 7 10 21 | mc9000107 | 3 21 |
| mc17001829 | 21 | mc9000110 | 1 3 7 |
| mc17001832 | 1 7 9 10 17 21 | mc9000118 | 1 6 21 |
| mc17001834 | 1 | mc9000120 | 4 7 |
| mc17001835 | 7 21 | mc9000121 | 3 7 16 21 |
| mc17001837 | 7 21 | mc9000123 | 10 21 |
| mc17001843 | 3 4 7 | mc9000125 | 7 9 10 |
| mc17001844 | 13 15 | mc9000127 | 21 |
| mc17001849 | 21 | mc9000131 | 4 7 |
| mc17001850 | 6 7 12 13 | mc9000146 | 10 12 21 |
| mc17001854 | 7 10 21 | mc9000166 | 7 |
| mc17001858 | 1 | mc9000174 | 4 21 |
| mc17001859 | 6 7 11 21 | mc9000176 | 10 13 21 |
| mc17001860 | 3 10 | mc9000209 | 10 13 21 |
| mc18000003 | 4 7 16 21 | mc9000220 | 6 7 21 |
| mc18000005 | 3 6 21 | mc9000221 | 6 |
| mc18000009 | 10 21 | mc9000223 | 3 6 21 |
| mc18000021 | 1 17 21 | mc9000227 | 7 12 17 21 |
| mc18000025 | 1 3 6 7 10 | mc9000231 | 1 2 13 15 20 21 24 |
| mc18000034 | 7 17 21 | mc9000232 | 15 21 |
| mc18000040 | 4 7 21 | mc9000233 | 5 7 |
| mc18000044 | 8 | mc9000234 | 1 3 4 7 21 |
| mc18000045 | 2 7 9 17 21 | mc9000235 | 10 13 21 |
| mc18000050 | 3 4 15 21 | mc9000237 | 5 21 |
| mc18000058 | 4 7 21 | mc9000238 | 7 12 13 21 |
| mc18000069 | 4 7 21 | mc9000239 | 7 12 21 |
| mc18000081 | 7 10 16 21 | mc9000240 | 7 12 21 22 23 |
| mc18000084 | 9 10 21 | mc9000243 | 7 |
| mc18000090 | 3 4 7 21 | mc9000244 | 6 7 10 13 21 |
| mc18000093 | 7 9 10 13 | mc9000245 | 3 6 7 11 |
| mc18000097 | 4 7 9 10 15 21 | mc9000246 | 1 10 |
| mc18000098 | 6 12 | mc9000248 | 4 7 16 |
| mc18000112 | 4 6 7 21 | mc9000249 | 10 21 |
| mc18000115 | 6 7 9 10 16 18 19 20 21 | mc9000252 | 1 3 4 7 8 13 |
| mc18000119 | 2 4 7 9 20 21 | mc9000256 | 4 6 7 9 13 21 |
| mc18000129 | 3 7 8 15 16 21 | mc9000259 | 1 4 7 |
| mc18000132 | 1 6 7 | mc9000260 | 7 9 21 |
| mc18000134 | 9 10 21 | mc9000261 | 9 10 11 21 |
| mc18000153 | 8 10 13 21 | mc9000263 | 3 4 6 7 17 21 |
| mc18000183 | 4 7 21 | mc9000266 | 3 4 7 17 21 22 |
| mc18000196 | 7 9 21 24 | mc9000267 | 1 7 9 10 21 |
| mc18000198 | 1 21 | mc9000269 | 4 7 |
| mc18000227 | 7 12 21 | mc9000274 | 21 |
| mc18000228 | 7 12 21 | mc9000278 | 10 18 21 22 |
| mc18000229 | 7 12 17 21 | mc9000279 | 7 8 10 |
| mc18000232 | 7 12 21 | mc9000281 | 1 6 7 8 21 |
| mc18000234 | 7 12 21 | mc9000282 | 7 15 16 |
| mc18000236 | 10 12 17 19 21 | mc9000284 | 7 21 |
| mc18000238 | 12 17 21 | mc9000285 | 4 7 10 16 21 |
| mc18000239 | 13 21 23 | mc9000288 | 1 7 21 |
| mc18000244 | 6 7 13 21 | mc9000298 | 3 7 21 |
| mc18000246 | 6 7 21 | mc9000301 | 7 21 |
| mc18000259 | 21 | mc9000304 | 21 |
| mc18000270 | 3 7 21 | mc9000315 | 7 10 21 |
| mc18000272 | 10 | mc9000317 | 10 |
| mc18000274 | 4 7 15 16 21 | mc9000326 | 4 7 13 16 20 21 |
| mc18000275 | 4 7 21 | mc9000337 | 3 7 |
| mc18000284 | 7 9 16 21 | mc9000347 | 1 7 21 |
| mc18000285 | 4 7 8 10 21 | mc9000357 | 7 |
| mc18000292 | 4 7 21 | mc9000358 | 7 12 21 |
| mc18000329 | 1 6 7 10 21 | mc9000386 | 7 12 |
| mc18000337 | 7 10 22 | mc9000417 | 6 7 21 |
| mc18000338 | 7 9 12 13 21 24 | mc9000421 | 6 12 21 |
| mc18000341 | 9 | mc9000422 | 7 12 13 16 17 20 21 |
| mc18000345 | 4 7 21 | mc9000432 | 4 7 18 19 21 24 |
| mc18000347 | 21 | mc9000441 | 8 10 21 |
| mc18000349 | 7 21 | mc9000444 | 4 7 13 15 21 |
| mc18000350 | 2 7 9 17 21 | mc9000448 | 4 7 11 15 21 |
| mc18000351 | 1 2 9 | mc9000465 | 7 13 |
| mc18000352 | 6 7 13 21 | mc9000466 | 6 |
| mc18000355 | 1 6 7 10 | mc9000468 | 1 21 |
| mc18000356 | 3 4 7 8 21 | mc9000469 | 7 8 10 12 13 19 |
| mc18000357 | 3 7 9 15 16 21 | mc9000471 | 1 6 7 9 10 21 |
| mc18000361 | 15 21 | mc9000478 | 7 16 19 21 |
| mc18000363 | 21 | mc9000482 | 1 4 21 |
| mc18000364 | 4 6 7 10 13 20 21 22 24 | mc9000486 | 7 21 |
| mc18000366 | 1 7 9 21 | mc9000498 | 16 22 24 |
| mc18000369 | 18 19 20 22 23 | mc9000499 | 1 3 6 7 10 11 21 |
| mc18000375 | 7 17 21 | mc9000500 | 6 21 |
| mc18000384 | 3 7 10 11 16 21 | mc9000503 | 7 21 |
| mc18000385 | 6 7 9 10 21 | mc9000504 | 4 7 21 |
| mc18000386 | 9 10 | mc9000509 | 1 9 |
| mc18000387 | 7 9 | mc9000513 | 4 7 21 |
| mc18000389 | 10 16 21 | mc9000521 | 7 12 17 21 |
| mc18000390 | 1 7 15 16 | mc9000522 | 21 |
| mc18000392 | 3 4 15 21 | mc9000524 | 1 7 10 |
| mc18000394 | 2 3 7 9 21 | mc9000525 | 8 10 16 |
| mc18000395 | 3 6 7 21 | mc9000526 | 1 21 |
| mc18000396 | 7 10 12 16 20 21 | mc9000527 | 7 21 |
| mc18000397 | 1 3 6 7 8 10 21 | mc9000528 | 21 |
| mc18000401 | 3 4 7 15 21 | mc9000532 | 10 13 |
| mc18000403 | 5 7 8 21 | mc9000629 | 10 13 21 |
| mc18000404 | 6 7 11 15 21 | mc9000634 | 10 13 |
| mc18000410 | 21 | mc9000641 | 4 |
| mc18000414 | 7 21 | mc9000649 | 21 |
| mc18000415 | 5 | mc9000652 | 4 7 20 21 |
| mc18000420 | 1 3 6 7 11 | mc9000655 | 21 |
| mc18000427 | 7 | mc9000656 | 1 |
| mc18000437 | 3 4 7 21 | mc9000672 | 1 2 7 9 10 16 |
| mc18000440 | 6 7 17 21 | mc9000675 | 1 7 21 |
| mc18000441 | 7 10 13 15 21 | mc9000677 | 1 12 13 16 17 21 |
| mc18000442 | 9 | mc9000692 | 4 7 21 |
| mc18000446 | 5 7 | mc9000696 | 7 21 |
| mc18000447 | 4 7 8 21 | mc9000705 | 10 12 17 21 |
| mc18000448 | 15 | mc9000710 | 21 |
| mc18000451 | 13 15 21 22 | mc9000713 | 6 |
| mc18000453 | 2 7 9 21 | mc9000717 | 12 21 |
| mc18000454 | 13 15 18 21 | mc9000718 | 6 7 10 21 |
| mc18000456 | 7 16 18 | mc9000720 | 7 10 |
| mc18000457 | 5 7 21 | mc9000721 | 7 |
| mc18000491 | 7 | mc9000724 | 4 7 21 |
| mc18000503 | 7 12 21 | mc9000726 | 1 6 21 |
| mc18000522 | 7 12 | mc9000728 | 1 |
| mc18000528 | 4 7 10 21 | mc9000731 | 6 11 21 |
| mc18000565 | 7 12 21 | mc9000732 | 9 |
| mc18000619 | 7 10 17 21 | mc9000733 | 9 21 |
| mc18000620 | 3 4 6 7 21 | mc9000734 | 5 7 |
| mc18000622 | 9 | mc9000738 | 17 21 |
| mc18000624 | 7 8 10 12 15 21 | mc9000744 | 10 13 15 20 21 |
| mc18000630 | 7 | mc9000749 | 1 11 21 |
| mc18000632 | 7 12 21 | mc9000753 | 9 17 21 |
| mc18000633 | 4 6 7 21 | mc9000755 | 10 21 |
| mc18000634 | 1 16 | mc9000759 | 2 7 9 21 |
| mc18000639 | 8 10 16 | mc9000762 | 7 10 |
| mc18000643 | 3 7 10 15 16 20 21 | mc9000763 | 6 7 10 13 18 |
| mc18000645 | 8 | mc9000765 | 7 12 21 |
| mc18000649 | 4 7 10 18 21 | mc9000769 | 6 10 |
| mc18000660 | 21 | mc9000770 | 10 21 |
| mc18000684 | 4 7 21 | mc9000773 | 9 21 |
| mc18000685 | 10 | mc9000774 | 7 9 |
| mc18000699 | 6 8 21 | mc9000785 | 6 22 |
| mc18000700 | 7 21 | mc9000787 | 6 21 |
| mc18000706 | 6 | mc9000788 | 19 |
| mc18000709 | 10 | mc9000797 | 7 9 13 21 |
| mc18000711 | 7 19 21 | mc9000798 | 1 7 9 21 |
| mc18000728 | 1 6 7 16 21 | mc9000799 | 7 9 13 21 |
| mc18000731 | 1 6 7 | mc9000800 | 7 9 21 |
| mc18000737 | 4 7 | mc9000801 | 7 21 |
| mc18000738 | 7 10 13 15 | mc9000839 | 1 7 11 21 |
| mc18000740 | 5 7 9 | mc9000840 | 3 7 |
| mc18000741 | 1 7 9 21 | mc9000844 | 1 |
| mc18000742 | 6 7 17 21 | mc9000850 | 9 10 12 21 |
| mc18000743 | 6 9 21 | mc9000853 | 6 |
| mc18000753 | 10 15 16 | mc9000855 | 3 9 21 |
| mc18000780 | 15 | mc9000856 | 6 10 |
| mc18000782 | 1 2 13 21 | mc9000861 | 10 12 21 |
| mc18000786 | 1 | mc9000862 | 1 6 7 |
| mc18000797 | 7 9 12 21 | mc9000863 | 7 |
| mc18000798 | 21 | mc9000865 | 6 7 10 12 15 16 21 |
| mc18000799 | 6 7 21 | mc9000883 | 1 7 |
| mc18000801 | 21 | mc9000884 | 1 7 |
| mc18000808 | 7 9 12 21 | mc9000886 | 7 10 13 15 21 |
| mc18000819 | 6 7 21 | mc9000887 | 1 2 7 9 21 |
| mc18000868 | 1 7 9 13 21 | mc9000888 | 6 7 9 21 |
| mc18000869 | 9 21 | mc9000891 | 1 2 21 |
| mc18000872 | 7 21 | mc9000892 | 7 |
| mc18000890 | 7 12 15 16 17 21 | mc9000897 | 6 11 19 |
| mc18000897 | 1 | mc9000898 | 11 12 19 20 21 23 24 |
| mc18000898 | 6 7 21 24 | mc9000899 | 21 |
| mc18000901 | 7 | mc9000901 | 1 21 |
| mc18000914 | 7 17 21 | mc9000903 | 1 6 7 10 21 |
| mc18000917 | 7 23 | mc9000908 | 4 7 21 |
| mc18000925 | 5 7 21 | mc9000916 | 10 |
| mc18000927 | 6 7 9 11 13 18 21 | mc9000919 | 2 7 21 |
| mc18000928 | 1 4 9 21 | mc9000937 | 7 |
| mc18000933 | 9 12 21 23 | mc9000940 | 1 3 4 6 7 8 10 11 21 |
| mc18000934 | 4 7 16 21 | mc9000943 | 1 21 |
| mc18000935 | 1 6 7 10 21 | mc9000945 | 3 6 7 10 |
| mc18000937 | 9 | mc9000946 | 7 9 10 |
| mc18000940 | 7 | mc9000954 | 1 2 7 9 21 |
| mc18000949 | 7 12 13 15 21 | mc9000960 | 1 7 |
| mc18000953 | 7 17 | mc9000961 | 1 2 7 9 21 |
| mc18000957 | 7 10 13 21 | mc9000962 | 1 |
| mc18000962 | 8 10 12 13 21 23 | mc9000964 | 3 21 |
| mc18000964 | 6 10 21 | mc9000968 | 1 |
| mc18000986 | 2 9 | mc9000970 | 6 7 8 11 13 21 |
| mc18000995 | 1 6 21 | mc9000971 | 9 10 12 |
| mc18000998 | 4 7 21 | mc9000972 | 9 10 12 21 22 |
| mc18001001 | 1 7 21 | mc9000978 | 6 |
| mc18001002 | 4 5 7 21 | mc9000981 | 2 7 21 |
| mc18001003 | 1 7 9 | mc9000983 | 4 7 16 21 |
| mc18001011 | 6 7 | mc9000990 | 7 21 |
| mc18001015 | 6 10 15 | mc9000991 | 3 7 12 21 |
| mc18001020 | 6 7 21 | mc9000992 | 21 |
| mc18001021 | 10 21 | mc9000996 | 4 7 |
| mc18001022 | 4 7 16 20 21 | mc9001005 | 12 21 |
| mc18001023 | 7 9 12 21 | mc9001008 | 10 |
| mc18001024 | 6 7 9 21 | mc9001010 | 5 21 |
| mc18001029 | 15 21 | mc9001011 | 7 9 21 |
| mc18001033 | 7 10 13 | mc9001013 | 1 6 8 10 |
| mc18001037 | 9 | mc9001014 | 4 7 21 |
| mc18001038 | 7 10 13 21 | mc9001015 | 1 11 |
| mc18001041 | 1 7 10 21 | mc9001016 | 1 3 7 11 21 |
| mc18001043 | 15 21 | mc9001023 | 1 |
| mc18001048 | 9 21 | mc9001024 | 9 21 |
| mc18001052 | 3 15 21 | mc9001028 | 4 7 21 |
| mc18001053 | 1 6 8 10 | mc9001029 | 2 7 9 21 |
| mc18001071 | 1 4 7 21 | mc9001036 | 6 7 8 10 20 21 |
| mc18001072 | 10 | mc9001039 | 7 9 11 21 |
| mc18001073 | 10 | mc9001041 | 1 6 7 10 21 |
| mc18001088 | 4 7 8 16 21 | mc9001043 | 7 |
| mc18001111 | 1 3 7 11 21 | mc9001045 | 6 9 |
| mc18001112 | 3 4 7 21 | mc9001046 | 10 15 16 21 |
| mc18001119 | 3 10 15 16 21 22 | mc9001048 | 1 7 9 21 |
| mc18001144 | 4 7 8 10 16 21 | mc9001053 | 7 12 21 |
| mc18001146 | 1 3 4 7 21 | mc9001054 | 7 12 |
| mc18001147 | 2 7 21 | mc9001058 | 9 |
| mc18001150 | 21 | mc9001059 | 6 7 9 21 |
| mc18001151 | 1 3 6 7 10 | mc9001068 | 10 |
| mc18001159 | 4 7 21 | mc9001073 | 1 4 6 7 10 12 16 |
| mc18001161 | 3 4 7 21 | mc9001078 | 1 2 7 |
| mc18001166 | 2 7 9 17 21 | mc9001079 | 12 13 |
| mc18001168 | 1 2 21 | mc9001080 | 6 7 21 |
| mc18001172 | 1 7 9 21 | mc9001083 | 1 7 11 13 16 17 20 21 |
| mc18001182 | 7 8 10 21 | mc9001089 | 6 |
| mc18001194 | 4 7 8 10 16 21 | mc9001093 | 10 21 |
| mc18001202 | 21 | mc9001103 | 7 |
| mc18001205 | 7 | mc9001104 | 7 8 9 11 13 15 21 |
| mc18001207 | 4 6 7 16 21 | mc9001119 | 5 7 21 |
| mc18001210 | 1 6 21 | mc9001121 | 2 7 9 21 |
| mc18001221 | 3 | mc9001124 | 1 15 21 |
| mc18001222 | 2 7 9 21 | mc9001127 | 16 21 |
| mc18001223 | 21 | mc9001132 | 3 7 |
| mc18001231 | 7 9 | mc9001134 | 7 10 12 |
| mc18001261 | 3 7 10 17 | mc9001135 | 4 7 10 |
| mc18001272 | 1 6 7 21 | mc9001138 | 7 |
| mc18001280 | 3 4 7 9 19 20 21 | mc9001139 | 4 6 7 8 10 21 |
| mc18001281 | 1 7 9 | mc9001142 | 4 7 8 21 |
| mc18001305 | 10 | mc9001144 | 1 6 7 10 19 20 21 |
| mc18001308 | 7 16 | mc9001155 | 7 10 16 21 |
| mc18001314 | 4 7 21 | mc9001158 | 1 2 21 |
| mc18001333 | 1 6 7 9 | mc9001159 | 21 |
| mc18001348 | 6 21 | mc9001160 | 1 5 7 21 |
| mc18001350 | 6 7 21 | mc9001161 | 4 7 8 21 |
| mc18001356 | 1 2 21 | mc9001164 | 7 21 |
| mc18001366 | 6 9 10 | mc9001172 | 4 7 9 10 |
| mc18001381 | 7 10 15 | mc9001178 | 7 10 |
| mc18001383 | 16 | mc9001180 | 6 |
| mc18001385 | 10 12 13 15 | mc9001181 | 7 12 21 |
| mc18001395 | 2 6 9 21 | mc9001182 | 12 21 |
| mc18001398 | 4 7 9 10 | mc9001184 | 21 |
| mc18001399 | 7 8 10 21 | mc9001185 | 6 7 9 21 |
| mc18001400 | 7 21 | mc9001186 | 4 15 21 |
| mc18001401 | 7 12 18 20 21 | mc9001191 | 4 21 |
| mc18001402 | 7 | mc9001192 | 6 7 13 21 |
| mc18001405 | 7 10 15 | mc9001197 | 1 7 8 |
| mc18001406 | 1 3 6 7 10 21 | mc9001200 | 4 7 8 10 21 |
| mc18001407 | 6 7 11 21 | mc9001202 | 1 6 7 10 |
| mc19000001 | 1 5 6 7 21 | mc9001205 | 9 10 21 |
| mc19000004 | 2 10 11 21 | mc9001206 | 21 |
| mc19000006 | 4 7 9 10 21 | mc9001213 | 6 7 |
| mc19000008 | 7 9 21 | mc9001214 | 1 2 7 |
| mc19000010 | 7 15 21 | mc9001218 | 6 7 8 10 21 |
| mc19000011 | 1 7 8 10 21 | mc9001222 | 6 11 21 |
| mc19000012 | 8 | mc9001245 | 4 7 10 21 |
| mc19000014 | 3 7 15 16 20 21 | mc9001270 | 10 21 |
| mc19000015 | 6 7 11 21 | mc9001272 | 7 8 13 17 20 21 |
| mc19000017 | 1 4 | mc9001275 | 4 7 21 |
| mc19000018 | 7 12 21 | mc9001280 | 15 21 |
| mc19000019 | 6 11 21 | mc9001281 | 4 5 7 21 |
| mc19000020 | 21 | mc9001282 | 1 6 |
| mc19000024 | 6 13 | mc9001288 | 3 8 21 |
| mc19000025 | 2 7 9 21 24 | mc9001289 | 1 7 16 21 |
| mc19000028 | 4 7 8 21 | mc9001293 | 6 7 10 21 |
| mc19000038 | 3 | mc9001294 | 1 21 24 |
| mc19000039 | 7 15 21 | mc9001299 | 9 21 |
| mc19000042 | 9 | mc9001303 | 10 |
| mc19000046 | 9 21 | mc9001310 | 4 7 16 19 21 |
| mc19000050 | 1 7 8 10 | mc9001323 | 21 |
| mc19000052 | 4 7 21 | mc9001337 | 7 9 10 21 |
| mc19000053 | 1 6 7 10 | mc9001339 | 5 7 21 |
| mc19000054 | 3 4 6 7 17 21 | mc9001368 | 7 9 21 |
| mc19000058 | 7 21 | mc9001373 | 15 21 |
| mc19000059 | 10 21 | mc9001380 | 7 10 12 13 17 21 |
| mc19000065 | 7 9 10 | mc9001382 | 21 |
| mc19000066 | 9 10 21 | mc9001384 | 7 10 15 16 20 21 |
| mc19000067 | 3 17 21 | mc9001386 | 1 21 |
| mc19000070 | 3 21 | mc9001388 | 10 |
| mc19000076 | 5 6 7 21 | mc9001393 | 6 21 |
| mc19000077 | 5 21 | mc9001405 | 1 |
| mc19000078 | 6 7 11 15 16 21 | mc9001409 | 1 24 |
| mc19000079 | 6 7 16 19 21 | mc9001410 | 4 7 16 21 |
| mc19000080 | 9 | mc9001412 | 6 7 21 |
| mc19000081 | 3 7 21 | mc9001415 | 11 21 |
| mc19000082 | 10 15 21 | mc9001416 | 1 11 |
| mc19000083 | 4 7 15 21 | mc9001428 | 4 5 21 |
| mc19000085 | 9 21 | mc9001432 | 9 21 |
| mc19000086 | 21 | mc9001433 | 8 10 |
| mc19000089 | 6 7 17 21 | mc9001443 | 6 7 21 |
| mc19000090 | 7 10 20 21 | mc9001445 | 2 7 9 21 24 |
| mc19000091 | 3 | mc9001459 | 2 7 9 13 16 17 21 |
| mc19000095 | 1 6 7 10 | mc9001461 | 10 |
| mc19000098 | 3 7 8 21 | mc9001471 | 10 |
| mc19000099 | 4 7 10 21 | mc9001477 | 1 7 10 |
| mc19000100 | 3 4 7 17 21 | mc9001492 | 1 21 |
| mc19000103 | 3 4 7 16 19 21 | mc9001493 | 1 6 7 10 |
| mc19000108 | 3 7 21 | mc9001494 | 1 21 |
| mc19000109 | 7 10 13 21 | mc9001514 | 12 16 21 |
| mc19000110 | 6 21 | mc9001523 | 1 2 7 |
| mc19000111 | 3 7 10 21 | mc9001527 | 6 21 |
| mc19000113 | 4 7 8 12 13 21 | mc9001528 | 6 7 9 21 |
| mc19000116 | 4 7 8 21 | mc9001531 | 1 7 9 11 21 |
| mc19000119 | 4 13 18 21 | mc9001539 | 4 7 16 21 |
| mc19000121 | 3 7 21 | mc9001549 | 1 7 21 |
| mc19000122 | 8 19 21 | mc9001550 | 9 10 |
| mc19000123 | 9 12 16 21 22 23 24 | mc9001559 | 9 21 |
| mc19000126 | 4 6 7 21 | mc9001566 | 7 8 10 13 24 |
| mc19000132 | 10 21 | mc9001567 | 6 21 |
| mc19000135 | 7 21 | mc9001568 | 3 4 7 15 21 |
| mc19000136 | 8 9 10 21 | mc9001582 | 7 21 |
| mc19000138 | 1 21 | mc9001591 | 7 |
| mc19000139 | 9 21 | mc9001593 | 7 |
| mc19000141 | 4 7 21 22 23 24 | mc9001595 | 1 6 7 8 21 |
| mc19000142 | 5 21 | mc9001620 | 1 6 13 21 |
| mc19000144 | 6 7 21 | mc9001623 | 7 |
| mc19000150 | 6 7 9 11 12 | mc9001627 | 7 21 |
| mc19000151 | 4 7 21 | mc9001628 | 9 10 12 21 |
| mc19000152 | 4 7 21 | mc9001633 | 1 21 |
| mc19000157 | 21 | mc9001634 | 1 |
| mc19000158 | 1 2 7 21 | mc9001636 | 7 9 21 |
| mc19000164 | 7 9 21 | mc9001645 | 7 8 10 |
| mc19000165 | 7 10 | mc9001646 | 7 21 |
| mc19000168 | 6 7 8 10 21 | mc9001664 | 7 9 12 21 |
| mc19000170 | 1 6 8 10 21 | mc9001673 | 1 21 |
| mc19000171 | 1 3 7 21 | mc9001675 | 7 9 21 |
| mc19000172 | 10 15 18 21 | mc9001677 | 7 9 17 21 |
| mc19000173 | 3 7 11 21 | mc9001678 | 5 21 |
| mc19000174 | 1 2 3 21 | mc9001691 | 4 7 21 |
| mc19000175 | 6 9 | mc9001694 | 15 |
| mc19000177 | 1 6 7 10 | mc9001697 | 10 16 21 |
| mc19000178 | 7 8 10 21 | mc9001704 | 7 10 |
| mc19000179 | 7 10 12 | mc9001707 | 21 |
| mc19000181 | 7 10 | mc9001708 | 4 21 |
| mc19000183 | 7 8 10 12 21 | mc9001710 | 7 17 21 23 24 |
| mc19000184 | 7 9 | mc9001716 | 7 |
| mc19000186 | 7 10 | mc9001729 | 3 7 17 21 |
| mc19000188 | 11 21 | mc9001730 | 1 21 |
| mc19000190 | 2 7 9 21 | mc9001755 | 16 19 |
| mc19000192 | 1 7 21 | mc9001758 | 3 6 7 21 |
| mc19000193 | 4 7 21 | mc9001761 | 7 12 |
| mc19000194 | 1 | mc9001764 | 1 6 7 10 16 21 |
| mc19000201 | 1 | mc9001767 | 9 21 |
| mc19000202 | 2 7 9 21 | mc9001771 | 7 10 21 |
| mc19000203 | 2 9 21 | mc9001772 | 7 9 12 21 |
| mc19000204 | 1 7 | mc9001774 | 7 9 12 21 |
| mc19000207 | 6 17 21 | mc9001776 | 3 7 11 21 |
| mc19000210 | 1 7 9 10 16 21 | mc9001792 | 6 |
| mc19000214 | 10 16 21 | mc9001793 | 9 21 |
| mc19000215 | 15 16 21 | mc9001794 | 10 15 |
| mc19000217 | 1 2 20 21 | mc9001803 | 5 21 |
| mc19000220 | 3 4 7 21 22 | mc9001805 | 7 |
| mc19000226 | 7 9 | mc9001807 | 1 3 6 7 10 21 |
| mc19000227 | 7 9 21 | mc9001809 | 1 7 9 21 |
| mc19000231 | 1 7 11 21 | mc9001822 | 21 |
| mc19000234 | 7 9 21 | mc9001826 | 10 12 13 21 |
| mc19000235 | 7 9 21 | mc9001827 | 1 7 21 |
| mc19000238 | 4 7 13 21 | mc9001829 | 1 6 8 10 |
| mc19000239 | 4 7 21 | mc9001830 | 5 7 21 |
| mc19000240 | 4 7 21 | mc9001831 | 1 7 21 |
| mc19000251 | 1 21 | mc9001833 | 21 |
| mc19000252 | 1 7 21 | mc9001834 | 7 |
| mc19000253 | 7 12 13 21 | mc9001836 | 3 7 21 |
| mc19000255 | 3 4 6 7 21 | mc9001837 | 4 7 21 |
| mc19000256 | 7 | mc9001844 | 3 4 7 8 10 11 21 |
| mc19000258 | 7 8 21 | mc9001850 | 21 |
| mc19000259 | 1 6 | mc9001851 | 7 8 21 |
| mc19000269 | 3 7 21 | mc9001855 | 7 8 10 15 21 |
| mc19000275 | 1 | mc9001856 | 1 3 6 7 21 |
| mc19000277 | 1 | mc9001859 | 9 10 12 13 21 23 24 |
| mc19000278 | 1 3 7 21 | mc9001860 | 21 |
| mc19000288 | 7 | mc9001861 | 2 7 9 |
| mc19000292 | 1 | mc9001862 | 3 |
| mc19000293 | 3 7 11 21 | mc9001863 | 7 |
| mc19000295 | 6 21 | mc9001865 | 10 |
| mc19000299 | 10 13 15 21 | mc9001867 | 1 10 11 |
| mc19000302 | 6 7 10 12 21 | mc9001868 | 1 11 |
| mc19000303 | 9 | mc9001870 | 1 21 |
| mc19000306 | 3 6 7 11 21 | mc9001871 | 3 15 16 |
| mc19000307 | 16 21 | mc9001873 | 10 15 16 21 |
| mc19000308 | 8 10 12 21 | mc9001875 | 7 9 21 |
| mc19000319 | 10 11 13 21 | mc9001877 | 21 |
| mc19000326 | 21 | mc9001879 | 7 21 |
| mc19000332 | 10 13 21 | mc9001880 | 7 21 |
| mc19000334 | 21 | mc9001883 | 1 6 7 10 16 21 |
| mc19000336 | 7 9 21 | mc9001885 | 1 6 7 10 |
| mc19000338 | 5 21 | mc9001886 | 6 7 10 21 |
| mc19000339 | 7 9 21 | mc9001892 | 1 10 21 |
| mc19000345 | 10 13 | mc9001893 | 1 |
| mc19000355 | 10 13 | mc9001895 | 7 12 21 |
| mc19000367 | 7 | mc9001897 | 6 7 13 16 21 |
| mc19000381 | 6 7 10 12 21 | mc9001898 | 6 |
| mc19000418 | 10 13 | mc9001899 | 7 21 |
| mc19000420 | 10 13 | mc9001904 | 21 |
| mc19000433 | 4 7 8 10 21 | mc9001908 | 7 11 21 |
| mc19000473 | 4 7 21 | mc9001909 | 6 21 |
| mc19000483 | 1 7 | mc9001915 | 6 |
| mc19000496 | 4 7 | mc9001919 | 21 |
| mc19000497 | 1 7 | mc9001924 | 6 7 21 |
| mc19000500 | 1 6 7 9 | mc9001925 | 6 7 21 |
| mc19000502 | 4 7 10 21 | mc9001927 | 9 21 |
| mc19000513 | 3 7 8 9 10 21 | mc9001932 | 7 10 12 16 21 |
| mc19000521 | 13 | mc9001935 | 2 7 9 21 |
| mc19000526 | 7 | mc9001936 | 7 12 21 |
| mc19000528 | 1 7 21 | mc9001938 | 15 21 |
| mc19000547 | 4 7 10 16 21 | mc9001941 | 3 11 21 |
| mc19000548 | 7 17 21 | mc9001944 | 10 21 |
| mc19000563 | 7 17 21 | mc9001946 | 6 |
| mc19000566 | 7 12 21 | mc9001959 | 1 7 21 |
| mc19000578 | 1 6 7 12 15 21 | mc9001960 | 11 13 21 |
| mc19000580 | 4 7 21 | mc9001961 | 1 3 6 8 10 |
| mc19000594 | 4 7 15 16 21 | mc9001963 | 3 4 7 |
| mc19000621 | 1 7 9 10 21 | mc9001964 | 3 4 7 16 20 21 |
| mc19000622 | 9 | mc9001966 | 12 15 16 21 |
| mc19000625 | 7 21 | mc9001971 | 8 11 21 |
| mc19000632 | 4 7 16 21 | mc9001973 | 2 7 9 11 17 21 |
| mc19000639 | 4 7 21 | mc9001975 | 10 15 21 |
| mc19000651 | 9 12 21 24 | mc9001979 | 10 16 20 |
| mc19000655 | 3 7 21 | mc9001982 | 6 |
| mc19000656 | 1 7 21 | mc9001983 | 6 |
| mc19000657 | 4 21 | mc9001984 | 6 |
| mc19000659 | 6 10 21 | mc9001986 | 6 |
| mc19000660 | 6 10 13 15 21 24 | mc9001987 | 6 |
| mc19000663 | 10 21 | mc9001988 | 13 20 |
| mc19000664 | 7 10 | mc9001989 | 7 8 17 |
| mc19000677 | 21 | mc9001994 | 7 9 12 21 |
| mc19000680 | 3 4 6 7 21 | mc9001995 | 10 15 |
| mc19000681 | 1 7 21 | mc9001998 | 3 7 11 15 21 |
| mc19000682 | 7 9 13 21 | mc9001999 | 7 21 |
| mc19000703 | 1 15 21 | mc9002002 | 1 6 7 |
| mc19000704 | 1 21 | mc9002003 | 7 10 |
| mc19000713 | 1 21 | mc9002011 | 8 21 |
| mc19000717 | 7 21 | mc9002035 | 9 10 15 21 |
| mc19000720 | 1 3 6 7 10 15 | mc9002040 | 20 |
| mc19000731 | 1 21 | mc9002041 | 6 |
| mc19000735 | 4 7 | mc9002045 | 7 21 |
| mc19000738 | 6 7 21 | mc9002047 | 10 12 13 15 |
| mc19000740 | 7 10 15 21 | mc9002051 | 2 3 7 12 17 21 |
| mc19000742 | 1 | mc9002052 | 12 13 15 21 |
| mc19000743 | 1 21 | mc9002054 | 12 13 15 21 |
| mc19000745 | 7 13 | mc9002079 | 6 7 10 16 20 21 |
| mc19000747 | 7 13 | mc9002084 | 7 21 |
| mc19000752 | 7 13 | mc9002106 | 10 21 |
| mc19000763 | 10 | mc9002111 | 4 7 15 16 21 |
| mc19000766 | 4 21 | mc9002112 | 21 |
| mc19000767 | 4 7 8 21 | mc9002113 | 7 10 12 |
| mc19000772 | 7 | mc9002115 | 1 21 |
| mc19000776 | 1 2 6 7 8 9 | mc9002117 | 17 |
| mc19000779 | 13 21 22 | mc9002118 | 1 7 10 21 |
| mc19000787 | 6 7 21 | mc9002124 | 9 21 |
| mc19000790 | 2 3 7 9 17 21 | mc9002127 | 7 10 13 |
| mc19000791 | 4 7 15 16 20 21 | mc9002128 | 1 2 21 24 |
| mc19000793 | 6 7 9 19 21 | mc9002129 | 1 6 7 8 10 21 |
| mc19000795 | 1 2 7 9 21 | mc9002131 | 1 |
| mc19000802 | 7 10 | mc9002132 | 7 9 21 |
| mc19000803 | 7 9 21 | mc9002134 | 7 9 21 |
| mc19000804 | 1 10 13 | mc9002136 | 7 9 21 |
| mc19000812 | 10 | mc9002139 | 7 9 21 |
| mc19000814 | 21 | mc9002141 | 15 21 |
| mc19000817 | 3 4 7 15 18 19 20 21 | mc9002143 | 10 15 |
| mc19000825 | 1 2 7 9 21 | mc9002144 | 7 10 11 12 15 |
| mc19000831 | 1 7 9 11 21 | mc9002145 | 9 21 |
| mc19000833 | 4 7 8 21 | mc9002146 | 5 7 10 19 20 21 |
| mc19000837 | 1 7 21 | mc9002148 | 18 21 |
| mc19000845 | 7 10 13 21 23 | mc9002152 | 7 9 10 |
| mc19000851 | 10 11 13 21 | mc9002158 | 1 21 |
| mc19000853 | 1 3 7 21 | mc9002159 | 5 7 21 |
| mc19000857 | 1 7 10 21 | mc9002165 | 4 7 8 9 10 12 16 17 18 21 |
| mc19000866 | 7 12 15 16 17 21 | mc9002177 | 13 16 24 |
| mc19000873 | 1 2 21 | mc9002181 | 10 |
| mc19000874 | 1 3 4 7 21 | mc9002182 | 6 9 21 |
| mc19000875 | 1 7 15 21 | mc9002184 | 6 7 |
| mc19000876 | 3 4 7 11 21 | mc9002190 | 7 10 13 21 24 |
| mc19000878 | 7 17 21 | mc9002191 | 1 2 7 9 21 |
| mc19000887 | 10 15 16 21 | mc9002201 | 1 6 7 21 |
| mc19000894 | 2 6 7 9 10 21 | mc9002215 | 7 21 |
| mc19000897 | 6 7 17 18 21 | mc9002217 | 2 7 9 17 21 |
| mc19000909 | 1 7 21 | mc9002221 | 1 6 |
| mc19000911 | 4 7 16 18 20 21 24 | mc9002223 | 1 6 7 9 21 |
| mc19000914 | 9 21 | mc9002226 | 7 20 21 |
| mc19000917 | 1 21 | mc9002232 | 5 21 |
| mc19000918 | 1 2 6 7 21 | mc9002233 | 7 |
| mc19000919 | 7 | mc9002234 | 1 |
| mc19000920 | 1 7 9 21 | mc9002242 | 10 15 |
| mc19000921 | 7 8 10 21 | mc9002245 | 10 |
| mc19000923 | 6 7 | mc9002251 | 7 10 11 12 15 |
| mc19000926 | 6 7 8 10 | mc9002253 | 7 10 11 12 13 15 19 |
| mc19000928 | 1 7 11 12 | mc9002267 | 7 9 |
| mc19000938 | 2 10 21 | mc9002268 | 3 4 7 21 |
| mc19000940 | 2 7 9 21 | mc9002275 | 4 6 7 17 21 |
| mc19000941 | 1 4 7 21 | mc9002277 | 7 10 21 |
| mc19000946 | 4 7 15 16 20 21 | mc9002279 | 7 21 |
| mc19000952 | 5 21 | mc9002281 | 1 7 9 |
| mc19000953 | 3 17 21 | mc9002284 | 1 21 |
| mc19000954 | 3 21 | mc9002285 | 1 6 7 10 21 |
| mc19000957 | 7 15 16 20 21 | mcx000002 | 3 21 |
| mc19000959 | 4 7 15 16 18 20 21 | mcx000014 | 4 7 13 |
| mc19000961 | 4 7 21 | mcx000016 | 7 9 10 12 13 21 24 |
| mc19000967 | 6 7 21 | mcx000017 | 7 9 12 21 24 |
| mc19000970 | 1 3 7 11 21 | mcx000018 | 7 |
| mc19000972 | 3 4 6 7 21 | mcx000028 | 7 9 21 |
| mc19000974 | 3 7 10 15 16 18 20 21 | mcx000030 | 1 6 7 15 |
| mc19000975 | 4 6 7 11 21 | mcx000033 | 9 21 |
| mc19000976 | 4 6 21 | mcx000034 | 4 7 8 21 |
| mc19000981 | 6 7 9 17 18 | mcx000035 | 6 7 9 21 |
| mc19000982 | 4 7 8 10 16 21 | mcx000036 | 6 8 21 |
| mc19000986 | 6 7 9 17 21 | mcx000037 | 7 |
| mc19000988 | 1 21 | mcx000038 | 3 4 6 7 17 21 |
| mc19000989 | 4 7 16 20 21 | mcx000040 | 4 7 21 |
| mc19000994 | 4 7 10 20 21 | mcx000041 | 1 |
| mc19000997 | 6 | mcx000043 | 1 3 6 7 17 21 |
| mc19000998 | 21 | mcx000044 | 7 9 13 17 21 |
| mc19001002 | 21 | mcx000047 | 21 |
| mc19001003 | 4 7 21 | mcx000050 | 1 19 21 |
| mc19001004 | 7 10 21 | mcx000053 | 1 7 10 21 |
| mc19001005 | 9 21 | mcx000077 | 15 21 |
| mc19001007 | 1 7 9 13 16 21 | mcx000097 | 7 9 12 |
| mc19001010 | 1 11 21 | mcx000108 | 9 21 |
| mc19001014 | 1 21 | mcx000114 | 7 9 21 |
| mc19001016 | 7 15 16 17 21 | mcx000121 | 7 9 21 |
| mc19001017 | 4 6 7 21 | mcx000123 | 7 8 12 13 20 21 |
| mc19001018 | 4 7 21 | mcx000124 | 1 7 21 |
| mc19001020 | 4 15 21 | mcx000126 | 7 12 13 15 18 21 |
| mc19001026 | 10 | mcx000131 | 7 17 21 |
| mc19001027 | 3 7 | mcx000156 | 9 10 |
| mc19001029 | 1 3 6 7 10 | mcx000160 | 4 |
| mc19001030 | 7 9 12 21 | mcx000166 | 6 7 21 |
| mc19001034 | 11 21 | mcx000171 | 3 4 7 21 |
| mc19001041 | 9 21 | mcx000178 | 12 13 21 |
| mc19001076 | 6 7 | mcx000180 | 6 7 10 12 17 21 24 |
| mc19001082 | 6 7 21 | mcx000181 | 10 |
| mc19001087 | 4 7 21 | mcx000183 | 10 |
| mc19001088 | 4 6 7 15 21 | mcx000202 | 1 21 |
| mc19001096 | 3 7 21 | mcx000204 | 21 |
| mc19001100 | 13 15 21 | mcx000219 | 7 21 |
| mc19001102 | 7 10 21 | mcx000229 | 1 6 21 |
| mc19001104 | 7 10 13 20 21 | mcx000230 | 9 12 21 |
| mc19001113 | 1 21 | mcx000232 | 1 6 7 |
| mc19001115 | 13 17 21 | mcx000234 | 7 8 11 15 |
| mc19001119 | 9 | mcx000235 | 2 7 9 21 |
| mc19001133 | 6 | mcx000237 | 6 7 8 |
| mc19001135 | 6 8 15 21 | mcx000238 | 1 6 7 10 |
| mc19001138 | 1 3 11 21 | mcx000240 | 6 21 |
| mc19001139 | 2 9 11 | mcx000243 | 7 9 12 17 23 |
| mc19001141 | 7 10 21 | mcx000244 | 8 18 21 |
| mc19001147 | 7 21 | mcx000247 | 3 4 7 21 |
| mc19001148 | 7 21 | mcx000248 | 6 7 21 |
| mc19001151 | 7 12 16 17 21 | mcx000261 | 7 8 10 13 15 |
| mc19001156 | 1 | mcx000264 | 9 21 |
| mc19001174 | 10 15 16 21 | mcx000286 | 10 21 |
| mc19001185 | 1 13 21 | mcx000287 | 7 |
| mc19001195 | 4 7 12 15 16 20 21 | mcx000293 | 10 11 21 24 |
| mc19001198 | 9 21 | mcx000301 | 4 7 21 |
| mc19001205 | 4 7 16 18 20 21 | mcx000313 | 2 7 9 21 |
| mc19001220 | 10 13 | mcx000322 | 4 7 21 |
| mc19001226 | 5 21 | mcx000336 | 4 7 21 |
| mc19001229 | 9 12 21 | mcx000340 | 9 21 |
| mc19001230 | 10 11 21 | mcx000343 | 5 7 21 |
| mc19001232 | 1 6 7 10 21 | mcx000344 | 3 |
| mc19001236 | 10 21 | mcx000345 | 3 |
| mc19001241 | 6 21 | mcx000346 | 1 |
| mc19001243 | 5 7 8 21 | mcx000370 | 6 7 15 21 |
| mc19001246 | 7 9 17 21 | mcx000374 | 10 13 |
| mc19001252 | 9 | mcx000375 | 6 21 |
| mc19001254 | 10 | mcx000404 | 7 10 21 |
| mc19001257 | 7 9 21 | mcx000407 | 1 7 21 |
| mc19001260 | 7 17 21 23 | mcx000414 | 4 7 |
| mc19001261 | 5 7 21 | mcx000415 | 4 7 21 |
| mc2000005 | 1 6 21 | mcx000417 | 1 2 7 9 15 21 |
| mc2000008 | 1 | mcx000418 | 7 9 10 |
| mc2000009 | 3 7 21 | mcx000421 | 2 9 13 21 |
| mc2000011 | 1 3 7 11 13 17 18 21 | mcx000422 | 9 10 21 |
| mc2000014 | 1 3 6 7 21 | mcx000423 | 7 |
| mc2000015 | 6 7 11 | mcx000427 | 2 7 9 12 15 |
| mc2000042 | 1 7 21 | mcx000454 | 7 10 21 |
| mc2000046 | 7 13 15 21 22 23 24 | mcx000458 | 21 |
| mc2000061 | 3 24 | mcx000463 | 6 |
| mc2000062 | 1 7 | mcx000468 | 21 |
| mc2000063 | 6 9 21 | mcx000471 | 15 21 |
| mc2000071 | 1 2 21 | mcx000484 | 4 7 21 |
| mc2000074 | 8 | mcx000485 | 1 7 13 15 21 |
| mc2000101 | 1 4 7 15 16 21 | mcx000487 | 21 |
| mc2000106 | 2 7 9 21 | mcx000491 | 7 |
| mc2000107 | 5 7 | mcx000510 | 7 19 23 |
| mc2000108 | 1 8 21 | mcx000515 | 10 |
| mc2000125 | 6 7 8 10 23 | mcx000516 | 4 5 7 21 |
| mc2000133 | 4 7 21 | mcx000517 | 4 21 |
| mc2000134 | 7 10 13 15 21 | mcx000519 | 7 10 11 13 15 21 |
| mc2000136 | 10 21 | mcx000520 | 8 |
| mc2000137 | 3 4 6 7 21 | mcx000521 | 7 21 |
| mc2000145 | 7 10 12 13 | mcx000539 | 7 10 15 |
| mc2000153 | 1 7 | mcx000546 | 8 16 |
| mc2000155 | 10 15 | mcx000558 | 3 4 7 15 18 20 21 |
| mc2000160 | 1 3 7 21 | mcx000586 | 6 9 16 |
| mc2000162 | 1 6 21 | mcx000608 | 7 9 20 21 |
| mc2000167 | 6 10 21 | mcx000615 | 4 13 |
| mc2000170 | 7 9 10 21 | mcx000620 | 1 21 |
| mc2000179 | 6 9 10 12 13 | mcx000638 | 1 6 8 10 16 19 |
| mc2000182 | 7 10 | mcx000640 | 1 6 10 21 |
| mc2000190 | 10 16 21 | mcx000641 | 21 |
| mc2000204 | 4 7 21 | mcx000646 | 6 10 21 |
| mc2000205 | 4 5 6 7 11 21 | mcx000651 | 7 9 12 13 |
| mc2000224 | 11 21 | mcx000653 | 24 |
| mc2000227 | 4 7 16 18 21 | mcx000654 | 9 10 12 21 |
| mc2000268 | 1 2 4 6 7 9 12 13 21 | mcx000656 | 9 10 12 21 23 24 |
| mc2000271 | 10 21 | mcx000657 | 9 10 12 21 |
| mc2000274 | 7 8 15 17 | mcx000660 | 1 |
| mc2000277 | 7 | mcx000661 | 7 |
| mc2000280 | 2 6 7 17 21 | mcx000670 | 4 7 21 |
| mc2000284 | 7 | mcx000675 | 21 |
| mc2000290 | 1 12 | mcx000680 | 1 |
| mc2000297 | 7 10 13 15 21 | mcx000682 | 7 9 21 |
| mc2000298 | 7 10 13 15 21 | mcx000683 | 7 9 13 15 21 |
| mc2000299 | 7 8 10 13 15 21 | mcx000685 | 10 16 |
| mc2000300 | 1 7 9 10 13 15 21 | mcx000686 | 1 6 7 |
| mc2000303 | 1 4 7 15 16 20 21 | mcx000687 | 1 2 7 9 21 |
| mc2000306 | 7 9 10 12 13 21 24 | mcx000688 | 6 7 21 |
| mc2000307 | 4 7 21 | mcx000690 | 7 12 15 16 21 |
| mc2000309 | 7 | mcx000691 | 10 |
| mc2000317 | 11 | mcx000692 | 10 17 21 |
| mc2000322 | 4 21 | mcx000693 | 1 3 6 7 17 21 |
| mc2000326 | 9 21 | mcx000694 | 1 7 |
| mc2000328 | 2 9 | mcx000695 | 3 21 |
| mc2000329 | 7 | mcx000698 | 1 6 7 10 15 |
| mc2000332 | 21 | mcx000699 | 4 7 16 21 |
| mc2000333 | 3 6 7 21 | mcx000700 | 6 10 12 13 |
| mc2000336 | 7 9 10 12 13 21 22 24 | mcx000705 | 21 |
| mc2000338 | 6 21 | mcx000707 | 5 21 |
| mc2000341 | 1 7 10 13 21 | mcx000708 | 1 3 |
| mc2000342 | 21 | mcx000709 | 1 16 19 |
| mc2000343 | 1 6 21 | mcx000711 | 1 2 7 9 21 |
| mc2000344 | 1 7 9 21 | mcx000712 | 8 9 |
| mc2000347 | 1 7 9 21 | mcx000716 | 6 |
| mc2000349 | 6 | mcx000719 | 1 2 |
| mc2000350 | 7 8 10 13 15 21 | mcx000753 | 7 12 13 21 |
| mc2000352 | 4 7 15 16 21 | mcx000755 | 8 15 21 |
| mc2000358 | 7 9 21 | mcx000756 | 6 7 8 11 21 |
| mc2000359 | 7 9 21 | mcx000758 | 6 7 17 21 |
| mc2000360 | 1 7 21 | mcx000761 | 7 17 21 |
| mc2000362 | 7 9 | mcx000784 | 7 8 21 |
| mc2000363 | 7 9 | mcx000788 | 1 6 7 10 21 |
| mc2000364 | 3 4 7 21 | mcx000789 | 7 9 |
| mc2000370 | 7 9 21 | mcx000821 | 21 23 24 |
| mc2000374 | 7 9 | mcx000829 | 7 17 19 21 |
| mc2000379 | 10 15 21 22 | mcx000845 | 4 7 8 10 16 21 |
| mc2000383 | 4 7 21 | mcx000909 | 4 7 12 16 20 21 |
| mc2000388 | 8 10 | mcx000910 | 3 7 21 |
| mc2000391 | 4 7 8 21 | mcx000913 | 1 16 20 |
| mc2000393 | 13 | mcx000920 | 4 7 21 |
| mc2000394 | 6 7 21 | mcx000921 | 5 7 |
| mc2000395 | 1 10 21 | mcx000949 | 4 7 10 |
| mc2000399 | 3 4 7 10 15 16 18 19 20 21 | mcx000971 | 10 15 16 |
| mc2000402 | 1 7 21 24 | mcx000974 | 4 7 10 16 20 21 |
| mc2000408 | 1 | mcx000975 | 7 8 17 21 |
| mc2000409 | 7 21 | mcx000982 | 7 12 15 16 21 |
| mc2000410 | 21 | mcx000995 | 7 9 12 13 15 16 20 21 |
| mc2000411 | 5 21 | mcx001002 | 7 9 10 |
| mc2000412 | 8 21 | mcx001003 | 10 12 13 |
| mc2000414 | 21 | mcx001004 | 6 7 10 |
| mc2000417 | 6 7 | mcx001005 | 3 7 21 |
| mc2000419 | 9 21 | mcx001010 | 9 21 |
| mc2000425 | 1 7 12 13 15 20 | mcx001011 | 9 10 |
| mc2000429 | 21 | mcx001012 | 3 4 7 15 21 |
| mc2000445 | 10 16 21 | mcx001014 | 7 10 13 18 21 |
| mc2000450 | 5 21 | mcx001015 | 4 7 10 21 |
| mc2000452 | 1 7 21 | mcx001017 | 9 10 21 |
| mc2000454 | 1 21 | mcx001018 | 7 21 |
| mc2000455 | 4 7 21 | mcx001019 | 3 4 7 11 21 |
| mc2000457 | 4 7 21 23 24 | mcx001020 | 7 23 |
| mc2000458 | 6 7 8 9 12 15 17 18 21 | mcx001023 | 1 6 21 |
| mc2000462 | 1 3 4 6 7 21 | mcx001027 | 10 11 12 15 |
| mc2000464 | 3 4 7 15 16 18 21 22 23 24 | mcx001041 | 3 5 7 16 21 |
| mc2000477 | 4 7 21 | mcx001043 | 4 7 8 16 21 |
| mc2000480 | 7 8 10 12 20 | mcx001049 | 1 2 6 7 19 |
| mc2000482 | 1 4 | mcx001067 | 4 7 16 21 |
| mc2000483 | 1 21 | mcx001076 | 9 21 |
| mc2000484 | 1 7 9 21 | mcx001082 | 4 6 7 21 |
| mc2000488 | 17 | mcx001091 | 1 6 7 9 |
| mc2000494 | 3 8 17 18 21 | mcx001111 | 3 4 7 11 20 21 |
| mc2000495 | 1 6 7 10 21 | mcx001112 | 2 9 |
| mc2000497 | 7 | mcx001113 | 2 7 9 21 |
| mc2000499 | 7 9 | mcx001114 | 7 9 12 21 |
| mc2000500 | 1 7 15 21 | mcx001117 | 21 |
| mc2000501 | 1 | mcx001129 | 10 21 |
| mc2000502 | 1 7 | mcx001134 | 10 |
| mc2000509 | 7 | mcx001135 | 10 |
| mc2000511 | 1 21 | mcx001139 | 19 |
| mc2000512 | 1 21 | mcx001154 | 4 7 21 |
| mc2000513 | 8 | mcx001165 | 4 7 21 |
| mc2000522 | 1 | mcx001169 | 6 7 17 21 |
| mc2000523 | 7 | mcx001216 | 4 7 16 21 |
| mc2000524 | 4 7 16 21 | mcx001219 | 6 7 8 9 10 20 |
| mc2000525 | 1 21 | mcx001237 | 1 5 6 7 10 21 |
| mc2000527 | 6 7 11 21 | mcx001256 | 7 10 15 16 17 |
| mc2000530 | 6 | mcx001279 | 2 9 |
| mc2000532 | 7 9 | mcx001280 | 7 9 21 |
| mc2000535 | 7 8 9 10 12 22 23 24 | mcx001282 | 4 7 21 |
| mc2000539 | 1 7 21 | mcx001285 | 7 9 21 23 24 |
| mc2000544 | 1 3 4 7 21 | mcx001290 | 1 21 |
| mc2000546 | 1 3 6 7 | mcx001293 | 16 |
| mc2000550 | 7 12 21 | mcx001294 | 7 21 |
| mc2000557 | 1 6 7 17 21 | mcx001297 | 6 7 9 21 |
| mc2000558 | 1 7 | mcx001298 | 1 6 7 10 13 18 21 |
| mc2000561 | 21 | mcx001300 | 1 2 11 21 |
| mc2000563 | 2 4 7 9 21 | mcx001310 | 21 |
| mc2000565 | 7 9 | mcx001314 | 7 9 21 |
| mc2000566 | 1 2 4 7 9 11 12 21 | mcx001318 | 5 |
| mc2000568 | 7 9 21 | mcx001326 | 7 9 21 |
| mc2000573 | 1 21 | mcx001349 | 7 21 |
| mc2000575 | 1 7 10 | mcx001351 | 7 |
| mc2000577 | 1 6 21 | mcx001353 | 7 10 15 21 |
| mc2000578 | 1 3 7 21 | mcx001361 | 10 |
| mc2000579 | 7 8 10 12 19 20 21 | mcx001363 | 5 |
| mc2000580 | 1 21 | mcx001368 | 7 12 16 |
| mc2000581 | 1 3 4 6 7 8 10 21 | mcx001379 | 4 7 21 |
| mc2000582 | 8 10 | mcx001385 | 10 21 |
| mc2000585 | 5 | mcx001392 | 1 6 7 10 |
| mc2000587 | 7 9 12 21 22 23 24 | mcx001393 | 8 21 |
| mc2000589 | 6 7 9 | mcx001396 | 21 |
| mc2000590 | 5 7 21 | mcx001400 | 6 7 15 21 |
| mc2000591 | 1 7 9 10 11 21 | mcx001408 | 7 17 21 |
| mc2000595 | 15 20 21 | mcx001420 | 4 7 15 |
| mc2000599 | 4 7 21 | mcx001421 | 6 7 21 |
| mc2000612 | 4 7 13 16 21 | mcx001425 | 7 10 |
| mc2000616 | 1 8 10 | mcx001426 | 6 7 8 11 21 |
| mc2000626 | 6 7 11 21 | mcx001428 | 7 17 21 |
| mc2000627 | 9 21 | mcx001432 | 7 12 17 21 |
| mc2000628 | 6 7 10 21 | mcx001433 | 7 10 |
| mc2000630 | 6 7 8 11 13 21 | mcx001443 | 7 9 21 |
| mc2000633 | 7 8 10 15 | mcx001445 | 9 |
| mc2000634 | 8 13 15 21 | mcx001457 | 21 |
| mc2000639 | 7 9 10 | mcx001464 | 16 21 |
| mc2000641 | 7 9 17 21 | mcx001466 | 6 7 21 23 24 |
| mc2000646 | 1 7 21 | mcx001477 | 9 10 12 21 |
| mc2000658 | 7 8 | mcx001485 | 7 12 16 |
| mc2000664 | 2 7 9 21 | mcx001500 | 10 21 |
| mc2000666 | 4 7 21 | mcx001521 | 1 7 21 |
| mc2000668 | 7 | mcx001522 | 1 3 7 11 21 |
| mc2000692 | 10 13 | mcx001524 | 12 15 21 |
| mc2000693 | 10 13 | mcx001532 | 7 8 10 17 21 |
| mc2000696 | 10 13 | mcx001540 | 4 7 |
| mc2000700 | 10 13 | mcx001547 | 1 21 |
| mc2000705 | 10 13 | mcx001549 | 3 7 21 |
| mc2000707 | 10 13 | mcx001551 | 4 7 21 |
| mc2000710 | 7 8 10 21 | mcx001553 | 3 7 21 |
| mc2000713 | 7 21 | mcx001554 | 10 |
| mc2000720 | 7 12 13 16 21 | mcx001565 | 10 21 |
| mc2000729 | 4 7 16 20 21 | mcx001571 | 15 21 |
| mc2000733 | 1 6 7 10 | mcx001577 | 4 7 21 |
| mc2000734 | 6 7 8 11 13 21 | mcx001579 | 6 21 |
| mc2000737 | 4 7 10 15 16 19 20 21 | mcx001603 | 1 6 |
| mc2000741 | 4 7 10 21 | mcx001605 | 1 21 |
| mc2000748 | 17 21 | mcx001607 | 10 11 21 |
| mc2000749 | 21 | mcx001613 | 6 7 20 |
| mc2000752 | 1 3 6 7 10 15 | mcx001620 | 13 21 |
| mc2000760 | 7 9 10 | mcx001623 | 1 7 8 10 21 |
| mc2000775 | 1 7 21 | mcx001636 | 1 7 21 |
| mc2000776 | 7 8 15 | mcx001642 | 10 |
| mc2000783 | 4 7 12 16 21 | mcx001645 | 7 12 21 |
| mc2000811 | 1 6 7 8 10 21 | mcx001674 | 7 |
| mc2000812 | 3 4 7 21 | mcx001681 | 21 |
| mc2000832 | 21 | mcx001683 | 7 12 21 24 |
| mc2000845 | 7 21 | mcx001688 | 10 |
| mc2000858 | 4 7 8 | mcx001690 | 6 7 9 17 21 |
| mc2000859 | 7 12 21 | mcx001694 | 21 |
| mc2000866 | 6 9 10 12 13 | mcx001695 | 6 7 21 |
| mc2000871 | 7 8 9 10 21 | mcx001696 | 6 7 10 19 21 23 |
| mc2000888 | 3 21 | mcx001698 | 3 7 15 16 20 21 |
| mc2000891 | 1 6 7 21 | mcx001699 | 1 21 |
| mc2000895 | 7 9 10 21 24 | mcx001700 | 10 |
| mc2000903 | 4 7 10 16 18 21 | mcx001701 | 4 7 10 |
| mc2000906 | 1 7 9 10 21 | mcx001705 | 3 11 21 |
| mc2000910 | 1 7 21 | mcx001718 | 16 |
| mc2000913 | 7 12 21 | mcx001720 | 7 9 10 21 |
| mc2000918 | 1 6 7 8 10 21 | mcx001724 | 21 |
| mc2000921 | 1 21 | mcx001729 | 7 12 17 21 |
| mc2000949 | 7 | mcx001731 | 10 13 21 |
| mc2000953 | 7 9 10 11 21 | mcx001735 | 10 13 21 |
| mc2000958 | 7 10 21 | mcx001744 | 3 4 7 21 |
| mc2000959 | 7 10 12 | mcx001746 | 8 12 17 21 |
| mc2000968 | 7 10 21 | mcx001748 | 17 20 21 24 |
| mc2000971 | 6 7 8 11 21 | mcx001749 | 6 21 |
| mc2000972 | 4 7 12 16 21 | mcx001756 | 3 4 7 15 21 |
| mc2000974 | 4 7 9 12 21 | mcx001761 | 3 4 7 21 |
| mc2000975 | 6 21 | mcx001762 | 21 |
| mc2000976 | 9 10 21 | mcx001764 | 4 7 21 |
| mc2000977 | 6 | mcx001765 | 7 21 |
| mc2000979 | 3 4 7 12 13 15 | mcx001766 | 6 |
| mc2000980 | 7 21 | mcx001769 | 10 |
| mc2000981 | 4 7 9 10 | mcx001781 | 1 4 7 8 21 |
| mc2000986 | 7 | mcx001783 | 7 10 19 |
| mc2000996 | 7 8 10 21 | mcx001787 | 7 9 18 21 |
| mc2001014 | 15 21 | mcx001789 | 8 |
| mc2001016 | 1 7 21 | mcx001790 | 4 7 21 |
| mc2001029 | 7 9 21 | mcx001793 | 1 7 |
| mc2001047 | 1 6 7 21 | mcx001800 | 6 7 21 |
| mc2001048 | 1 6 7 | mcx001801 | 7 21 |
| mc2001061 | 21 | mcx001802 | 1 2 7 21 |
| mc2001062 | 1 7 9 21 | mcy000003 | 6 7 13 17 19 21 23 |
| mc2001066 | 1 21 |  |  |
