# Supplementary material for: Predicting Functions of Proteins in Mouse Based on Weighted Protein-Protein Interaction Network and Protein Hybrid Properties
Source: PLoS One. 2011 Jan 19;6(1):e14556. doi: 10.1371/journal.pone.0014556 (PMC3023709; doi:10.1371/journal.pone.0014556)
Supplement: Table S2 — Test set for network-based method. The Mfun ID and Functional number (see Table 1) of proteins are shown. (0.63 MB DOC) [file pone.0014556.s002.doc]

Table S2. Test set for network-based method. The Mfun ID and Functional number (see Table 1) of proteins are shown.

| MfunGD ID | Functional number(s) | MfunGD ID | Functional number(s) |
| --- | --- | --- | --- |
| mc10000040 | 8 10 | mc2001627 | 10 13 |
| mc1000020 | 1 21 | mc2001630 | 10 13 |
| mc10000461 | 1 6 7 10 15 16 | mc2001660 | 1 2 4 7 9 10 21 |
| mc10000530 | 9 10 | mc2001691 | 1 6 9 |
| mc10000676 | 7 9 | mc2001819 | 21 |
| mc10000680 | 7 12 16 17 21 | mc2001902 | 7 9 17 |
| mc10000696 | 1 6 7 21 | mc2001948 | 10 13 |
| mc10000778 | 7 10 21 | mc2001953 | 10 13 |
| mc10000781 | 1 | mc2001993 | 7 13 17 21 23 |
| mc10000901 | 6 7 | mc2002053 | 7 12 20 21 |
| mc10000963 | 11 21 24 | mc2002155 | 1 2 7 21 |
| mc10000967 | 21 | mc2002234 | 7 21 23 |
| mc10000994 | 7 | mc2002336 | 4 7 21 |
| mc10001030 | 1 2 7 21 | mc2002342 | 4 7 16 21 |
| mc10001040 | 1 7 21 | mc2002352 | 10 21 |
| mc10001088 | 4 7 21 | mc2002391 | 1 21 |
| mc10001101 | 7 13 | mc2002392 | 10 12 |
| mc10001130 | 3 4 6 7 21 | mc2002413 | 7 10 15 16 18 19 20 21 |
| mc10001146 | 1 5 6 7 | mc2002457 | 7 9 12 21 |
| mc10001167 | 7 10 11 13 21 | mc2002533 | 3 7 12 21 |
| mc10001171 | 6 7 21 24 | mc2002540 | 9 21 |
| mc10001191 | 7 10 | mc2002628 | 7 9 21 |
| mc10001201 | 3 4 7 20 21 | mc2002673 | 1 2 7 |
| mc10001211 | 4 7 8 20 21 | mc2002681 | 3 6 7 10 11 12 |
| mc10001212 | 1 4 6 7 10 21 | mc2002682 | 7 |
| mc1000122 | 1 4 6 7 8 10 15 16 18 20 21 | mc2002769 | 3 7 17 21 |
| mc10001231 | 1 2 3 7 9 11 21 | mc2002796 | 9 |
| mc10001261 | 7 | mc2002810 | 6 21 |
| mc10001390 | 1 6 7 10 | mc2002864 | 15 |
| mc10001572 | 7 9 12 19 21 22 24 | mc2002972 | 1 6 7 10 21 |
| mc10001595 | 7 15 16 21 | mc2002997 | 6 7 8 10 12 21 22 |
| mc10001684 | 1 11 13 15 17 21 | mc2003033 | 7 13 21 23 |
| mc10001685 | 1 11 13 15 17 21 | mc2003047 | 7 9 12 21 |
| mc10001853 | 1 3 6 7 21 | mc2003124 | 3 7 10 17 21 |
| mc10001890 | 1 9 21 24 | mc2003140 | 1 7 9 |
| mc10001891 | 1 9 21 24 | mc2003253 | 7 10 21 |
| mc10001892 | 1 9 21 24 | mc2003313 | 4 7 16 |
| mc10001896 | 1 9 21 24 | mc2003361 | 10 |
| mc10001900 | 1 9 21 24 | mc2003371 | 6 7 11 |
| mc10001920 | 1 2 21 | mc2003406 | 10 13 |
| mc10001929 | 2 7 9 17 19 21 | mc2003454 | 10 12 |
| mc10001944 | 7 9 10 17 21 | mc3000003 | 4 7 8 10 20 21 24 |
| mc1000205 | 11 21 | mc3000036 | 7 10 22 |
| mc1000284 | 4 7 9 10 21 | mc3000069 | 7 9 |
| mc1000373 | 7 9 10 12 13 | mc3000112 | 1 7 21 |
| mc1000379 | 1 | mc3000156 | 7 8 10 21 |
| mc1000669 | 1 2 7 9 | mc3000172 | 4 7 21 |
| mc1000671 | 1 2 7 9 | mc3000222 | 5 |
| mc1000699 | 1 10 16 21 | mc3000260 | 6 7 8 10 21 |
| mc1000789 | 20 21 24 | mc3000287 | 21 |
| mc1000790 | 20 21 24 | mc3000360 | 9 10 12 21 |
| mc1000791 | 20 21 24 | mc3000375 | 7 21 |
| mc1000793 | 20 21 24 | mc3000561 | 9 10 12 21 |
| mc1000797 | 1 7 21 | mc3000613 | 6 7 17 |
| mc1000804 | 20 21 24 | mc3000725 | 7 9 12 21 |
| mc1000818 | 2 7 9 17 19 21 | mc3000806 | 6 7 9 21 |
| mc1000918 | 7 9 | mc3000899 | 7 9 10 12 21 22 24 |
| mc1000919 | 1 6 21 | mc3001075 | 7 9 12 21 |
| mc1000955 | 6 7 9 17 21 | mc3001098 | 1 3 8 |
| mc1000994 | 1 6 7 10 12 13 16 | mc3001127 | 9 17 21 |
| mc1001003 | 3 4 7 12 15 16 19 20 21 | mc3001182 | 7 19 |
| mc1001028 | 4 7 9 21 | mc3001249 | 7 |
| mc1001147 | 1 16 | mc3001254 | 1 7 10 21 |
| mc1001203 | 7 8 9 10 21 | mc3001308 | 7 21 |
| mc1001247 | 4 7 15 16 21 | mc3001312 | 7 21 |
| mc1001287 | 1 6 7 21 | mc3001355 | 1 6 10 11 |
| mc1001422 | 7 12 17 21 | mc3001373 | 1 21 |
| mc1001592 | 13 21 | mc3001376 | 1 21 |
| mc1001633 | 1 6 7 10 | mc3001378 | 1 21 |
| mc1001668 | 9 | mc3001404 | 1 6 7 11 21 |
| mc1001694 | 4 6 7 11 15 16 18 | mc3001422 | 2 7 9 21 |
| mc1001708 | 1 2 7 12 21 23 | mc3001455 | 7 9 16 21 24 |
| mc1001733 | 6 7 9 21 | mc3001480 | 3 7 10 17 |
| mc1001740 | 7 17 21 | mc3001497 | 3 7 10 |
| mc1001788 | 1 6 7 10 | mc3001510 | 1 2 7 13 |
| mc1001793 | 4 7 21 | mc3001511 | 1 2 13 |
| mc1001910 | 6 7 12 17 21 | mc3001523 | 9 12 21 |
| mc1001968 | 4 7 15 21 | mc3001527 | 9 12 21 24 |
| mc1002084 | 7 21 | mc3001537 | 9 21 |
| mc1002104 | 4 7 16 21 | mc3001552 | 11 24 |
| mc1002175 | 1 7 | mc3001554 | 11 21 |
| mc1002184 | 4 7 10 21 | mc3001556 | 11 21 |
| mc1002195 | 1 4 7 15 16 20 21 | mc3001557 | 11 21 24 |
| mc1002267 | 4 6 7 9 11 21 | mc3001559 | 11 21 24 |
| mc1002280 | 16 | mc3001562 | 7 8 10 21 |
| mc1002290 | 7 12 21 23 | mc3001593 | 9 21 |
| mc1002292 | 1 7 9 12 13 | mc3001627 | 7 21 |
| mc1002294 | 7 9 10 21 24 | mc3001739 | 1 6 7 8 12 13 21 24 |
| mc1002357 | 7 8 10 22 24 | mc3001749 | 6 |
| mc1002391 | 1 6 7 8 10 21 | mc3001757 | 6 7 10 23 |
| mc1002398 | 1 7 | mc3001920 | 1 3 6 7 9 11 21 23 |
| mc1002451 | 3 7 17 21 | mc3001943 | 1 7 21 24 |
| mc1002456 | 7 15 16 21 | mc3001950 | 1 7 21 |
| mc1002490 | 6 7 16 19 21 | mc3001979 | 6 7 10 21 |
| mc1002561 | 3 4 7 15 16 | mc3001990 | 7 13 21 |
| mc1002642 | 1 6 7 8 10 | mc3001993 | 7 13 |
| mc1002696 | 10 16 21 | mc3002029 | 9 10 21 |
| mc1002722 | 7 11 | mc3002031 | 9 10 15 21 |
| mc11000093 | 1 2 | mc3002140 | 6 7 11 |
| mc11000108 | 9 21 | mc3002147 | 6 7 |
| mc11000112 | 6 21 | mc3002271 | 1 2 7 |
| mc11000156 | 1 21 | mc4000006 | 6 |
| mc11000249 | 4 7 21 | mc4000054 | 6 7 9 10 21 |
| mc11000489 | 7 | mc4000132 | 1 4 7 8 13 21 |
| mc11000565 | 7 9 10 12 21 23 24 | mc4000172 | 6 7 21 |
| mc11000567 | 7 9 10 12 21 23 24 | mc4000180 | 1 6 7 9 |
| mc11000652 | 4 7 10 21 | mc4000185 | 2 9 |
| mc11000752 | 10 13 | mc4000299 | 7 21 |
| mc11000788 | 7 9 10 21 | mc4000302 | 9 10 12 21 23 24 |
| mc11000804 | 7 9 15 21 | mc4000448 | 7 13 17 19 21 |
| mc11000818 | 6 7 11 12 | mc4000454 | 1 6 7 10 21 |
| mc11000839 | 9 21 | mc4000465 | 10 13 21 |
| mc11000841 | 9 21 | mc4000466 | 10 13 21 |
| mc11000850 | 1 2 7 18 21 24 | mc4000482 | 1 3 6 7 21 |
| mc11000873 | 9 21 | mc4000543 | 6 7 10 16 20 21 |
| mc11000880 | 1 3 4 7 9 21 | mc4000735 | 7 9 21 |
| mc11000881 | 9 10 12 21 | mc4000777 | 7 9 13 21 |
| mc11000939 | 10 13 | mc4000783 | 1 2 7 9 21 |
| mc11000942 | 10 13 | mc4000837 | 7 8 10 16 21 22 |
| mc11000951 | 10 13 | mc4000853 | 4 7 8 10 21 |
| mc11000981 | 3 10 15 16 18 21 23 | mc4000906 | 3 4 7 15 20 21 |
| mc11001041 | 9 21 | mc4000969 | 7 9 12 22 24 |
| mc11001047 | 1 7 9 11 21 | mc4001017 | 12 15 21 |
| mc11001090 | 6 21 | mc4001020 | 12 15 21 |
| mc11001177 | 2 3 7 9 17 21 | mc4001030 | 7 10 11 12 15 21 |
| mc11001206 | 2 6 7 9 12 15 16 17 21 | mc4001031 | 7 10 11 12 15 21 |
| mc11001221 | 7 9 21 22 23 24 | mc4001032 | 7 10 11 12 15 21 |
| mc11001252 | 5 7 | mc4001110 | 7 9 21 |
| mc11001298 | 1 7 9 13 21 24 | mc4001112 | 1 7 9 13 21 24 |
| mc11001418 | 10 13 | mc4001213 | 1 10 |
| mc11001423 | 10 13 21 | mc4001336 | 7 9 21 24 |
| mc11001431 | 10 13 | mc4001353 | 7 |
| mc11001557 | 21 | mc4001411 | 1 2 7 9 21 |
| mc11001572 | 7 9 | mc4001443 | 8 10 21 |
| mc11001673 | 6 7 9 17 21 | mc4001452 | 9 11 |
| mc11001782 | 7 11 | mc4001499 | 1 6 8 10 21 |
| mc11001783 | 10 13 | mc4001582 | 9 21 |
| mc11001794 | 4 5 7 21 | mc4001609 | 7 8 9 10 12 17 21 |
| mc11001860 | 1 7 21 | mc4001626 | 10 15 16 |
| mc11001861 | 1 7 21 | mc4001627 | 1 2 21 24 |
| mc11001884 | 1 6 21 | mc4001777 | 7 |
| mc11001886 | 3 6 7 | mc4001796 | 6 10 21 |
| mc11001894 | 7 9 12 17 21 | mc4001805 | 6 7 9 |
| mc11001900 | 7 8 17 21 22 | mc4001823 | 10 |
| mc11001902 | 1 2 6 7 8 21 | mc4001893 | 1 3 6 7 10 |
| mc11001936 | 7 | mc4001927 | 5 6 8 |
| mc11001942 | 2 7 9 21 | mc4001948 | 1 3 6 7 |
| mc11001952 | 4 7 15 16 21 | mc4001954 | 3 7 10 12 16 17 21 |
| mc11002009 | 1 7 10 21 | mc4001977 | 9 12 15 21 |
| mc11002018 | 7 | mc4002000 | 1 21 |
| mc11002020 | 6 7 9 10 12 13 | mc4002018 | 10 21 |
| mc11002054 | 4 7 8 10 13 16 18 21 24 | mc4002025 | 1 6 7 8 10 21 24 |
| mc11002061 | 4 7 10 21 | mc4002040 | 3 7 9 10 12 15 17 |
| mc11002119 | 21 | mc4002059 | 6 7 21 |
| mc11002122 | 7 17 21 | mc4002135 | 9 |
| mc11002123 | 7 17 21 | mc4002147 | 9 |
| mc11002126 | 7 17 18 19 20 21 22 23 24 | mc4002274 | 1 6 7 21 |
| mc11002127 | 7 17 18 19 20 21 22 23 24 | mc4002287 | 6 7 |
| mc11002128 | 7 17 18 19 20 21 22 23 24 | mc4002350 | 1 2 7 21 |
| mc11002132 | 7 17 18 19 20 21 22 23 24 | mc4002369 | 7 9 21 |
| mc11002133 | 7 17 18 19 20 21 22 23 24 | mc4002408 | 8 9 21 |
| mc11002142 | 7 12 21 | mc4002487 | 7 10 21 |
| mc11002152 | 7 10 | mc4002506 | 3 4 21 |
| mc11002163 | 4 7 10 21 | mc4002544 | 10 13 18 21 |
| mc11002178 | 7 17 21 | mc4002554 | 7 10 11 12 15 21 |
| mc11002202 | 7 10 12 17 22 | mc4002558 | 4 7 21 |
| mc11002247 | 7 10 16 19 21 | mc5000020 | 1 7 9 21 |
| mc11002267 | 1 6 16 21 | mc5000022 | 1 7 9 21 |
| mc11002292 | 7 10 15 16 17 21 | mc5000029 | 7 21 |
| mc11002295 | 7 9 10 17 21 | mc5000251 | 3 4 6 21 |
| mc11002299 | 10 11 13 21 | mc5000302 | 4 7 10 |
| mc11002309 | 2 7 9 17 19 21 | mc5000426 | 7 8 10 21 22 24 |
| mc11002320 | 3 6 7 11 16 21 | mc5000428 | 7 9 13 20 21 |
| mc11002347 | 3 4 21 | mc5000469 | 1 6 7 10 21 |
| mc11002385 | 1 6 7 8 9 10 12 13 15 21 | mc5000512 | 1 6 7 8 10 |
| mc11002405 | 7 10 | mc5000519 | 22 |
| mc11002418 | 1 7 9 21 | mc5000522 | 1 6 7 10 |
| mc11002419 | 1 7 9 | mc5000604 | 4 8 16 21 |
| mc11002425 | 1 6 7 10 | mc5000638 | 7 10 12 13 15 16 20 21 |
| mc11002537 | 6 | mc5000640 | 7 9 |
| mc11002558 | 3 7 17 21 | mc5000774 | 10 13 |
| mc11002610 | 1 3 7 | mc5000846 | 1 |
| mc11002634 | 8 | mc5000849 | 9 10 12 21 23 24 |
| mc11002725 | 1 21 | mc5000850 | 9 10 12 21 |
| mc11002732 | 1 7 21 24 | mc5000854 | 9 10 12 21 22 23 24 |
| mc11002744 | 1 6 7 10 | mc5001040 | 1 11 21 |
| mc11002763 | 1 7 21 | mc5001041 | 1 11 21 24 |
| mc11002794 | 10 13 | mc5001052 | 1 11 21 |
| mc12000004 | 7 9 10 21 | mc5001053 | 1 13 21 |
| mc12000083 | 5 7 | mc5001129 | 12 13 15 |
| mc12000100 | 7 21 | mc5001305 | 6 7 8 10 11 21 24 |
| mc12000117 | 7 | mc5001422 | 1 10 12 13 |
| mc12000377 | 5 | mc5001490 | 7 9 16 |
| mc12000575 | 4 7 15 16 21 | mc5001525 | 6 7 8 10 21 |
| mc12000602 | 7 9 17 21 | mc5001564 | 9 |
| mc12000693 | 7 8 9 10 21 | mc5001605 | 1 7 9 12 17 21 |
| mc12000761 | 6 7 10 | mc5001606 | 4 7 9 21 |
| mc12000763 | 4 7 13 16 21 | mc5001646 | 7 8 16 21 |
| mc12000765 | 4 7 16 18 21 | mc5001695 | 4 7 21 |
| mc12000790 | 7 | mc5001707 | 6 7 10 21 |
| mc12000805 | 1 6 7 8 10 21 | mc5001715 | 1 7 12 13 |
| mc12000823 | 6 7 11 18 21 | mc5001738 | 6 7 12 21 22 24 |
| mc12000833 | 7 11 21 | mc5001740 | 1 2 7 9 11 21 |
| mc12000913 | 1 6 7 | mc5001753 | 2 7 9 17 19 21 |
| mc12000953 | 1 21 | mc5001755 | 6 7 21 |
| mc12000954 | 1 21 | mc5001914 | 21 |
| mc12001014 | 4 6 7 9 10 21 | mc5001918 | 1 2 6 7 10 19 |
| mc12001060 | 9 12 16 | mc5001927 | 6 21 |
| mc12001154 | 7 9 | mc5001996 | 7 11 12 17 21 23 |
| mc12001225 | 8 21 | mc5002002 | 7 9 21 22 |
| mc12001231 | 8 21 | mc5002062 | 6 7 9 17 21 |
| mc12001239 | 8 | mc5002077 | 7 8 10 16 18 21 |
| mc12001249 | 8 | mc5002078 | 3 4 7 17 21 23 |
| mc12001342 | 1 | mc5002099 | 1 2 7 9 11 21 |
| mc12001415 | 1 2 7 21 24 | mc5002137 | 1 3 6 7 8 10 13 15 21 |
| mc12001421 | 7 12 13 16 17 21 23 | mc5002161 | 4 7 18 21 |
| mc12001449 | 1 6 7 8 10 15 21 | mc5002230 | 7 17 21 |
| mc12001472 | 7 | mc5002254 | 7 10 17 21 |
| mc13000011 | 7 | mc5002257 | 6 7 8 9 10 17 21 |
| mc13000020 | 1 7 13 21 | mc5002290 | 7 9 15 17 21 |
| mc13000022 | 11 | mc5002306 | 1 9 11 21 24 |
| mc13000024 | 11 | mc5002309 | 1 9 11 21 24 |
| mc13000026 | 1 7 13 21 24 | mc5002312 | 1 2 7 9 21 |
| mc13000028 | 1 7 21 | mc5002366 | 1 7 17 21 |
| mc13000029 | 1 7 21 | mc6000010 | 7 10 21 |
| mc13000097 | 7 13 17 19 21 23 | mc6000030 | 1 21 |
| mc13000155 | 7 10 12 16 | mc6000036 | 1 7 8 21 |
| mc13000204 | 11 21 | mc6000116 | 10 21 |
| mc13000206 | 11 21 | mc6000149 | 7 10 15 16 21 |
| mc13000225 | 3 7 17 21 | mc6000171 | 7 9 12 21 22 23 24 |
| mc13000232 | 7 21 | mc6000286 | 1 |
| mc13000317 | 7 21 | mc6000328 | 10 16 21 |
| mc13000318 | 3 7 17 21 | mc6000352 | 1 7 |
| mc13000322 | 3 7 17 21 | mc6000354 | 1 |
| mc13000330 | 3 7 17 21 | mc6000383 | 10 21 |
| mc13000336 | 3 7 17 21 | mc6000402 | 2 9 |
| mc13000339 | 3 7 17 21 | mc6000472 | 6 13 21 |
| mc13000344 | 3 7 21 | mc6000492 | 6 7 13 |
| mc13000354 | 7 21 | mc6000504 | 21 |
| mc13000399 | 10 | mc6000575 | 4 6 7 16 17 18 21 |
| mc13000408 | 7 10 21 | mc6000647 | 1 7 21 |
| mc13000409 | 10 21 | mc6000655 | 2 7 9 21 |
| mc13000500 | 6 8 | mc6000886 | 10 |
| mc13000503 | 6 8 | mc6001058 | 1 7 |
| mc13000508 | 6 8 | mc6001112 | 7 21 |
| mc13000509 | 8 | mc6001113 | 7 13 21 |
| mc13000511 | 6 8 | mc6001153 | 4 7 16 21 |
| mc13000515 | 8 | mc6001246 | 7 15 21 |
| mc13000522 | 8 | mc6001261 | 1 7 9 21 |
| mc13000528 | 7 17 21 | mc6001298 | 6 7 11 |
| mc13000530 | 7 17 21 | mc6001338 | 10 |
| mc13000548 | 1 2 21 | mc6001373 | 7 10 15 16 21 |
| mc13000627 | 4 7 21 | mc6001561 | 1 7 9 12 21 |
| mc13000703 | 1 7 | mc6001612 | 7 8 9 19 21 23 |
| mc13000828 | 4 7 16 21 | mc6001673 | 7 9 21 |
| mc13000886 | 1 6 7 10 | mc6001732 | 4 7 21 |
| mc13000907 | 4 7 16 21 | mc6001735 | 4 7 21 |
| mc13001007 | 6 21 | mc6001758 | 7 10 15 16 21 |
| mc13001008 | 6 21 | mc6001782 | 7 9 12 21 |
| mc13001017 | 6 21 | mc6001793 | 6 7 9 17 21 |
| mc13001026 | 1 2 6 7 | mc6001796 | 9 12 21 24 |
| mc13001068 | 9 | mc6001797 | 9 12 21 24 |
| mc13001076 | 3 7 10 21 | mc6001806 | 8 9 10 21 |
| mc13001200 | 7 9 13 21 | mc6001837 | 10 |
| mc13001298 | 20 21 24 | mc6001839 | 7 |
| mc13001357 | 4 7 21 | mc6001872 | 1 2 7 21 |
| mc13001528 | 15 | mc6001878 | 1 7 10 21 |
| mc13001564 | 7 8 10 13 21 | mc6001902 | 7 12 17 21 |
| mc13001676 | 1 7 10 13 | mc6001923 | 9 12 21 |
| mc13001802 | 2 4 7 16 19 20 21 | mc6001939 | 3 21 |
| mc13001843 | 10 13 | mc6001960 | 4 7 21 |
| mc14000103 | 4 7 10 20 21 | mc6001982 | 7 10 13 21 |
| mc14000126 | 6 7 | mc6001987 | 7 10 13 21 |
| mc14000131 | 6 7 | mc6001992 | 7 10 13 15 18 21 |
| mc14000140 | 3 7 10 21 | mc6002004 | 7 10 17 21 |
| mc14000168 | 6 7 10 23 | mc6002008 | 7 10 |
| mc14000257 | 7 8 9 10 21 | mc6002010 | 7 10 |
| mc14000411 | 6 7 8 10 11 13 21 | mc6002014 | 7 10 11 12 21 |
| mc14000549 | 10 13 | mc6002020 | 7 10 11 12 21 |
| mc14000610 | 10 13 | mc6002076 | 7 9 10 16 21 |
| mc14000616 | 10 13 | mc6002127 | 7 8 21 |
| mc14000618 | 10 13 | mc6002128 | 1 10 12 13 |
| mc14000654 | 1 7 13 20 21 | mc6002147 | 4 7 |
| mc14000655 | 1 3 | mc6002184 | 9 21 |
| mc14000656 | 1 3 7 21 | mc6002187 | 9 21 |
| mc14000670 | 1 7 13 20 21 | mc6002193 | 1 |
| mc14000694 | 10 13 | mc6002242 | 3 7 10 19 |
| mc14000696 | 10 13 | mc6002260 | 7 9 10 21 |
| mc14000698 | 10 13 | mc6002295 | 5 7 21 |
| mc14000699 | 10 13 | mc6002331 | 4 7 21 |
| mc14000730 | 1 6 9 21 | mc6002348 | 7 8 9 21 |
| mc14000758 | 2 6 7 9 13 17 19 21 23 | mc6002350 | 1 2 7 21 |
| mc14000818 | 6 | mc6002355 | 10 21 |
| mc14000819 | 6 | mc6002356 | 10 21 |
| mc14000820 | 6 21 | mc6002357 | 7 10 21 |
| mc14000822 | 6 21 | mc6002359 | 7 10 21 |
| mc14000826 | 6 15 21 | mc7000013 | 10 |
| mc14000827 | 6 15 21 | mc7000037 | 13 17 19 21 |
| mc14000828 | 6 | mc7000133 | 9 |
| mc14000830 | 6 15 21 | mc7000244 | 4 7 9 21 |
| mc14000835 | 7 9 12 21 | mc7000275 | 7 10 21 |
| mc14000850 | 7 9 12 21 | mc7000277 | 7 8 10 |
| mc14000892 | 7 | mc7000310 | 21 |
| mc14000896 | 1 7 9 | mc7000311 | 7 13 21 |
| mc14000964 | 1 6 7 10 | mc7000347 | 1 2 7 21 23 |
| mc14001039 | 7 16 18 20 21 | mc7000460 | 21 |
| mc14001094 | 1 6 7 | mc7000480 | 7 9 11 21 |
| mc14001101 | 6 7 15 16 20 21 | mc7000482 | 7 9 11 21 |
| mc14001112 | 6 7 9 21 | mc7000489 | 1 2 7 9 21 |
| mc14001184 | 2 9 13 21 | mc7000495 | 7 9 11 21 23 |
| mc14001239 | 7 | mc7000499 | 1 7 9 21 |
| mc14001515 | 21 | mc7000513 | 7 |
| mc14001671 | 7 | mc7000595 | 4 6 7 21 |
| mc15000081 | 9 12 21 23 24 | mc7000622 | 4 7 21 |
| mc15000129 | 2 5 7 9 | mc7000658 | 1 7 9 |
| mc15000332 | 1 6 7 10 15 21 | mc7000732 | 7 9 21 |
| mc15000359 | 4 7 21 | mc7000883 | 6 |
| mc15000361 | 7 8 10 15 21 22 | mc7000884 | 6 21 |
| mc15000379 | 1 | mc7000885 | 6 21 |
| mc15000550 | 1 | mc7000887 | 6 21 |
| mc15000577 | 6 7 21 | mc7000888 | 6 21 |
| mc15000753 | 5 | mc7000894 | 6 21 |
| mc15000766 | 1 6 8 10 | mc7000896 | 6 |
| mc15000807 | 21 | mc7000897 | 6 |
| mc15000891 | 1 2 7 21 | mc7000899 | 6 7 10 15 21 |
| mc15000933 | 8 9 10 12 21 24 | mc7000900 | 6 21 |
| mc15000941 | 10 13 15 18 21 | mc7000901 | 6 21 |
| mc15000957 | 7 10 21 | mc7000956 | 9 |
| mc15000959 | 7 8 9 21 | mc7000998 | 1 6 21 |
| mc15000994 | 3 4 7 16 21 | mc7001015 | 9 10 21 |
| mc15001004 | 3 4 7 21 | mc7001024 | 7 9 11 12 21 22 24 |
| mc15001097 | 7 9 21 | mc7001030 | 9 12 21 |
| mc15001102 | 1 2 7 9 21 | mc7001036 | 1 23 24 |
| mc15001113 | 1 2 7 9 21 | mc7001040 | 7 9 13 21 |
| mc15001146 | 7 12 21 | mc7001041 | 7 9 13 21 |
| mc15001239 | 1 6 7 10 | mc7001045 | 1 2 21 |
| mc15001257 | 1 2 9 21 | mc7001046 | 1 2 21 |
| mc15001410 | 1 7 9 12 17 20 21 | mc7001119 | 9 12 21 |
| mc15001413 | 1 2 21 23 | mc7001143 | 7 10 17 |
| mc15001436 | 6 9 10 12 13 23 | mc7001152 | 9 10 12 21 23 24 |
| mc15001438 | 7 10 12 17 | mc7001161 | 9 10 12 21 23 24 |
| mc15001441 | 7 9 10 17 21 | mc7001164 | 9 10 12 21 23 24 |
| mc15001442 | 7 10 15 16 21 | mc7001265 | 1 7 9 13 21 24 |
| mc15001445 | 1 6 10 11 21 | mc7001334 | 4 7 10 12 16 20 21 |
| mc15001457 | 7 12 17 21 | mc7001459 | 6 9 21 |
| mc15001476 | 9 21 | mc7001490 | 6 7 21 |
| mc15001495 | 4 7 8 21 | mc7001605 | 7 9 10 |
| mc15001504 | 4 7 21 | mc7001765 | 2 9 13 21 23 |
| mc15001511 | 1 7 21 | mc7001817 | 4 7 10 17 |
| mc15001529 | 7 17 | mc7001836 | 10 13 |
| mc15001546 | 7 17 19 21 | mc7001859 | 10 13 |
| mc15001547 | 7 17 | mc7001917 | 9 18 22 |
| mc15001553 | 7 17 19 23 | mc7001918 | 9 18 22 |
| mc15001557 | 7 17 | mc7001919 | 10 13 21 |
| mc15001586 | 4 7 21 | mc7001921 | 10 13 21 |
| mc15001606 | 4 7 15 16 18 21 | mc7001931 | 10 13 |
| mc15001618 | 4 7 9 21 | mc7001934 | 10 13 |
| mc15001620 | 7 9 17 21 | mc7002020 | 7 9 21 |
| mc15001659 | 6 7 9 17 21 | mc7002052 | 10 13 |
| mc16000100 | 1 21 | mc7002053 | 10 13 |
| mc16000202 | 2 7 9 13 17 19 21 23 | mc7002064 | 10 13 21 |
| mc16000216 | 6 7 | mc7002068 | 10 13 |
| mc16000245 | 3 6 7 10 12 13 21 | mc7002072 | 7 9 21 |
| mc16000264 | 3 7 8 10 15 21 | mc7002118 | 10 13 |
| mc16000283 | 1 6 7 10 15 16 | mc7002199 | 4 7 21 |
| mc16000315 | 3 7 | mc7002220 | 7 9 10 21 |
| mc16000338 | 10 13 | mc7002310 | 1 21 |
| mc16000339 | 10 13 | mc7002442 | 7 9 13 21 |
| mc16000362 | 7 10 16 | mc7002462 | 3 7 9 17 21 |
| mc16000433 | 3 4 5 7 | mc7002469 | 1 6 7 10 21 |
| mc16000496 | 7 10 15 21 | mc7002470 | 1 2 21 23 |
| mc16000562 | 1 6 7 | mc7002495 | 2 7 9 17 19 21 |
| mc16000576 | 1 12 | mc7002496 | 3 7 21 |
| mc16000798 | 16 19 20 | mc7002535 | 7 9 21 |
| mc16000998 | 1 6 7 8 10 21 | mc7002701 | 4 7 21 |
| mc16001056 | 12 13 15 16 20 21 | mc7002727 | 7 |
| mc16001168 | 7 9 10 12 | mc7002813 | 12 16 |
| mc16001316 | 1 | mc7002815 | 12 15 |
| mc16001346 | 9 12 21 | mc7002824 | 3 7 9 10 15 21 |
| mc16001375 | 7 12 13 21 | mc7002853 | 6 7 9 17 21 |
| mc16001391 | 7 10 21 | mc7002887 | 7 13 16 17 19 21 |
| mc17000070 | 1 3 5 6 7 16 21 | mc7002916 | 3 7 21 |
| mc17000157 | 7 9 21 | mc7002920 | 7 10 |
| mc17000244 | 10 | mc7002999 | 7 10 12 13 |
| mc17000267 | 10 | mc7003005 | 7 9 13 |
| mc17000333 | 7 17 21 | mc7003010 | 6 |
| mc17000399 | 5 7 21 | mc7003024 | 1 6 7 10 15 16 18 19 20 21 |
| mc17000436 | 6 7 13 21 | mc8000016 | 7 9 21 |
| mc17000497 | 1 3 6 8 10 | mc8000187 | 3 7 15 |
| mc17000557 | 1 4 7 10 13 17 21 | mc8000291 | 11 12 13 21 |
| mc17000560 | 4 7 21 | mc8000331 | 9 10 21 |
| mc17000572 | 1 2 3 4 6 7 10 11 16 18 19 20 21 | mc8000332 | 9 21 |
| mc17000573 | 1 3 6 7 10 | mc8000338 | 6 9 21 |
| mc17000579 | 7 9 | mc8000420 | 5 7 10 |
| mc17000581 | 4 7 21 | mc8000424 | 9 10 12 21 |
| mc17000597 | 3 6 7 15 21 | mc8000499 | 3 6 7 8 10 15 21 |
| mc17000658 | 1 2 6 7 9 | mc8000509 | 10 |
| mc17000660 | 1 2 7 9 | mc8000583 | 4 7 21 |
| mc17000669 | 1 7 9 13 21 | mc8000586 | 1 6 7 10 21 |
| mc17000670 | 1 2 7 9 21 | mc8000706 | 21 |
| mc17000746 | 4 7 21 | mc8000870 | 1 |
| mc17000778 | 7 11 22 24 | mc8000872 | 1 9 21 |
| mc17000785 | 9 21 | mc8000894 | 7 9 12 13 21 24 |
| mc17000810 | 7 11 13 21 | mc8000934 | 7 12 21 |
| mc17000814 | 10 11 13 21 | mc8000954 | 10 12 21 22 23 24 |
| mc17000815 | 10 11 13 21 | mc8000963 | 7 9 |
| mc17000818 | 7 11 13 | mc8000980 | 4 7 10 21 |
| mc17000837 | 7 17 21 | mc8001014 | 1 2 7 9 21 |
| mc17000869 | 11 | mc8001018 | 7 19 21 |
| mc17000871 | 11 | mc8001065 | 9 |
| mc17000873 | 11 | mc8001115 | 4 7 21 |
| mc17000879 | 11 | mc8001128 | 4 7 8 10 21 |
| mc17000883 | 11 | mc8001197 | 6 7 11 21 |
| mc17000900 | 10 13 | mc8001203 | 3 4 7 21 |
| mc17001011 | 4 8 23 24 | mc8001209 | 3 7 21 |
| mc17001047 | 6 7 11 21 | mc8001210 | 1 6 7 10 16 21 |
| mc17001204 | 7 9 10 21 | mc8001239 | 3 4 7 21 |
| mc17001206 | 3 6 7 15 | mc8001254 | 7 11 15 |
| mc17001307 | 7 17 21 | mc8001261 | 6 7 9 21 |
| mc17001408 | 7 21 | mc8001286 | 3 6 7 15 16 21 |
| mc17001433 | 1 6 8 10 21 | mc8001398 | 9 13 21 |
| mc17001552 | 7 21 | mc8001402 | 1 11 12 21 |
| mc17001599 | 10 21 | mc8001406 | 1 11 21 |
| mc17001625 | 7 | mc8001407 | 1 11 21 |
| mc17001739 | 7 9 10 15 17 21 | mc8001408 | 1 11 21 |
| mc17001755 | 7 8 10 | mc8001418 | 7 10 13 |
| mc17001831 | 7 16 21 | mc8001427 | 7 9 12 21 24 |
| mc18000100 | 6 7 8 10 17 | mc8001475 | 1 3 6 7 10 21 |
| mc18000143 | 1 9 | mc8001484 | 15 16 |
| mc18000211 | 7 12 17 21 | mc8001533 | 7 12 17 18 19 21 |
| mc18000266 | 7 10 12 13 18 21 22 23 | mc8001579 | 1 21 |
| mc18000279 | 1 6 7 21 | mc8001582 | 1 21 |
| mc18000409 | 7 12 17 21 | mc8001586 | 11 21 |
| mc18000416 | 7 9 21 | mc8001588 | 1 11 21 |
| mc18000425 | 6 7 | mc8001642 | 7 9 21 |
| mc18000458 | 5 7 21 | mc8001643 | 6 7 21 |
| mc18000504 | 7 12 21 | mc8001668 | 1 7 9 21 |
| mc18000505 | 7 12 21 | mc8001753 | 3 4 7 21 |
| mc18000506 | 7 12 | mc8002016 | 7 9 10 21 |
| mc18000507 | 7 12 | mc8002018 | 7 13 17 21 23 |
| mc18000508 | 7 12 21 | mc8002046 | 6 7 |
| mc18000512 | 7 12 21 | mc9000016 | 7 9 10 12 21 22 24 |
| mc18000514 | 7 12 21 | mc9000042 | 6 7 21 |
| mc18000515 | 7 12 21 | mc9000046 | 6 7 21 |
| mc18000516 | 7 12 | mc9000096 | 1 6 8 10 21 |
| mc18000531 | 7 | mc9000253 | 6 7 9 17 21 |
| mc18000654 | 4 7 21 | mc9000286 | 7 13 |
| mc18000680 | 4 7 20 21 | mc9000442 | 7 9 10 13 21 |
| mc18000688 | 1 6 | mc9000480 | 3 4 7 |
| mc18000694 | 1 6 7 | mc9000630 | 10 13 21 |
| mc18000695 | 16 | mc9000632 | 10 13 |
| mc18000725 | 7 9 | mc9000636 | 10 13 |
| mc18000803 | 1 | mc9000651 | 6 7 11 21 |
| mc18000815 | 9 10 | mc9000680 | 9 10 12 |
| mc18000830 | 1 6 7 | mc9000722 | 7 9 |
| mc18000922 | 1 6 10 13 15 16 20 | mc9000727 | 3 7 11 16 17 21 |
| mc18000939 | 1 10 12 13 | mc9000790 | 1 16 21 |
| mc18000947 | 1 6 7 10 | mc9000922 | 6 7 15 17 21 |
| mc18001044 | 7 21 | mc9000963 | 6 7 21 |
| mc18001228 | 7 9 | mc9000965 | 7 9 |
| mc19000029 | 1 6 7 10 15 16 21 | mc9000973 | 9 10 12 21 22 |
| mc19000051 | 1 2 7 | mc9001038 | 7 9 11 21 |
| mc19000056 | 7 9 21 | mc9001087 | 1 2 7 21 |
| mc19000107 | 3 7 9 12 15 17 21 | mc9001112 | 4 10 15 16 21 |
| mc19000130 | 6 7 17 21 | mc9001131 | 7 21 |
| mc19000131 | 7 9 10 | mc9001150 | 4 7 8 10 16 21 |
| mc19000147 | 7 13 17 21 23 | mc9001162 | 6 7 8 10 21 |
| mc19000153 | 4 7 21 | mc9001203 | 6 21 |
| mc19000189 | 11 21 | mc9001208 | 1 6 7 10 15 |
| mc19000243 | 7 10 21 | mc9001219 | 7 9 10 |
| mc19000276 | 1 2 9 | mc9001221 | 5 7 21 |
| mc19000350 | 10 13 | mc9001224 | 7 13 17 19 21 |
| mc19000353 | 10 13 | mc9001287 | 2 7 9 17 21 |
| mc19000456 | 7 8 10 21 | mc9001301 | 1 7 9 13 16 18 |
| mc19000465 | 7 8 10 21 | mc9001362 | 1 4 7 15 16 21 |
| mc19000519 | 1 7 9 21 | mc9001379 | 7 17 21 |
| mc19000520 | 1 7 9 21 24 | mc9001419 | 11 |
| mc19000533 | 6 | mc9001525 | 1 2 7 21 |
| mc19000561 | 7 9 24 | mc9001581 | 7 13 15 16 20 21 |
| mc19000568 | 1 7 10 | mc9001590 | 7 12 19 |
| mc19000716 | 1 7 | mc9001615 | 7 9 12 |
| mc19000739 | 7 13 17 21 23 | mc9001724 | 7 10 12 21 |
| mc19000755 | 1 21 | mc9001747 | 1 6 7 10 12 16 21 |
| mc19000819 | 1 7 9 11 13 21 | mc9001769 | 7 9 10 21 |
| mc19000822 | 1 7 9 11 13 21 | mc9001825 | 1 7 10 21 |
| mc19000823 | 1 9 11 21 | mc9001853 | 1 6 7 21 |
| mc19000824 | 1 7 9 11 13 21 | mc9001874 | 7 10 |
| mc19000827 | 1 9 11 21 24 | mc9001876 | 7 10 12 13 |
| mc19000830 | 1 9 11 21 24 | mc9001907 | 7 10 12 16 17 21 |
| mc19000855 | 6 21 | mc9001928 | 1 6 7 8 9 10 |
| mc19000932 | 1 7 11 12 21 | mc9001981 | 2 7 9 17 19 21 |
| mc19000933 | 1 7 11 12 21 | mc9002125 | 9 21 |
| mc19000934 | 1 7 21 | mc9002155 | 5 |
| mc19000956 | 9 21 | mc9002249 | 7 10 12 13 15 19 |
| mc19001001 | 7 9 21 | mc9002280 | 4 7 9 21 |
| mc19001053 | 9 10 21 | mcx000005 | 9 |
| mc19001068 | 1 9 10 21 | mcx000056 | 9 13 |
| mc19001130 | 4 7 10 21 | mcx000203 | 1 7 9 21 |
| mc19001181 | 1 13 21 | mcx000242 | 1 3 6 7 8 10 15 21 |
| mc19001184 | 1 21 | mcx000297 | 7 9 21 23 |
| mc19001187 | 6 7 11 | mcx000300 | 3 6 7 11 21 |
| mc19001199 | 9 21 | mcx000302 | 3 7 |
| mc2000041 | 1 6 7 | mcx000325 | 4 7 21 |
| mc2000055 | 6 7 10 15 | mcx000362 | 7 9 10 12 13 21 22 24 |
| mc2000076 | 4 7 21 | mcx000372 | 3 7 17 21 |
| mc2000131 | 1 7 | mcx000398 | 3 4 7 11 |
| mc2000154 | 7 9 21 | mcx000451 | 1 6 7 10 15 21 |
| mc2000161 | 7 9 13 17 21 | mcx000456 | 7 16 19 21 |
| mc2000210 | 3 4 7 15 18 21 | mcx000530 | 4 7 16 21 |
| mc2000212 | 21 | mcx000643 | 3 4 7 13 17 18 19 21 |
| mc2000218 | 1 10 | mcx000659 | 3 4 7 21 |
| mc2000269 | 1 7 21 23 24 | mcx000677 | 7 9 12 21 22 24 |
| mc2000325 | 7 17 21 | mcx000681 | 1 6 7 10 21 |
| mc2000434 | 4 7 10 21 | mcx000684 | 7 9 21 |
| mc2000528 | 6 7 11 21 | mcx000714 | 10 16 21 |
| mc2000562 | 7 9 21 | mcx000759 | 7 9 10 |
| mc2000601 | 4 7 12 16 18 21 | mcx000914 | 1 2 7 8 21 23 |
| mc2000642 | 7 21 22 | mcx000960 | 7 17 21 |
| mc2000671 | 1 7 11 13 21 | mcx001007 | 7 12 15 16 21 |
| mc2000698 | 10 13 | mcx001110 | 1 10 11 12 15 21 |
| mc2000822 | 2 7 9 12 16 17 21 | mcx001116 | 1 2 6 7 |
| mc2000865 | 7 9 10 | mcx001301 | 4 7 |
| mc2000960 | 3 7 21 | mcx001348 | 5 |
| mc2001006 | 7 9 21 | mcx001364 | 3 4 15 21 |
| mc2001057 | 1 4 7 21 | mcx001465 | 1 6 7 10 16 |
| mc2001112 | 2 7 17 | mcx001467 | 7 10 15 16 20 21 |
| mc2001169 | 2 7 9 21 | mcx001628 | 1 5 6 7 |
| mc2001303 | 1 11 21 | mcx001629 | 5 7 21 |
| mc2001570 | 10 13 | mcx001639 | 1 2 7 21 |
| mc2001590 | 10 13 | mcx001643 | 1 2 6 7 |
| mc2001594 | 10 13 | mcx001677 | 3 4 6 7 15 21 |
| mc2001597 | 10 13 | mcx001682 | 1 |
| mc2001603 | 10 13 | mcx001693 | 6 7 16 21 |
| mc2001613 | 10 13 | mcx001707 | 9 10 12 |
| mc2001617 | 10 13 | mcx001736 | 1 2 6 7 |
| mc2001618 | 10 13 | mcx001754 | 6 7 17 21 |
| mc2001623 | 10 13 | mcx001785 | 1 7 |
