# Supplementary material for: Predicting Functions of Proteins in Mouse Based on Weighted Protein-Protein Interaction Network and Protein Hybrid Properties
Source: PLoS One. 2011 Jan 19;6(1):e14556. doi: 10.1371/journal.pone.0014556 (PMC3023709; doi:10.1371/journal.pone.0014556)
Supplement: Table S3 — Training set for hybrid-property based method. The Mfun ID and Functional number (see Table 1) of proteins are shown. (1.65 MB DOC) [file pone.0014556.s003.doc]

Table S3. Training set for hybrid-property based method. The Mfun ID and Functional number (see Table 1) of proteins are shown.

| MfunGD ID | Functional number(s) | MfunGD ID | Functional number(s) |
| --- | --- | --- | --- |
| mc10000007 | 8 10 | mc2001915 | 21 |
| mc10000055 | 7 9 21 | mc2001921 | 4 7 |
| mc10000076 | 7 | mc2001967 | 7 9 21 22 |
| mc10000125 | 6 7 16 17 19 21 | mc2001996 | 7 21 |
| mc10000128 | 7 9 21 | mc2002058 | 10 |
| mc10000129 | 4 7 21 | mc2002067 | 7 10 21 |
| mc10000142 | 20 21 | mc2002089 | 6 7 21 |
| mc10000155 | 6 7 16 20 21 | mc2002090 | 8 |
| mc1000018 | 21 | mc2002095 | 7 10 15 16 20 21 |
| mc10000195 | 8 | mc2002101 | 1 3 |
| mc10000237 | 3 4 7 9 21 | mc2002103 | 7 |
| mc10000240 | 1 2 7 21 | mc2002112 | 7 |
| mc10000248 | 4 7 | mc2002119 | 1 7 10 |
| mc10000267 | 9 21 | mc2002121 | 1 13 21 |
| mc10000281 | 5 7 21 | mc2002126 | 7 9 10 |
| mc10000288 | 10 12 21 | mc2002130 | 6 7 16 19 21 23 |
| mc10000302 | 1 7 | mc2002159 | 4 21 |
| mc10000317 | 6 9 21 | mc2002163 | 7 17 21 |
| mc1000033 | 7 8 10 12 13 21 23 24 | mc2002168 | 5 21 |
| mc10000352 | 15 | mc2002182 | 7 21 22 23 24 |
| mc10000354 | 1 2 21 | mc2002193 | 1 21 |
| mc10000355 | 15 21 | mc2002197 | 7 9 21 |
| mc10000356 | 8 10 21 | mc2002228 | 4 7 21 |
| mc10000360 | 6 7 21 | mc2002238 | 4 21 |
| mc10000401 | 6 7 13 21 | mc2002260 | 6 10 |
| mc10000402 | 9 | mc2002261 | 7 9 17 21 |
| mc10000429 | 1 21 | mc2002280 | 4 7 10 21 |
| mc1000045 | 1 6 | mc2002281 | 2 7 9 21 |
| mc10000451 | 3 17 21 | mc2002311 | 7 |
| mc10000452 | 10 20 21 | mc2002326 | 7 10 |
| mc10000466 | 3 7 21 | mc2002358 | 9 21 |
| mc10000474 | 9 | mc2002383 | 7 12 |
| mc10000475 | 6 9 | mc2002452 | 4 7 |
| mc10000488 | 9 21 | mc2002475 | 7 |
| mc10000493 | 3 4 6 7 21 | mc2002543 | 4 7 21 |
| mc10000503 | 16 22 | mc2002631 | 6 7 9 21 |
| mc10000509 | 4 6 7 21 24 | mc2002637 | 8 |
| mc10000517 | 3 11 21 | mc2002671 | 21 |
| mc1000052 | 3 | mc2002677 | 7 |
| mc10000523 | 3 7 21 | mc2002686 | 7 21 |
| mc10000578 | 6 21 | mc2002702 | 6 7 8 10 |
| mc10000580 | 5 7 21 | mc2002704 | 8 |
| mc10000601 | 4 7 21 | mc2002706 | 1 4 6 7 8 15 21 |
| mc10000608 | 7 8 10 13 21 | mc2002717 | 11 13 |
| mc10000612 | 4 7 21 | mc2002718 | 11 13 21 23 |
| mc10000619 | 21 | mc2002725 | 7 |
| mc1000062 | 1 6 7 10 15 21 | mc2002751 | 4 15 21 |
| mc10000622 | 1 | mc2002754 | 6 |
| mc10000624 | 4 7 21 | mc2002775 | 7 |
| mc10000692 | 7 12 | mc2002779 | 24 |
| mc10000694 | 3 4 7 21 | mc2002781 | 7 |
| mc10000698 | 7 | mc2002792 | 3 4 7 15 21 |
| mc10000705 | 7 | mc2002795 | 7 21 |
| mc10000716 | 10 21 | mc2002804 | 1 7 21 |
| mc10000736 | 1 6 8 10 | mc2002806 | 6 7 |
| mc10000741 | 10 21 | mc2002811 | 3 21 |
| mc10000744 | 6 21 | mc2002823 | 7 10 13 21 |
| mc10000745 | 15 21 | mc2002830 | 3 7 17 21 |
| mc10000746 | 7 21 | mc2002837 | 7 21 |
| mc10000756 | 21 | mc2002843 | 4 7 8 21 |
| mc10000762 | 1 2 7 | mc2002847 | 15 |
| mc10000783 | 7 8 21 22 23 24 | mc2002857 | 12 21 |
| mc10000848 | 7 10 | mc2002863 | 10 |
| mc10000853 | 6 17 21 | mc2002880 | 16 |
| mc10000889 | 7 8 21 | mc2002889 | 1 7 |
| mc10000904 | 1 10 21 | mc2002890 | 1 |
| mc10000941 | 7 9 10 21 | mc2002898 | 10 |
| mc10000958 | 21 | mc2002941 | 1 7 10 21 |
| mc10000960 | 7 10 21 | mc2002952 | 3 21 |
| mc10000974 | 7 21 | mc2002965 | 1 6 |
| mc1000101 | 9 | mc2002987 | 9 |
| mc10001026 | 21 | mc2002994 | 9 12 21 |
| mc10001029 | 10 | mc2003012 | 6 8 21 |
| mc10001037 | 6 9 21 | mc2003019 | 7 21 |
| mc10001043 | 4 6 7 8 17 21 | mc2003026 | 8 24 |
| mc10001049 | 21 | mc2003036 | 7 |
| mc1000105 | 4 7 17 21 | mc2003037 | 7 21 |
| mc10001067 | 7 21 | mc2003043 | 4 7 21 |
| mc10001069 | 7 16 21 | mc2003049 | 5 7 10 13 15 18 21 |
| mc10001075 | 3 6 21 | mc2003054 | 7 12 17 21 24 |
| mc10001083 | 10 15 21 | mc2003061 | 7 21 |
| mc10001091 | 6 13 | mc2003064 | 3 4 6 7 8 15 21 |
| mc10001104 | 4 7 21 | mc2003069 | 1 |
| mc10001111 | 21 | mc2003082 | 8 10 |
| mc10001126 | 21 | mc2003087 | 8 9 21 |
| mc10001127 | 1 6 7 | mc2003105 | 7 9 12 |
| mc10001129 | 1 7 21 | mc2003127 | 15 |
| mc10001138 | 1 3 5 21 | mc2003130 | 1 21 |
| mc10001143 | 1 6 7 10 | mc2003131 | 1 7 |
| mc10001155 | 4 7 21 | mc2003141 | 7 21 |
| mc10001185 | 5 21 | mc2003199 | 21 |
| mc10001190 | 10 | mc2003251 | 2 7 10 11 21 |
| mc10001206 | 21 | mc2003254 | 7 10 21 |
| mc10001221 | 4 7 21 | mc2003274 | 6 |
| mc10001232 | 4 21 | mc2003276 | 7 12 |
| mc10001237 | 9 | mc2003307 | 4 7 8 20 21 |
| mc10001252 | 7 17 21 | mc2003328 | 9 |
| mc10001263 | 4 | mc2003336 | 4 21 24 |
| mc10001278 | 7 | mc2003337 | 4 7 15 21 |
| mc10001311 | 7 10 15 16 18 21 | mc2003338 | 9 |
| mc10001316 | 6 9 17 21 | mc2003344 | 21 |
| mc1000134 | 6 9 21 | mc2003362 | 15 |
| mc1000137 | 9 11 12 13 | mc2003364 | 7 |
| mc10001391 | 3 4 7 20 21 | mc2003365 | 21 |
| mc1000140 | 9 10 12 21 | mc2003367 | 4 7 21 |
| mc10001410 | 7 8 21 | mc2003379 | 4 5 7 21 |
| mc10001429 | 5 21 | mc2003383 | 4 7 15 16 21 |
| mc10001440 | 9 10 21 | mc2003394 | 7 |
| mc10001464 | 21 | mc2003399 | 17 21 |
| mc10001467 | 17 20 21 24 | mc2003417 | 7 |
| mc10001479 | 7 9 21 | mc2003425 | 1 2 6 7 10 21 |
| mc10001484 | 1 7 21 | mc2003433 | 15 |
| mc10001491 | 1 6 8 10 | mc2003437 | 6 7 9 21 |
| mc10001519 | 1 | mc2003446 | 7 13 15 21 |
| mc10001557 | 7 9 24 | mc2003451 | 4 7 21 |
| mc10001564 | 6 13 17 21 | mc2003459 | 4 7 21 |
| mc10001569 | 3 4 7 15 21 | mc3000051 | 7 8 |
| mc10001574 | 7 9 12 21 | mc3000053 | 7 21 |
| mc1000158 | 4 7 21 | mc3000056 | 21 |
| mc10001623 | 7 | mc3000073 | 7 |
| mc10001625 | 9 10 12 21 | mc3000118 | 8 9 10 13 21 |
| mc10001627 | 9 10 12 21 | mc3000144 | 4 6 7 17 21 |
| mc10001642 | 7 | mc3000152 | 1 3 4 6 7 21 |
| mc10001645 | 10 | mc3000155 | 6 21 24 |
| mc10001686 | 4 7 21 | mc3000160 | 4 7 21 |
| mc10001704 | 21 | mc3000198 | 21 |
| mc10001716 | 6 21 23 24 | mc3000248 | 7 21 |
| mc10001720 | 4 7 | mc3000250 | 12 17 21 |
| mc10001732 | 6 7 10 12 21 | mc3000286 | 7 21 |
| mc10001744 | 3 4 7 17 21 | mc3000291 | 5 21 |
| mc10001753 | 17 21 | mc3000303 | 7 |
| mc1000179 | 16 21 | mc3000311 | 6 7 9 11 21 |
| mc1000181 | 11 13 | mc3000433 | 1 6 7 10 |
| mc10001846 | 1 6 21 | mc3000438 | 7 |
| mc10001851 | 1 7 9 13 21 | mc3000443 | 10 21 |
| mc10001854 | 15 21 | mc3000481 | 4 7 21 |
| mc10001861 | 6 7 10 21 | mc3000513 | 4 7 21 |
| mc10001863 | 2 7 9 17 21 | mc3000519 | 4 7 10 21 |
| mc10001877 | 7 21 | mc3000627 | 5 7 21 |
| mc10001879 | 2 9 | mc3000642 | 10 |
| mc10001888 | 7 21 | mc3000643 | 10 |
| mc10001889 | 10 21 24 | mc3000679 | 7 10 21 |
| mc10001895 | 1 9 | mc3000689 | 10 |
| mc10001910 | 1 21 | mc3000703 | 9 |
| mc10001914 | 21 | mc3000704 | 1 7 21 |
| mc10001918 | 1 3 4 6 21 | mc3000708 | 10 |
| mc10001922 | 4 7 | mc3000765 | 5 7 21 |
| mc10001923 | 9 12 21 | mc3000767 | 8 |
| mc10001926 | 7 | mc3000780 | 9 |
| mc10001932 | 7 | mc3000782 | 10 21 |
| mc10001935 | 6 10 21 | mc3000805 | 7 |
| mc10001943 | 7 9 10 17 21 | mc3000876 | 7 8 10 13 21 |
| mc10001953 | 21 | mc3000910 | 9 10 21 24 |
| mc10001954 | 4 5 7 21 | mc3000950 | 6 7 8 9 21 |
| mc10001955 | 10 16 19 21 23 | mc3000952 | 21 |
| mc1000198 | 7 10 | mc3000969 | 7 |
| mc1000201 | 6 9 | mc3000980 | 7 10 15 16 18 19 20 21 24 |
| mc10002036 | 13 | mc3000986 | 7 13 15 18 21 |
| mc1000207 | 11 16 21 | mc3000995 | 6 21 |
| mc1000215 | 7 9 12 17 21 | mc3000998 | 10 |
| mc1000240 | 7 10 16 19 20 21 24 | mc3000999 | 10 |
| mc1000299 | 21 | mc3001004 | 9 10 17 24 |
| mc1000328 | 7 8 10 12 21 | mc3001013 | 1 |
| mc1000346 | 6 21 | mc3001014 | 1 3 21 |
| mc1000347 | 7 | mc3001020 | 7 |
| mc1000348 | 7 | mc3001023 | 4 8 10 17 21 |
| mc1000385 | 1 6 21 | mc3001029 | 21 |
| mc1000395 | 4 7 15 24 | mc3001033 | 10 |
| mc1000440 | 9 12 21 | mc3001036 | 7 16 20 21 |
| mc1000475 | 5 21 | mc3001065 | 7 |
| mc1000480 | 10 | mc3001071 | 1 |
| mc1000481 | 7 10 21 | mc3001085 | 1 21 |
| mc1000518 | 7 9 10 13 | mc3001097 | 19 20 21 23 24 |
| mc1000548 | 6 11 | mc3001130 | 9 12 21 |
| mc1000600 | 1 6 | mc3001134 | 7 9 21 |
| mc1000611 | 4 6 7 8 15 21 | mc3001184 | 18 19 20 21 |
| mc1000634 | 1 7 10 21 | mc3001212 | 7 20 |
| mc1000645 | 4 7 11 21 | mc3001228 | 8 10 |
| mc1000675 | 6 | mc3001235 | 5 21 |
| mc1000687 | 6 7 15 | mc3001247 | 7 16 21 |
| mc1000692 | 1 6 7 10 15 17 | mc3001270 | 7 |
| mc1000695 | 7 | mc3001280 | 7 |
| mc1000697 | 8 | mc3001281 | 21 |
| mc1000707 | 7 10 21 | mc3001305 | 4 7 21 |
| mc1000713 | 9 | mc3001306 | 12 21 |
| mc1000761 | 1 | mc3001325 | 2 7 8 11 21 |
| mc1000775 | 4 7 15 16 21 | mc3001331 | 12 21 |
| mc1000852 | 6 21 | mc3001336 | 4 6 7 21 |
| mc1000855 | 7 21 | mc3001338 | 4 6 7 21 |
| mc1000879 | 4 7 21 | mc3001342 | 10 |
| mc1000911 | 6 7 12 17 21 | mc3001347 | 10 18 21 22 |
| mc1000921 | 6 | mc3001370 | 7 21 |
| mc1000925 | 4 21 | mc3001375 | 1 21 |
| mc1000933 | 1 9 | mc3001379 | 1 2 6 7 9 21 |
| mc1000938 | 7 8 17 | mc3001384 | 4 7 21 |
| mc1000957 | 6 7 11 21 | mc3001409 | 6 7 21 |
| mc1000966 | 1 7 17 21 | mc3001410 | 4 7 21 |
| mc1001006 | 1 21 | mc3001411 | 10 13 21 22 24 |
| mc1001007 | 1 21 | mc3001448 | 1 13 23 |
| mc1001008 | 5 21 | mc3001449 | 3 7 8 18 |
| mc1001014 | 1 2 7 21 | mc3001462 | 6 9 17 21 |
| mc1001032 | 5 21 | mc3001467 | 7 21 |
| mc1001044 | 8 | mc3001496 | 1 11 |
| mc1001069 | 21 | mc3001503 | 2 7 9 21 |
| mc1001073 | 7 12 17 21 | mc3001516 | 1 21 |
| mc1001093 | 13 15 21 23 | mc3001528 | 9 12 21 |
| mc1001111 | 6 9 | mc3001530 | 10 13 21 24 |
| mc1001113 | 7 21 | mc3001532 | 3 7 |
| mc1001118 | 7 10 21 | mc3001551 | 11 21 |
| mc1001124 | 6 7 8 11 21 | mc3001567 | 8 10 11 15 |
| mc1001126 | 6 7 8 | mc3001592 | 9 21 |
| mc1001129 | 1 6 | mc3001598 | 7 8 9 10 12 |
| mc1001157 | 7 | mc3001615 | 1 7 11 21 |
| mc1001160 | 9 21 | mc3001622 | 7 |
| mc1001167 | 1 11 | mc3001628 | 7 21 |
| mc1001173 | 6 15 21 | mc3001643 | 3 6 7 21 23 24 |
| mc1001182 | 1 7 11 13 21 | mc3001659 | 11 |
| mc1001196 | 7 21 | mc3001744 | 6 10 21 |
| mc1001229 | 9 10 21 | mc3001754 | 1 7 |
| mc1001242 | 6 7 | mc3001773 | 6 7 |
| mc1001243 | 10 21 | mc3001774 | 5 6 7 17 21 |
| mc1001262 | 13 | mc3001815 | 1 7 21 |
| mc1001268 | 7 24 | mc3001823 | 21 |
| mc1001277 | 7 10 16 21 | mc3001863 | 1 6 7 10 |
| mc1001283 | 21 | mc3001871 | 4 7 |
| mc1001296 | 21 | mc3001909 | 6 8 10 11 12 13 15 18 22 |
| mc1001297 | 1 6 | mc3001985 | 7 23 |
| mc1001307 | 21 | mc3001989 | 7 13 |
| mc1001312 | 9 | mc3002034 | 7 9 10 21 22 |
| mc1001359 | 6 7 21 | mc3002038 | 6 17 21 23 24 |
| mc1001380 | 7 8 10 15 21 | mc3002051 | 7 10 15 22 |
| mc1001383 | 21 | mc3002063 | 4 5 7 21 |
| mc1001403 | 8 15 21 | mc3002065 | 1 3 21 |
| mc1001461 | 17 21 | mc3002141 | 6 7 11 |
| mc1001463 | 4 7 21 | mc3002148 | 21 |
| mc1001471 | 5 21 | mc3002150 | 1 7 |
| mc1001473 | 6 7 8 10 13 15 16 18 20 21 | mc3002151 | 6 21 |
| mc1001483 | 6 9 | mc3002192 | 6 7 |
| mc1001505 | 7 11 | mc3002194 | 1 |
| mc1001542 | 7 | mc3002226 | 21 |
| mc1001558 | 1 11 13 18 19 20 | mc3002228 | 22 24 |
| mc1001559 | 3 4 6 7 8 10 15 21 | mc3002253 | 7 |
| mc1001564 | 5 7 21 | mc3002255 | 4 7 10 21 |
| mc1001566 | 7 21 | mc4000008 | 1 6 7 8 10 18 |
| mc1001570 | 4 5 7 21 | mc4000019 | 6 8 10 21 |
| mc1001602 | 10 13 21 | mc4000068 | 1 6 21 |
| mc1001603 | 13 15 21 | mc4000085 | 7 |
| mc1001606 | 10 13 15 | mc4000094 | 3 8 21 |
| mc1001615 | 8 10 12 | mc4000103 | 3 21 24 |
| mc1001623 | 6 21 | mc4000106 | 7 10 |
| mc1001646 | 6 9 | mc4000109 | 6 21 |
| mc1001662 | 7 21 | mc4000114 | 7 |
| mc1001669 | 7 10 12 15 | mc4000195 | 6 |
| mc1001671 | 6 10 21 | mc4000292 | 6 7 17 21 |
| mc1001677 | 4 7 21 | mc4000307 | 7 21 |
| mc1001682 | 7 8 10 21 22 | mc4000319 | 21 22 |
| mc1001697 | 11 17 21 23 | mc4000326 | 21 |
| mc1001700 | 7 10 21 22 | mc4000330 | 9 21 |
| mc1001705 | 1 | mc4000339 | 1 7 |
| mc1001714 | 1 | mc4000400 | 8 21 |
| mc1001721 | 6 8 13 24 | mc4000406 | 7 21 |
| mc1001724 | 6 | mc4000425 | 1 7 |
| mc1001725 | 7 10 13 21 | mc4000427 | 7 |
| mc1001727 | 1 6 8 10 | mc4000450 | 4 7 12 13 21 |
| mc1001729 | 10 | mc4000456 | 21 |
| mc1001734 | 7 21 | mc4000469 | 3 8 21 |
| mc1001735 | 6 7 9 21 | mc4000471 | 17 18 21 22 |
| mc1001748 | 7 12 13 19 23 | mc4000485 | 7 |
| mc1001749 | 9 21 | mc4000488 | 4 7 21 |
| mc1001751 | 7 9 10 12 13 21 23 24 | mc4000497 | 1 7 |
| mc1001753 | 2 7 9 17 21 | mc4000503 | 10 12 17 |
| mc1001757 | 10 21 | mc4000516 | 4 5 9 21 |
| mc1001760 | 3 4 7 | mc4000559 | 4 7 21 |
| mc1001803 | 7 21 | mc4000574 | 5 21 |
| mc1001804 | 20 21 24 | mc4000580 | 7 9 10 12 13 16 21 |
| mc1001807 | 6 7 12 13 21 | mc4000597 | 3 7 21 |
| mc1001832 | 2 9 21 | mc4000599 | 4 21 |
| mc1001867 | 16 21 | mc4000622 | 16 21 |
| mc1001943 | 7 | mc4000623 | 4 7 21 |
| mc1001956 | 13 | mc4000625 | 3 7 17 21 |
| mc1001973 | 21 | mc4000673 | 7 16 17 21 22 24 |
| mc1001987 | 6 10 | mc4000678 | 24 |
| mc1001999 | 8 10 | mc4000681 | 3 6 7 8 10 15 17 21 |
| mc1002042 | 15 16 20 21 24 | mc4000687 | 15 |
| mc1002051 | 8 17 21 | mc4000722 | 4 7 21 |
| mc1002060 | 7 21 | mc4000741 | 7 9 21 |
| mc1002068 | 1 11 13 | mc4000750 | 3 6 21 |
| mc1002077 | 1 2 7 21 | mc4000751 | 4 6 7 21 |
| mc1002087 | 1 21 | mc4000752 | 7 |
| mc1002099 | 7 9 11 24 | mc4000760 | 7 8 10 21 |
| mc1002116 | 1 | mc4000784 | 7 9 21 |
| mc1002125 | 1 7 | mc4000802 | 4 7 8 11 21 24 |
| mc1002139 | 10 21 | mc4000832 | 15 21 |
| mc1002143 | 6 10 21 | mc4000965 | 4 21 |
| mc1002145 | 4 7 8 15 18 21 | mc4001004 | 4 16 21 |
| mc1002149 | 4 7 21 | mc4001015 | 7 10 11 12 15 21 |
| mc1002160 | 4 7 16 21 | mc4001079 | 21 |
| mc1002191 | 1 4 7 15 16 20 21 | mc4001137 | 7 12 15 17 19 21 24 |
| mc1002194 | 6 9 | mc4001145 | 7 8 |
| mc1002222 | 7 8 10 | mc4001169 | 7 |
| mc1002227 | 6 7 8 22 | mc4001186 | 1 6 7 8 10 |
| mc1002231 | 4 6 21 | mc4001191 | 1 7 21 24 |
| mc1002236 | 7 8 9 10 11 13 18 21 | mc4001199 | 7 10 12 21 |
| mc1002245 | 10 21 | mc4001202 | 8 10 21 |
| mc1002254 | 5 6 7 | mc4001222 | 9 |
| mc1002260 | 7 17 21 | mc4001223 | 4 7 21 |
| mc1002289 | 7 9 10 15 21 | mc4001227 | 6 7 |
| mc1002301 | 19 | mc4001252 | 7 8 10 12 15 16 |
| mc1002321 | 7 10 21 22 | mc4001290 | 7 21 |
| mc1002326 | 13 21 | mc4001292 | 9 |
| mc1002335 | 13 21 | mc4001296 | 5 |
| mc1002337 | 13 21 | mc4001331 | 7 10 21 23 24 |
| mc1002354 | 7 16 17 21 | mc4001338 | 9 |
| mc1002368 | 6 7 8 9 10 | mc4001344 | 7 |
| mc1002378 | 7 21 | mc4001404 | 7 9 |
| mc1002403 | 6 9 | mc4001421 | 7 |
| mc1002408 | 4 5 7 17 21 | mc4001439 | 1 6 7 21 |
| mc1002410 | 7 | mc4001475 | 7 21 |
| mc1002429 | 6 7 9 21 | mc4001491 | 1 7 21 |
| mc1002436 | 3 6 7 8 10 | mc4001537 | 7 21 |
| mc1002470 | 10 21 | mc4001542 | 7 15 21 |
| mc1002474 | 6 | mc4001557 | 4 7 21 |
| mc1002497 | 10 13 21 | mc4001567 | 4 7 21 |
| mc1002521 | 21 | mc4001577 | 7 9 21 |
| mc1002530 | 5 6 7 21 | mc4001591 | 4 |
| mc1002560 | 1 2 7 15 21 | mc4001613 | 4 21 |
| mc1002569 | 7 | mc4001615 | 10 15 16 |
| mc1002587 | 12 13 15 21 | mc4001625 | 4 7 21 |
| mc1002605 | 3 21 | mc4001637 | 2 7 9 21 |
| mc1002625 | 5 6 7 21 | mc4001658 | 4 7 16 21 |
| mc1002635 | 7 21 | mc4001669 | 1 10 |
| mc1002648 | 1 21 | mc4001694 | 13 21 |
| mc1002675 | 4 7 21 | mc4001700 | 21 |
| mc11000010 | 7 | mc4001704 | 1 6 7 |
| mc11000020 | 7 | mc4001715 | 13 17 21 24 |
| mc11000031 | 1 6 8 10 21 | mc4001716 | 6 |
| mc11000032 | 2 | mc4001718 | 5 |
| mc11000039 | 4 7 9 21 | mc4001727 | 4 7 21 |
| mc11000041 | 21 | mc4001730 | 7 |
| mc11000044 | 7 | mc4001740 | 21 |
| mc11000052 | 10 13 21 | mc4001765 | 7 21 |
| mc11000062 | 7 21 | mc4001768 | 1 |
| mc11000063 | 7 10 | mc4001771 | 7 |
| mc11000070 | 7 10 | mc4001779 | 6 9 |
| mc11000071 | 3 21 | mc4001791 | 1 6 7 15 16 |
| mc11000087 | 4 7 18 21 22 24 | mc4001795 | 3 4 6 7 21 |
| mc11000096 | 1 3 7 21 | mc4001820 | 12 21 22 |
| mc11000114 | 3 7 17 21 | mc4001830 | 5 7 21 |
| mc11000151 | 3 6 11 | mc4001831 | 4 7 21 |
| mc11000212 | 6 9 21 | mc4001834 | 7 17 |
| mc11000290 | 7 | mc4001839 | 7 17 21 |
| mc11000406 | 5 21 | mc4001873 | 3 7 9 17 21 |
| mc11000413 | 15 | mc4001881 | 4 7 21 |
| mc11000458 | 7 13 16 21 22 23 | mc4001883 | 1 4 6 7 8 10 16 21 |
| mc11000467 | 6 | mc4001885 | 1 11 21 |
| mc11000510 | 15 21 | mc4001917 | 7 |
| mc11000512 | 1 21 | mc4001928 | 3 7 17 21 |
| mc11000564 | 7 9 10 12 21 | mc4001934 | 7 21 |
| mc11000580 | 7 | mc4001940 | 10 |
| mc11000598 | 1 6 7 10 15 21 | mc4001944 | 7 8 10 21 |
| mc11000679 | 3 6 7 21 | mc4001960 | 7 19 |
| mc11000686 | 7 | mc4001978 | 2 12 |
| mc11000715 | 15 21 | mc4001993 | 10 |
| mc11000723 | 1 6 7 8 10 11 18 21 | mc4001996 | 4 6 7 9 21 |
| mc11000735 | 1 8 21 | mc4002001 | 1 7 |
| mc11000784 | 6 9 | mc4002012 | 4 7 21 |
| mc11000837 | 4 13 21 | mc4002019 | 7 |
| mc11000902 | 21 | mc4002023 | 21 |
| mc11000923 | 7 21 | mc4002043 | 1 6 7 9 13 21 24 |
| mc11000925 | 7 21 | mc4002054 | 8 |
| mc11000938 | 5 21 | mc4002061 | 5 7 21 |
| mc11000946 | 21 | mc4002062 | 5 7 21 |
| mc11000961 | 4 7 21 | mc4002064 | 3 7 21 |
| mc11000969 | 7 17 21 24 | mc4002069 | 1 21 |
| mc11000975 | 9 10 21 | mc4002086 | 7 |
| mc11000976 | 1 7 9 | mc4002115 | 7 12 |
| mc11000999 | 8 | mc4002127 | 6 7 |
| mc11001003 | 1 7 21 | mc4002148 | 6 7 11 21 |
| mc11001021 | 7 | mc4002162 | 13 21 |
| mc11001063 | 7 | mc4002164 | 1 7 21 |
| mc11001066 | 6 7 | mc4002165 | 6 7 11 |
| mc11001067 | 7 8 10 21 | mc4002168 | 6 7 8 21 24 |
| mc11001095 | 4 7 21 | mc4002194 | 4 21 |
| mc11001099 | 4 7 21 | mc4002220 | 10 21 |
| mc11001105 | 7 8 12 17 21 | mc4002227 | 3 21 |
| mc11001116 | 1 6 21 | mc4002244 | 6 7 9 21 |
| mc11001141 | 4 7 8 15 19 20 21 | mc4002268 | 3 4 7 17 21 |
| mc11001156 | 7 12 17 21 | mc4002277 | 10 20 21 |
| mc11001175 | 2 7 8 9 21 24 | mc4002278 | 11 12 13 21 |
| mc11001179 | 2 12 13 16 17 19 21 23 | mc4002301 | 6 13 |
| mc11001190 | 10 15 16 21 24 | mc4002316 | 7 21 |
| mc11001193 | 6 21 | mc4002320 | 10 21 |
| mc11001208 | 7 | mc4002325 | 2 7 9 17 21 |
| mc11001256 | 7 13 15 16 20 21 | mc4002330 | 1 21 |
| mc11001268 | 9 15 16 21 | mc4002342 | 1 2 7 21 |
| mc11001271 | 3 7 8 10 21 | mc4002344 | 10 |
| mc11001272 | 4 8 10 | mc4002346 | 9 |
| mc11001276 | 9 21 23 24 | mc4002357 | 9 |
| mc11001288 | 7 9 10 | mc4002371 | 4 7 10 21 |
| mc11001290 | 7 | mc4002388 | 4 7 21 |
| mc11001294 | 21 | mc4002391 | 6 7 |
| mc11001295 | 6 7 | mc4002396 | 17 21 |
| mc11001309 | 24 | mc4002403 | 6 21 |
| mc11001313 | 1 6 7 10 18 | mc4002406 | 5 7 21 |
| mc11001353 | 9 12 13 21 24 | mc4002407 | 3 7 21 |
| mc11001363 | 6 7 | mc4002454 | 7 17 21 |
| mc11001372 | 7 10 12 21 | mc4002469 | 6 7 9 17 21 |
| mc11001442 | 1 | mc4002495 | 4 6 7 10 21 |
| mc11001458 | 5 15 21 | mc4002505 | 3 4 21 |
| mc11001462 | 10 | mc4002508 | 6 7 9 21 |
| mc11001471 | 21 | mc4002516 | 7 21 |
| mc11001474 | 7 8 9 21 | mc4002519 | 21 |
| mc11001475 | 9 10 21 | mc4002522 | 7 13 |
| mc11001479 | 2 7 9 21 | mc4002523 | 10 21 |
| mc11001481 | 1 3 17 21 | mc4002527 | 21 |
| mc11001502 | 8 20 | mc4002531 | 13 |
| mc11001509 | 7 | mc4002535 | 7 |
| mc11001513 | 10 | mc4002536 | 21 |
| mc11001519 | 7 | mc4002546 | 10 12 13 15 21 |
| mc11001528 | 8 10 | mc4002555 | 7 |
| mc11001559 | 1 2 21 24 | mc4002561 | 10 |
| mc11001566 | 7 10 13 | mc4002565 | 4 7 10 21 |
| mc11001569 | 4 7 21 | mc4002574 | 1 4 6 7 8 10 16 21 |
| mc11001574 | 21 | mc4002581 | 7 |
| mc11001595 | 11 21 | mc5000012 | 10 |
| mc11001614 | 6 7 | mc5000017 | 11 21 |
| mc11001615 | 6 | mc5000039 | 6 9 |
| mc11001621 | 2 7 9 17 21 | mc5000078 | 4 7 21 |
| mc11001623 | 21 | mc5000091 | 7 17 |
| mc11001656 | 3 7 21 | mc5000092 | 7 17 |
| mc11001669 | 15 | mc5000093 | 7 17 |
| mc11001677 | 6 7 | mc5000094 | 7 |
| mc11001700 | 1 2 7 10 12 13 21 23 | mc5000131 | 21 |
| mc11001718 | 1 2 7 21 | mc5000154 | 7 10 13 21 22 23 24 |
| mc11001742 | 14 | mc5000186 | 21 |
| mc11001745 | 7 | mc5000189 | 6 7 21 |
| mc11001751 | 7 9 21 | mc5000190 | 6 7 11 21 |
| mc11001758 | 10 21 | mc5000199 | 3 4 7 21 |
| mc11001762 | 8 21 | mc5000217 | 4 7 17 21 |
| mc11001775 | 3 4 7 21 | mc5000231 | 7 |
| mc11001790 | 5 21 | mc5000232 | 9 |
| mc11001805 | 3 7 17 21 | mc5000236 | 21 22 24 |
| mc11001828 | 21 | mc5000238 | 7 9 21 |
| mc11001834 | 9 10 21 | mc5000242 | 1 6 7 15 21 |
| mc11001882 | 1 | mc5000250 | 1 7 24 |
| mc11001895 | 7 13 17 19 21 23 | mc5000253 | 1 2 7 |
| mc11001899 | 7 8 17 22 | mc5000325 | 16 |
| mc11001969 | 7 10 13 21 22 | mc5000347 | 7 10 21 |
| mc11001970 | 9 10 | mc5000362 | 7 21 |
| mc11001979 | 7 21 | mc5000369 | 15 |
| mc11001985 | 7 10 21 | mc5000376 | 1 7 21 |
| mc11002013 | 5 21 | mc5000381 | 21 |
| mc11002019 | 7 9 10 12 13 | mc5000385 | 7 21 |
| mc11002026 | 4 7 8 20 21 | mc5000389 | 21 |
| mc11002038 | 3 6 8 10 15 21 | mc5000400 | 7 21 |
| mc11002041 | 4 7 15 16 21 | mc5000406 | 5 21 |
| mc11002043 | 16 21 | mc5000418 | 1 3 6 7 |
| mc11002058 | 7 8 10 13 16 21 24 | mc5000419 | 1 3 6 7 9 10 |
| mc11002063 | 3 7 21 | mc5000431 | 12 15 21 22 |
| mc11002070 | 21 | mc5000437 | 21 |
| mc11002081 | 7 21 | mc5000456 | 7 |
| mc11002084 | 21 | mc5000499 | 1 7 8 9 21 |
| mc11002095 | 21 | mc5000505 | 8 |
| mc11002125 | 7 17 20 21 24 | mc5000518 | 13 16 |
| mc11002140 | 7 9 21 | mc5000523 | 10 15 18 21 |
| mc11002143 | 3 21 | mc5000534 | 12 20 |
| mc11002157 | 22 24 | mc5000535 | 10 21 |
| mc11002160 | 9 21 24 | mc5000539 | 9 21 |
| mc11002162 | 20 21 | mc5000594 | 1 11 |
| mc11002165 | 4 7 10 21 | mc5000620 | 3 7 17 21 |
| mc11002179 | 7 12 | mc5000621 | 4 21 |
| mc11002181 | 7 12 21 | mc5000696 | 9 21 |
| mc11002183 | 10 | mc5000697 | 7 |
| mc11002186 | 1 6 7 9 12 17 21 | mc5000707 | 3 15 21 |
| mc11002190 | 6 7 8 21 | mc5000720 | 5 17 21 |
| mc11002191 | 1 7 | mc5000743 | 1 2 |
| mc11002195 | 1 2 21 | mc5000747 | 10 21 23 |
| mc11002207 | 7 | mc5000756 | 7 21 |
| mc11002208 | 6 7 9 21 | mc5000784 | 5 7 21 |
| mc11002209 | 4 7 9 21 | mc5000792 | 7 |
| mc11002224 | 1 21 | mc5000794 | 7 |
| mc11002225 | 21 | mc5000796 | 10 12 15 21 |
| mc11002238 | 9 21 | mc5000841 | 9 |
| mc11002260 | 10 | mc5000845 | 5 7 |
| mc11002262 | 2 7 9 21 | mc5000862 | 7 9 21 |
| mc11002268 | 1 7 10 | mc5000863 | 7 9 21 |
| mc11002272 | 4 7 21 | mc5000874 | 7 9 12 13 21 |
| mc11002280 | 7 8 9 21 | mc5000878 | 1 6 7 10 |
| mc11002306 | 3 7 21 | mc5000886 | 10 |
| mc11002319 | 3 6 7 11 16 21 | mc5000899 | 6 7 13 17 19 21 23 |
| mc11002322 | 7 | mc5000906 | 6 |
| mc11002333 | 1 6 7 11 21 | mc5000916 | 9 |
| mc11002335 | 9 21 | mc5000946 | 6 7 9 10 19 21 |
| mc11002340 | 1 6 7 10 | mc5000963 | 6 7 17 21 |
| mc11002341 | 7 | mc5001009 | 3 5 6 7 8 11 12 13 15 17 21 |
| mc11002348 | 10 12 21 | mc5001036 | 6 7 21 |
| mc11002350 | 10 21 | mc5001057 | 9 21 |
| mc11002363 | 6 10 21 | mc5001098 | 1 7 9 21 |
| mc11002371 | 4 7 | mc5001111 | 21 |
| mc11002372 | 21 | mc5001116 | 7 9 |
| mc11002411 | 9 | mc5001138 | 3 15 21 |
| mc11002521 | 12 13 | mc5001140 | 21 |
| mc11002531 | 7 9 | mc5001152 | 1 6 7 21 |
| mc11002533 | 9 21 | mc5001159 | 21 |
| mc11002534 | 21 | mc5001169 | 13 21 |
| mc11002538 | 3 17 21 | mc5001175 | 7 16 21 |
| mc11002546 | 5 7 21 | mc5001177 | 4 7 |
| mc11002562 | 6 7 21 | mc5001223 | 4 7 21 |
| mc11002564 | 7 10 | mc5001260 | 6 9 21 |
| mc11002576 | 4 7 15 16 21 | mc5001269 | 1 21 |
| mc11002581 | 1 6 7 8 | mc5001271 | 3 7 21 |
| mc11002584 | 1 7 15 18 21 | mc5001300 | 8 |
| mc11002588 | 10 | mc5001341 | 7 21 |
| mc11002593 | 6 | mc5001381 | 4 7 21 24 |
| mc11002620 | 9 21 | mc5001390 | 3 4 |
| mc11002632 | 1 | mc5001411 | 7 |
| mc11002671 | 1 21 | mc5001416 | 9 21 |
| mc11002680 | 7 | mc5001417 | 21 24 |
| mc11002686 | 1 6 7 10 15 | mc5001429 | 3 10 21 |
| mc11002727 | 1 7 9 21 | mc5001430 | 1 |
| mc11002743 | 1 6 7 10 | mc5001464 | 21 |
| mc11002754 | 6 | mc5001488 | 7 |
| mc11002771 | 7 12 16 20 21 | mc5001492 | 15 |
| mc11002801 | 4 7 10 12 13 15 20 21 | mc5001511 | 20 21 24 |
| mc12000005 | 7 9 17 21 | mc5001547 | 7 21 |
| mc12000011 | 1 3 4 7 16 21 | mc5001552 | 7 10 12 17 21 |
| mc12000016 | 7 10 | mc5001553 | 1 6 7 8 10 21 |
| mc12000027 | 7 17 21 | mc5001555 | 1 2 5 7 9 21 |
| mc12000033 | 17 | mc5001561 | 4 7 15 21 |
| mc12000068 | 7 16 18 | mc5001587 | 1 |
| mc12000073 | 1 | mc5001598 | 7 9 21 22 23 24 |
| mc12000090 | 7 | mc5001600 | 7 10 |
| mc12000111 | 1 | mc5001602 | 4 21 |
| mc12000115 | 1 3 7 | mc5001607 | 7 |
| mc12000134 | 5 7 21 | mc5001612 | 3 4 6 21 |
| mc12000150 | 3 7 21 | mc5001614 | 6 7 10 12 17 21 |
| mc12000172 | 1 19 21 | mc5001650 | 3 7 21 |
| mc12000186 | 6 7 21 | mc5001683 | 4 7 15 16 21 |
| mc12000194 | 7 13 21 24 | mc5001698 | 21 |
| mc12000205 | 4 7 21 | mc5001717 | 7 |
| mc12000209 | 1 | mc5001726 | 6 7 8 10 21 |
| mc12000212 | 10 13 22 | mc5001756 | 9 |
| mc12000230 | 21 | mc5001764 | 7 |
| mc12000266 | 7 | mc5001767 | 7 9 13 21 |
| mc12000282 | 11 13 | mc5001770 | 15 16 |
| mc12000315 | 6 7 9 21 | mc5001792 | 7 9 21 |
| mc12000317 | 10 21 23 24 | mc5001806 | 23 24 |
| mc12000320 | 4 7 10 21 | mc5001812 | 2 7 9 |
| mc12000322 | 6 7 10 21 | mc5001815 | 3 21 |
| mc12000338 | 15 18 21 23 | mc5001817 | 3 21 |
| mc12000339 | 9 21 | mc5001833 | 21 |
| mc12000360 | 7 8 9 10 | mc5001873 | 1 21 |
| mc12000378 | 7 | mc5001897 | 5 6 7 17 21 |
| mc12000410 | 2 7 9 10 21 | mc5001916 | 6 7 11 21 |
| mc12000432 | 1 10 21 | mc5001922 | 7 |
| mc12000435 | 7 12 21 | mc5001923 | 1 |
| mc12000450 | 4 7 21 22 | mc5001931 | 8 |
| mc12000478 | 5 6 7 8 21 | mc5001983 | 17 21 |
| mc12000482 | 9 21 | mc5001986 | 3 7 21 |
| mc12000500 | 7 8 10 15 21 | mc5002011 | 10 16 21 |
| mc12000511 | 4 7 10 13 21 | mc5002013 | 6 7 21 |
| mc12000541 | 3 4 7 17 21 | mc5002014 | 6 7 21 |
| mc12000542 | 1 21 | mc5002018 | 7 9 15 17 21 |
| mc12000573 | 4 7 21 | mc5002021 | 21 |
| mc12000582 | 1 16 19 20 | mc5002025 | 21 |
| mc12000589 | 5 7 21 | mc5002034 | 11 13 21 |
| mc12000598 | 7 | mc5002039 | 7 8 10 21 |
| mc12000652 | 1 3 4 7 11 21 | mc5002047 | 4 7 16 20 21 |
| mc12000653 | 21 | mc5002061 | 21 23 |
| mc12000689 | 21 | mc5002064 | 6 7 21 |
| mc12000702 | 1 6 7 10 | mc5002083 | 6 7 21 |
| mc12000726 | 7 | mc5002088 | 7 8 |
| mc12000728 | 6 7 9 21 | mc5002098 | 7 10 13 |
| mc12000753 | 21 | mc5002104 | 4 7 21 |
| mc12000798 | 4 7 9 10 | mc5002116 | 1 6 |
| mc12000816 | 4 7 10 13 20 21 | mc5002122 | 4 7 21 |
| mc12000820 | 4 7 | mc5002123 | 4 15 |
| mc12000821 | 4 7 21 | mc5002128 | 7 21 |
| mc12000849 | 1 7 17 21 | mc5002144 | 11 |
| mc12000862 | 1 12 13 21 23 | mc5002151 | 7 8 10 13 21 22 24 |
| mc12000888 | 1 3 7 | mc5002167 | 11 |
| mc12000901 | 9 12 13 21 23 24 | mc5002175 | 10 |
| mc12000922 | 7 8 10 | mc5002183 | 9 |
| mc12000933 | 4 7 21 | mc5002186 | 6 7 |
| mc12000936 | 10 13 | mc5002213 | 4 7 15 16 21 |
| mc12000952 | 1 21 | mc5002217 | 1 4 7 21 |
| mc12000970 | 7 16 21 | mc5002235 | 10 12 13 21 |
| mc12000981 | 6 7 | mc5002242 | 7 9 21 |
| mc12000987 | 1 5 6 7 | mc5002246 | 1 7 |
| mc12000991 | 3 21 | mc5002250 | 6 7 9 21 |
| mc12000992 | 1 | mc5002253 | 1 |
| mc12001000 | 4 7 21 | mc5002292 | 7 10 16 21 |
| mc12001018 | 21 | mc5002297 | 4 7 21 |
| mc12001080 | 4 21 | mc5002299 | 7 21 |
| mc12001110 | 10 12 21 | mc5002317 | 1 3 4 6 7 21 |
| mc12001114 | 4 5 6 7 21 | mc5002321 | 8 10 21 24 |
| mc12001137 | 7 | mc5002330 | 1 4 7 21 |
| mc12001140 | 3 4 7 | mc5002350 | 21 |
| mc12001180 | 9 21 | mc5002360 | 6 |
| mc12001182 | 7 8 21 | mc5002371 | 1 21 |
| mc12001202 | 7 | mc5002421 | 1 6 7 10 |
| mc12001203 | 2 7 9 21 | mc5002438 | 3 |
| mc12001209 | 21 | mc5002449 | 4 7 21 |
| mc12001216 | 12 15 | mc5002453 | 1 5 6 7 10 |
| mc12001241 | 8 | mc6000024 | 7 8 17 22 |
| mc12001255 | 1 3 4 7 16 18 | mc6000026 | 7 8 17 22 |
| mc12001268 | 22 23 | mc6000052 | 4 7 16 20 21 |
| mc12001336 | 7 13 17 21 | mc6000065 | 4 7 21 |
| mc12001337 | 3 10 17 | mc6000072 | 21 |
| mc12001350 | 5 7 | mc6000086 | 7 8 21 24 |
| mc12001390 | 1 6 7 10 | mc6000099 | 4 7 |
| mc12001401 | 7 10 | mc6000111 | 10 12 13 21 24 |
| mc12001414 | 1 6 7 10 | mc6000126 | 4 7 21 |
| mc12001423 | 7 | mc6000137 | 7 |
| mc12001458 | 3 | mc6000157 | 7 10 12 21 |
| mc12001465 | 4 7 21 | mc6000174 | 12 21 |
| mc12001581 | 21 | mc6000190 | 6 21 23 |
| mc12001613 | 7 16 21 | mc6000196 | 4 7 21 |
| mc12001618 | 12 21 | mc6000199 | 6 7 |
| mc12001631 | 7 10 21 | mc6000202 | 10 12 13 21 24 |
| mc13000006 | 8 9 10 21 | mc6000211 | 7 12 17 21 |
| mc13000009 | 10 | mc6000259 | 7 21 |
| mc13000023 | 1 2 7 9 11 21 | mc6000268 | 20 24 |
| mc13000061 | 1 7 21 | mc6000280 | 9 17 21 |
| mc13000103 | 7 10 15 16 20 | mc6000284 | 10 16 21 |
| mc13000115 | 1 | mc6000309 | 7 |
| mc13000123 | 6 8 21 | mc6000319 | 12 21 |
| mc13000127 | 6 7 11 21 | mc6000408 | 4 |
| mc13000131 | 4 7 16 20 21 | mc6000409 | 12 14 |
| mc13000146 | 1 | mc6000437 | 21 |
| mc13000151 | 6 7 18 21 | mc6000440 | 6 7 10 15 |
| mc13000177 | 10 | mc6000442 | 5 21 |
| mc13000224 | 10 13 21 | mc6000457 | 3 7 21 |
| mc13000230 | 7 17 21 | mc6000459 | 6 21 |
| mc13000242 | 3 7 17 21 | mc6000467 | 6 13 21 24 |
| mc13000261 | 10 21 | mc6000468 | 6 21 |
| mc13000273 | 10 21 | mc6000490 | 6 21 |
| mc13000332 | 3 7 17 21 | mc6000493 | 6 7 10 13 21 22 23 24 |
| mc13000350 | 9 12 13 21 | mc6000499 | 7 10 21 |
| mc13000377 | 1 7 9 13 21 | mc6000508 | 10 12 13 21 24 |
| mc13000379 | 12 | mc6000518 | 10 12 13 21 24 |
| mc13000400 | 10 | mc6000581 | 4 7 9 21 |
| mc13000407 | 21 | mc6000582 | 7 21 |
| mc13000420 | 7 10 11 15 21 | mc6000584 | 7 21 |
| mc13000448 | 3 4 7 15 21 | mc6000600 | 7 21 |
| mc13000490 | 6 7 10 13 15 23 24 | mc6000602 | 7 21 |
| mc13000544 | 24 | mc6000603 | 7 21 |
| mc13000558 | 1 6 | mc6000606 | 7 |
| mc13000585 | 4 7 10 16 21 | mc6000610 | 7 13 21 |
| mc13000587 | 21 24 | mc6000612 | 7 13 21 |
| mc13000590 | 4 7 21 | mc6000616 | 16 22 |
| mc13000595 | 7 9 13 17 18 | mc6000617 | 24 |
| mc13000603 | 9 21 | mc6000623 | 1 21 |
| mc13000631 | 1 21 | mc6000630 | 4 7 21 |
| mc13000669 | 8 | mc6000656 | 9 |
| mc13000680 | 7 | mc6000675 | 4 7 21 |
| mc13000704 | 7 9 21 | mc6000703 | 5 6 7 21 |
| mc13000750 | 7 21 | mc6000731 | 6 7 22 24 |
| mc13000762 | 7 15 21 | mc6000759 | 7 10 13 15 21 24 |
| mc13000766 | 1 7 21 | mc6000780 | 4 7 21 |
| mc13000768 | 16 19 20 | mc6000818 | 10 21 |
| mc13000769 | 17 21 23 24 | mc6000843 | 7 21 |
| mc13000771 | 7 12 17 21 | mc6000853 | 1 7 9 11 12 13 16 21 |
| mc13000784 | 5 7 21 | mc6000855 | 12 13 21 |
| mc13000791 | 2 9 | mc6000872 | 4 7 16 18 19 20 21 22 23 24 |
| mc13000846 | 7 9 17 | mc6001029 | 13 21 |
| mc13000853 | 7 10 | mc6001034 | 4 7 16 21 |
| mc13000859 | 7 | mc6001039 | 21 |
| mc13000860 | 10 12 21 | mc6001053 | 1 21 |
| mc13000863 | 5 7 9 15 21 | mc6001062 | 10 13 18 21 |
| mc13000869 | 2 21 | mc6001065 | 13 15 21 |
| mc13000876 | 6 7 9 13 15 17 18 21 22 | mc6001069 | 9 21 |
| mc13000904 | 2 9 | mc6001094 | 1 7 12 21 |
| mc13000917 | 12 13 15 | mc6001110 | 7 13 21 |
| mc13000919 | 9 21 | mc6001159 | 5 21 |
| mc13000928 | 9 10 12 21 | mc6001163 | 10 |
| mc13000940 | 18 | mc6001167 | 9 12 21 |
| mc13000941 | 4 7 21 | mc6001204 | 7 9 10 |
| mc13000944 | 6 21 | mc6001210 | 7 16 21 |
| mc13001001 | 6 | mc6001212 | 7 9 21 23 |
| mc13001040 | 3 4 6 7 21 | mc6001213 | 7 16 21 |
| mc13001046 | 9 | mc6001226 | 6 21 |
| mc13001090 | 7 21 | mc6001232 | 1 6 7 8 10 21 |
| mc13001099 | 5 6 7 21 | mc6001242 | 21 |
| mc13001188 | 2 7 9 21 | mc6001244 | 6 7 21 |
| mc13001204 | 4 7 | mc6001278 | 7 |
| mc13001216 | 1 2 7 9 21 | mc6001286 | 7 9 17 21 |
| mc13001228 | 6 | mc6001291 | 7 9 10 21 |
| mc13001242 | 20 | mc6001305 | 6 9 21 |
| mc13001258 | 4 7 | mc6001309 | 1 |
| mc13001291 | 17 | mc6001312 | 1 6 |
| mc13001349 | 6 7 9 21 | mc6001352 | 7 21 |
| mc13001384 | 6 7 12 21 | mc6001387 | 6 7 12 21 |
| mc13001390 | 1 21 | mc6001396 | 4 7 16 21 |
| mc13001399 | 1 | mc6001397 | 5 21 |
| mc13001400 | 9 21 | mc6001402 | 7 16 |
| mc13001446 | 1 7 21 | mc6001450 | 1 2 7 21 |
| mc13001453 | 1 6 21 24 | mc6001470 | 21 |
| mc13001480 | 4 7 12 16 21 | mc6001472 | 4 21 |
| mc13001484 | 9 21 | mc6001475 | 7 15 16 17 18 19 20 |
| mc13001492 | 3 | mc6001523 | 4 7 21 |
| mc13001515 | 10 13 21 23 24 | mc6001532 | 3 6 21 |
| mc13001518 | 1 7 21 | mc6001559 | 2 12 |
| mc13001519 | 4 7 21 24 | mc6001566 | 6 7 |
| mc13001524 | 7 15 | mc6001582 | 7 10 |
| mc13001535 | 1 4 7 8 21 | mc6001613 | 10 13 21 22 23 24 |
| mc13001540 | 5 21 | mc6001618 | 7 8 |
| mc13001541 | 3 21 | mc6001627 | 1 6 |
| mc13001542 | 4 6 7 8 21 | mc6001634 | 6 7 12 17 21 |
| mc13001545 | 1 3 4 6 7 8 21 | mc6001646 | 21 |
| mc13001548 | 3 7 8 16 21 | mc6001660 | 9 12 21 |
| mc13001591 | 4 7 24 | mc6001672 | 21 |
| mc13001606 | 4 7 21 | mc6001675 | 10 12 21 |
| mc13001607 | 6 7 17 21 | mc6001686 | 7 |
| mc13001667 | 1 21 | mc6001692 | 12 15 |
| mc13001715 | 7 21 | mc6001700 | 1 7 9 13 21 |
| mc13001726 | 4 7 21 | mc6001710 | 10 |
| mc13001730 | 10 15 21 | mc6001720 | 12 13 15 |
| mc13001733 | 4 7 16 18 19 20 21 22 23 24 | mc6001731 | 7 21 |
| mc13001737 | 1 6 10 21 | mc6001764 | 7 8 9 21 |
| mc13001741 | 3 7 21 | mc6001777 | 4 7 |
| mc13001788 | 7 10 12 21 | mc6001781 | 9 21 |
| mc13001790 | 5 21 | mc6001789 | 1 2 7 9 21 |
| mc13001818 | 7 11 15 21 | mc6001792 | 7 9 17 21 |
| mc14000014 | 7 9 | mc6001798 | 7 8 17 21 |
| mc14000018 | 3 21 | mc6001822 | 1 4 7 9 21 |
| mc14000084 | 10 21 | mc6001861 | 6 7 9 21 |
| mc14000089 | 9 12 | mc6001880 | 10 |
| mc14000100 | 1 7 21 | mc6001889 | 3 4 7 21 |
| mc14000128 | 1 7 21 22 23 24 | mc6001895 | 17 21 |
| mc14000139 | 21 | mc6001901 | 7 10 13 21 |
| mc14000147 | 9 12 21 24 | mc6001921 | 7 9 21 |
| mc14000158 | 9 | mc6001934 | 15 21 |
| mc14000160 | 6 | mc6001952 | 1 |
| mc14000162 | 7 21 | mc6001977 | 9 21 |
| mc14000165 | 21 | mc6001979 | 7 10 13 21 |
| mc14000166 | 1 7 | mc6001983 | 7 10 13 21 |
| mc14000183 | 1 | mc6002000 | 7 8 9 10 12 13 15 21 |
| mc14000202 | 7 9 12 21 22 | mc6002007 | 7 13 21 22 |
| mc14000218 | 4 7 21 | mc6002012 | 7 10 |
| mc14000246 | 4 7 21 | mc6002024 | 10 21 |
| mc14000247 | 7 21 | mc6002036 | 4 7 21 |
| mc14000325 | 7 9 21 | mc6002078 | 21 |
| mc14000344 | 21 | mc6002080 | 1 7 10 21 |
| mc14000351 | 7 21 | mc6002081 | 4 10 21 |
| mc14000352 | 4 21 | mc6002090 | 12 |
| mc14000354 | 4 7 | mc6002094 | 18 |
| mc14000360 | 15 | mc6002110 | 6 7 |
| mc14000379 | 1 21 | mc6002114 | 21 |
| mc14000384 | 4 7 21 | mc6002181 | 9 21 24 |
| mc14000385 | 6 7 9 21 | mc6002186 | 9 21 |
| mc14000387 | 1 2 7 21 | mc6002189 | 21 |
| mc14000391 | 9 21 | mc6002197 | 7 9 12 21 |
| mc14000433 | 9 21 24 | mc6002259 | 7 9 10 |
| mc14000467 | 21 | mc6002265 | 7 8 |
| mc14000513 | 2 9 11 | mc6002273 | 7 8 10 |
| mc14000548 | 3 6 8 | mc6002327 | 10 12 13 |
| mc14000559 | 7 21 | mc6002354 | 7 |
| mc14000595 | 6 7 9 19 21 | mc6002362 | 12 13 |
| mc14000597 | 7 9 21 | mc6002366 | 10 |
| mc14000617 | 10 13 | mc6002371 | 10 12 13 |
| mc14000643 | 1 21 | mc6002372 | 10 12 13 |
| mc14000646 | 1 7 | mc6002374 | 10 12 13 |
| mc14000650 | 24 | mc6002376 | 10 |
| mc14000651 | 1 3 7 21 | mc6002377 | 10 |
| mc14000663 | 10 | mc7000005 | 4 7 8 21 |
| mc14000674 | 7 9 12 21 | mc7000006 | 10 |
| mc14000677 | 1 7 13 21 24 | mc7000019 | 9 10 21 |
| mc14000684 | 7 12 13 20 | mc7000036 | 7 13 19 21 23 |
| mc14000729 | 7 21 | mc7000038 | 6 7 8 10 13 21 23 |
| mc14000731 | 5 21 | mc7000040 | 1 6 10 21 24 |
| mc14000740 | 11 | mc7000061 | 7 21 |
| mc14000742 | 3 7 15 21 | mc7000068 | 7 21 |
| mc14000777 | 7 22 | mc7000078 | 7 9 10 21 |
| mc14000779 | 1 2 7 21 | mc7000105 | 4 7 21 |
| mc14000784 | 6 7 8 21 | mc7000124 | 1 3 6 21 24 |
| mc14000793 | 1 7 18 19 | mc7000127 | 4 7 21 |
| mc14000799 | 7 | mc7000145 | 10 21 |
| mc14000831 | 6 15 21 | mc7000157 | 10 21 |
| mc14000838 | 3 7 21 | mc7000171 | 4 7 21 |
| mc14000839 | 3 7 21 | mc7000188 | 4 7 21 |
| mc14000840 | 3 4 7 21 | mc7000189 | 4 7 21 |
| mc14000869 | 5 21 | mc7000204 | 3 7 11 21 |
| mc14000870 | 1 7 | mc7000213 | 1 6 |
| mc14000888 | 3 4 7 21 | mc7000231 | 7 21 |
| mc14000952 | 3 4 7 21 | mc7000237 | 21 24 |
| mc14001036 | 1 3 4 5 6 7 8 10 13 15 17 18 21 22 23 24 | mc7000245 | 3 4 7 |
| mc14001046 | 13 15 19 21 | mc7000273 | 1 7 |
| mc14001060 | 6 7 10 12 13 18 21 23 | mc7000295 | 21 |
| mc14001070 | 9 21 | mc7000320 | 17 21 |
| mc14001071 | 14 | mc7000328 | 4 6 7 8 21 |
| mc14001079 | 4 7 15 21 | mc7000329 | 1 2 7 8 10 12 13 21 |
| mc14001104 | 10 | mc7000336 | 7 12 16 17 21 |
| mc14001171 | 7 11 12 17 21 | mc7000357 | 7 21 |
| mc14001186 | 5 11 12 13 15 21 | mc7000358 | 4 21 |
| mc14001190 | 7 9 | mc7000362 | 1 2 7 8 9 12 13 21 23 24 |
| mc14001191 | 4 7 21 | mc7000373 | 7 |
| mc14001200 | 4 7 21 | mc7000384 | 7 10 17 21 23 |
| mc14001208 | 5 21 | mc7000399 | 4 7 21 |
| mc14001211 | 7 | mc7000406 | 4 6 7 21 |
| mc14001224 | 6 7 8 10 21 | mc7000408 | 7 |
| mc14001233 | 21 | mc7000415 | 7 21 |
| mc14001236 | 21 | mc7000430 | 6 |
| mc14001276 | 12 20 21 24 | mc7000443 | 4 7 15 21 |
| mc14001299 | 7 | mc7000444 | 7 21 |
| mc14001412 | 21 | mc7000445 | 21 |
| mc14001422 | 7 10 12 13 16 18 20 21 | mc7000463 | 1 |
| mc14001430 | 4 21 22 24 | mc7000470 | 7 21 |
| mc14001434 | 7 21 | mc7000491 | 11 |
| mc14001444 | 3 4 7 21 | mc7000492 | 7 |
| mc14001462 | 7 | mc7000497 | 1 7 9 21 |
| mc14001489 | 6 21 | mc7000520 | 7 21 |
| mc14001535 | 4 7 21 | mc7000526 | 6 7 8 10 15 21 23 24 |
| mc14001543 | 7 17 | mc7000554 | 7 15 |
| mc14001547 | 7 21 | mc7000560 | 21 |
| mc14001581 | 7 | mc7000587 | 6 7 21 |
| mc14001632 | 7 9 21 | mc7000590 | 9 21 |
| mc14001637 | 7 10 15 | mc7000610 | 4 7 21 |
| mc14001658 | 10 13 | mc7000614 | 4 7 21 |
| mc14001659 | 1 3 | mc7000625 | 4 7 21 |
| mc15000015 | 1 21 | mc7000629 | 7 17 21 24 |
| mc15000025 | 7 8 15 | mc7000639 | 6 9 |
| mc15000027 | 10 21 | mc7000651 | 6 7 9 13 17 21 24 |
| mc15000087 | 21 | mc7000654 | 7 |
| mc15000098 | 1 | mc7000665 | 7 8 10 12 21 |
| mc15000100 | 7 | mc7000669 | 10 11 12 13 21 |
| mc15000144 | 7 21 | mc7000674 | 9 21 |
| mc15000147 | 3 7 10 | mc7000676 | 9 21 |
| mc15000187 | 1 21 | mc7000704 | 21 |
| mc15000228 | 6 | mc7000733 | 1 7 8 9 12 13 21 |
| mc15000231 | 6 | mc7000737 | 7 |
| mc15000278 | 7 | mc7000781 | 6 7 9 21 |
| mc15000282 | 3 6 7 8 10 17 18 | mc7000844 | 8 10 |
| mc15000284 | 7 | mc7000852 | 4 7 21 |
| mc15000298 | 10 12 13 21 24 | mc7000862 | 10 |
| mc15000305 | 6 | mc7000866 | 4 7 21 |
| mc15000309 | 7 17 21 | mc7000889 | 6 |
| mc15000324 | 1 4 7 21 | mc7000907 | 13 22 |
| mc15000330 | 9 12 21 23 24 | mc7000916 | 7 12 13 17 19 21 23 |
| mc15000358 | 10 | mc7000949 | 7 21 |
| mc15000366 | 10 21 | mc7000965 | 5 6 7 21 |
| mc15000382 | 6 8 | mc7000979 | 1 21 |
| mc15000396 | 21 | mc7000986 | 3 7 11 15 |
| mc15000408 | 7 10 11 12 13 21 22 | mc7001004 | 1 7 8 |
| mc15000454 | 4 21 | mc7001005 | 5 6 7 8 21 24 |
| mc15000500 | 9 12 21 22 | mc7001026 | 7 13 17 18 |
| mc15000503 | 4 21 | mc7001042 | 6 7 9 17 18 |
| mc15000526 | 7 15 21 | mc7001075 | 10 |
| mc15000535 | 7 10 | mc7001089 | 4 7 20 21 |
| mc15000566 | 4 7 21 | mc7001105 | 1 2 7 9 21 |
| mc15000573 | 1 7 | mc7001117 | 21 |
| mc15000578 | 9 | mc7001157 | 10 |
| mc15000579 | 7 15 21 | mc7001174 | 6 7 21 |
| mc15000585 | 9 21 24 | mc7001215 | 6 7 21 |
| mc15000621 | 15 21 | mc7001226 | 9 21 |
| mc15000623 | 13 21 | mc7001232 | 1 21 |
| mc15000669 | 7 12 21 | mc7001252 | 15 |
| mc15000686 | 10 | mc7001260 | 21 |
| mc15000714 | 7 | mc7001270 | 10 |
| mc15000737 | 12 17 21 | mc7001281 | 17 |
| mc15000764 | 10 | mc7001373 | 21 |
| mc15000801 | 1 7 9 21 | mc7001424 | 1 7 10 18 21 |
| mc15000803 | 21 | mc7001456 | 4 7 10 16 21 |
| mc15000808 | 11 21 | mc7001463 | 7 21 |
| mc15000823 | 3 7 21 | mc7001476 | 1 11 |
| mc15000833 | 21 | mc7001479 | 6 7 21 |
| mc15000852 | 3 4 7 | mc7001492 | 4 7 21 |
| mc15000876 | 4 7 21 | mc7001494 | 3 9 21 |
| mc15000886 | 7 21 | mc7001499 | 6 9 21 |
| mc15000894 | 6 | mc7001500 | 6 9 21 |
| mc15000896 | 6 | mc7001513 | 21 |
| mc15000905 | 10 | mc7001525 | 6 7 |
| mc15000910 | 7 21 | mc7001543 | 21 |
| mc15000927 | 7 9 17 21 | mc7001601 | 10 12 21 |
| mc15000936 | 7 10 | mc7001690 | 6 |
| mc15000940 | 10 13 15 18 21 | mc7001693 | 2 7 9 21 |
| mc15000978 | 3 7 17 21 | mc7001695 | 1 4 21 |
| mc15000986 | 4 7 21 | mc7001713 | 1 6 7 21 |
| mc15000987 | 4 7 16 21 22 23 24 | mc7001718 | 21 |
| mc15000992 | 1 10 | mc7001727 | 7 10 15 16 21 |
| mc15001003 | 3 4 7 21 | mc7001741 | 7 10 12 13 |
| mc15001010 | 5 7 | mc7001742 | 9 |
| mc15001014 | 7 9 18 21 22 23 | mc7001745 | 10 13 |
| mc15001024 | 9 17 21 22 | mc7001771 | 5 |
| mc15001033 | 7 9 21 | mc7001774 | 7 9 10 21 |
| mc15001058 | 3 4 6 7 21 | mc7001779 | 7 8 10 21 24 |
| mc15001061 | 12 21 | mc7001789 | 7 8 10 21 |
| mc15001064 | 7 | mc7001791 | 1 7 21 |
| mc15001065 | 4 7 12 21 | mc7001792 | 1 6 21 |
| mc15001072 | 1 2 4 7 21 | mc7001799 | 6 7 9 10 21 |
| mc15001084 | 1 4 7 21 | mc7001802 | 1 |
| mc15001087 | 21 | mc7001807 | 3 7 21 |
| mc15001107 | 1 2 7 9 | mc7001809 | 10 21 |
| mc15001118 | 10 13 18 21 | mc7001810 | 7 |
| mc15001121 | 7 | mc7001815 | 7 9 21 |
| mc15001128 | 7 9 10 21 | mc7001816 | 10 |
| mc15001133 | 1 21 | mc7001822 | 10 13 |
| mc15001160 | 8 12 17 | mc7001837 | 10 13 |
| mc15001161 | 8 | mc7001838 | 7 21 |
| mc15001211 | 21 | mc7001893 | 10 13 |
| mc15001230 | 6 | mc7001914 | 18 22 |
| mc15001233 | 17 21 | mc7001940 | 21 |
| mc15001236 | 17 21 | mc7001948 | 6 7 21 |
| mc15001283 | 9 | mc7001951 | 7 |
| mc15001284 | 9 | mc7001962 | 10 13 |
| mc15001288 | 9 | mc7001974 | 6 21 |
| mc15001327 | 19 21 | mc7002003 | 10 13 |
| mc15001358 | 21 | mc7002023 | 6 9 21 |
| mc15001376 | 4 | mc7002050 | 10 13 |
| mc15001397 | 1 | mc7002078 | 6 9 |
| mc15001460 | 16 21 24 | mc7002113 | 10 13 |
| mc15001491 | 7 15 | mc7002141 | 7 21 |
| mc15001528 | 7 17 | mc7002145 | 7 |
| mc15001530 | 21 | mc7002201 | 6 7 21 |
| mc15001532 | 7 17 20 | mc7002207 | 4 7 8 9 13 21 |
| mc15001555 | 7 17 | mc7002215 | 1 21 |
| mc15001568 | 1 17 21 | mc7002270 | 5 |
| mc15001572 | 7 21 | mc7002283 | 5 7 21 |
| mc15001592 | 2 7 9 21 | mc7002318 | 1 21 |
| mc15001609 | 4 7 15 16 18 19 21 | mc7002320 | 1 7 |
| mc15001617 | 3 4 7 17 21 | mc7002333 | 7 |
| mc15001640 | 21 | mc7002350 | 13 |
| mc15001642 | 4 7 15 16 21 | mc7002369 | 6 9 21 |
| mc15001651 | 7 21 | mc7002370 | 6 7 9 21 |
| mc15001664 | 6 7 11 21 | mc7002379 | 15 |
| mc16000018 | 4 7 21 | mc7002385 | 7 |
| mc16000042 | 4 7 | mc7002396 | 1 6 |
| mc16000045 | 7 | mc7002398 | 4 7 21 |
| mc16000050 | 4 7 21 | mc7002420 | 2 7 9 10 |
| mc16000052 | 9 | mc7002444 | 7 10 12 |
| mc16000095 | 1 21 | mc7002448 | 5 7 21 |
| mc16000102 | 6 15 21 | mc7002451 | 16 17 |
| mc16000124 | 7 8 21 23 24 | mc7002477 | 6 10 17 21 |
| mc16000137 | 3 7 15 16 17 21 | mc7002481 | 10 |
| mc16000145 | 2 9 21 | mc7002483 | 1 7 21 |
| mc16000178 | 4 7 15 16 19 21 | mc7002486 | 4 7 21 |
| mc16000186 | 4 21 | mc7002497 | 7 21 |
| mc16000204 | 1 7 9 21 | mc7002502 | 7 10 11 12 21 |
| mc16000205 | 4 7 | mc7002513 | 4 7 21 |
| mc16000207 | 7 | mc7002525 | 7 21 |
| mc16000220 | 4 7 21 | mc7002537 | 7 21 |
| mc16000224 | 3 10 15 18 24 | mc7002574 | 1 7 9 17 21 |
| mc16000230 | 7 8 10 15 21 | mc7002592 | 1 6 7 10 15 16 18 19 20 21 |
| mc16000239 | 7 18 21 22 | mc7002600 | 7 21 |
| mc16000241 | 3 21 | mc7002603 | 7 21 |
| mc16000276 | 7 | mc7002615 | 4 7 21 |
| mc16000280 | 1 2 7 | mc7002624 | 6 7 12 21 |
| mc16000281 | 7 10 12 | mc7002648 | 1 2 16 |
| mc16000352 | 9 | mc7002662 | 5 6 21 |
| mc16000367 | 1 21 | mc7002667 | 6 7 8 10 15 21 |
| mc16000372 | 5 7 21 | mc7002672 | 4 7 21 |
| mc16000374 | 9 | mc7002721 | 3 16 |
| mc16000380 | 5 7 21 | mc7002723 | 7 15 21 |
| mc16000401 | 1 2 21 | mc7002775 | 10 |
| mc16000411 | 7 | mc7002799 | 4 7 21 |
| mc16000429 | 21 | mc7002800 | 21 |
| mc16000478 | 7 9 17 21 | mc7002801 | 7 24 |
| mc16000481 | 10 15 | mc7002803 | 6 9 21 |
| mc16000483 | 10 21 | mc7002849 | 4 7 21 |
| mc16000528 | 11 12 21 23 | mc7002871 | 2 6 9 11 12 |
| mc16000550 | 1 21 | mc7002881 | 3 10 15 |
| mc16000565 | 21 | mc7002883 | 7 9 16 21 24 |
| mc16000597 | 6 7 12 | mc7002922 | 10 |
| mc16000600 | 1 7 9 21 | mc7002933 | 7 21 22 |
| mc16000604 | 9 21 | mc7002934 | 7 17 21 |
| mc16000606 | 7 | mc7002935 | 7 10 12 21 |
| mc16000610 | 1 6 7 | mc7002952 | 9 12 21 |
| mc16000624 | 1 7 10 21 | mc7002958 | 10 |
| mc16000652 | 8 | mc7002968 | 4 7 21 |
| mc16000678 | 4 7 | mc7002970 | 7 |
| mc16000703 | 21 | mc7002974 | 7 21 |
| mc16000753 | 7 21 | mc7002993 | 7 21 23 |
| mc16000794 | 21 | mc7002995 | 4 7 10 12 16 20 21 |
| mc16000812 | 7 10 12 21 | mc7002996 | 6 |
| mc16000861 | 6 9 | mc7003004 | 6 |
| mc16000869 | 10 13 | mc7003008 | 6 7 21 |
| mc16000903 | 6 | mc7003026 | 1 21 |
| mc16000937 | 1 6 21 | mc7003031 | 10 21 |
| mc16000941 | 7 | mc7003040 | 10 |
| mc16000943 | 10 | mc8000004 | 8 |
| mc16000972 | 7 | mc8000008 | 7 21 |
| mc16001007 | 7 21 | mc8000028 | 7 10 |
| mc16001008 | 4 | mc8000149 | 4 7 |
| mc16001015 | 4 7 21 | mc8000150 | 21 |
| mc16001078 | 4 7 20 21 | mc8000169 | 3 4 7 21 |
| mc16001079 | 5 7 21 | mc8000173 | 21 |
| mc16001104 | 1 6 10 | mc8000191 | 6 |
| mc16001155 | 6 | mc8000205 | 3 6 7 21 |
| mc16001182 | 21 | mc8000210 | 7 21 |
| mc16001216 | 6 7 11 21 23 | mc8000280 | 11 12 13 21 |
| mc16001246 | 6 7 9 10 12 22 24 | mc8000285 | 11 12 13 21 |
| mc16001268 | 7 21 | mc8000288 | 11 12 13 21 |
| mc16001277 | 9 12 17 21 | mc8000297 | 11 12 13 21 |
| mc16001313 | 1 11 21 | mc8000305 | 11 13 |
| mc16001364 | 7 | mc8000308 | 11 12 13 21 |
| mc16001390 | 10 | mc8000309 | 11 12 13 21 |
| mc17000005 | 7 9 12 | mc8000311 | 11 12 13 21 |
| mc17000016 | 1 7 | mc8000312 | 11 12 13 21 |
| mc17000023 | 1 2 | mc8000317 | 1 6 7 |
| mc17000026 | 4 6 7 17 21 | mc8000319 | 21 |
| mc17000048 | 6 7 8 9 12 17 21 | mc8000325 | 1 6 9 21 |
| mc17000058 | 4 21 | mc8000342 | 4 7 17 21 |
| mc17000067 | 7 8 10 21 | mc8000345 | 4 7 |
| mc17000076 | 9 21 | mc8000355 | 4 7 21 |
| mc17000078 | 7 10 15 | mc8000378 | 3 7 21 |
| mc17000163 | 3 10 15 21 | mc8000380 | 5 21 |
| mc17000167 | 6 7 11 21 | mc8000386 | 7 8 15 21 |
| mc17000174 | 7 21 | mc8000393 | 4 7 21 |
| mc17000175 | 7 21 | mc8000396 | 9 21 |
| mc17000176 | 12 17 | mc8000399 | 1 7 |
| mc17000190 | 4 7 21 | mc8000422 | 9 10 12 |
| mc17000191 | 7 9 21 | mc8000459 | 7 |
| mc17000222 | 7 21 | mc8000464 | 1 21 |
| mc17000227 | 1 6 7 | mc8000470 | 8 10 15 18 20 21 24 |
| mc17000237 | 10 | mc8000471 | 8 10 15 18 20 21 24 |
| mc17000283 | 10 21 | mc8000493 | 3 4 7 8 21 |
| mc17000292 | 10 21 | mc8000533 | 1 3 7 |
| mc17000314 | 7 21 | mc8000534 | 3 |
| mc17000317 | 7 21 | mc8000544 | 1 6 7 10 |
| mc17000323 | 4 7 21 | mc8000565 | 4 7 15 21 |
| mc17000325 | 6 7 11 21 24 | mc8000580 | 7 10 13 15 16 21 22 24 |
| mc17000338 | 10 | mc8000598 | 4 |
| mc17000373 | 1 3 21 | mc8000602 | 6 21 |
| mc17000378 | 11 | mc8000616 | 7 |
| mc17000387 | 7 10 21 | mc8000625 | 6 7 10 16 21 24 |
| mc17000392 | 15 20 21 23 24 | mc8000643 | 1 2 7 9 21 |
| mc17000397 | 2 7 9 21 | mc8000644 | 7 |
| mc17000426 | 7 21 | mc8000661 | 7 9 21 23 |
| mc17000429 | 7 10 12 20 21 | mc8000732 | 6 7 17 21 |
| mc17000430 | 6 7 | mc8000787 | 1 21 |
| mc17000440 | 8 10 21 22 23 24 | mc8000804 | 9 |
| mc17000447 | 7 10 13 21 | mc8000810 | 7 10 |
| mc17000467 | 8 21 | mc8000849 | 7 10 |
| mc17000468 | 7 9 10 | mc8000856 | 7 21 |
| mc17000473 | 16 18 22 24 | mc8000871 | 5 7 |
| mc17000481 | 7 12 21 | mc8000877 | 8 |
| mc17000484 | 7 8 10 16 21 | mc8000905 | 10 |
| mc17000485 | 6 21 24 | mc8000909 | 2 7 9 15 21 |
| mc17000487 | 7 8 10 22 23 24 | mc8000918 | 7 12 21 |
| mc17000489 | 7 16 21 | mc8000920 | 4 7 10 21 |
| mc17000508 | 2 7 9 17 21 | mc8000931 | 10 15 21 |
| mc17000509 | 4 7 21 | mc8000937 | 21 |
| mc17000511 | 7 8 9 10 12 | mc8000958 | 1 |
| mc17000516 | 9 10 21 | mc8000978 | 9 21 |
| mc17000542 | 7 | mc8000990 | 3 7 10 21 |
| mc17000558 | 3 7 11 21 | mc8001004 | 10 16 20 21 |
| mc17000568 | 7 | mc8001022 | 7 8 |
| mc17000574 | 4 7 21 | mc8001029 | 6 7 11 21 |
| mc17000580 | 1 6 7 21 | mc8001041 | 7 21 |
| mc17000601 | 6 7 21 | mc8001083 | 4 10 13 21 |
| mc17000602 | 1 3 7 | mc8001092 | 7 8 17 |
| mc17000605 | 7 12 21 | mc8001119 | 4 7 21 |
| mc17000612 | 7 12 | mc8001137 | 3 6 7 21 |
| mc17000618 | 7 10 21 | mc8001151 | 7 12 21 |
| mc17000627 | 9 21 | mc8001158 | 6 |
| mc17000630 | 2 7 9 21 | mc8001171 | 1 7 8 10 21 24 |
| mc17000633 | 1 | mc8001178 | 6 |
| mc17000646 | 4 7 8 11 24 | mc8001182 | 7 |
| mc17000651 | 7 21 | mc8001186 | 21 |
| mc17000654 | 7 21 | mc8001201 | 1 6 7 10 11 21 |
| mc17000665 | 4 7 21 | mc8001208 | 10 12 21 |
| mc17000681 | 6 7 17 21 | mc8001226 | 7 |
| mc17000683 | 7 21 | mc8001252 | 13 24 |
| mc17000685 | 6 7 21 | mc8001253 | 1 3 7 |
| mc17000687 | 7 9 10 | mc8001258 | 7 9 |
| mc17000690 | 5 7 21 | mc8001313 | 7 |
| mc17000696 | 4 7 10 15 21 | mc8001316 | 1 7 10 21 |
| mc17000704 | 5 7 21 | mc8001363 | 6 15 |
| mc17000724 | 6 10 13 | mc8001396 | 6 7 8 10 12 13 |
| mc17000744 | 8 10 | mc8001409 | 1 11 21 |
| mc17000750 | 7 | mc8001442 | 7 10 |
| mc17000751 | 6 21 | mc8001443 | 7 10 |
| mc17000766 | 3 4 7 | mc8001450 | 1 15 19 |
| mc17000767 | 4 7 21 | mc8001460 | 10 21 |
| mc17000772 | 1 3 7 21 | mc8001463 | 3 7 17 21 |
| mc17000777 | 6 7 11 | mc8001466 | 9 10 12 21 |
| mc17000780 | 14 | mc8001470 | 4 7 15 21 |
| mc17000790 | 18 21 22 | mc8001474 | 4 |
| mc17000791 | 13 21 | mc8001488 | 4 6 7 8 17 21 |
| mc17000802 | 11 13 21 | mc8001560 | 12 13 14 15 21 |
| mc17000823 | 19 23 | mc8001568 | 2 7 12 17 21 |
| mc17000824 | 21 | mc8001575 | 3 |
| mc17000839 | 12 16 21 | mc8001592 | 1 11 21 |
| mc17000845 | 1 | mc8001597 | 1 |
| mc17000854 | 10 11 13 | mc8001598 | 7 10 15 |
| mc17000857 | 11 | mc8001610 | 8 |
| mc17000887 | 7 | mc8001611 | 9 |
| mc17000889 | 13 24 | mc8001614 | 7 |
| mc17000945 | 10 13 | mc8001615 | 1 6 7 |
| mc17000978 | 1 7 21 | mc8001621 | 12 15 18 22 |
| mc17000991 | 6 10 12 13 | mc8001624 | 2 3 4 7 9 21 |
| mc17000993 | 7 10 21 | mc8001626 | 16 17 20 21 |
| mc17001000 | 7 10 21 | mc8001638 | 1 6 7 21 |
| mc17001007 | 7 10 | mc8001650 | 7 |
| mc17001016 | 9 12 21 | mc8001660 | 5 21 |
| mc17001042 | 4 5 7 21 | mc8001662 | 21 |
| mc17001049 | 7 | mc8001669 | 6 21 |
| mc17001068 | 7 17 21 | mc8001672 | 9 21 |
| mc17001069 | 7 21 | mc8001676 | 4 7 13 15 21 |
| mc17001072 | 7 9 | mc8001710 | 21 |
| mc17001074 | 21 | mc8001743 | 8 11 21 |
| mc17001075 | 7 21 | mc8001749 | 1 7 |
| mc17001080 | 6 8 21 | mc8001752 | 1 6 21 |
| mc17001145 | 8 13 21 22 | mc8001760 | 7 10 |
| mc17001152 | 10 | mc8001767 | 2 7 9 |
| mc17001167 | 3 6 7 10 12 15 16 17 21 | mc8001768 | 7 |
| mc17001169 | 2 7 9 17 21 | mc8001799 | 6 7 |
| mc17001186 | 8 | mc8001805 | 1 11 21 |
| mc17001203 | 7 9 19 21 | mc8001810 | 7 17 19 21 23 |
| mc17001238 | 4 7 21 | mc8001827 | 4 7 21 |
| mc17001245 | 24 | mc8001851 | 17 21 |
| mc17001273 | 3 4 7 17 21 | mc8001857 | 1 7 |
| mc17001277 | 6 7 21 | mc8001871 | 7 |
| mc17001279 | 6 8 9 21 | mc8001886 | 6 |
| mc17001281 | 2 7 9 21 | mc8001927 | 1 4 7 10 15 16 21 |
| mc17001299 | 4 7 21 | mc8001942 | 7 |
| mc17001300 | 1 7 21 | mc8001990 | 6 7 21 |
| mc17001312 | 10 | mc8001998 | 1 6 7 21 |
| mc17001328 | 7 10 12 16 18 21 24 | mc8002007 | 3 14 |
| mc17001361 | 15 16 21 | mc8002037 | 1 9 13 |
| mc17001375 | 6 | mc8002052 | 7 |
| mc17001416 | 7 10 15 16 18 19 20 21 | mc8002058 | 10 |
| mc17001419 | 2 7 9 21 | mc8002063 | 21 |
| mc17001428 | 5 6 7 21 | mc8002066 | 21 |
| mc17001503 | 7 | mc8002068 | 21 |
| mc17001510 | 2 7 9 21 | mc8002074 | 8 |
| mc17001559 | 21 | mc8002078 | 1 7 21 |
| mc17001564 | 4 7 21 | mc8002085 | 6 7 9 21 |
| mc17001572 | 7 10 21 | mc8002087 | 4 7 17 21 |
| mc17001591 | 16 21 | mc8002095 | 5 21 |
| mc17001598 | 13 24 | mc8002118 | 11 12 13 21 |
| mc17001604 | 7 | mc8002124 | 7 21 |
| mc17001610 | 1 | mc8002132 | 7 |
| mc17001612 | 6 | mc9000009 | 10 21 |
| mc17001626 | 7 | mc9000013 | 7 |
| mc17001642 | 6 7 8 10 | mc9000037 | 12 17 21 |
| mc17001678 | 4 7 21 | mc9000038 | 12 17 21 |
| mc17001679 | 1 2 21 | mc9000048 | 1 6 12 15 16 17 21 |
| mc17001748 | 1 7 9 21 | mc9000055 | 10 15 21 |
| mc17001804 | 6 7 21 | mc9000065 | 4 7 10 13 21 |
| mc17001836 | 7 21 | mc9000114 | 7 12 21 |
| mc17001840 | 7 21 | mc9000142 | 9 |
| mc17001841 | 7 10 11 13 21 | mc9000167 | 6 7 |
| mc17001857 | 1 | mc9000168 | 6 7 |
| mc18000026 | 7 | mc9000169 | 6 7 |
| mc18000033 | 7 17 21 | mc9000195 | 4 7 21 |
| mc18000037 | 7 21 | mc9000214 | 4 7 21 |
| mc18000049 | 3 4 15 21 | mc9000228 | 1 13 21 22 23 24 |
| mc18000056 | 4 7 9 10 | mc9000247 | 1 10 |
| mc18000085 | 3 | mc9000250 | 6 9 |
| mc18000127 | 3 4 21 | mc9000254 | 9 |
| mc18000130 | 6 | mc9000257 | 1 6 10 |
| mc18000142 | 7 8 10 21 | mc9000273 | 7 9 10 |
| mc18000145 | 1 9 | mc9000276 | 21 |
| mc18000166 | 7 21 | mc9000341 | 7 |
| mc18000240 | 1 7 21 | mc9000348 | 1 |
| mc18000267 | 7 21 | mc9000353 | 1 7 21 |
| mc18000342 | 1 | mc9000361 | 13 21 |
| mc18000344 | 4 7 21 | mc9000380 | 12 21 |
| mc18000388 | 8 | mc9000416 | 6 21 |
| mc18000398 | 21 | mc9000427 | 21 |
| mc18000417 | 21 | mc9000443 | 9 12 13 21 24 |
| mc18000434 | 7 15 21 | mc9000488 | 21 |
| mc18000445 | 6 7 8 11 13 21 | mc9000501 | 11 15 21 |
| mc18000459 | 7 21 | mc9000508 | 21 |
| mc18000480 | 7 | mc9000515 | 19 20 23 24 |
| mc18000495 | 12 19 21 23 | mc9000609 | 10 12 |
| mc18000523 | 7 12 | mc9000678 | 7 21 |
| mc18000526 | 9 21 | mc9000689 | 7 8 10 |
| mc18000527 | 4 7 10 21 | mc9000711 | 6 |
| mc18000547 | 7 | mc9000714 | 24 |
| mc18000573 | 12 19 21 23 | mc9000715 | 10 16 21 |
| mc18000615 | 7 | mc9000716 | 7 21 |
| mc18000627 | 4 7 12 21 | mc9000729 | 9 21 |
| mc18000637 | 6 7 10 21 | mc9000747 | 4 7 21 |
| mc18000697 | 5 7 21 | mc9000761 | 6 |
| mc18000702 | 6 8 13 21 | mc9000764 | 6 7 10 18 21 |
| mc18000810 | 9 21 | mc9000779 | 7 12 16 18 19 21 |
| mc18000817 | 9 10 | mc9000786 | 7 21 |
| mc18000888 | 7 9 21 | mc9000802 | 7 |
| mc18000894 | 1 24 | mc9000834 | 21 |
| mc18000919 | 17 21 | mc9000848 | 4 7 |
| mc18000931 | 6 7 10 13 18 22 24 | mc9000854 | 12 17 21 |
| mc18000951 | 6 7 9 21 | mc9000902 | 3 6 7 10 15 21 |
| mc18000955 | 7 | mc9000906 | 3 |
| mc18000968 | 6 | mc9000934 | 4 7 |
| mc18000975 | 6 7 9 21 | mc9000942 | 10 21 |
| mc18000977 | 21 | mc9000944 | 9 10 21 |
| mc18000988 | 15 | mc9000957 | 6 9 12 21 |
| mc18001012 | 6 | mc9000979 | 15 |
| mc18001018 | 7 21 | mc9000980 | 9 |
| mc18001019 | 8 | mc9000994 | 1 |
| mc18001039 | 3 21 24 | mc9001006 | 12 21 |
| mc18001045 | 6 7 21 | mc9001027 | 5 21 |
| mc18001047 | 9 21 | mc9001034 | 7 9 12 21 |
| mc18001055 | 6 9 17 21 | mc9001035 | 7 21 |
| mc18001110 | 7 9 24 | mc9001057 | 4 7 8 15 21 |
| mc18001163 | 1 2 7 9 10 13 18 19 21 22 23 24 | mc9001066 | 7 13 15 16 18 21 |
| mc18001176 | 5 7 21 | mc9001072 | 7 9 12 13 21 24 |
| mc18001183 | 7 | mc9001076 | 5 21 |
| mc18001191 | 7 21 | mc9001084 | 7 |
| mc18001209 | 1 | mc9001091 | 7 12 13 17 21 |
| mc18001230 | 7 9 | mc9001137 | 21 |
| mc18001241 | 7 | mc9001154 | 8 17 21 |
| mc18001265 | 4 21 | mc9001163 | 6 |
| mc18001266 | 2 3 4 7 9 21 | mc9001169 | 7 |
| mc18001268 | 7 9 12 21 | mc9001176 | 9 12 13 21 22 24 |
| mc18001273 | 4 21 | mc9001177 | 10 12 |
| mc18001279 | 3 4 7 9 19 20 21 | mc9001189 | 3 21 |
| mc18001309 | 3 21 | mc9001195 | 3 4 |
| mc18001353 | 7 | mc9001196 | 7 |
| mc18001357 | 6 9 21 | mc9001198 | 7 21 |
| mc18001370 | 12 16 18 19 | mc9001215 | 6 7 8 10 21 |
| mc19000009 | 10 | mc9001231 | 4 7 |
| mc19000063 | 7 21 | mc9001237 | 6 9 |
| mc19000068 | 6 21 | mc9001263 | 4 7 10 15 21 |
| mc19000071 | 6 7 21 | mc9001292 | 2 7 9 |
| mc19000075 | 7 | mc9001327 | 6 7 8 20 21 |
| mc19000084 | 21 | mc9001333 | 7 21 |
| mc19000125 | 1 6 21 | mc9001336 | 1 21 |
| mc19000137 | 3 | mc9001370 | 17 21 |
| mc19000146 | 7 10 | mc9001372 | 7 8 10 21 |
| mc19000148 | 6 21 | mc9001375 | 1 3 6 7 10 21 |
| mc19000154 | 7 10 21 | mc9001402 | 7 17 21 |
| mc19000180 | 10 | mc9001413 | 1 6 7 10 16 |
| mc19000182 | 1 6 7 | mc9001426 | 7 21 |
| mc19000191 | 9 21 | mc9001429 | 5 21 |
| mc19000195 | 1 2 11 | mc9001457 | 6 |
| mc19000209 | 21 | mc9001481 | 7 21 |
| mc19000219 | 5 21 | mc9001490 | 21 |
| mc19000230 | 3 6 10 15 21 | mc9001551 | 4 7 9 21 |
| mc19000233 | 21 | mc9001560 | 16 |
| mc19000244 | 21 | mc9001570 | 6 7 12 16 21 |
| mc19000257 | 10 21 | mc9001613 | 9 12 |
| mc19000273 | 7 9 13 21 24 | mc9001625 | 10 |
| mc19000274 | 7 | mc9001632 | 1 3 6 7 8 11 |
| mc19000280 | 1 | mc9001635 | 3 4 7 21 |
| mc19000287 | 21 | mc9001656 | 9 21 |
| mc19000304 | 6 11 | mc9001663 | 6 7 10 21 |
| mc19000305 | 21 | mc9001701 | 7 |
| mc19000310 | 10 | mc9001709 | 7 21 |
| mc19000314 | 10 | mc9001734 | 1 3 6 7 8 10 |
| mc19000318 | 10 21 | mc9001777 | 3 4 21 |
| mc19000328 | 10 | mc9001779 | 7 13 17 21 |
| mc19000329 | 10 | mc9001781 | 7 13 17 21 |
| mc19000333 | 10 | mc9001791 | 1 24 |
| mc19000348 | 7 9 21 | mc9001796 | 1 21 |
| mc19000365 | 21 | mc9001801 | 1 7 9 21 |
| mc19000370 | 6 | mc9001813 | 7 8 |
| mc19000372 | 11 21 | mc9001823 | 7 |
| mc19000379 | 8 10 15 16 18 20 21 | mc9001824 | 1 6 7 10 21 |
| mc19000408 | 21 | mc9001835 | 7 10 21 |
| mc19000481 | 1 21 | mc9001849 | 4 7 21 |
| mc19000516 | 7 | mc9001869 | 1 3 6 |
| mc19000592 | 4 7 | mc9001884 | 17 21 |
| mc19000596 | 4 7 16 21 | mc9001887 | 6 |
| mc19000612 | 3 4 7 17 21 | mc9001901 | 6 7 12 16 17 19 21 23 |
| mc19000613 | 3 4 7 17 21 | mc9001905 | 1 21 |
| mc19000661 | 7 13 15 21 24 | mc9001918 | 1 7 13 |
| mc19000674 | 9 21 | mc9001922 | 5 |
| mc19000678 | 1 | mc9001930 | 1 10 21 |
| mc19000688 | 10 15 16 21 | mc9001931 | 21 |
| mc19000689 | 1 6 7 8 10 21 | mc9001933 | 9 21 |
| mc19000700 | 4 7 21 | mc9001939 | 15 21 |
| mc19000732 | 1 | mc9001940 | 1 3 7 11 21 |
| mc19000757 | 3 | mc9001945 | 6 |
| mc19000778 | 10 15 21 | mc9001962 | 7 17 21 |
| mc19000781 | 4 6 7 21 | mc9001969 | 1 7 21 |
| mc19000834 | 7 9 21 | mc9001974 | 7 |
| mc19000835 | 7 12 17 21 | mc9001991 | 10 15 16 21 |
| mc19000836 | 1 7 21 | mc9001992 | 7 |
| mc19000841 | 21 | mc9002013 | 8 21 |
| mc19000847 | 3 21 | mc9002039 | 7 17 21 |
| mc19000856 | 9 | mc9002046 | 1 11 21 |
| mc19000864 | 4 21 | mc9002053 | 12 13 15 21 |
| mc19000871 | 10 | mc9002080 | 6 7 10 16 20 21 |
| mc19000872 | 7 21 | mc9002121 | 1 2 21 24 |
| mc19000879 | 1 21 | mc9002126 | 6 10 11 |
| mc19000881 | 1 | mc9002142 | 9 13 21 |
| mc19000891 | 7 12 | mc9002176 | 21 |
| mc19000899 | 6 21 | mc9002180 | 21 |
| mc19000901 | 6 21 | mc9002187 | 21 |
| mc19000908 | 21 | mc9002198 | 1 6 7 10 |
| mc19000927 | 7 9 21 | mc9002212 | 4 7 21 |
| mc19000935 | 1 21 | mc9002225 | 1 6 7 |
| mc19000939 | 21 | mc9002240 | 10 12 13 21 22 24 |
| mc19000951 | 13 15 20 21 | mc9002246 | 3 6 8 21 |
| mc19000987 | 6 7 9 17 21 | mc9002250 | 10 11 12 15 |
| mc19000993 | 8 9 21 | mc9002260 | 7 21 |
| mc19001015 | 7 17 21 23 24 | mc9002264 | 10 13 |
| mc19001025 | 6 7 12 13 16 17 21 | mc9002271 | 7 12 16 21 22 |
| mc19001033 | 9 | mc9002278 | 21 |
| mc19001035 | 11 | mc9002282 | 7 9 17 21 |
| mc19001092 | 6 11 21 | mcm000001 | 2 7 9 21 |
| mc19001095 | 3 7 21 | mcm000002 | 2 7 9 21 |
| mc19001117 | 1 2 7 21 | mcm000003 | 2 7 9 21 |
| mc19001118 | 1 7 | mcm000004 | 2 7 9 21 |
| mc19001190 | 1 2 7 21 | mcm000005 | 2 7 9 21 |
| mc19001192 | 5 6 21 | mcm000006 | 2 7 9 21 |
| mc19001210 | 7 8 9 21 | mcm000007 | 2 7 9 21 |
| mc19001240 | 10 | mcm000008 | 2 7 9 21 |
| mc19001245 | 7 9 17 21 | mcm000009 | 2 7 9 21 |
| mc2000006 | 4 6 7 21 | mcm000010 | 2 7 9 21 |
| mc2000034 | 7 17 21 | mcm000011 | 2 7 9 21 |
| mc2000035 | 4 7 21 | mcm000012 | 2 7 9 21 |
| mc2000064 | 1 2 7 21 | mcm000013 | 2 7 9 21 |
| mc2000109 | 21 | mcx000008 | 6 |
| mc2000123 | 7 | mcx000019 | 7 20 21 |
| mc2000130 | 1 7 | mcx000020 | 7 |
| mc2000135 | 10 21 | mcx000021 | 5 7 |
| mc2000138 | 7 21 | mcx000022 | 9 |
| mc2000141 | 4 7 | mcx000031 | 6 16 18 19 20 |
| mc2000159 | 7 9 13 23 | mcx000048 | 4 5 7 11 12 |
| mc2000175 | 21 | mcx000051 | 1 7 10 21 |
| mc2000194 | 7 13 17 21 22 23 24 | mcx000059 | 4 7 |
| mc2000200 | 3 7 17 21 | mcx000064 | 18 |
| mc2000251 | 7 8 10 18 21 | mcx000080 | 9 10 12 21 |
| mc2000258 | 1 7 | mcx000120 | 9 21 |
| mc2000278 | 1 6 7 | mcx000122 | 21 |
| mc2000296 | 7 10 13 15 21 | mcx000150 | 3 4 7 21 |
| mc2000302 | 8 | mcx000159 | 4 |
| mc2000310 | 5 | mcx000179 | 6 7 10 21 24 |
| mc2000320 | 4 7 | mcx000182 | 10 12 21 |
| mc2000321 | 7 | mcx000191 | 8 |
| mc2000345 | 9 10 21 | mcx000236 | 7 21 |
| mc2000348 | 1 7 10 13 21 23 24 | mcx000246 | 13 21 |
| mc2000366 | 7 9 21 | mcx000250 | 1 7 11 13 21 |
| mc2000380 | 7 | mcx000260 | 21 |
| mc2000390 | 7 10 15 21 | mcx000272 | 7 |
| mc2000392 | 13 | mcx000288 | 5 21 |
| mc2000401 | 7 12 15 16 20 21 | mcx000307 | 7 |
| mc2000405 | 7 9 | mcx000308 | 5 7 21 |
| mc2000415 | 1 7 | mcx000317 | 4 7 21 |
| mc2000416 | 1 3 21 | mcx000320 | 4 7 21 |
| mc2000421 | 21 | mcx000331 | 7 21 |
| mc2000426 | 1 7 21 | mcx000335 | 4 7 21 |
| mc2000427 | 3 7 8 10 | mcx000400 | 1 10 21 |
| mc2000432 | 7 10 20 21 | mcx000405 | 6 |
| mc2000440 | 7 9 12 17 21 | mcx000408 | 15 |
| mc2000463 | 4 7 21 | mcx000434 | 10 |
| mc2000469 | 3 4 7 21 | mcx000441 | 10 13 |
| mc2000474 | 7 15 16 21 | mcx000482 | 5 21 |
| mc2000487 | 12 | mcx000500 | 4 7 21 |
| mc2000490 | 7 9 21 | mcx000523 | 7 10 21 |
| mc2000496 | 7 | mcx000578 | 15 21 |
| mc2000498 | 7 8 | mcx000629 | 4 7 11 21 |
| mc2000508 | 21 | mcx000649 | 7 10 21 |
| mc2000538 | 7 | mcx000664 | 16 19 20 21 |
| mc2000551 | 7 | mcx000668 | 7 |
| mc2000555 | 1 | mcx000672 | 4 7 21 |
| mc2000576 | 1 6 21 | mcx000720 | 1 4 7 8 21 |
| mc2000583 | 21 | mcx000748 | 7 10 19 |
| mc2000584 | 7 | mcx000749 | 3 4 7 17 21 |
| mc2000593 | 8 10 | mcx000750 | 7 12 |
| mc2000629 | 1 | mcx000795 | 21 |
| mc2000643 | 1 2 11 | mcx000812 | 5 7 21 |
| mc2000675 | 1 13 20 | mcx000819 | 4 21 |
| mc2000711 | 7 8 9 11 12 21 | mcx000822 | 21 23 24 |
| mc2000712 | 4 7 21 | mcx000825 | 7 |
| mc2000716 | 10 | mcx000831 | 17 19 21 |
| mc2000721 | 4 5 7 9 18 21 24 | mcx000835 | 17 19 |
| mc2000723 | 12 13 16 17 20 24 | mcx000840 | 1 7 21 |
| mc2000738 | 1 4 6 7 8 10 11 13 15 16 18 19 20 21 | mcx000849 | 24 |
| mc2000747 | 5 7 21 | mcx000912 | 1 16 20 |
| mc2000850 | 10 12 13 21 24 | mcx000930 | 4 7 8 |
| mc2000856 | 4 7 | mcx000935 | 3 5 7 |
| mc2000861 | 3 11 17 21 | mcx000963 | 4 6 7 11 21 |
| mc2000880 | 4 7 21 | mcx000964 | 7 9 18 21 |
| mc2000881 | 7 | mcx000986 | 6 7 21 |
| mc2000915 | 1 6 7 10 21 | mcx000996 | 1 21 |
| mc2000931 | 7 12 17 | mcx000999 | 10 |
| mc2000944 | 3 4 7 17 21 | mcx001016 | 6 7 9 12 |
| mc2001004 | 9 | mcx001022 | 3 4 6 21 |
| mc2001010 | 7 9 12 21 | mcx001039 | 3 4 7 |
| mc2001054 | 4 7 | mcx001046 | 4 6 7 17 21 |
| mc2001072 | 7 12 13 19 23 | mcx001048 | 1 2 6 7 19 |
| mc2001076 | 4 6 7 8 10 17 21 | mcx001063 | 10 12 13 21 |
| mc2001083 | 18 21 24 | mcx001064 | 3 21 |
| mc2001089 | 1 6 7 13 17 21 24 | mcx001086 | 7 9 21 |
| mc2001108 | 2 9 13 21 24 | mcx001100 | 1 21 |
| mc2001113 | 7 9 13 21 22 24 | mcx001115 | 10 13 |
| mc2001127 | 7 8 10 21 24 | mcx001136 | 10 |
| mc2001132 | 1 3 6 7 10 17 21 | mcx001142 | 4 7 21 |
| mc2001154 | 4 7 21 | mcx001143 | 9 |
| mc2001162 | 7 8 10 | mcx001187 | 4 7 21 |
| mc2001187 | 4 7 15 16 20 21 | mcx001223 | 4 7 21 |
| mc2001192 | 4 7 15 16 21 | mcx001224 | 21 |
| mc2001232 | 7 15 16 21 | mcx001228 | 21 |
| mc2001254 | 7 21 | mcx001229 | 7 11 21 |
| mc2001285 | 7 10 12 19 20 | mcx001239 | 3 4 7 21 |
| mc2001286 | 1 | mcx001277 | 18 19 20 21 22 23 24 |
| mc2001298 | 1 7 21 | mcx001299 | 5 7 21 |
| mc2001314 | 3 | mcx001315 | 7 21 |
| mc2001340 | 10 | mcx001321 | 10 21 |
| mc2001356 | 6 10 12 15 21 | mcx001339 | 6 7 21 |
| mc2001360 | 4 7 21 | mcx001342 | 7 9 21 |
| mc2001361 | 1 7 | mcx001346 | 7 10 15 21 23 24 |
| mc2001369 | 21 24 | mcx001347 | 5 |
| mc2001372 | 21 | mcx001365 | 9 10 12 |
| mc2001377 | 6 7 8 21 | mcx001389 | 7 10 16 19 20 21 23 24 |
| mc2001497 | 5 6 7 21 | mcx001409 | 7 |
| mc2001512 | 10 13 21 | mcx001410 | 4 7 |
| mc2001532 | 10 13 | mcx001442 | 3 4 7 15 21 |
| mc2001645 | 1 | mcx001509 | 10 12 |
| mc2001648 | 2 7 9 21 | mcx001548 | 21 |
| mc2001649 | 7 | mcx001552 | 3 7 21 |
| mc2001652 | 4 7 21 | mcx001585 | 3 7 17 21 |
| mc2001674 | 4 7 21 | mcx001617 | 6 7 21 |
| mc2001678 | 21 | mcx001646 | 6 |
| mc2001695 | 12 21 24 | mcx001647 | 9 10 21 |
| mc2001696 | 9 21 | mcx001656 | 4 6 7 16 21 |
| mc2001715 | 10 13 21 | mcx001664 | 16 |
| mc2001733 | 7 | mcx001689 | 7 |
| mc2001734 | 1 21 | mcx001691 | 7 21 |
| mc2001754 | 4 6 7 8 17 21 | mcx001692 | 1 2 7 21 |
| mc2001778 | 10 | mcx001697 | 4 7 21 |
| mc2001785 | 21 | mcx001719 | 7 8 9 21 |
| mc2001789 | 6 21 | mcx001753 | 7 17 21 |
| mc2001792 | 9 12 21 24 | mcx001755 | 7 15 16 21 |
| mc2001817 | 6 | mcx001757 | 1 7 |
| mc2001835 | 7 10 21 | mcx001758 | 1 |
