# Supplementary material for: Predicting Functions of Proteins in Mouse Based on Weighted Protein-Protein Interaction Network and Protein Hybrid Properties
Source: PLoS One. 2011 Jan 19;6(1):e14556. doi: 10.1371/journal.pone.0014556 (PMC3023709; doi:10.1371/journal.pone.0014556)
Supplement: Table S4 — Test set for hybrid-property based method. The Mfun ID and Functional number (see Table 1) of proteins are shown. (0.16 MB DOC) [file pone.0014556.s004.doc]

Table S4. Test set for hybrid-property based method. The Mfun ID and Functional number (see Table 1) of proteins are shown.

| MfunGD ID | Functional number(s) | MfunGD ID | Functional number(s) |
| --- | --- | --- | --- |
| mc10000290 | 10 12 21 | mc2003452 | 4 7 21 |
| mc10000856 | 6 17 21 | mc2003460 | 4 7 21 |
| mc1000090 | 7 9 21 23 | mc3001007 | 6 10 13 |
| mc10000975 | 9 | mc3001019 | 7 12 21 |
| mc10001060 | 10 12 13 | mc3001175 | 7 19 |
| mc10001142 | 9 | mc3001351 | 10 13 |
| mc10001945 | 3 6 7 10 21 | mc3001400 | 1 2 |
| mc10001961 | 10 12 13 | mc3001571 | 7 |
| mc10002008 | 10 13 | mc3001899 | 6 7 |
| mc10002025 | 10 13 | mc3002102 | 10 21 |
| mc1000376 | 1 10 | mc3002149 | 1 7 21 |
| mc1000824 | 1 7 21 | mc3002251 | 21 |
| mc1000829 | 6 7 10 | mc4000148 | 7 |
| mc1001260 | 10 12 13 | mc4000467 | 10 13 21 |
| mc1001392 | 8 | mc4000572 | 1 21 |
| mc1001808 | 6 12 13 21 | mc4000575 | 4 7 21 |
| mc1001928 | 8 10 | mc4000621 | 24 |
| mc1002063 | 7 17 21 | mc4001526 | 10 13 |
| mc1002316 | 10 13 | mc4002265 | 7 21 |
| mc1002333 | 13 21 | mc4002444 | 7 |
| mc1002338 | 10 13 | mc5000603 | 7 10 21 |
| mc1002347 | 10 13 | mc5001032 | 6 |
| mc1002349 | 10 13 | mc5001047 | 1 11 21 |
| mc1002351 | 10 13 | mc5001645 | 1 6 8 10 |
| mc11000220 | 1 2 3 6 7 9 10 15 21 | mc5001663 | 7 |
| mc11000409 | 7 12 16 20 21 | mc5001910 | 7 21 |
| mc11000687 | 7 11 21 | mc5002241 | 7 |
| mc11000692 | 10 13 | mc5002455 | 7 |
| mc11000750 | 4 7 21 | mc6000269 | 7 21 |
| mc11000753 | 4 7 21 | mc6000301 | 6 21 24 |
| mc11000754 | 4 7 21 | mc6000353 | 1 7 11 21 |
| mc11000756 | 4 7 21 | mc6000461 | 10 13 |
| mc11000947 | 10 13 | mc6000527 | 10 13 21 |
| mc11000959 | 10 13 | mc6000544 | 10 13 |
| mc11001389 | 10 13 | mc6000580 | 4 7 21 |
| mc11001415 | 10 13 | mc6000621 | 7 |
| mc11001417 | 10 12 13 21 | mc6000677 | 9 10 |
| mc11001545 | 1 | mc6001347 | 10 12 |
| mc11001667 | 3 15 | mc6001628 | 7 9 17 21 |
| mc11002091 | 10 21 | mc6001705 | 10 13 |
| mc11002342 | 1 7 | mc6001747 | 4 7 21 |
| mc11002758 | 4 21 | mc6001971 | 6 21 |
| mc12000557 | 6 9 | mc6002049 | 10 12 13 21 24 |
| mc12000645 | 7 | mc6002052 | 10 12 13 21 24 |
| mc12000880 | 7 17 21 | mc6002053 | 10 12 13 21 24 |
| mc12001229 | 8 21 | mc6002157 | 1 7 10 21 |
| mc12001624 | 3 9 21 24 | mc6002325 | 10 12 13 |
| mc13000310 | 4 7 21 | mc6002369 | 10 |
| mc13000505 | 8 | mc6002373 | 10 |
| mc13001062 | 7 10 12 21 24 | mc7000103 | 7 21 |
| mc13001087 | 7 21 | mc7000107 | 10 13 |
| mc13001137 | 6 7 21 | mc7000130 | 4 7 21 |
| mc13001433 | 9 12 21 22 24 | mc7000144 | 10 |
| mc13001844 | 4 7 21 | mc7000182 | 7 21 |
| mc13001851 | 7 21 | mc7000198 | 10 21 |
| mc13001853 | 4 7 21 | mc7000398 | 7 21 |
| mc14000005 | 7 12 16 17 21 | mc7000400 | 4 7 21 |
| mc14000374 | 6 7 17 21 | mc7000577 | 7 8 12 13 15 17 18 19 21 22 23 |
| mc14000628 | 10 13 | mc7000607 | 4 7 21 |
| mc14000649 | 1 3 7 | mc7000608 | 4 7 21 |
| mc14000814 | 6 | mc7000611 | 4 7 21 |
| mc14001032 | 9 21 | mc7000877 | 6 21 |
| mc14001326 | 7 10 12 16 19 20 21 23 24 | mc7001071 | 10 |
| mc15000178 | 7 12 17 21 | mc7001078 | 10 |
| mc15000306 | 5 | mc7001558 | 10 13 |
| mc15000836 | 7 21 | mc7001578 | 10 13 |
| mc15001222 | 21 23 24 | mc7001580 | 10 13 |
| mc15001420 | 10 13 | mc7001581 | 10 13 |
| mc15001538 | 7 17 20 | mc7001830 | 10 13 |
| mc16000359 | 1 7 21 | mc7001847 | 10 13 |
| mc16000373 | 5 7 21 | mc7001879 | 10 13 |
| mc16000541 | 8 9 21 | mc7001880 | 10 13 |
| mc16000945 | 10 13 | mc7001886 | 10 13 |
| mc16001073 | 7 21 | mc7001891 | 10 13 |
| mc16001188 | 21 | mc7001894 | 10 13 |
| mc16001215 | 21 | mc7001902 | 10 13 |
| mc16001353 | 5 6 7 21 | mc7001911 | 10 13 21 |
| mc17000281 | 10 21 | mc7001964 | 10 13 |
| mc17000282 | 10 21 | mc7001995 | 10 13 |
| mc17000290 | 10 21 | mc7001998 | 10 13 |
| mc17000291 | 10 21 | mc7002007 | 10 13 |
| mc17000294 | 4 7 21 | mc7002010 | 7 9 10 13 |
| mc17000668 | 4 7 21 | mc7002061 | 10 13 21 |
| mc17000911 | 10 13 | mc7002103 | 10 12 13 |
| mc17000924 | 10 13 | mc7002115 | 10 13 |
| mc17000925 | 10 13 | mc7002328 | 7 |
| mc17000927 | 10 13 | mc7002331 | 7 |
| mc17000937 | 10 13 | mc7002498 | 7 21 |
| mc17000992 | 7 10 21 | mc7002507 | 4 7 21 |
| mc17001274 | 3 4 7 17 21 | mc7002559 | 7 21 |
| mc17001411 | 7 9 10 | mc7002773 | 10 13 |
| mc17001506 | 21 | mc7002794 | 10 13 |
| mc17001839 | 7 21 | mc7002975 | 4 7 21 |
| mc18000400 | 10 | mc7002990 | 10 |
| mc18000545 | 7 | mc7003032 | 4 7 21 |
| mc18000588 | 12 19 21 23 | mc8000277 | 11 12 13 21 |
| mc18000708 | 6 | mc8001577 | 1 11 21 |
| mc18000831 | 1 2 7 21 | mc8001594 | 1 21 |
| mc18001035 | 7 10 13 21 | mc8002130 | 4 7 21 |
| mc18001102 | 7 9 10 21 | mc8002143 | 4 7 21 |
| mc19000351 | 10 13 | mc9000124 | 7 9 10 |
| mc19000364 | 10 13 | mc9000178 | 10 13 |
| mc19000416 | 10 13 | mc9000219 | 7 21 |
| mc19000425 | 10 13 | mc9000293 | 7 21 |
| mc19000924 | 1 7 | mc9000327 | 4 7 21 |
| mc2000533 | 10 | mc9000594 | 10 13 |
| mc2000632 | 4 7 17 21 | mc9000627 | 10 13 |
| mc2000701 | 10 13 | mc9000640 | 10 13 |
| mc2001389 | 10 13 | mc9000776 | 7 9 |
| mc2001391 | 10 13 | mc9001032 | 9 21 |
| mc2001397 | 10 13 | mc9001715 | 4 7 20 21 |
| mc2001410 | 10 13 | mc9002055 | 1 7 |
| mc2001431 | 10 13 | mc9002186 | 3 7 |
| mc2001437 | 10 13 | mc9002254 | 10 11 12 15 21 |
| mc2001468 | 10 12 13 | mc9002261 | 4 7 21 |
| mc2001473 | 10 13 | mcx000251 | 4 7 21 |
| mc2001486 | 10 13 | mcx000431 | 10 13 |
| mc2001569 | 10 13 | mcx000437 | 10 13 |
| mc2001588 | 10 13 | mcx000655 | 24 |
| mc2001665 | 7 9 21 23 24 | mcx000673 | 1 3 7 11 21 |
| mc2001989 | 7 13 17 21 23 | mcx001210 | 7 17 20 |
| mc2002735 | 6 7 8 13 17 21 23 | mcx001446 | 1 2 7 21 |
| mc2003420 | 10 13 | mcx001782 | 10 13 |
