# Supplementary material for: Predicting Functions of Proteins in Mouse Based on Weighted Protein-Protein Interaction Network and Protein Hybrid Properties
Source: PLoS One. 2011 Jan 19;6(1):e14556. doi: 10.1371/journal.pone.0014556 (PMC3023709; doi:10.1371/journal.pone.0014556)
Supplement: Table S5 — The 90 optimized hybrid features generated by IFS procedure from a total of 132 features. (0.11 MB DOC) [file pone.0014556.s005.doc]

Table S5. The 90 optimized hybrid features generated by IFS procedure from a total of 132 features.

| Order | Feature | Subtypes of protein hybrid properties |
| --- | --- | --- |
| 1 | composition of H | Polarity |
| 2 | distribution of H | Normalized Van Der Waals Volume |
| 3 | transition of HC | Secondary Structure |
| 4 | k | Amino Acids Composition |
| 5 | transition of HC | Normalized Van Der Waals Volume |
| 6 | distribution of H | Hydrophobicity |
| 7 | distribution of H | Polarity |
| 8 | distribution of H | Solvent Accessibility |
| 9 | distribution of H | Secondary Structure |
| 10 | transition of HE | Solvent Accessibility |
| 11 | s | Amino Acids Composition |
| 12 | t | Amino Acids Composition |
| 13 | distribution of H | Secondary Structure |
| 14 | composition of H | Solvent Accessibility |
| 15 | c | Amino Acids Composition |
| 16 | transition of EC | Secondary Structure |
| 17 | distribution of E | Secondary Structure |
| 18 | distribution of E | Hydrophobicity |
| 19 | v | Amino Acids Composition |
| 20 | distribution of C | Polarizability |
| 21 | f | Amino Acids Composition |
| 22 | w | Amino Acids Composition |
| 23 | distribution of C | Polarity |
| 24 | p | Amino Acids Composition |
| 25 | distribution of H | Polarity |
| 26 | l | Amino Acids Composition |
| 27 | r | Amino Acids Composition |
| 28 | distribution of H | Hydrophobicity |
| 29 | distribution of C | Secondary Structure |
| 30 | h | Amino Acids Composition |
| 31 | composition of C | Polarity |
| 32 | distribution of C | Normalized Van Der Waals Volume |
| 33 | i | Amino Acids Composition |
| 34 | distribution of E | Polarizability |
| 35 | distribution of C | Secondary Structure |
| 36 | distribution of C | Polarizability |
| 37 | y | Amino Acids Composition |
| 38 | distribution of H | Normalized Van Der Waals Volume |
| 39 | distribution of E | Normalized Van Der Waals Volume |
| 40 | d | Amino Acids Composition |
| 41 | distribution of H | Solvent Accessibility |
| 42 | composition of E | Polarizability |
| 43 | composition of H | Secondary Structure |
| 44 | distribution of H | Normalized Van Der Waals Volume |
| 45 | a | Amino Acids Composition |
| 46 | transition of HE | Hydrophobicity |
| 47 | distribution of H | Polarizability |
| 48 | distribution of C | Secondary Structure |
| 49 | q | Amino Acids Composition |
| 50 | distribution of C | Hydrophobicity |
| 51 | transition of HE | Polarizability |
| 52 | composition of E | Secondary Structure |
| 53 | m | Amino Acids Composition |
| 54 | distribution of E | Secondary Structure |
| 55 | distribution of H | Solvent Accessibility |
| 56 | distribution of H | Polarizability |
| 57 | transition of EC | Hydrophobicity |
| 58 | distribution of E | Polarizability |
| 59 | distribution of C | Polarity |
| 60 | n | Amino Acids Composition |
| 61 | distribution of C | Hydrophobicity |
| 62 | distribution of C | Secondary Structure |
| 63 | distribution of H | Hydrophobicity |
| 64 | g | Amino Acids Composition |
| 65 | distribution of C | Normalized Van Der Waals Volume |
| 66 | composition of C | Hydrophobicity |
| 67 | distribution of E | Polarizability |
| 68 | distribution of E | Secondary Structure |
| 69 | distribution of E | Polarity |
| 70 | distribution of E | Normalized Van Der Waals Volume |
| 71 | e | Amino Acids Composition |
| 72 | distribution of E | Normalized Van Der Waals Volume |
| 73 | transition of EC | Polarizability |
| 74 | distribution of C | Normalized Van Der Waals Volume |
| 75 | distribution of H | Polarity |
| 76 | distribution of H | Polarizability |
| 77 | composition of C | Secondary Structure |
| 78 | distribution of C | Polarity |
| 79 | distribution of E | Hydrophobicity |
| 80 | transition of EC | Polarity |
| 81 | transition of HE | Normalized Van Der Waals Volume |
| 82 | distribution of H | Solvent Accessibility |
| 83 | composition of E | Normalized Van Der Waals Volume |
| 84 | distribution of H | Normalized Van Der Waals Volume |
| 85 | distribution of C | Polarity |
| 86 | distribution of H | Secondary Structure |
| 87 | transition of HE | Polarity |
| 88 | transition of HC | Polarizability |
| 89 | distribution of H | Solvent Accessibility |
| 90 | distribution of E | Polarity |
